# Systematic review and bayesian network meta-analysis: comparative efficacy and safety of six commonly used biologic therapies for moderate-to-severe Crohn’s disease

**DOI:** 10.3389/fphar.2024.1475222

**Published:** 2025-01-09

**Authors:** Haohang Su, Shengwei Xiao, Zhiqing Liang, Tianrong Xun, Jinfang Zhang, Xixiao Yang

**Affiliations:** ^1^ Department of Pharmacy, Shenzhen Hospital, Southern Medical University, Shenzhen, China; ^2^ School of Pharmaceutical Sciences, Southern Medical University, Guangzhou, China; ^3^ Cancer Center, Shenzhen Hospital (Futian) of Guangzhou University of Chinese Medicine, Shenzhen, China; ^4^ Shenzhen Traditional Chinese Medicine Oncology Medical Center, Shenzhen, China; ^5^ Shenzhen Clinical Research Center for Digestive Disease, Shenzhen, China

**Keywords:** bayesian network meta-analysis, Crohn’s disease, biologic agents, efficacy, safety, GRADE

## Abstract

**Background:**

In contrast to previous network meta-analysis using classical frequentist methods, we evaluated the efficacy and safety of six frequently-used biologics through a Bayesian method.

**Methods:**

Web of Science, Scopus, CENTRAL, ClinicalTrials.gov and ICTRP were searched to collect randomized controlled trials (RCTs) in adults with moderate-to-severe Crohn’s disease, comparing Infliximab, Adalimumab, Certolizumab pegol, Ustekinumab, Risankizumab, or Vedolizumab, relative to placebo or an active comparator for induction of clinical response (two different definitions) and maintenance of clinical remission. A random-effects model was performed with rankings according to the surface under cumulative ranking curve (SUCRA) probability. Finally, we completed sensitivity and consistency analyses, and evaluated the certainty of evidence through GRADE working group guidance.

**Results:**

We identified 22 and 20 RCTs for induction and maintenance therapy, respectively. Infliximab combined with azathioprine was most effective for inducing clinical response in TNF (tumor necrosis factor) antagonist-naïve patients. For TNF antagonist-experienced patients, Ustekinumab (SUCRA 86.19) and Risankizumab (SUCRA 62.56) have the largest SUCRA in induction of clinical response. Risankizumab has the lowest risk of adverse events (SUCRA 84.81), serious adverse events (SUCRA 94.23), and serious infections (SUCRA 79.73) in induction therapy. Adalimumab and the 10 mg/kg regimen of Infliximab rank highest for maintaining clinical remission.

**Conclusion:**

This analysis suggests that Infliximab in combination with azathioprine may be preferred biologic agents for induction therapy in TNF antagonist-naïve patients. For TNF antagonist-experienced patients, Ustekinumab and Risankizumab may be preferred biologic agents for induction therapy. Risankizumab potentially has the lowest safety risk worth exploring in induction therapy. Adalimumab and the 10 mg/kg regimen of Infliximab have maintenance efficacy benefits for responders to induction therapy.

**Systematic Review Registration:**

https://www.crd.york.ac.uk/prospero/display_record.php?RecordID=458609, Identifier CRD42023458609.

## 1 Introduction

Crohn’s disease (CD) is a chronic inflammatory gastrointestinal disease characterized by multifactorial pathogenesis and refractoriness. Current therapies focus on reducing intestinal inflammation. As the discovery of novel drug targets and the increasing number of patients with CD refractory to conventional therapies, biologic therapies have gradually come to the forefront. However, with the introduction and clinical availability of infliximab (Knight et al., 1993; van Dullemen et al., 1995), the first tumor necrosis factor α (TNFα) antagonist for the treatment of CD, problems with its efficacy emerged. Among CD patients treated with TNFα antagonists, approximately two-thirds of the patients treated with TNFα antagonists are either primary non-response or loss of response (LOR) over time (Sandborn et al., 2012). Consequently, researchers have developed several new biologic agents based on different immune mechanisms to address the non-response to TNFα antagonists in CD patients: interleukin antagonists like Ustekinumab and Risankizumab (Benson et al., 2011; Singh et al., 2015), anti-integrin monoclonal antibody like Vedolizumab (Soler et al., 2009).

With the popularization of biologics with differing efficacy and safety profiles, clinicians have more therapeutic options when they make medical treatment plans for CD patients. However, they also face tough choices because of the uncertainty of the appropriate positioning of different agents in the disease course. In addition, it is a challenge for clinicians to determine the best pharmacotherapy for a patient who represents a primary non-response or secondary loss of response despite having undergone biologic therapy. These choices involve comprehensive decision-making analysis of the clinical rational medication incorporating patients’ preferences as well as diagnosis and medical recommendations from clinicians. Therefore, researchers collect various clinical data through clinical trials and experiment with different treatment options to achieve clinical rational medication.

Nevertheless, today’s redundant and variable-quality clinical trial results inevitably tire out the clinicians in need, there is a particular demand for systematic reviews to analyze valuable clinical trial results to provide a clear and concise clinical evidence base. Network Meta-Analysis (NMA), a statistical method for comparing multiple treatment options simultaneously through an integrated analysis of both direct and indirect evidence from RCTs, has provided a wealth of evidence on the safety and efficacy of therapies lacking head-to-head RCTs data for many clinical guidelines and Health Technology Assessments (HTAs).

In NMA, Bayesian methods offer several advantages over classical frequentist methods. First, Bayesian methods provide greater flexibility in handling uncertainty by incorporating prior beliefs through prior probability distributions and offering a more comprehensive representation of uncertainty in parameter estimation. Second, Bayesian methods offer advantages in dealing with heterogeneity, allowing for a more nuanced understanding of variability across studies and facilitating the use of complex models, thereby capturing data structure and patterns more effectively (Sutton and Abrams, 2001; Dias et al., 2014a; Higgins and Cochrane, 2019).

With new RCTs being initiated or completed on a daily basis, it is necessary to update the evidence collected, complied and presented by the NMA in order to provide the up-to-date evaluations. In this study, we conducted an updated systematic review and NMA with a Bayesian consistency DerSimonian-Laird random-effects model (Borenstein et al., 2010; Kanters, 2022; Watt and Del Giovane, 2022), using Markov chain Monte Carlo (MCMC) simulation, comparing the relative efficacy and safety of Infliximab, Adalimumab and Certolizumab pegol (three TNFα antagonists), Ustekinumab and Risankizumab (two interleukin antagonists), Vedolizumab (an anti-integrin monoclonal antibody), biosimilars (CT-P13 and BI 695501), and TNFα antagonists combined with immunosuppressants, for adult moderate-to-severe CD patients stratified by history of TNF antagonist treatment in the induction and maintenance therapy. We used Grading of Recommendations Assessment, Development, and Evaluation (GRADE) working group guidance to evaluate the certainty of evidence (Puhan et al., 2014).

## 2 Methods

The methods and recommendations of the PRISMA (Preferred Reporting Items for Systematic Reviews and Meta-Analysis) extension statement for NMA for healthcare interventions, and the Cochrane Handbook were used to conduct this systematic review (Hutton et al., 2015; Higgins and Cochrane, 2019). Good research practices from the report of ISPOR (International Society for Pharmacoeconomics and Outcomes Research) for conducting indirect treatment comparisons and NMA for healthcare decision making were referenced (Hoaglin et al., 2011). The study protocol has been prospectively registered in PROSPERO (International Prospective Register of Systematic Reviews, www.crd.york.ac.uk/prospero). The registration number is CRD42023458609.

### 2.1 Search strategy

A comprehensive search of multiple electronic databases, including PubMed, Embase, Web of Science, Scopus, CENTRAL (Cochrane Central Register of Controlled Trials), ClinicalTrials.gov and ICTRP (International Clinical Trials Registry Platform), from inception to 8 June 2023, without language or geographic area restrictions were conducted. MeSH/Emtree words combined with free words were used for the literature search (see Supplementary Table S1 in the Supplementary File 1 [SF1], full search strategy).

### 2.2 Selection criteria

Two investigators independently searched the study titles, abstracts and full texts of relevant studies, to identify articles of interest based on the following inclusion and exclusion criteria.

The selection criteria were in strict accordance with the PICOS (Patients, Intervention, Comparators, Outcomes, Study designs) principle. Study included in this NMA were phase Ⅱ or Ⅲ RCTs meeting the following criteria: (1) including adult (18–75 years of age) patients with moderate-to-severe CD (defined by CDAI 220–450); (2) receiving a biologic treatment with TNFα antagonists (Infliximab, Adalimumab or Certolizumab pegol), anti-interleukin (Ustekinumab, Risankizumab), anti-integrin (Vedolizumab), either alone or in combination with immunosuppressants, and their biosimilars, as the first-line biologic or after previous biologic exposure; (3) minimum duration of therapy of 4 weeks for trials reporting induction of response or remission in active disease and 22 weeks in trials reporting maintenance of response or remission; (4) compared to placebo or another medication for CD patients; (5) reporting induction of clinical remission (defined by a CDAI <150) or clinical response (defined by a reduction in the CDAI ≥70 points compared to baseline [CDAI-70], some studies defined the clinical response threshold of CDAI as 100 [CDAI-100], while some reported both CDAI-100 and CDAI-70), maintenance of remission or clinical response (among patients with clinical response to induction therapy in re-randomization trials and among all patients in treat-through trials), or safety (adverse events [AEs], serious adverse events [SAEs], and serious infections [SIs] as defined by the study authors). Only trials conducted in biologic and immunosuppressant naïve patients, in which patients were initiated on biologic monotherapy alone and compared with biologics and immunosuppressants in combination, were included as trials of combination therapy. In contrast, trials specifically enrolling immunosuppressant failed patients to evaluate a single biologic therapy or to compare two or more biologic therapies were included as trials of respective biologic monotherapy.

We excluded studies where: (1) trials of biologics to prevent postoperative recurrence; (2) trials comparing biologic treatment with surgical treatment; (3) pilot studies; (4) trials comparing therapeutic drug monitoring (TDM) with standard therapy; (5) trials with treatment failure or occurrence of complications as the primary end point; (6) trials of the effect of differences in baseline characteristics (C-reactive protein, prior anti-TNFα therapy, severity and disease distribution) on efficacy; (7) trials with unspecified interventions (a class but not a precise medication was specifical); (8) trials of the withdrawal of medications; (9) trials comparing the optimized dosing regimen by pharmacokinetic dashboard (combined with TDM in the OPTIMIZE [Proactive infliximab optimization using a pharmacokinetic dashboard versus standard of care in patients with Crohn’s disease] trial) with the standard dosing regimen; (10) trials of the effect of different preoperative medications on postoperative complications; (11) trials of dosing interval lengthening in maintenance phase; (12) early terminated trials; (13) trials without results; (14) trials included patients with other subtypes of inflammatory bowel diseases (IBD, CD is a subtypes of IBD); (15) reviews, letters, conference abstracts, animal studies; (16) trials of novel therapies without reported phase Ⅲ data; (17) trials of advanced therapies not frequently used for the treatment of moderate-to-severe CD in clinical practice.

### 2.3 Data analysis

Two study investigators (Haohang Su and Shengwei Xiao) independently rated the risk of bias of included RCTs using RoB 2 (version 2 of the Cochrane risk-of-bias tool for randomized trials) (Sterne et al., 2019). Any discrepancies were resolved by consensus or in consultation with a third reviewer (Xixiao Yang). Five domains (randomization process, deviations from intended interventions, Missing outcome data, measurement of the outcome, selection of the reported result) were assessed separately as “low risk”, “high risk”, or “some concerns” and analyzed together to produce a final risk of bias judgement for each study (see Supplementary Figure S1 in SF1, traffic light plot of the risk of bias assessment). A sensitivity analysis was conducted excluding the inferior quality trials.

A standardized data extraction form was used to capture study-, participant-, disease-, and treatment-related characteristics, and was done independently by two investigators. Any discrepancies were resolved by consensus or in consultation with the third reviewer. The most complete report of trial data, based on intent-to-treat analysis principles for efficacy outcomes and last observation carried forward for safety outcomes, was used for extraction if study results were reported in multiple publications. In addition, Individual patient-level data was not sought in this review.

The primary efficacy outcome chosen in induction trials included achievement of clinical response (defined by a reduction in the CDAI ≥70 [CDAI-70] or ≥100 [CDAI-100] points compared to baseline). Secondary outcome was achievement of clinical remission, defined by a CDAI <150. For maintenance trials, the primary efficacy outcome was maintenance of clinical remission, secondary outcomes were CDAI-70 and CDAI-100. The results of evaluations of CDAI-100 as the predominant for interpretation when discrepancies between the results of CDAI-70 and CDAI-100 in the evaluations of clinical response occurred (Sands et al., 2024). Both responder re-randomization and treat-through trials were included, although data informing remission rates among induction responders was preferentially used when available for the primary maintenance outcome in treat-through studies. A sensitivity analysis was conducted excluding treat-through trials. The safety outcomes evaluated in induction or maintenance trials were adverse events (AEs), serious adverse events (SAEs), and serious infections (SIs), as defined by the primary study authors (included the timepoint for the safety summary).

Data from a medication’s approved dosage and administration was used, but the included subcutaneous injections formulation of Vedolizumab (Vedolizumab SC) evaluated in the VISIBLE-2 trial is an exception because of its potential clinical advantage. In trials had multiple outcomes from different times, we extracted the data from week 6 for the induction phase (if data from week 6 were not available, the earliest data from week 4 onwards were extracted), and the latest result for the maintenance phase. Long-term open-label trials were not used. The denominator used in all trials was based on intent-to-treat analysis with dropouts or missing data treated as non-responders for remission and response outcomes; last-observation-carried-forward (LOCF) or observed-case (OC) was used for missing continuous values; missing categorical variables were imputed using non-responder imputation (NRI).

Other extracted covariables included location of trial conduct, number of trials centers or sites, sample size (included the number of TNF antagonist-naïve patients and total sample size), trial design (received medication, dosage regimen, duration of intervention, and randomization), dosage and schedule of active/comparator group, severity of CD at randomization, definition of outcomes, outcome timepoints, concomitant medications (divided into active group and placebo/comparator group, medications included aminosalicylates, immunosuppressants, and corticosteroids).

A Bayesian random-effects model was built given the anticipated differences between trials with respect to patient enrolment criteria, patient populations, outcome timepoint of evaluations and interventions (assessments of model fit are presented in the results). A sensitivity analysis was conducted changing the original random-effects model to a Mantel-Haenszel fixed-effect model. Pooled odds ratios (ORs) with 95% credible interval (CrI) were estimated, and statistical heterogeneity was evaluated using the I^2^ statistic. We also conducted a sensitivity analysis using relative risk (RR) as the effect size.

In the Bayesian model for evaluating the induction and maintenance of clinical remission in moderate-to-severe CD patients, the MCMC parameters were set to 4 chains, 20,000 adaptation iterations, 200,000 total iterations, burn-in iterations were half of total iterations rounded down and the thin parameter was 1. The priors on the baseline and relative treatment effects were defined as independent normal priors (used with mean 0 and standard deviation 15u, where u is the largest maximum likelihood estimator in single trials), an uniform distribution with range 0 to u was used as the prior on variance of relative treatment effects (van Valkenhoef et al., 2012). In the other evaluations, the total iterations were set to 50,000. A sensitivity analysis was conducted changing the original standard deviation of independent normal priors of the baseline treatment effects and relative treatment effects to 10u.

Statistical heterogeneity of these various outcomes was demonstrated by forest plots (see Supplementary Figures S2A–2E; Supplementary Figures S3–S7 in SF1, network plots and forest plots of direct comparisons). Small study effects and publication bias were examined by assessing for funnel plot asymmetry (see Supplementary Figures S8–S14 in SF1, all funnel plots). A sensitivity analysis was conducted excluding the trials which data points fell outside the 90% confidence interval in the funnel plots. Model fit was assessed by computing the Deviance Information Criteria (DIC) and producing a leverage plot, all leverage plots are presented in Supplementary Figures S15–S19 (Dias et al., 2014a). MCMC model convergence was assessed by Brooks-Gelman-Rubin diagnosis plot, computing shrink factor and potential scale reduction factor (PSRF), all assessment results of convergence are presented in Supplementary Figures S20–S24 in SF1. The relative ranking of agents for each outcome was expressed using rankogram (a kind of bar chart showing the probability of each treatment ranking in each rank) and the surface under the cumulative ranking curve (SUCRA). Higher SUCRA values correspond to better efficacy or safety outcomes for induction/maintenance (better means that the corresponding intervention is more effective or has lower safety risks). The relative effectiveness of biologics for each outcome was compared in league heat plot and forest plot. All plots of comparative evaluation results are presented in Supplementary Figures S25–S39 in SF1.

In inconsistency analysis, we plotted the posterior mean deviance contributions of the individual data points for the consistency model and the inconsistency model along with the line of equality (see Supplementary Figure S40 in SF1, all consistency vs. inconsistency plots) and analyzed the anomalous data points specifically (Dias et al., 2014b). Finally, we used GRADE working group guidance to evaluate the certainty of evidence (Puhan et al., 2014) (see Supplementary Tables S2–S6 in SF1). All data analysis described above were conducted using ‘BUGSnet’ package in R statistical software (version 4.3.1). (Beliveau et al., 2019).

## 3 Results

### 3.1 Search results

The search strategy yielded 16,252 citations, of which 3,214 were duplicates and removed. Of the remaining 13,038 records that were screened, 103 full text articles were reviewed 31 trials in 28 studies were eligible for inclusion (see Supplementary Figure S41 in SF1, flow diagram of selection). A total of 22 RCTs were involved in evaluations of induction efficacy and safety in patients with moderate-to-severe CD, of which 13 and 8 RCTs evaluated induction efficacy in TNF antagonist-naïve and -experienced patients, respectively. There were 20 RCTs evaluating maintenance efficacy and safety in patients with moderate-to-severe CD, including 11 and 6 RCTs evaluating maintenance efficacy in TNF antagonist-naïve and experienced patients, respectively.

Patient and trial-level characteristics for induction and maintenance are summarized in Supplementary Tables S7–S8 in SF1, five of the included trials were completed in a single country, and the rest were completed in multiple centers or sites. Patient inclusion and exclusion criteria varied among these trials, mainly manifested in severity of CD (3 RCTs were defined as CDAI 220–400, the DIAMOND trial was defined as CDAI ≥220 (Matsumoto et al., 2016), all others were defined as CDAI 220–450), TNF antagonists using history (8 RCTs excluded TNF antagonist-experienced patients and 15 RCTs included patients with prior failure of TNF antagonists, the other trials required conventional therapy failure as an inclusion criterion), and concomitant medications. On the comparator settings, one head-to-head, active-comparator trial (SEAVUE) comparing Ustekinumab with Adalimumab was identified (Sands et al., 2022); two head-to-head, active-comparator trials (the SONIC, DIAMOND trials) comparing biologic with combination therapy (biologic combined with azathioprine) were identified (Colombel et al., 2010; Matsumoto et al., 2016); and two three-arm trials (the ACCENT-1 and FORTIFY trials) comparing different doses of Infliximab or Risankizumab were identified (Hanauer et al., 2002; Ferrante et al., 2022). One placebo-controlled trial evaluating Vedolizumab SC was included, although subcutaneous injections formulation has not been approved (by U.S. Food and Drug Administration [FDA]) as an administration of Vedolizumab in the treatment of CD. Two head-to-head, biosimilar-comparator trials (Infliximab and Adalimumab were each compared with their biosimilars [CT-P13 and BI 695501]) were identified (Ye et al., 2019; Hanauer et al., 2021). All of the included trials were re-randomization trials, except for the SONIC, DIAMOND, SEAVUE, PRECISE-1 trials, which were treat-through trials. All induction or maintenance outcomes were uniformly evaluated based on standard definition of the CDAI, between weeks 4 and 10 for induction therapy phase and weeks 22 and 60 for maintenance phase, and the results of secondary outcomes are presented in Supplementary Result 1-4 in SF1.

### 3.2 Efficacy in overall patients with Crohn’s disease

#### 3.2.1 Induction therapy

A total of 22 RCTs including 6,427 moderate-to-severe CD patients evaluated induction clinical response (CDAI-70 and CDAI-100) after treatment with Infliximab (n = 3 trials, one biosimilar-comparator trial [CT-P13] and one combination-therapy-comparator trial [combined with azathioprine] were included) (Targan et al., 1997; Colombel et al., 2010; Ye et al., 2019), Adalimumab (n = 6 trials, one biosimilar-comparator trial [BI 695501] and one combination-therapy-comparator trial [combined with azathioprine] were included) (Hanauer et al., 2006; Sandborn et al., 2007c; Watanabe et al., 2012; Matsumoto et al., 2016; Chen et al., 2020; Hanauer et al., 2021), Certolizumab Pegol (n = 2 trials) (Sandborn et al., 2007a; Sandborn et al., 2011), Ustekinumab (n = 4 trials, included one active-comparator [Adalimumab] trial) (Sandborn et al., 2012; Feagan et al., 2016; Sands et al., 2022), Risankizumab (n = 3 trials) (Feagan et al., 2017; D'Haens et al., 2022), Vedolizumab (n = 4 trials) (Sandborn et al., 2013; Sands et al., 2014; Takeda, 2017; Watanabe et al., 2020).

**FIGURE 1:**
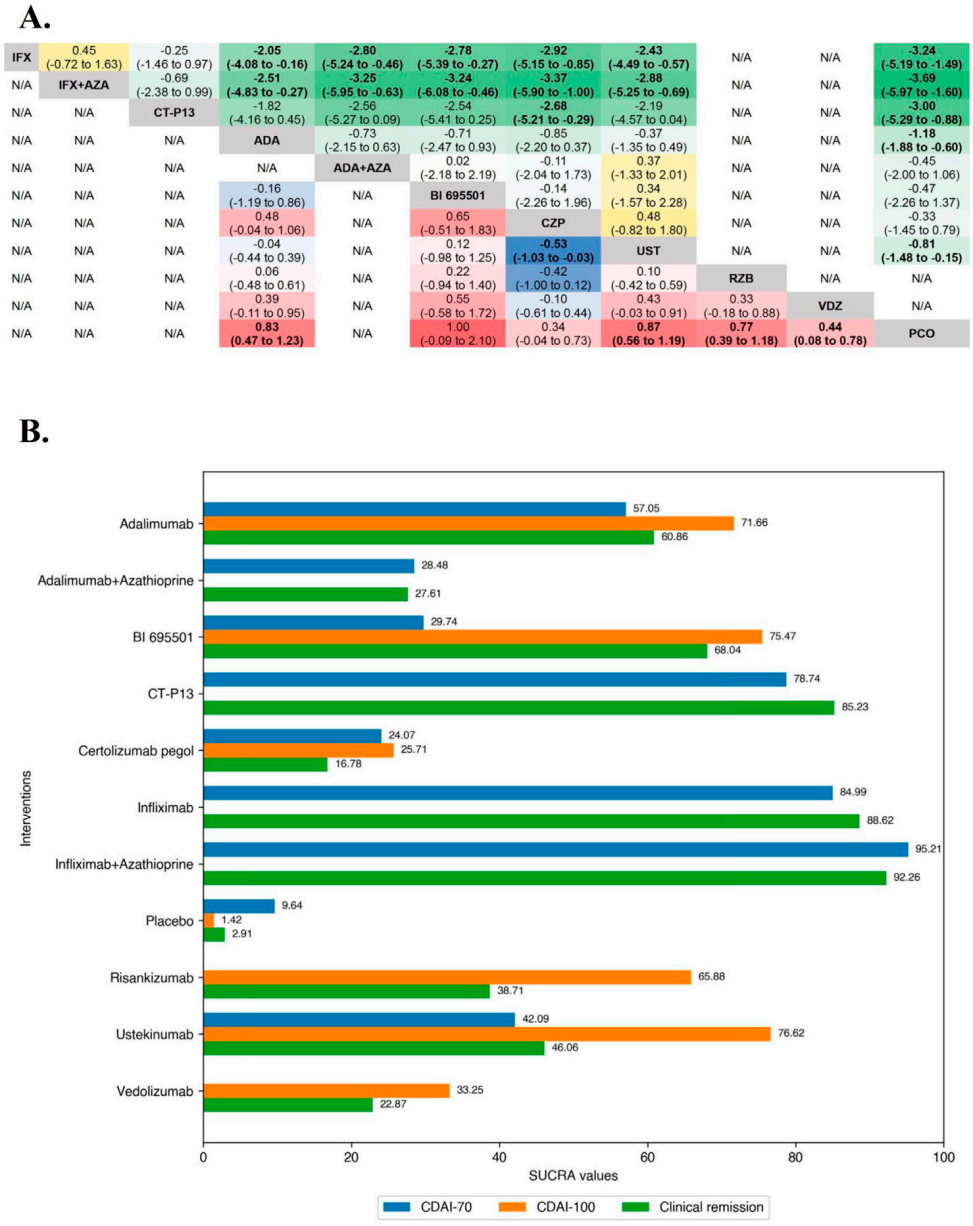
**(A)** SUCRA (the surface under cumulative ranking curve) values of each intervention in efficacy evaluations of induction therapy in overall patients with moderate-to-severe Crohn’s disease. SUCRA values are expressed on a scale from 0 to 100. The closer the value is to 100, the more likely the intervention is to be among the most effective. Conversely, the closer the value is to 0, the less effective the treatment is likely to be. **(B)** League heat plot of mixed comparisons of inducing clinical response (defined by a reduction in the CDAI [Crohn’s Disease Activity Index] ≥70 [CDAI-70] or ≥100 [CDAI-100] points compared to baseline) between included biologic agents in overall patients with moderate-to-severe Crohn’s disease, reporting odds ratios on the logarithmic scale (log OR) with 95% confidence intervals. Log OR greater or less than 0 are indicated in red/yellow or blue/green, respectively, in comparisons of inducing CDAI-100/CDAI-70, and the darker the color, the greater the odds ratios. Bold type represents statistically significant superiority/inferiority of the intervention over the comparator.

In the evaluation of inducing CDAI-70 in moderate-to-severe CD patients, funnel plots did not show evidence of publication bias or small study effects after including outcome timepoint as a moderator, and also suggests that this part of the heterogeneity caused by differences in outcome timepoint contributed to the asymmetry of the funnel plot. The Brooks-Gelman-Rubin diagnosis plot and the calculated PSRF indicated convergence of the MCMC sampling. On direct meta-analysis, all agents except Risankizumab and Vedolizumab, which CDAI-70 were not evaluated in included RCTs, were significantly superior to placebo for inducing CDAI-70. Neither Infliximab nor Adalimumab had significant superiority over their respective biosimilars or combinations with azathioprine. On network meta-analysis, Infliximab alone and Infliximab in combination with azathioprine were both significantly superior to Adalimumab, Ustekinumab, BI 695501, Adalimumab and azathioprine in combination, and Certolizumab Pegol. The evidence also supported a significant superiority of CT-P13 over Certolizumab Pegol (log OR 2.68 [95% CrI: 0.29, 5.21], Figure 1A). Overall, Infliximab in combination with azathioprine (SUCRA 95.21) and Infliximab alone (SUCRA 84.99) were ranked highest (Figure 1B).

**FIGURE 2:**
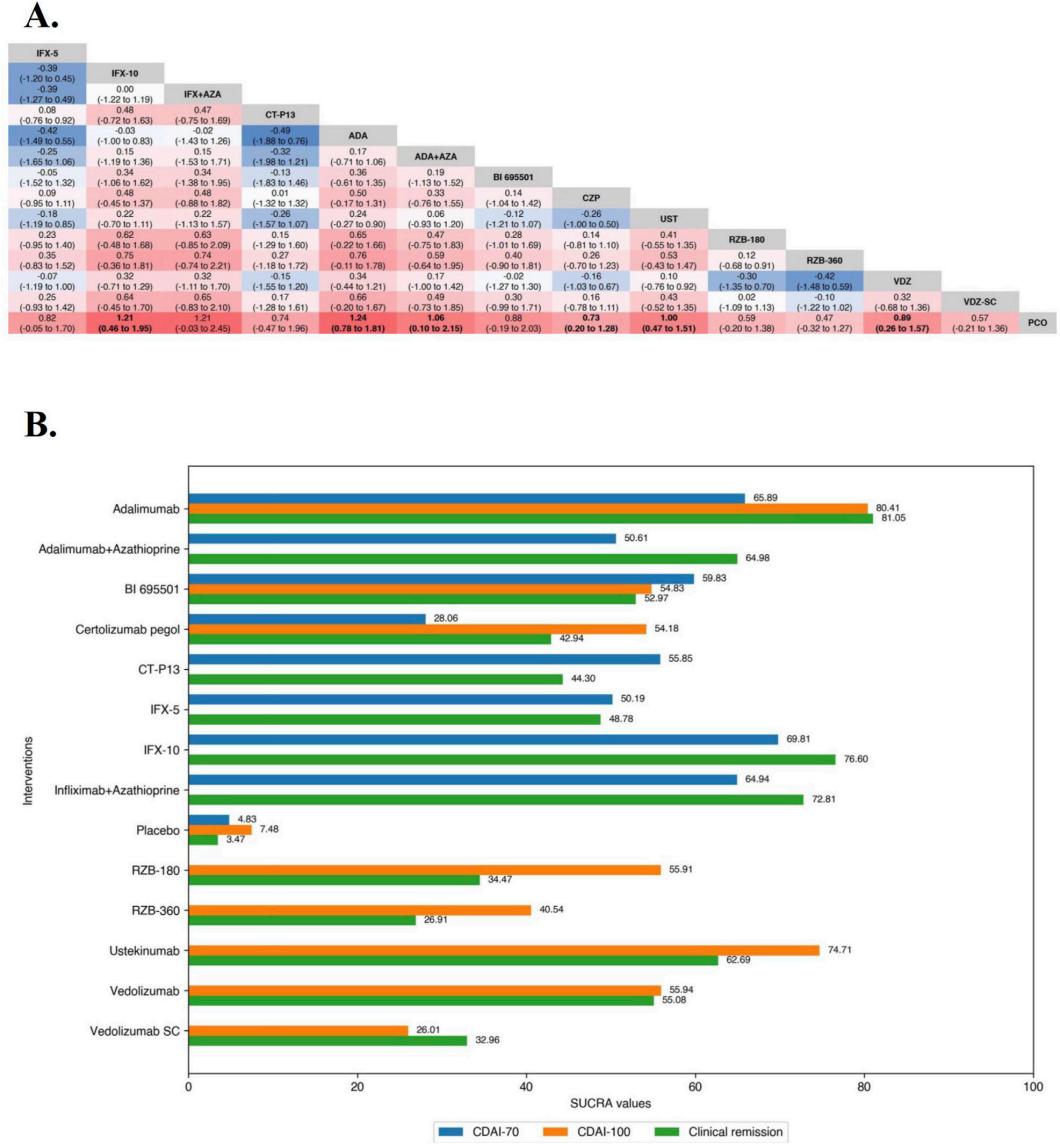
**(A)** SUCRA (the surface under cumulative ranking curve) values of each intervention in efficacy evaluation of maintenance therapy in overall patients with moderate-to-severe Crohn’s disease. SUCRA values are expressed on a scale from 0 to 100. The closer the value is to 100, the more likely the intervention is to be among the most effective. Conversely, the closer the value is to 0, the less effective the treatment is likely to be. **(B)** League heat plots of mixed comparisons of maintaining clinical remission (defined by a CDAI [Crohn’s Disease Activity Index] < 150) between included biologic agents in overall patients with moderate-to-severe Crohn’s disease, reporting odds ratios on the logarithmic scale (log OR) with 95% confidence intervals. Log OR greater or less than 0 are indicated in red or blue, respectively, in comparisons of maintaining clinical remission, and the darker the color, the greater the odds ratios. Bold type represents statistically significant superiority/inferiority of the intervention over the comparator.

In the evaluation of inducing CDAI-100 in moderate-to-severe CD patients, the results of funnel plot and model convergence assessments were within appropriate and controllable range. The leverage plot suggested that the SEAVUE trial may have contributed to the poor model fit and a sensitivity analysis was conducted excluding the SEAVUE trial. On direct meta-analysis, all agents except Infliximab (CDAI-100 were not evaluated in included RCTs) and Vedolizumab (OR 1.49 [95% CI: 0.94, 2.37]), were significantly superior to placebo for inducing CDAI-100. There was no benefit of Adalimumab over BI 695501 (OR 0.86 [95% CI: 0.35, 2.15]) for inducing CDAI-100, and similarly no benefit of Ustekinumab over Adalimumab (OR 1.09 [95% CI: 0.71, 1.67]). On network meta-analysis, Ustekinumab was significantly superior to Certolizumab Pegol (log OR 0.53 [95% CrI: 0.03, 1.03], Figure 1A). Ustekinumab (SUCRA 76.62), BI 695501 (SUCRA 75.47), and Adalimumab (SUCRA 71.66) were ranked highest (Figure 2B).

#### 3.2.2 Maintenance therapy

Overall, 20 RCTs including 4,834 patients with moderate-to-severe CD evaluated maintenance clinical remission. Therapies that were evaluated included Infliximab (n = 4 trials, included one biosimilar-, one combination-therapy-comparator trial and one three-arm trial [the ACCENT-1 trial]) (Rutgeerts et al., 1999; Hanauer et al., 2002; Colombel et al., 2010; Ye et al., 2019), Adalimumab (n = 6 trials, included one biosimilar-comparator trial and one combination-therapy-comparator trial) (Colombel et al., 2007; Sandborn et al., 2007b; Rutgeerts et al., 2012; Watanabe et al., 2012; Matsumoto et al., 2016; Hanauer et al., 2021), Certolizumab Pegol (n = 2 trials) (Sandborn et al., 2007a; Schreiber et al., 2007), Ustekinumab (n = 3 trials, one active-comparator [Adalimumab] trial was included) (Sandborn et al., 2012; Feagan et al., 2016; Ferrante et al., 2022; Sands et al., 2022), Risankizumab (n = 1 trial, one three-arm trial [the FORTIFY trial]) (Ferrante et al., 2022), Vedolizumab (n = 4 trials, included one trial evaluating Vedolizumab SC) (Sandborn et al., 2013; Takeda, 2017; Watanabe et al., 2020; Vermeire et al., 2022).

In the evaluation of maintaining clinical remission in moderate-to-severe CD patients, funnel plots did not show evidence of publication bias or small study effects, the Brooks-Gelman-Rubin diagnosis plot and the calculated PSRF indicated convergence of the MCMC sampling. On direct meta-analysis, all agents included Vedolizumab SC (OR 1.77 [95% CI: 1.15, 2.71]) were significantly superior to placebo. Comparisons between treatment groups of biologic agents (5 mg/kg maintenance regimen of Infliximab [IFX-5] vs. 10 mg/kg maintenance regimen of Infliximab [IFX-10] in the ACCENT-1 trial and 180 mg maintenance regimen of Risankizumab [RZB-180] vs. 360 mg maintenance regimen of Risankizumab [RZB-360] in the FORTIFY trial) were not statistically different in either of the two included three-arm trials. Neither the corresponding biosimilar nor combination of azathioprine had a significant effect on the efficacy of infliximab or adalimumab compared with monotherapy. There was no benefit of Ustekinumab over Adalimumab (OR 1.18 [95% CI: 0.78, 1.79]). On network meta-analysis, Adalimumab (SUCRA 81.05), IFX-10 (SUCRA 76.60), Infliximab in combination with azathioprine (SUCRA 72.81), Adalimumab in combination with azathioprine (SUCRA 64.98) were ranked highest (Figure 2).

### 3.3 Efficacy in TNF antagonist-naïve patients with Crohn’s disease

#### 3.3.1 Induction therapy

A total of 14 induction RCTs including 3,050 TNF antagonist-naïve patients with moderate-to-severe CD evaluated induction clinical response (CDAI-70 and CDAI-70) after treatment with Infliximab (n = 3 trials, included one biosimilar-, one combination-therapy-comparator trial) (Targan et al., 1997; Colombel et al., 2010; Ye et al., 2019), Adalimumab (n = 4 trials, included one combination-therapy-comparator trial) (Hanauer et al., 2006; Watanabe et al., 2012; Matsumoto et al., 2016; Chen et al., 2020), Certolizumab Pegol (n = 2 trials) (Sandborn et al., 2007a; Sandborn et al., 2011), Ustekinumab (n = 2 trials, included one active-comparator [Adalimumab] trial) (Feagan et al., 2016; Sands et al., 2022), Vedolizumab (n = 3 trials) (Sandborn et al., 2013; Sands et al., 2014; Watanabe et al., 2020).

**FIGURE 3:**
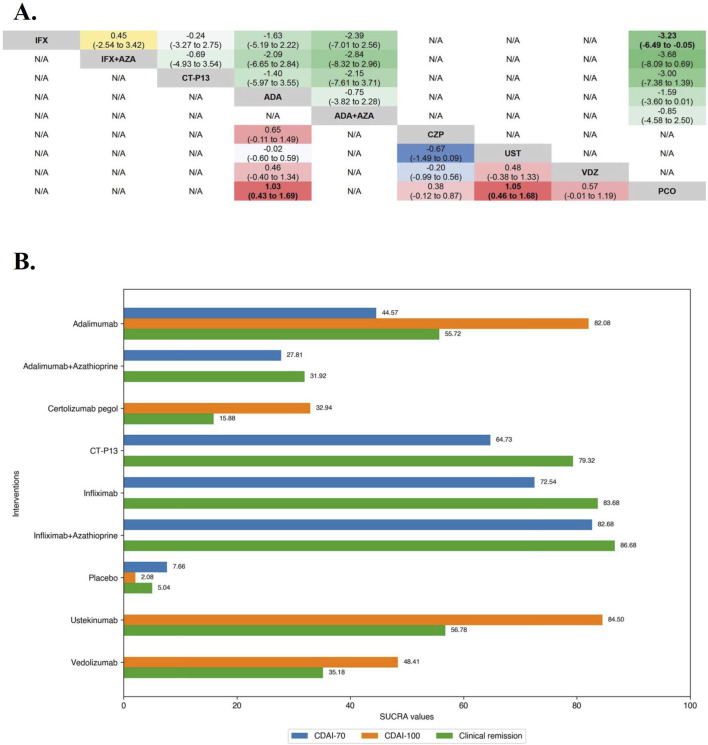
**(A)** SUCRA (the surface under cumulative ranking curve) values of each intervention in efficacy evaluations of induction therapy in tumor necrosis factor antagonist-naïve patients with moderate-to-severe Crohn’s disease. SUCRA values are expressed on a scale from 0 to 100. The closer the value is to 100, the more likely the intervention is to be among the most effective. Conversely, the closer the value is to 0, the less effective the treatment is likely to be. **(B)** League heat plots of mixed comparisons of inducing clinical response (defined by a reduction in the CDAI [Crohn’s Disease Activity Index] ≥70 [CDAI-70] or ≥100 [CDAI-100] points compared to baseline) between included biologic agents in tumor necrosis factor antagonist-naïve patients with moderate-to-severe Crohn’s disease, reporting odds ratios on the logarithmic scale (log OR) with 95% confidence intervals. Log OR greater or less than 0 are indicated in red/yellow or blue/green, respectively, in comparisons of inducing CDAI-100/CDAI-70, and the darker the color, the greater the odds ratios. Bold type represents statistically significant superiority/inferiority of the intervention over the comparator.

In the evaluation of inducing CDAI-70 in TNF antagonist-naïve CD patients, the results of funnel plot, model fit and convergence assessments do not require any special attention. On direct meta-analysis, Infliximab (OR 22.00 [95% CI: 5.17, 93.56]) and Adalimumab (OR 4.46 [95% CI: 2.08, 9.56]) were significantly superior to placebo, and the other biologic agents were not evaluated for CDAI-70 in the included RCTs. Similarly, for both Infliximab (OR 0.64 [95% CI: 0.40, 1.02]) and Adalimumab (OR 2.05 [95% CI: 0.83, 5.08]), there was no benefit of their monotherapies over combinations with azathioprine, and Infliximab did not differ significantly from CT-P13 (OR 1.28 [95% CI: 0.71, 2.30]) for inducing CDAI-70. On network meta-analysis, Infliximab in combination with azathioprine (SUCRA 82.68) and Infliximab alone (SUCRA 72.54) were ranked highest (Figure 3).

In the evaluation of inducing CDAI-100 in TNF antagonist-naïve CD patients, all but Infliximab, which CDAI-100 was not evaluated in included RCTs, were significantly superior to placebo on direct meta-analysis, and there was no benefit of Ustekinumab over Adalimumab (OR 1.09 [95% CI: 0.71, 1.67]). On network meta-analysis, Ustekinumab (SUCRA 84.50) and Adalimumab (SUCRA 82.08) were ranked highest (Figure 3).

#### 3.3.2 Maintenance therapy

Only maintenance clinical remission was evaluated for TNF antagonist-naïve/experienced patients due to the lack of clinical data on the maintenance CDAI-70 and CDAI-100 to form a network. Overall, 11 RCTs including 2,335 TNF antagonist-naïve patients with moderate-to-severe CD evaluated maintenance clinical remission. Therapies that were evaluated included Infliximab (n = 4 trials, included one biosimilar-comparator trial, one combination-therapy-comparator trial and on three-arm trial [the ACCENT-1 trial]) (Rutgeerts et al., 1999; Hanauer et al., 2002; Colombel et al., 2010; Ye et al., 2019), Adalimumab (n = 2 trials, included one combination-therapy-comparator trial) (Colombel et al., 2007; Matsumoto et al., 2016), Ustekinumab (n = 2 trials, included one active-comparator trial) (Feagan et al., 2016; Sands et al., 2022), Vedolizumab (n = 3 trials, included one trial evaluating Vedolizumab SC) (Sandborn et al., 2013; Watanabe et al., 2020; Vermeire et al., 2022).

**FIGURE 4:**
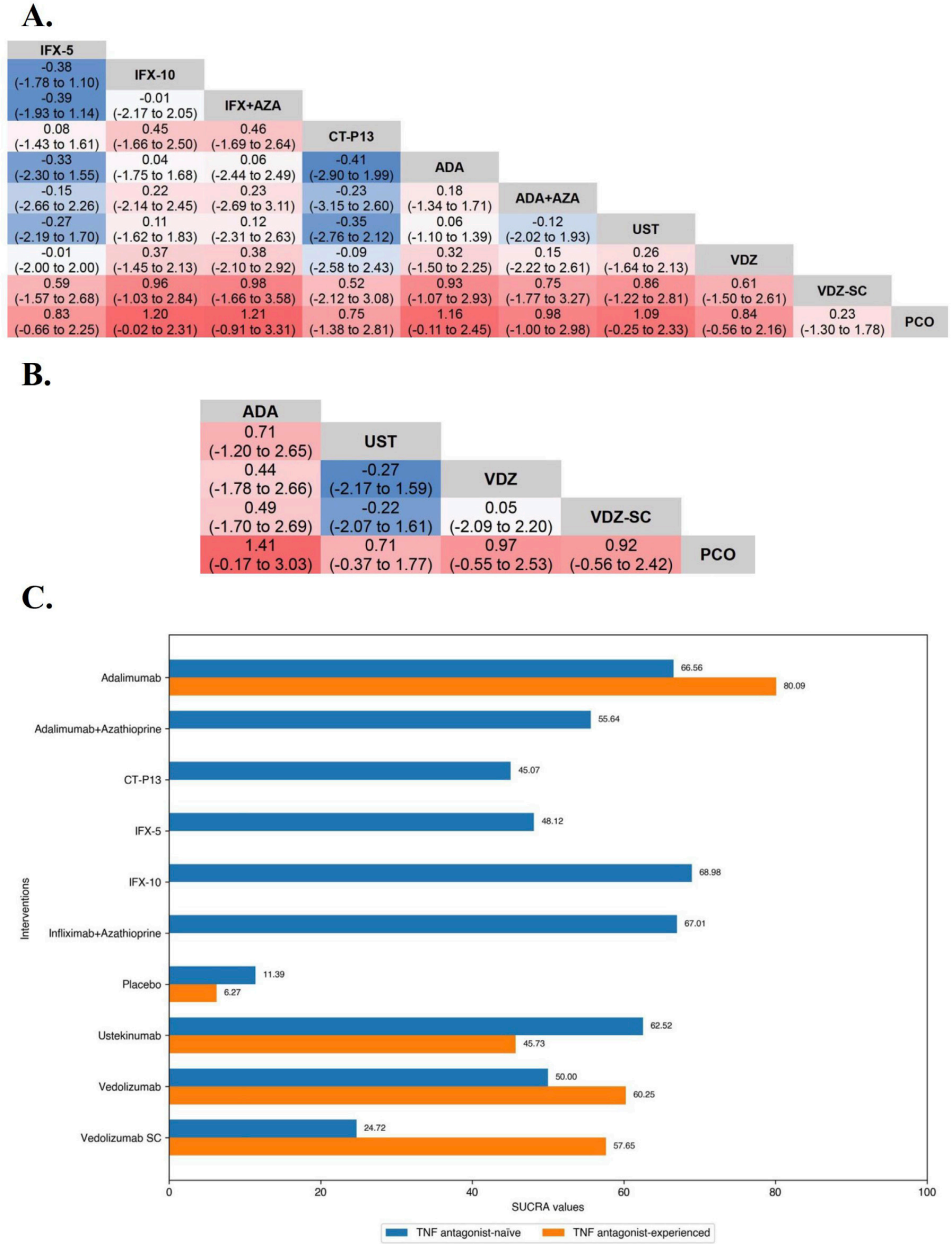
**(A)** SUCRA (the surface under cumulative ranking curve) values of each intervention in evaluation of maintaining clinical remission in tumor necrosis factor antagonist-naïve/experienced patients with moderate-to-severe Crohn’s disease. SUCRA values are expressed on a scale from 0 to 100. The closer the value is to 100, the more likely the intervention is to be among the most effective. Conversely, the closer the value is to 0, the less effective the treatment is likely to be. **(B)** League heat plots of mixed comparisons of maintaining clinical remission (defined by a CDAI [Crohn’s Disease Activity Index] < 150) between included biologic agents in tumor necrosis factor antagonist-naive patients with moderate-to-severe Crohn’s disease, reporting odds ratios on the logarithmic scale (log OR) with 95% confidence intervals. Log OR greater or less than 0 are indicated in red or blue, respectively, in comparisons of maintaining clinical remission, and the darker the color, the greater the odds ratios. Bold type represents statistically significant superiority/inferiority of the intervention over the comparator. **(C)** League heat plots of mixed comparisons of maintaining clinical remission (defined by a CDAI [Crohn’s Disease Activity Index] < 150) between included biologic agents in tumor necrosis factor antagonist-experienced patients with moderate-to-severe Crohn’s disease, reporting odds ratios on the logarithmic scale (log OR) with 95% confidence intervals. Log OR greater or less than 0 are indicated in red or blue, respectively, in comparisons of maintaining clinical remission, and the darker the color, the greater the odds ratios. Bold type represents statistically significant superiority/inferiority of the intervention over the comparator.

In the evaluation of maintaining clinical remission in TNF antagonist-naïve patients, the results of funnel plot, model fit and convergence assessments do not require any special attention. On direct meta-analysis, all but Ustekinumab (OR 1.96 [95% CI: 0.89, 4.34]) and Vedolizumab SC (OR 1.26 [95% CI: 0.67, 2.36]) were significantly superior to placebo, and comparisons between active agent regimens did not show significant differences. On network meta-analysis, IFX-10 (SUCRA 68.98), Infliximab in combination with azathioprine (SUCRA 67.01), and Adalimumab alone (SUCRA 66.56) were ranked highest (Figures 4A, B).

### 3.4 Efficacy in TNF antagonist-experienced patients with Crohn’s disease

#### 3.4.1 Induction therapy

Overall, 10 RCTs including 2,131 TNF antagonist-experienced patients with moderate-to-severe CD evaluated induction clinical response (CDAI-70 and CDAI-70). Therapies that were evaluated included Adalimumab (n = 2 trials) (Sandborn et al., 2007c; Watanabe et al., 2012), Certolizumab Pegol (n = 1 trial) (Sandborn et al., 2007a), Ustekinumab (n = 3 trials) (Sandborn et al., 2012; Feagan et al., 2016), Risankizumab (n = 1 trial) (Feagan et al., 2017), and Vedolizumab (n = 3 trials) (Sandborn et al., 2013; Sands et al., 2014; Watanabe et al., 2020).

**FIGURE 5:**
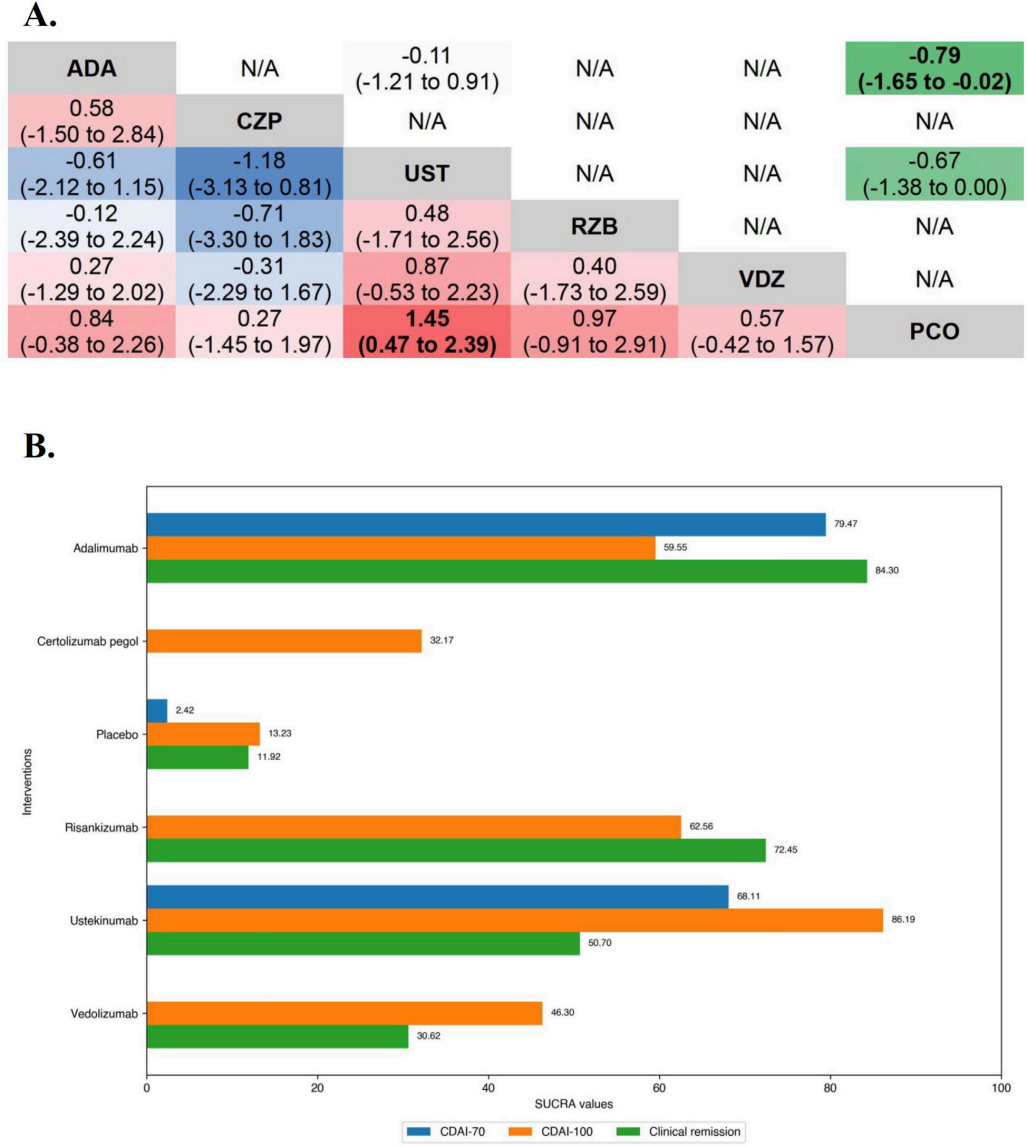
**(A)** SUCRA (the surface under cumulative ranking curve) values of each intervention in efficacy evaluations of induction therapy in tumor necrosis factor antagonist-experienced patients with moderate-to-severe Crohn’s disease. SUCRA values are expressed on a scale from 0 to 100. The closer the value is to 100, the more likely the intervention is to be among the most effective. Conversely, the closer the value is to 0, the less effective the treatment is likely to be. **(B)** League heat plots of mixed comparisons of inducing clinical response (defined by a reduction in the CDAI [Crohn’s Disease Activity Index] ≥70 [CDAI-70] or ≥100 [CDAI-100] points compared to baseline) between included biologic agents in tumor necrosis factor antagonist-experienced moderate-to-severe Crohn’s disease, reporting odds ratios on the logarithmic scale (log OR) with 95% confidence intervals. Log OR greater or less than 0 are indicated in red/yellow or blue/green, respectively, in comparisons of inducing CDAI-100/CDAI-70, and the darker the color, the greater the odds ratios. Bold type represents statistically significant superiority/inferiority of the intervention over the comparator.

In the evaluation of inducing CDAI-70 in TNF antagonist-experienced CD patients, the results of funnel plot, model fit and convergence assessments do not require any special attention. On direct meta-analysis, only two placebo-controlled trials each of Adalimumab (OR 2.09 [95% CI: 1.34, 3.27]) and Ustekinumab (OR 1.79 [95% CI: 1.23, 2.58]) were included, and both biologics had a significantly superiority over placebo. Direct and network comparisons for induction CDAI-70 in TNF antagonist-experienced CD patients were similar, and the efficacy of Adalimumab was higher than that of Ustekinumab (Figure 5).

In the evaluation of inducing CDAI-100 in TNF antagonist-experienced CD patients, all but Risankizumab (OR 2.55 [95% CI: 0.86, 7.60]) and Certolizumab Pegol (OR 1.30 [95% CI: 0.64, 2.62]) were significantly superior to placebo on direct meta-analysis. On network meta-analysis, Ustekinumab (SUCRA 86.19) and Risankizumab (SUCRA 62.56) were ranked highest (Figure 5).

#### 3.4.2 Maintenance therapy

Only maintenance clinical remission was evaluated for TNF antagonist-naïve/experienced patients. A total of 5 RCTs including 866 TNF antagonist-experienced patients with moderate-to-severe CD evaluated maintenance clinical remission after treatment with Adalimumab (n = 1 trial), Ustekinumab (n = 2 trials), Vedolizumab (n = 2 trials, included one trial evaluating Vedolizumab SC).

In the evaluation of maintaining clinical remission in TNF antagonist-experienced patients, all agents were significantly superior to placebo on direct meta-analysis. On network meta-analysis, Adalimumab (SUCRA 80.09) and Vedolizumab (SUCRA 60.25) were ranked highest (Figure 4C).

### 3.5 Safety in overall patients with Crohn’s disease

Due to the lack of safety subgroup analysis data on the medication history of TNF antagonist, only the safety of each biologic agents in overall CD patients was evaluated.

#### 3.5.1 Induction therapy

**FIGURE 6:**
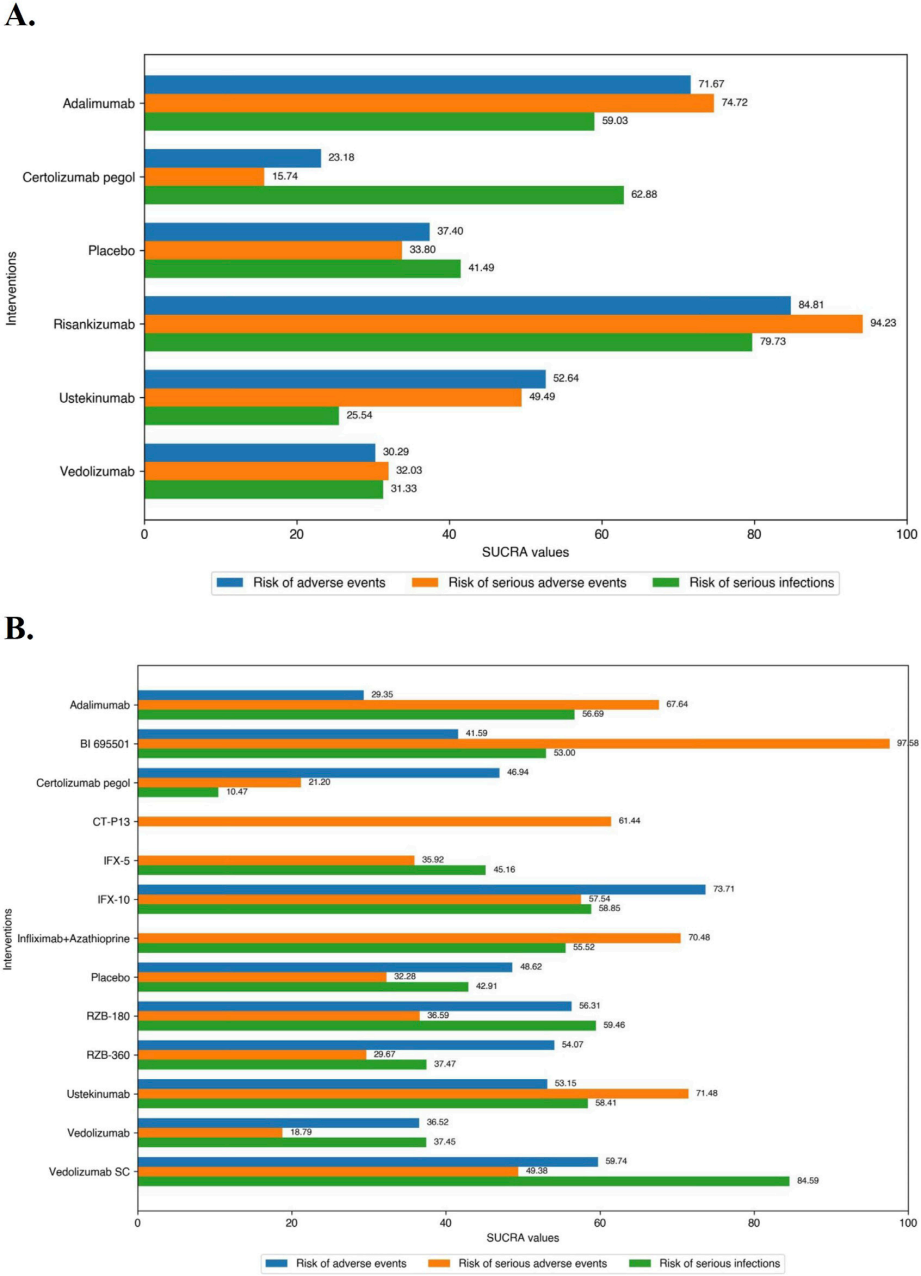
**(A)** SUCRA (the surface under cumulative ranking curve) values of each intervention in safety evaluations of induction therapy in overall patients with moderate-to-severe Crohn’s disease. SUCRA values are expressed on a scale from 0 to 100. The closer the value is to 100, the more likely the intervention is to be among the most safety. Conversely, the closer the value is to 0, the less effective the treatment is likely to be. **(B)** SUCRA (the surface under cumulative ranking curve) values of each intervention in safety evaluations of maintenance therapy in overall patients with moderate-to-severe Crohn’s disease. SUCRA values are expressed on a scale from 0 to 100. The closer the value is to 100, the more likely the intervention is to be among the most safety. Conversely, the closer the value is to 0, the less effective the treatment is likely to be.

In the safety evaluation of patient with moderate-to-severe CD in induction therapy, direct and network comparisons for risk of AEs, SAEs, or SIs in induction therapy were similar, with no statistical differences between any of the included interventions. On network meta-analysis, Risankizumab (SUCRA 84.81), Adalimumab (SUCRA 71.67), and Ustekinumab (SUCRA 52.64) had the lowest risk of AEs during induction therapy. Risankizumab (SUCRA 94.23), Adalimumab (SUCRA 74.72), and Ustekinumab (SUCRA 49.49) had the lowest risk of SAEs during induction therapy, and Risankizumab had a significant safety benefit over Ustekinumab, Vedolizumab, and Certolizumab Pegol. Risankizumab (SUCRA 79.73), Certolizumab Pegol (SUCRA 62.88), and Adalimumab (SUCRA 59.03) had the lowest risk of SIs during induction therapy (Figure 6A).

#### 3.5.2 Maintenance therapy

In the safety evaluation of patients with moderate-to-severe CD in maintenance therapy, there was no significantly difference between included interventions in evaluation of the risk of AEs on direct meta-analysis. In the SONIC trial (Infliximab vs. Infliximab combined with azathioprine), Infliximab alone (5 mg/kg maintenance regimen of Infliximab) had a significantly higher risk of SAEs than in combination with azathioprine (OR 1.77 [95% CI: 1.03, 3.05]), while the other comparisons between the agents were not significantly different. On network meta-analysis, IFX-10 (SUCRA 73.71), Vedolizumab SC (SUCRA 59.74), and RZB-180 (SUCRA 56.31) had the lowest risk of AEs during maintenance therapy. BI 695501 (SUCRA 97.58), Ustekinumab (SUCRA 71.48), Infliximab in combination with azathioprine (SUCRA 70.48) had the lowest risk of SAEs during maintenance therapy, and BI 695501 had a significant safety superiority over almost all of the other agents (except Ustekinumab and Infliximab in combination with azathioprine). Vedolizumab SC (SUCRA 84.59), RZB-180 (SUCRA 59.46), and IFX-10 (SUCRA 58.85) had the lowest risk of SIs during maintenance therapy (Figure 6B), and Vedolizumab SC had a significant lower risk of SIs compared to Certolizumab Pegol (log OR -2.22 [95% CrI: −4.42, −0.17]).

### 3.6 Sensitivity analysis and consistency analysis

We conducted several sensitivity analyses and the general consistency of the results confirms the robustness of our findings (all sensitivity analyses results are detailed in Supplementary File 2). In consistency vs. inconsistency plots, the contributions to the deviance are very similar and close to 1, indicating no evidence of inconsistency in our networks.

## 4 Discussion

In this updated systematic review and Bayesian network meta-analysis, we combined direct and indirect evidence from 31 RCTs to evaluate the efficacy of different therapies for inducing and maintaining clinical remission or clinical response in moderate-to-severe CD populations. Several important findings from this network meta-analysis may inform clinical decisions. Firstly, two TNF antagonists, Infliximab and Adalimumab, continue to have a high status in induction and maintenance therapy. In addition, Infliximab in combination with azathioprine has a significant superiority in induction therapy in TNF antagonist-naïve patients, and appropriately increasing the dose of infliximab (10 mg/kg) may improve the efficacy of maintenance therapy. Secondly, Ustekinumab and Risankizumab may be the therapeutic agents of choice for TNF antagonist-experienced patients. High-dose Risankizumab (360 mg) also failed to show more efficacy benefits than low-dose Risankizumab (180 mg) in maintenance therapy. Thirdly, Vedolizumab does not offer superior therapeutic safety commensurate with its mechanism, and the subcutaneous injection formulation of Vedolizumab needs to demonstrate advantages beyond efficacy to establish its superiority over the widely approved intravenous infusion formulation. Finally, Risankizumab may be have worth-exploring lower safety risks in induction therapy. Altogether, these findings will inform the optimal sequencing of biologic therapy for patients with different histories of biologic administration.

Despite the emergence of new biologic agents, TNF antagonists remain most extensively experienced in the therapy of CD. In addition to the high-ranking performance in this network meta-analysis, TNF antagonists play a defined role in patients with fistulizing or structuring CD (Present et al., 1999; Sands et al., 2004; Bouhnik et al., 2018; Lee et al., 2018; Rodríguez-Lago et al., 2020), and seems to be a valuable option in the treatment of extraintestinal manifestations given the good response rates in a retrospective analysis of the Swiss IBD Cohort Study (Vavricka et al., 2017), though we acknowledge estimates supporting a large effect size for Infliximab have a low confidence due to the small number of induction RCTs available for inclusion and the small sample size of RCTs that were included. There was no statistically significant difference between 5 and 10 mg/kg intravenous infusion of Infliximab, which are both approved for the treatment of CD. However, in the three-arm ACCENT-1 trial, high-dose Infliximab (IFX-10) was associated with a superiority of SUCRA compared to the low-dose (IFX-5) in maintenance therapy (Hanauer et al., 2002). Combined with the fact that there is currently no evidence of higher safety risks associated with higher doses of Infliximab, this seems to suggest that increasing the dose of Infliximab has the potential to improve efficacy in maintenance therapy. While also as a TNF antagonist, Certolizumab Pegol did not show similar efficacy in the treatment of CD in this NMA. Nevertheless, in a retrospective analysis compared the real-world efficacy and safety of different TNF antagonists in biologic-naïve patients with CD, Certolizumab Pegol was comparable in efficacy to Adalimumab (Singh et al., 2016), and a head-to-head active-comparator RCT is required to judge the precise efficacy of Certolizumab Pegol.

Though the irreplaceable role of TNF antagonists in the treatment of CD, unfortunately one-third of patients with CD do not respond to initial treatment with TNF antagonists, and another one-third have only a transient response requiring a dose increase or switching to another therapy (Hanauer et al., 2002; Hanauer et al., 2006; Colombel et al., 2007; Sandborn et al., 2007a; Schreiber et al., 2007). Preventative combination treatment comes into view and may be the management of non-response. In our analysis, Infliximab in combination with azathioprine demonstrated clear efficacy benefit whereas Adalimumab in combination with azathioprine did not. In the SONIC trial, TNF antagonist-naïve patients with moderate-to-severe CD who received Infliximab in combination with azathioprine had a significant benefit on the rates of corticosteroid-free clinical remission compared to those receiving Infliximab alone or azathioprine alone, with comparable toxicity among groups (Colombel et al., 2010). In contrast, the open-label DIAMOND trial in which the efficacy of Adalimumab in combination with azathioprine did not differ significantly from that of Adalimumab monotherapy in patients naïve to both TNF antagonists and immunosuppression (Matsumoto et al., 2016). In both trials, patients receiving combination therapy who experienced worsening of CD were significantly fewer than those receiving TNF antagonist monotherapy (*p* = 0.045 in SONIC, p< 0.001 in DIAMOND, using Fisher’s Exact Test). This suggests that combination therapy may help prevent LOR to TNF antagonists, although LOR is not the only cause of worsening of CD (Ben-Horin and Chowers, 2011).

However, the populations of DIAMOND and SONIC were naïve to immunosuppressants and biologics, meaning that the effect of combination therapy in preventing LOR in immunosuppressant- and biologic-experienced patients remains unknown. Therefore, some researchers evaluated the benefit to risk ratio of concomitant immunosuppressives with scheduled TNF antagonists by identifying the influence of withdrawal of immunosuppressives. In an open-label interventional study reported by Van Assche G et al., there was no significant difference to continuation of immunosuppression in combination for < or = 6 months compared to scheduled Infliximab monotherapy in the proportions of patients with secondary non-response (Van Assche et al., 2008). Continuation of Adalimumab combined with oral thiopurines beyond 6 months offers no clear benefit over scheduled Adalimumab monotherapy in the DIAMOND-2 trial (Hisamatsu et al., 2019). Similarly, there is no evidence of superior efficacy of concomitant immunomodulating medication in therapy with non-TNF antagonistic biologics but come with an increased risk of SIs (Hu et al., 2021; Yzet et al., 2021). Overall, the role of concomitant immunosuppressives in treatment of CD still needs to be explored further.

In recent years, patents of Infliximab and Adalimumab have expired in many countries, and the huge biologics market has made biosimilar manufacturers eager. Both biosimilars, CT-P13 and BI 695501, were comparable in efficacy and safety to their respective originators in direct head-to-head comparisons (Ye et al., 2019; Hanauer et al., 2021). Subcutaneous injections formulation of CT-P13 (CT-P13 SC) is a product worthy of attention. The pharmacokinetics, as manifested by trough concentrations of the agents, increased after CT-P13 switching from intravenous to subcutaneous administration in a phase 1 study (Schreiber et al., 2021). Smith PJ et al. observed high treatment persistence rates and low rates of immunogenicity in IBD patients with maintenance therapy switching from Infliximab to CT-P13 SC in a multicenter retrospective cohort study (Smith et al., 2022). The results of the LIBERTY-CD trial demonstrate that maintenance therapy with CT-P13 SC provides both a robust clinical benefit and the convenience of subcutaneous administration to moderate-to-severe CD patients (Colombel et al., 2023).

In this network meta-analysis, although Adalimumab had the largest SUCRA value in the evaluations of inducing clinical remission and CDAI-70 in TNF antagonist-experienced CD patients, we recommend Ustekinumab and Risankizumab as the therapeutic agents of choice for induction therapy in TNF antagonist-experienced patients, because of the intransitivity caused by the inclusion criteria, which selectively included the patients with intolerance or secondary non-response to Infliximab, in the GAIN trial (Sandborn et al., 2007c). In contrast, the SEAVUE trial, a head-to-head trial to standard doses of different biologic agents for CD treatment, strengthens the reliability of our findings (Sands et al., 2022). The results of inconsistency analysis showed that there was no inconsistency between direct and indirect evidence of the comparison of Adalimumab and Ustekinumab (Dias et al., 2010; Dias et al., 2014b).

Notably, the high clinical benefit in SEAVUE compared to other RCTs may be attributed to several factors: the short disease course, the biologic-naïve baseline characteristics of enrolled patients (received early biologic treatment [EBT]), and non-placebo-controlled design (patients were aware they received active study therapy) (Sands et al., 2022). But in fact, the superiority of EBT over conventional therapy (CT) for CD is disputed. The result of an upgrade of a previous CADTH (Canadian Agency for Drugs and Technologies in Health) rapid response reported by Thompson W et al. suggested that the clinical effectiveness of EBT compared to CT for CD in adults is still unclear but did point to possible benefits which require further study (Thompson and Argaez, 2019). In contrast, Ungaro RC et al. demonstrated that EBT was associated with improved clinical outcomes compared to late or CT in both prospective clinical trials and real-world studies in a systematic review (Ungaro et al., 2020). In addition, early TNF antagonist therapy is a more cost-effective management of CD compared with conventional therapy (Marchetti et al., 2013; Beilman et al., 2019). However, it is worth noting that the results of these studies may not be generalizable to other biologics as they mainly involved EBT of TNF antagonists.

Between Ustekinumab and Risankizumab, in the SEQUENCE trial (Nct, 2020), a phase 3 head-to-head study compared these two interleukin antagonists for the treatment of patients with moderate-to-severe CD who have failed at least one TNF antagonists. Risankizumab had demonstrated non-inferiority for clinical remission at week 24 and superiority of endoscopic remission at week 48 over Ustekinumab, and showed superiority versus Ustekinumab for all ranked secondary endpoints, including achievement of clinical remission at week 48, endoscopic response at week 48 and 24, steroid-free endoscopic remission at week 48, and steroid-free clinical remission at week 48, with no new safety risks identified. These results are posted on AbbVie News Center. Ustekinumab and Risankizumab actually differ in mechanisms of action, although they are both interleukin antagonists. Unlike Ustekinumab, which inhibits the biological activity of interleukin-12 (IL-12) and interleukin-23 (IL-23) by blocking the common p40 subunit of these two cytokines (Benson et al., 2011), Risankizumab downregulates IL-23-mediated inflammation by binding with high affinity to the p19 subunit of IL-23 (Singh et al., 2015). Therefore, it does not affect the IL-12-dependent T-cell pathways that are implicated in infection and cancer risk (Brunda et al., 1993; Stobie et al., 2000), potentially resulting in a lower safety risk (Wong and Cross, 2019).

Sadly, detailed data from the SEQUENCE trial is not available, which may have resulted in the potential efficacy superiority of Risankizumab over Ustekinumab not being reflected in our analysis. Nevertheless, we obtained and analyzed the key information from several included placebo-controlled trials studying Risankizumab and come up with the following findings (Feagan et al., 2017; D'Haens et al., 2022; Ferrante et al., 2022): First, Risankizumab has a ranking superiority over other biologics on induction of clinical remission in TNF antagonist-experienced CD patients, which may be paralleled by the result of the newly completed SEQUENCE trial; Second, the efficacy of high dose of Risankizumab (360 mg) was no better than that of low dose (180 mg) in the maintenance therapy; Third, Risankizumab has a relatively low safety risk in induction therapy, which may be associated with the specific mechanism of action.

Compared to the other biologics, Vedolizumab provides a more acceptable safety profile in its mechanism. By specifically targeting the α4β7/MAdCAM-1 pathway, Vedolizumab reduces localized immune responses in the gut, thereby providing therapeutic effects against intestinal inflammation. This mechanism allows for treatment efficacy in the gut without interfering with T-cell trafficking to the central nervous system or compromising systemic immune function (Wyant et al., 2016). However, Vedolizumab did not stand out either on efficacy or safety in this analysis. In other words, there is no superiority of Vedolizumab over interleukin antagonists for induction therapy in TNF antagonist-experienced patients. Although we acknowledge that a head-to-head comparison would be required to firmly confirm this conclusion, the results of several recent real-world studies have also demonstrated that Ustekinumab has a better efficacy in CD patients who have failed treatment with TNF antagonists compared to Vedolizumab (Alric et al., 2020; Biemans et al., 2020; Townsend et al., 2020; Manlay et al., 2021; Garcia et al., 2024). For example, García MJ et al. reported a prospective study including 207 Vedolizumab-treated patients and 628 Ustekinumab-treated patients who had TNF antagonist failure or intolerance from ENEIDA registry (Nationwide study on genetic and environmental determinants of inflammatory bowel disease): Vedolizumab was associated with a higher risk of treatment discontinuation compared with Ustekinumab, adjusted by corticosteroids at baseline, moderate-severe activity in Harvey-Bradshaw Index (HBI), and high levels of C-reactive protein at baseline (Garcia et al., 2024). The potential differences on efficacy between Ustekinumab and Vedolizumab in patients with prior failure of TNF antagonists may be attributed to different mechanisms for down-regulating IL-23-driven compensatory inflammation after blockade of TNF pathway (Schmitt et al., 2019). Vedolizumab SC may have superiority on safety and patient medication compliance because of its method of administration and was approved for maintenance therapy of CD in several countries, although it did not have benefit on efficacy in our network meta-analysis. Nevertheless, in the future, the management of switching the intravenous infusions formulation to the subcutaneous injections formulation in maintenance therapy phase deserves attentions.

In this meta-analysis, we also collected and analyzed the safety outcomes of the included RCTs and the results showed the superiority of Risankizumab and BI 695501 in induction and maintenance therapy, respectively. However, these findings should be interpreted cautiously for several reasons. First, these safety findings are at odds with the results of some recent real-world studies. For example, the results of multicenter California-IBD cohort study reported by Singh S et al. suggested that Ustekinumab was associated with a lower risk of SIs in CD patients compared with TNF antagonists and Vedolizumab, but the risk of SIs in maintenance therapy with Ustekinumab, Vedolizumab and three original TNF antagonists did not significantly different in our analysis (Singh et al., 2023). Vedolizumab and TNF antagonists showed comparable risks of SIs in other studies besides this one by Kirchgesner et al. (2022), Singh et al. (2022). In addition, in the study by Bressler B et al., Vedolizumab-treated patients were less likely to experience SAEs compared with TNF antagonists after adjustment using inverse probability weighting (Bressler et al., 2021). To clarify this ambiguity, head-to-head, direct comparisons and more real-world studies were needed. Second, the treatment of CD is a lifelong process, clearly, even the RCT with the longest study duration of 60 weeks included in our analysis, would not be able to cover the entire course of a CD patient, and therefore interpretations of these safety findings are restricted. In several long-term open-label studies, multiple agents, included Ustekinumab and Vedolizumab, maintained good efficacy in maintenance of remission and no new safety signal was observed, although LOR continues to be a problem in later treatment (Vermeire et al., 2017; Loftus et al., 2020; Sandborn et al., 2022). Third, safety information of medical products from RCTs designed for registrational purposes is often under-detected and under-utilized. Indeed, the information provided in most published RCT reports is generally insufficient and inconsistent to comprehensively summarize the safety of medical products, even in RCTs reports published in high-impact journals. No significant differences in AEs rates between the interventions and placebo reported in some studies do not imply that the studying drugs are truly safe (Phillips et al., 2019). Therefore, the evaluations of safety should integrate the reported RCTs studies and real-world evidences. Consistency of reporting important safety results across trials should be improved to facilitate comprehensive comparisons, which can be referred to the work that the COMET (Core Outcome Measures in Effectiveness Trials) group contributes to (Gargon, 2016), and reporting safety data according to the CONSORT (Consolidated Standards of Reporting Trials) harm checklist should be promoted in RCTs reports (Cuschieri, 2019). Nevertheless, the dominances of Risankizumab, BI 695501 and Vedolizumab SC in risks of SAEs in induction therapy, in risks of SAEs, and of SIs in maintenance therapy, respectively, in our analysis deserve further investigation.

Our analysis has several distinct strengths over other previous meta-analyses comparing the safety and efficacy of biologics for moderate-to-severe CD. First, we utilized Bayesian methods instead of a classical Frequentist method, and employed MCMC which can leverage prior information and circumvent the limitations of hypothesis testing, for parameter estimation and sampling from the posterior distribution, and provided complete assessments of model fit and convergence of MCMC model. Second, we evaluated efficacy separately with different definitions of clinical response (CDAI-70 and CDAI-100) to reduce heterogeneity. Third, we review some of the published key results of the SEQUENCE trial, and included a direct head-to-head comparison (SEAVUE) between Ustekinumab and Adalimumab, which reduces uncertainty of the results, validates the results of indirect comparison and strengthens the comparative networks. Fourthly, we also included comparisons between different dose groups in two three-arm trials (ACCENT-1 and FORTIFY) in evaluations of maintenance therapy, which further enriches our comparative networks, and an agent, Vedolizumab SC, with potential advantages on safety and patient compliance.

Of course, we also acknowledge that there are several limitations and shortcomings in our analysis. First, we evaluated the safety and efficacy of biologics in stratified analyses according to their history of TNF antagonist treatment in CD patients, as well as in the overall CD patients. Although it might seem unnecessary, we believe this approach could be beneficial for managing patients with an ambiguous history of TNF antagonist treatment. Second, we used clinical response and clinical remission as primary efficacy outcomes for induction and maintenance therapy, respectively. Actually, in the CORE-IBD consensus initiative (first international consensus-based Core Outcome Set [COS] for use in IBD RCTs) (Collaborators et al., 2022), there was controversy among panelists about historically defining clinical remission as a CDAI score of <150. Furthermore, endoscopic outcomes, defined by the more recognized SES-CD (Simple Endoscopic Score for Crohn’s Disease) (Daperno et al., 2004), and corticosteroid-free remission, which is more clinically significant and improves the sensitivity of detecting efficacy, are included in the configuration of outcomes in the CORE-IBD consensus. Although these outcomes are highly regarded, we believe that optimizing the setting of outcomes remains challenging in the face of heterogeneity in outcome definitions across RCTs distributed over several decades, as well as the lack of relevant data of outcomes and few stratified analyses according to history of TNF antagonist treatment in many RCTs. Third, we acknowledge the heterogeneity in RCTs design and baseline characteristics of studying patients between the included studies. The baseline characteristics of enrolled patients in recently completed RCTs, influenced by decades of development in biologics for treating CD, differ from those in earlier studies: the proportion of patients enrolled with a history of surgery for CD in recent RCTs seems to be lower. For example, in the VISIBLE-2 trial (2022), 26.9% (110/409) of enrolled patients with a history of surgery for CD, compared to 41.8% (466/1115) in the GEMINI-2 trial (2013), indicating a significant difference. Additionally, patients enrolled in recent RCTs had a longer duration of CD and an increased proportion with a history of biologic treatment. To control the overall heterogeneity of the pooled results, we conservatively used global random effects model, included both re-randomization and treat-though trials in our analysis, and finally assessed the impact of model selection on the robustness of the results by sensitivity analysis.

In conclusion, this updated systematic review and network meta-analysis, utilizing a Bayesian consistency random-effects model, highlights several key findings for the treatment of CD: Infliximab combined with azathioprine is particularly superior for induction therapy in TNF antagonist-naïve patients. Adalimumab and the 10 mg/kg maintenance regimen of Infliximab have maintenance efficacy benefits. Ustekinumab and Risankizumab may be preferred biologic agents for induction therapy in TNF antagonist-experienced patients. Risankizumab maybe have the advantage worth exploring of lower safety risks. In contrast, there is insufficient evidence from RCTs to support the safety advantage of Vedolizumab. Our analysis provides valuable insights for the risk-benefit assessments of biologics in CD treatment by patients and healthcare professionals.

## Data Availability

The original contributions presented in the study are included in the article/Supplementary Material, further inquiries can be directed to the corresponding authors.

## Abbreviations

ADA, Adalimumab; ADA + AZA, Adalimumab in combination with azathioprine; AEs, adverse events; CDAI, Crohn’s disease activity index; CDAI-70/100, clinical response defined by a reduction in the Crohn’s disease activity index ≥70/100 points compared to baseline; CD, Crohn’s disease; CI, confidence interval; CrI, credible interval; CZP, Certolizumab pegol; IFX, Infliximab; IFX-5/10, the 5/10 mg/kg maintenance regimen of Infliximab; IFX + AZA, Infliximab in combination with azathioprine; MCMC, Markov chain Monte Carlo; PCO, placebo; RCTs, randomized controlled trials; RZB, Risankizumab; RZB-180/360, the 180/360 mg maintenance regimen of Risankizumab; SAEs, serious adverse events; SF, Supplementary File; SIs, serious infections; SUCRA, the surface under cumulative ranking curve; TNF, tumor necrosis factor; UST, Ustekinumab; VDZ, Vedolizumab; VDZ-SC, subcutaneous injections formulation of Vedolizumab.
