# Supplementary material for: Systematic review and bayesian network meta-analysis: comparative efficacy and safety of six commonly used biologic therapies for moderate-to-severe Crohn’s disease
Source: Front Pharmacol. 2025 Jan 9;15:1475222. doi: 10.3389/fphar.2024.1475222 (PMC11794990; doi:10.3389/fphar.2024.1475222)
Supplement: Supplementary file 1 [file DataSheet2.docx]

**SUPPLEMENTARY FILE 2: SENSITIVITY ANALYSES**

Sensitivity analysis 1: changed the original DerSimonian-Laird random-effects model to a Mantel-Haenszel fixed-effect model; Sensitivity analysis 2: changed the standard deviation of original independent normal priors on the baseline treatment effects and relative treatment effects in the model to 10u; Sensitivity analysis 3: excluded the trials in which the data points in funnel plot fell outside the 90% confidence interval; Sensitivity analysis 4: excluded the treat-through trials (the SONIC, DIAMOND, SEAVUE, and PRECISE-1); Sensitivity analysis 5: excluded the trials with a high/some-concerns risk of bias (DIAMOND and Rutgeerts P et al., 1999); Sensitivity analysis 6: excluded the trials contributing to the model’s poor fit; Sensitivity analysis 7: changed the original effect size (Odds Ratio) to Relative Risk.

| Outcome | Treatment | Comparator | Main estimate | Sensitivity analysis 1 | Sensitivity analysis 2 | Sensitivity analysis 3 | Sensitivity analysis 4 | Sensitivity analysis 5 | Sensitivity analysis 6 | Sensitivity analysis 7  (Relative Risk) |
| --- | --- | --- | --- | --- | --- | --- | --- | --- | --- | --- |
| **Moderate-to-severe CD patients** | | | | | | | | | | |
| Inducing clinical remission | Infliximab | Infliximab+azathioprine | -0.12  (-0.84 to 0.60) | -0.12  (-0.55 to 0.31) | -0.12  (-0.83 to 0.60) | N/A | N/A | -0.12  (-0.84 to 0.60) | -0.11  (-0.76 to 0.52) | -0.05  (-0.56 to 0.46) |
| Inducing clinical remission | Infliximab | CT-P13 | 0.11  (-0.68 to 0.89) | 0.11  (-0.43 to 0.65) | 0.11  (-0.67 to 0.89) | N/A | N/A | 0.11  (-0.68 to 0.89) | 0.11  (-0.61 to 0.82) | 0.05  (-0.50 to 0.59) |
| Inducing clinical remission | Infliximab | Adalimumab | 2.14  (0.09 to 5.58) | 2.20  (0.28 to 5.51) | 2.15  (0.11 to 5.39) | N/A | N/A | 2.16  (0.10 to 5.39) | 2.13  (0.04 to 5.30) | 1.04  (-0.33 to 3.04) |
| Inducing clinical remission | Infliximab | Adalimumab+azathioprine | 2.89  (0.64 to 6.42) | 2.95  (0.89 to 6.31) | 2.91  (0.65 to 6.25) | N/A | N/A | N/A | 2.88  (0.61 to 6.12) | 1.26  (-0.20 to 3.32) |
| Inducing clinical remission | Infliximab | BI 695501 | 1.79  (-0.48 to 5.35) | 1.85  (-0.22 to 5.21) | 1.81  (-0.47 to 5.17) | N/A | N/A | 1.82  (-0.47 to 5.18) | 1.79  (-0.50 to 5.05) | 0.93  (-0.55 to 2.98) |
| Inducing clinical remission | Infliximab | Certolizumab Pegol | 3.12  (1.06 to 6.58) | 3.12  (1.20 to 6.44) | 3.13  (1.08 to 6.38) | N/A | N/A | 3.14  (1.08 to 6.39) | 3.15  (1.08 to 6.32) | 1.75  (0.37 to 3.75) |
| Inducing clinical remission | Infliximab | Ustekinumab | 2.47  (0.45 to 5.91) | 2.44  (0.53 to 5.75) | 2.48  (0.47 to 5.74) | N/A | N/A | 2.49  (0.46 to 5.72) | 2.36  (0.28 to 5.53) | 1.23  (-0.12 to 3.23) |
| Inducing clinical remission | Infliximab | Risankizumab | 2.64  (0.56 to 6.10) | 2.65  (0.72 to 5.97) | 2.66  (0.60 to 5.92) | N/A | N/A | 2.66  (0.57 to 5.92) | 2.67  (0.59 to 5.86) | 1.37  (-0.03 to 3.38) |
| Inducing clinical remission | Infliximab | Vedolizumab | 2.99  (0.93 to 6.42) | 2.95  (1.02 to 6.27) | 3.00  (0.96 to 6.24) | N/A | N/A | 3.00  (0.95 to 6.25) | 3.02  (0.93 to 6.19) | 1.65  (0.28 to 3.68) |
| Inducing clinical remission | Infliximab | placebo | 3.40  (1.41 to 6.83) | 3.40  (1.51 to 6.71) | 3.41  (1.44 to 6.63) | N/A | N/A | 3.42  (1.43 to 6.63) | 3.43  (1.40 to 6.59) | 1.94  (0.63 to 3.93) |
| Inducing clinical remission | Infliximab+azathioprine | CT-P13 | 0.23  (-0.84 to 1.29) | 0.23  (-0.46 to 0.91) | 0.23  (-0.83 to 1.29) | N/A | N/A | 0.23  (-0.84 to 1.29) | 0.22  (-0.73 to 1.18) | 0.10  (-0.65 to 0.85) |
| Inducing clinical remission | Infliximab+azathioprine | Adalimumab | 2.27  (0.07 to 5.77) | 2.32  (0.35 to 5.65) | 2.28  (0.10 to 5.57) | N/A | N/A | 2.29  (0.09 to 5.59) | 2.25  (0.06 to 5.47) | 1.10  (-0.37 to 3.14) |
| Inducing clinical remission | Infliximab+azathioprine | Adalimumab+azathioprine | 3.02  (0.64 to 6.61) | 3.08  (0.97 to 6.45) | 3.04  (0.65 to 6.44) | N/A | N/A | N/A | 3.00  (0.63 to 6.31) | 1.32  (-0.23 to 3.43) |
| Inducing clinical remission | Infliximab+azathioprine | BI 695501 | 1.92  (-0.48 to 5.51) | 1.97  (-0.14 to 5.35) | 1.94  (-0.46 to 5.35) | N/A | N/A | 1.95  (-0.46 to 5.37) | 1.90  (-0.46 to 5.23) | 0.99  (-0.58 to 3.09) |
| Inducing clinical remission | Infliximab+azathioprine | Certolizumab Pegol | 3.25  (1.05 to 6.75) | 3.24  (1.27 to 6.57) | 3.26  (1.08 to 6.57) | N/A | N/A | 3.27  (1.07 to 6.58) | 3.27  (1.09 to 6.49) | 1.80  (0.34 to 3.86) |
| Inducing clinical remission | Infliximab+azathioprine | Ustekinumab | 2.60  (0.44 to 6.10) | 2.56  (0.60 to 5.89) | 2.62  (0.47 to 5.93) | N/A | N/A | 2.62  (0.45 to 5.93) | 2.48  (0.29 to 5.70) | 1.28  (-0.15 to 3.34) |
| Inducing clinical remission | Infliximab+azathioprine | Risankizumab | 2.77  (0.57 to 6.28) | 2.78  (0.79 to 6.11) | 2.79  (0.59 to 6.10) | N/A | N/A | 2.79  (0.58 to 6.11) | 2.79  (0.61 to 6.03) | 1.43  (-0.06 to 3.50) |
| Inducing clinical remission | Infliximab+azathioprine | Vedolizumab | 3.11  (0.92 to 6.61) | 3.08  (1.10 to 6.41) | 3.13  (0.97 to 6.43) | N/A | N/A | 3.13  (0.95 to 6.46) | 3.14  (0.95 to 6.37) | 1.71  (0.25 to 3.79) |
| Inducing clinical remission | Infliximab+azathioprine | placebo | 3.53  (1.40 to 7.02) | 3.52  (1.58 to 6.84) | 3.54  (1.43 to 6.82) | N/A | N/A | 3.55  (1.42 to 6.84) | 3.55  (1.43 to 6.76) | 2.00  (0.60 to 4.03) |
| Inducing clinical remission | CT-P13 | Adalimumab | 2.05  (-0.17 to 5.56) | 2.10  (0.09 to 5.44) | 2.05  (-0.15 to 5.37) | N/A | N/A | 2.06  (-0.16 to 5.37) | 2.03  (-0.21 to 5.28) | 1.00  (-0.49 to 3.06) |
| Inducing clinical remission | CT-P13 | Adalimumab+azathioprine | 2.80  (0.39 to 6.40) | 2.85  (0.71 to 6.23) | 2.81  (0.40 to 6.22) | N/A | N/A | N/A | 2.79  (0.37 to 6.10) | 1.22  (-0.35 to 3.34) |
| Inducing clinical remission | CT-P13 | BI 695501 | 1.69  (-0.73 to 5.30) | 1.75  (-0.40 to 5.13) | 1.71  (-0.70 to 5.13) | N/A | N/A | 1.72  (-0.70 to 5.14) | 1.69  (-0.73 to 5.02) | 0.89  (-0.70 to 3.00) |
| Inducing clinical remission | CT-P13 | Certolizumab Pegol | 3.02  (0.81 to 6.56) | 3.02  (1.02 to 6.36) | 3.03  (0.82 to 6.36) | N/A | N/A | 3.04  (0.82 to 6.37) | 3.06  (0.82 to 6.30) | 1.71  (0.22 to 3.78) |
| Inducing clinical remission | CT-P13 | Ustekinumab | 2.38  (0.19 to 5.90) | 2.34  (0.34 to 5.68) | 2.39  (0.21 to 5.72) | N/A | N/A | 2.39  (0.19 to 5.71) | 2.27  (0.04 to 5.51) | 1.19  (-0.28 to 3.26) |
| Inducing clinical remission | CT-P13 | Risankizumab | 2.55  (0.32 to 6.08) | 2.56  (0.54 to 5.90) | 2.56  (0.33 to 5.89) | N/A | N/A | 2.56  (0.32 to 5.91) | 2.58  (0.34 to 5.84) | 1.33  (-0.18 to 3.41) |
| Inducing clinical remission | CT-P13 | Vedolizumab | 2.89  (0.68 to 6.41) | 2.85  (0.84 to 6.19) | 2.90  (0.71 to 6.22) | N/A | N/A | 2.90  (0.71 to 6.23) | 2.93  (0.69 to 6.16) | 1.61  (0.13 to 3.71) |
| Inducing clinical remission | CT-P13 | placebo | 3.31  (1.15 to 6.81) | 3.30  (1.32 to 6.63) | 3.31  (1.17 to 6.61) | N/A | N/A | 3.31  (1.16 to 6.62) | 3.34  (1.15 to 6.57) | 1.91  (0.47 to 3.95) |
| Inducing clinical remission | Adalimumab | Adalimumab+azathioprine | 0.73  (-0.13 to 1.63) | 0.74  (0.08 to 1.42) | 0.74  (-0.13 to 1.62) | 0.74  (-0.04 to 1.53) | N/A | N/A | 0.74  (-0.08 to 1.56) | 0.22  (-0.29 to 0.72) |
| Inducing clinical remission | Adalimumab | BI 695501 | -0.37  (-1.27 to 0.54) | -0.36  (-1.07 to 0.34) | -0.36  (-1.27 to 0.55) | N/A | N/A | -0.36  (-1.27 to 0.55) | -0.36  (-1.20 to 0.48) | -0.11  (-0.64 to 0.41) |
| Inducing clinical remission | Adalimumab | Certolizumab Pegol | 0.97  (0.35 to 1.68) | 0.92  (0.49 to 1.35) | 0.97  (0.34 to 1.68) | 0.76  (0.18 to 1.39) | N/A | 0.97  (0.34 to 1.68) | 1.02  (0.45 to 1.66) | 0.70  (0.22 to 1.26) |
| Inducing clinical remission | Adalimumab | Ustekinumab | 0.32  (-0.12 to 0.90) | 0.24  (-0.08 to 0.56) | 0.32  (-0.12 to 0.90) | 0.17  (-0.24 to 0.66) | N/A | 0.32  (-0.12 to 0.90) | 0.22  (-0.20 to 0.72) | 0.17  (-0.13 to 0.64) |
| Inducing clinical remission | Adalimumab | Risankizumab | 0.50  (-0.18 to 1.21) | 0.46  (-0.05 to 0.95) | 0.50  (-0.17 to 1.21) | 0.29  (-0.34 to 0.94) | N/A | 0.50  (-0.17 to 1.21) | 0.55  (-0.07 to 1.20) | 0.33  (-0.20 to 0.90) |
| Inducing clinical remission | Adalimumab | Vedolizumab | 0.84  (0.23 to 1.55) | 0.75  (0.27 to 1.24) | 0.84  (0.23 to 1.54) | 0.61  (0.02 to 1.26) | N/A | 0.84  (0.23 to 1.54) | 0.88  (0.30 to 1.53) | 0.61  (0.12 to 1.18) |
| Inducing clinical remission | Adalimumab | placebo | 1.26  (0.85 to 1.74) | 1.20  (0.88 to 1.53) | 1.26  (0.85 to 1.74) | 1.04  (0.63 to 1.51) | N/A | 1.26  (0.85 to 1.74) | 1.30  (0.92 to 1.75) | 0.90  (0.59 to 1.28) |
| Inducing clinical remission | Adalimumab+azathioprine | BI 695501 | -1.10  (-2.37 to 0.15) | -1.10  (-2.08 to -0.14) | -1.10  (-2.37 to 0.16) | N/A | N/A | N/A | -1.10  (-2.28 to 0.07) | -0.33  (-1.06 to 0.39) |
| Inducing clinical remission | Adalimumab+azathioprine | Certolizumab Pegol | 0.24  (-0.85 to 1.37) | 0.18  (-0.63 to 0.97) | 0.23  (-0.84 to 1.36) | 0.02  (-0.95 to 1.03) | N/A | N/A | 0.28  (-0.71 to 1.33) | 0.48  (-0.19 to 1.25) |
| Inducing clinical remission | Adalimumab+azathioprine | Ustekinumab | -0.41  (-1.38 to 0.66) | -0.50  (-1.25 to 0.23) | -0.41  (-1.38 to 0.65) | -0.57  (-1.45 to 0.37) | N/A | N/A | -0.51  (-1.43 to 0.45) | -0.05  (-0.59 to 0.68) |
| Inducing clinical remission | Adalimumab+azathioprine | Risankizumab | -0.23  (-1.35 to 0.89) | -0.28  (-1.13 to 0.54) | -0.24  (-1.34 to 0.89) | -0.45  (-1.46 to 0.58) | N/A | N/A | -0.19  (-1.21 to 0.86) | 0.10  (-0.60 to 0.88) |
| Inducing clinical remission | Adalimumab+azathioprine | Vedolizumab | 0.10  (-0.96 to 1.25) | 0.01  (-0.82 to 0.83) | 0.10  (-0.95 to 1.23) | -0.13  (-1.11 to 0.91) | N/A | N/A | 0.14  (-0.84 to 1.19) | 0.38  (-0.27 to 1.17) |
| Inducing clinical remission | Adalimumab+azathioprine | placebo | 0.53  (-0.45 to 1.53) | 0.46  (-0.29 to 1.20) | 0.52  (-0.44 to 1.53) | 0.30  (-0.58 to 1.23) | N/A | N/A | 0.57  (-0.33 to 1.50) | 0.68  (0.12 to 1.34) |
| Inducing clinical remission | BI 695501 | Certolizumab Pegol | 1.34  (0.25 to 2.50) | 1.28  (0.45 to 2.11) | 1.33  (0.24 to 2.49) | N/A | N/A | 1.33  (0.24 to 2.50) | 1.38  (0.37 to 2.45) | 0.81  (0.13 to 1.59) |
| Inducing clinical remission | BI 695501 | Ustekinumab | 0.69  (-0.29 to 1.79) | 0.60  (-0.17 to 1.38) | 0.69  (-0.30 to 1.78) | N/A | N/A | 0.69  (-0.30 to 1.78) | 0.58  (-0.35 to 1.57) | 0.28  (-0.27 to 1.02) |
| Inducing clinical remission | BI 695501 | Risankizumab | 0.87  (-0.25 to 2.03) | 0.81  (-0.05 to 1.68) | 0.86  (-0.26 to 2.02) | N/A | N/A | 0.86  (-0.26 to 2.01) | 0.90  (-0.13 to 1.98) | 0.44  (-0.28 to 1.23) |
| Inducing clinical remission | BI 695501 | Vedolizumab | 1.20  (0.13 to 2.37) | 1.11  (0.26 to 1.97) | 1.20  (0.13 to 2.37) | N/A | N/A | 1.19  (0.13 to 2.37) | 1.24  (0.23 to 2.32) | 0.72  (0.04 to 1.52) |
| Inducing clinical remission | BI 695501 | placebo | 1.62  (0.65 to 2.66) | 1.56  (0.79 to 2.34) | 1.62  (0.64 to 2.66) | N/A | N/A | 1.62  (0.64 to 2.66) | 1.66  (0.75 to 2.63) | 1.01  (0.44 to 1.69) |
| Inducing clinical remission | Certolizumab Pegol | Ustekinumab | -0.65  (-1.27 to 0.04) | -0.68  (-1.08 to -0.27) | -0.65  (-1.27 to 0.03) | -0.59  (-1.12 to -0.03) | N/A | -0.65  (-1.27 to 0.03) | -0.79  (-1.40 to -0.19) | -0.52  (-1.00 to 0.02) |
| Inducing clinical remission | Certolizumab Pegol | Risankizumab | -0.47  (-1.23 to 0.24) | -0.46  (-0.95 to 0.02) | -0.47  (-1.22 to 0.24) | -0.46  (-1.10 to 0.14) | N/A | -0.47  (-1.22 to 0.24) | -0.47  (-1.14 to 0.17) | -0.38  (-0.96 to 0.19) |
| Inducing clinical remission | Certolizumab Pegol | Vedolizumab | -0.14  (-0.80 to 0.58) | -0.17  (-0.63 to 0.30) | -0.14  (-0.79 to 0.58) | -0.15  (-0.72 to 0.46) | N/A | -0.14  (-0.80 to 0.58) | -0.14  (-0.75 to 0.49) | -0.10  (-0.63 to 0.47) |
| Inducing clinical remission | Certolizumab Pegol | placebo | 0.28  (-0.22 to 0.79) | 0.28  (0.00 to 0.57) | 0.28  (-0.21 to 0.79) | 0.29  (-0.12 to 0.70) | N/A | 0.28  (-0.22 to 0.79) | 0.29  (-0.15 to 0.72) | 0.20  (-0.18 to 0.59) |
| Inducing clinical remission | Ustekinumab | Risankizumab | 0.17  (-0.55 to 0.81) | 0.22  (-0.27 to 0.69) | 0.17  (-0.54 to 0.82) | 0.12  (-0.50 to 0.69) | N/A | 0.17  (-0.55 to 0.81) | 0.32  (-0.33 to 0.95) | 0.15  (-0.43 to 0.66) |
| Inducing clinical remission | Ustekinumab | Vedolizumab | 0.51  (-0.12 to 1.14) | 0.51  (0.05 to 0.97) | 0.51  (-0.11 to 1.13) | 0.44  (-0.12 to 1.00) | N/A | 0.51  (-0.12 to 1.14) | 0.65  (0.06 to 1.27) | 0.43  (-0.09 to 0.92) |
| Inducing clinical remission | Ustekinumab | placebo | 0.93  (0.49 to 1.33) | 0.96  (0.68 to 1.25) | 0.93  (0.50 to 1.33) | 0.87  (0.50 to 1.23) | N/A | 0.93  (0.49 to 1.33) | 1.08  (0.67 to 1.50) | 0.72  (0.37 to 1.03) |
| Inducing clinical remission | Risankizumab | Vedolizumab | 0.34  (-0.35 to 1.09) | 0.30  (-0.23 to 0.83) | 0.34  (-0.34 to 1.09) | 0.32  (-0.30 to 0.98) | N/A | 0.34  (-0.34 to 1.09) | 0.33  (-0.30 to 1.01) | 0.28  (-0.28 to 0.89) |
| Inducing clinical remission | Risankizumab | placebo | 0.76  (0.24 to 1.31) | 0.74  (0.37 to 1.14) | 0.76  (0.25 to 1.31) | 0.75  (0.30 to 1.24) | N/A | 0.76  (0.25 to 1.31) | 0.76  (0.29 to 1.26) | 0.58  (0.16 to 1.02) |
| Inducing clinical remission | Vedolizumab | placebo | 0.42  (-0.07 to 0.88) | 0.45  (0.09 to 0.82) | 0.42  (-0.07 to 0.88) | 0.43  (-0.01 to 0.85) | N/A | 0.42  (-0.07 to 0.88) | 0.42  (-0.03 to 0.86) | 0.30  (-0.11 to 0.68) |
| Inducing CDAI-70 | Infliximab | Infliximab+azathioprine | -0.45  (-1.63 to 0.72) | -0.45  (-0.92 to 0.01) | -0.46  (-1.62 to 0.72) | N/A | N/A | -0.45  (-1.64 to 0.74) | N/A | -0.13  (-0.59 to 0.33) |
| Inducing CDAI-70 | Infliximab | CT-P13 | 0.25  (-0.97 to 1.46) | 0.25  (-0.35 to 0.84) | 0.24  (-0.96 to 1.47) | N/A | N/A | 0.25  (-0.98 to 1.48) | N/A | 0.06  (-0.40 to 0.53) |
| Inducing CDAI-70 | Infliximab | Adalimumab | 2.05  (0.16 to 4.08) | 2.13  (0.73 to 3.81) | 2.05  (0.19 to 4.10) | N/A | N/A | 2.06  (0.19 to 4.13) | N/A | 0.86  (-0.02 to 1.97) |
| Inducing CDAI-70 | Infliximab | Adalimumab+azathioprine | 2.80  (0.46 to 5.24) | 2.89  (1.21 to 4.80) | 2.81  (0.47 to 5.28) | N/A | N/A | N/A | N/A | 0.96  (-0.03 to 2.15) |
| Inducing CDAI-70 | Infliximab | BI 695501 | 2.78  (0.27 to 5.39) | 2.86  (0.92 to 5.02) | 2.78  (0.24 to 5.42) | N/A | N/A | 2.78  (0.25 to 5.49) | N/A | 0.92  (-0.06 to 2.11) |
| Inducing CDAI-70 | Infliximab | Certolizumab Pegol | 2.92  (0.85 to 5.15) | 2.92  (1.51 to 4.58) | 2.90  (0.87 to 5.16) | N/A | N/A | 2.92  (0.84 to 5.18) | N/A | 1.28  (0.33 to 2.45) |
| Inducing CDAI-70 | Infliximab | Ustekinumab | 2.43  (0.57 to 4.49) | 2.44  (1.06 to 4.10) | 2.42  (0.58 to 4.50) | N/A | N/A | 2.44  (0.57 to 4.52) | N/A | 1.02  (0.15 to 2.13) |
| Inducing CDAI-70 | Infliximab | placebo | 3.24  (1.49 to 5.19) | 3.25  (1.88 to 4.89) | 3.23  (1.51 to 5.22) | N/A | N/A | 3.25  (1.51 to 5.25) | N/A | 1.46  (0.64 to 2.54) |
| Inducing CDAI-70 | Infliximab+azathioprine | CT-P13 | 0.69  (-0.99 to 2.38) | 0.70  (-0.05 to 1.46) | 0.70  (-0.98 to 2.39) | N/A | N/A | 0.70  (-1.01 to 2.41) | N/A | 0.20  (-0.46 to 0.86) |
| Inducing CDAI-70 | Infliximab+azathioprine | Adalimumab | 2.51  (0.27 to 4.83) | 2.59  (1.11 to 4.31) | 2.50  (0.31 to 4.83) | N/A | N/A | 2.52  (0.29 to 4.87) | N/A | 1.00  (0.01 to 2.19) |
| Inducing CDAI-70 | Infliximab+azathioprine | Adalimumab+azathioprine | 3.25  (0.63 to 5.95) | 3.34  (1.60 to 5.30) | 3.26  (0.65 to 5.97) | N/A | N/A | N/A | N/A | 1.09  (0.01 to 2.36) |
| Inducing CDAI-70 | Infliximab+azathioprine | BI 695501 | 3.24  (0.46 to 6.08) | 3.31  (1.32 to 5.52) | 3.24  (0.45 to 6.11) | N/A | N/A | 3.24  (0.45 to 6.17) | N/A | 1.05  (-0.03 to 2.33) |
| Inducing CDAI-70 | Infliximab+azathioprine | Certolizumab Pegol | 3.37  (1.00 to 5.90) | 3.37  (1.89 to 5.09) | 3.36  (1.03 to 5.88) | N/A | N/A | 3.38  (0.97 to 5.90) | N/A | 1.41  (0.36 to 2.67) |
| Inducing CDAI-70 | Infliximab+azathioprine | Ustekinumab | 2.88  (0.69 to 5.25) | 2.89  (1.44 to 4.61) | 2.88  (0.71 to 5.23) | N/A | N/A | 2.90  (0.68 to 5.27) | N/A | 1.16  (0.17 to 2.36) |
| Inducing CDAI-70 | Infliximab+azathioprine | placebo | 3.69  (1.60 to 5.97) | 3.70  (2.26 to 5.40) | 3.69  (1.63 to 5.96) | N/A | N/A | 3.71  (1.61 to 6.00) | N/A | 1.60  (0.67 to 2.77) |
| Inducing CDAI-70 | CT-P13 | Adalimumab | 1.82  (-0.45 to 4.16) | 1.89  (0.37 to 3.65) | 1.81  (-0.43 to 4.16) | N/A | N/A | 1.82  (-0.44 to 4.19) | N/A | 0.80  (-0.19 to 2.00) |
| Inducing CDAI-70 | CT-P13 | Adalimumab+azathioprine | 2.56  (-0.09 to 5.27) | 2.65  (0.86 to 4.63) | 2.56  (-0.07 to 5.30) | N/A | N/A | N/A | N/A | 0.90  (-0.19 to 2.17) |
| Inducing CDAI-70 | CT-P13 | BI 695501 | 2.54  (-0.25 to 5.41) | 2.61  (0.58 to 4.84) | 2.54  (-0.28 to 5.43) | N/A | N/A | 2.54  (-0.27 to 5.48) | N/A | 0.86  (-0.23 to 2.11) |
| Inducing CDAI-70 | CT-P13 | Certolizumab Pegol | 2.68  (0.29 to 5.21) | 2.67  (1.14 to 4.43) | 2.67  (0.29 to 5.21) | N/A | N/A | 2.68  (0.26 to 5.24) | N/A | 1.21  (0.16 to 2.47) |
| Inducing CDAI-70 | CT-P13 | Ustekinumab | 2.19  (-0.04 to 4.57) | 2.20  (0.69 to 3.95) | 2.19  (-0.03 to 4.56) | N/A | N/A | 2.19  (-0.04 to 4.58) | N/A | 0.96  (-0.03 to 2.16) |
| Inducing CDAI-70 | CT-P13 | placebo | 3.00  (0.88 to 5.29) | 3.00  (1.51 to 4.74) | 3.00  (0.88 to 5.30) | N/A | N/A | 3.00  (0.88 to 5.32) | N/A | 1.40  (0.46 to 2.57) |
| Inducing CDAI-70 | Adalimumab | Adalimumab+azathioprine | 0.73  (-0.63 to 2.15) | 0.74  (-0.15 to 1.72) | 0.75  (-0.62 to 2.16) | 0.74  (-0.62 to 2.15) | N/A | N/A | N/A | 0.09  (-0.36 to 0.56) |
| Inducing CDAI-70 | Adalimumab | BI 695501 | 0.71  (-0.93 to 2.47) | 0.70  (-0.57 to 2.12) | 0.71  (-0.94 to 2.44) | 0.71  (-0.91 to 2.47) | N/A | 0.70  (-0.96 to 2.47) | N/A | 0.05  (-0.40 to 0.51) |
| Inducing CDAI-70 | Adalimumab | Certolizumab Pegol | 0.85  (-0.37 to 2.20) | 0.79  (0.35 to 1.22) | 0.84  (-0.39 to 2.21) | 0.85  (-0.37 to 2.17) | N/A | 0.85  (-0.40 to 2.22) | N/A | 0.41  (-0.13 to 0.97) |
| Inducing CDAI-70 | Adalimumab | Ustekinumab | 0.37  (-0.49 to 1.35) | 0.31  (-0.08 to 0.70) | 0.37  (-0.50 to 1.33) | 0.37  (-0.49 to 1.32) | N/A | 0.37  (-0.51 to 1.36) | N/A | 0.15  (-0.23 to 0.56) |
| Inducing CDAI-70 | Adalimumab | placebo | 1.18  (0.60 to 1.88) | 1.12  (0.82 to 1.42) | 1.18  (0.60 to 1.87) | 1.18  (0.61 to 1.86) | N/A | 1.18  (0.60 to 1.88) | N/A | 0.60  (0.34 to 0.89) |
| Inducing CDAI-70 | Adalimumab+azathioprine | BI 695501 | -0.02  (-2.19 to 2.18) | -0.04  (-1.64 to 1.63) | -0.04  (-2.20 to 2.16) | -0.02  (-2.18 to 2.21) | N/A | N/A | N/A | -0.04  (-0.68 to 0.61) |
| Inducing CDAI-70 | Adalimumab+azathioprine | Certolizumab Pegol | 0.11  (-1.73 to 2.04) | 0.04  (-1.02 to 1.04) | 0.10  (-1.76 to 2.05) | 0.11  (-1.72 to 2.02) | N/A | N/A | N/A | 0.31  (-0.39 to 1.04) |
| Inducing CDAI-70 | Adalimumab+azathioprine | Ustekinumab | -0.37  (-2.01 to 1.33) | -0.44  (-1.47 to 0.54) | -0.38  (-2.02 to 1.31) | -0.37  (-1.99 to 1.30) | N/A | N/A | N/A | 0.06  (-0.53 to 0.67) |
| Inducing CDAI-70 | Adalimumab+azathioprine | placebo | 0.45  (-1.06 to 2.00) | 0.37  (-0.64 to 1.32) | 0.44  (-1.07 to 1.99) | 0.45  (-1.05 to 1.99) | N/A | N/A | N/A | 0.50  (-0.01 to 1.05) |
| Inducing CDAI-70 | BI 695501 | Certolizumab Pegol | 0.14  (-1.96 to 2.26) | 0.08  (-1.40 to 1.43) | 0.14  (-1.95 to 2.26) | 0.14  (-1.98 to 2.23) | N/A | 0.15  (-1.98 to 2.30) | N/A | 0.36  (-0.34 to 1.08) |
| Inducing CDAI-70 | BI 695501 | Ustekinumab | -0.34  (-2.28 to 1.57) | -0.39  (-1.86 to 0.94) | -0.34  (-2.26 to 1.57) | -0.34  (-2.28 to 1.54) | N/A | -0.33  (-2.29 to 1.60) | N/A | 0.10  (-0.49 to 0.72) |
| Inducing CDAI-70 | BI 695501 | placebo | 0.47  (-1.37 to 2.26) | 0.41  (-1.03 to 1.72) | 0.48  (-1.33 to 2.26) | 0.47  (-1.36 to 2.25) | N/A | 0.48  (-1.36 to 2.29) | N/A | 0.55  (0.03 to 1.09) |
| Inducing CDAI-70 | Certolizumab Pegol | Ustekinumab | -0.48  (-1.80 to 0.82) | -0.47  (-0.87 to -0.08) | -0.48  (-1.79 to 0.83) | -0.48  (-1.77 to 0.80) | N/A | -0.48  (-1.83 to 0.84) | N/A | -0.26  (-0.81 to 0.30) |
| Inducing CDAI-70 | Certolizumab Pegol | placebo | 0.33  (-0.79 to 1.45) | 0.33  (0.02 to 0.64) | 0.33  (-0.80 to 1.46) | 0.33  (-0.78 to 1.44) | N/A | 0.33  (-0.82 to 1.48) | N/A | 0.19  (-0.29 to 0.67) |
| Inducing CDAI-70 | Ustekinumab | placebo | 0.81  (0.15 to 1.48) | 0.81  (0.57 to 1.04) | 0.81  (0.15 to 1.49) | 0.81  (0.16 to 1.48) | N/A | 0.81  (0.13 to 1.50) | N/A | 0.45  (0.16 to 0.73) |
| Inducing CDAI-100 | Adalimumab | BI 695501 | -0.16  (-1.19 to 0.86) | -0.15  (-1.10 to 0.77) | -0.15  (-1.17 to 0.86) | N/A | N/A | N/A | -0.16  (-1.22 to 0.88) | -0.02  (-0.29 to 0.25) |
| Inducing CDAI-100 | Adalimumab | Certolizumab Pegol | 0.48  (-0.04 to 1.06) | 0.47  (0.07 to 0.86) | 0.49  (-0.04 to 1.07) | 0.43  (-0.09 to 1.02) | N/A | N/A | 0.53  (-0.08 to 1.22) | 0.31  (-0.01 to 0.63) |
| Inducing CDAI-100 | Adalimumab | Ustekinumab | -0.04  (-0.44 to 0.39) | -0.05  (-0.36 to 0.26) | -0.03  (-0.44 to 0.40) | 0.03  (-0.38 to 0.48) | N/A | N/A | 0.03  (-0.56 to 0.68) | -0.02  (-0.22 to 0.19) |
| Inducing CDAI-100 | Adalimumab | Risankizumab | 0.06  (-0.48 to 0.61) | 0.05  (-0.37 to 0.47) | 0.06  (-0.48 to 0.63) | 0.00  (-0.53 to 0.58) | N/A | N/A | 0.10  (-0.53 to 0.77) | 0.02  (-0.32 to 0.34) |
| Inducing CDAI-100 | Adalimumab | Vedolizumab | 0.39  (-0.11 to 0.95) | 0.36  (-0.05 to 0.76) | 0.40  (-0.11 to 0.95) | 0.34  (-0.15 to 0.90) | N/A | N/A | 0.44  (-0.14 to 1.10) | 0.23  (-0.08 to 0.55) |
| Inducing CDAI-100 | Adalimumab | placebo | 0.83  (0.47 to 1.23) | 0.81  (0.52 to 1.11) | 0.84  (0.47 to 1.24) | 0.78  (0.42 to 1.20) | N/A | N/A | 0.88  (0.42 to 1.39) | 0.53  (0.32 to 0.75) |
| Inducing CDAI-100 | BI 695501 | Certolizumab Pegol | 0.65  (-0.51 to 1.83) | 0.62  (-0.38 to 1.64) | 0.65  (-0.49 to 1.81) | N/A | N/A | N/A | 0.70  (-0.51 to 1.96) | 0.33  (-0.09 to 0.75) |
| Inducing CDAI-100 | BI 695501 | Ustekinumab | 0.12  (-0.98 to 1.25) | 0.10  (-0.87 to 1.09) | 0.11  (-0.98 to 1.23) | N/A | N/A | N/A | 0.19  (-1.00 to 1.43) | 0.00  (-0.33 to 0.34) |
| Inducing CDAI-100 | BI 695501 | Risankizumab | 0.22  (-0.94 to 1.40) | 0.20  (-0.81 to 1.23) | 0.21  (-0.93 to 1.37) | N/A | N/A | N/A | 0.27  (-0.94 to 1.52) | 0.04  (-0.39 to 0.45) |
| Inducing CDAI-100 | BI 695501 | Vedolizumab | 0.55  (-0.58 to 1.72) | 0.51  (-0.50 to 1.53) | 0.54  (-0.59 to 1.71) | N/A | N/A | N/A | 0.60  (-0.57 to 1.86) | 0.25  (-0.16 to 0.68) |
| Inducing CDAI-100 | BI 695501 | placebo | 1.00  (-0.09 to 2.10) | 0.97  (0.00 to 1.95) | 0.99  (-0.09 to 2.08) | N/A | N/A | N/A | 1.04  (-0.09 to 2.22) | 0.55  (0.21 to 0.90) |
| Inducing CDAI-100 | Certolizumab Pegol | Ustekinumab | -0.53  (-1.03 to -0.03) | -0.52  (-0.86 to -0.18) | -0.53  (-1.05 to -0.03) | -0.39  (-0.96 to 0.13) | N/A | N/A | -0.51  (-1.09 to 0.07) | -0.33  (-0.63 to -0.03) |
| Inducing CDAI-100 | Certolizumab Pegol | Risankizumab | -0.42  (-1.00 to 0.12) | -0.42  (-0.81 to -0.03) | -0.43  (-1.01 to 0.12) | -0.43  (-0.99 to 0.11) | N/A | N/A | -0.43  (-1.06 to 0.16) | -0.29  (-0.64 to 0.04) |
| Inducing CDAI-100 | Certolizumab Pegol | Vedolizumab | -0.10  (-0.61 to 0.44) | -0.11  (-0.49 to 0.26) | -0.10  (-0.61 to 0.44) | -0.10  (-0.60 to 0.43) | N/A | N/A | -0.10  (-0.65 to 0.49) | -0.09  (-0.41 to 0.25) |
| Inducing CDAI-100 | Certolizumab Pegol | placebo | 0.34  (-0.04 to 0.73) | 0.35  (0.09 to 0.60) | 0.34  (-0.06 to 0.73) | 0.35  (-0.04 to 0.73) | N/A | N/A | 0.34  (-0.09 to 0.77) | 0.22  (-0.01 to 0.45) |
| Inducing CDAI-100 | Ustekinumab | Risankizumab | 0.10  (-0.42 to 0.59) | 0.10  (-0.27 to 0.48) | 0.10  (-0.41 to 0.60) | -0.03  (-0.57 to 0.52) | N/A | N/A | 0.07  (-0.51 to 0.64) | 0.04  (-0.28 to 0.34) |
| Inducing CDAI-100 | Ustekinumab | Vedolizumab | 0.43  (-0.03 to 0.91) | 0.41  (0.05 to 0.77) | 0.43  (-0.03 to 0.92) | 0.30  (-0.19 to 0.84) | N/A | N/A | 0.41  (-0.11 to 0.97) | 0.25  (-0.05 to 0.55) |
| Inducing CDAI-100 | Ustekinumab | placebo | 0.87  (0.56 to 1.19) | 0.87  (0.64 to 1.10) | 0.87  (0.56 to 1.19) | 0.74  (0.39 to 1.13) | N/A | N/A | 0.85  (0.47 to 1.23) | 0.55  (0.36 to 0.73) |
| Inducing CDAI-100 | Risankizumab | Vedolizumab | 0.33  (-0.18 to 0.88) | 0.31  (-0.09 to 0.71) | 0.33  (-0.18 to 0.88) | 0.34  (-0.18 to 0.87) | N/A | N/A | 0.34  (-0.22 to 0.94) | 0.21  (-0.12 to 0.57) |
| Inducing CDAI-100 | Risankizumab | placebo | 0.77  (0.39 to 1.18) | 0.77  (0.47 to 1.06) | 0.77  (0.38 to 1.18) | 0.78  (0.40 to 1.17) | N/A | N/A | 0.77  (0.36 to 1.21) | 0.51  (0.27 to 0.77) |
| Inducing CDAI-100 | Vedolizumab | placebo | 0.44  (0.08 to 0.78) | 0.46  (0.19 to 0.73) | 0.44  (0.08 to 0.78) | 0.44  (0.09 to 0.78) | N/A | N/A | 0.44  (0.04 to 0.80) | 0.30  (0.06 to 0.53) |
| Risk of AEs in induction therapy | Adalimumab | Certolizumab Pegol | -0.44  (-1.32 to 0.50) | -0.49  (-0.98 to 0.00) | -0.43  (-1.32 to 0.51) | -0.17  (-0.89 to 0.55) | N/A | N/A | N/A | -0.19  (-0.55 to 0.18) |
| Risk of AEs in induction therapy | Adalimumab | Ustekinumab | -0.16  (-0.79 to 0.55) | -0.24  (-0.63 to 0.15) | -0.16  (-0.79 to 0.54) | 0.09  (-0.49 to 0.67) | N/A | N/A | N/A | -0.07  (-0.30 to 0.19) |
| Risk of AEs in induction therapy | Adalimumab | Risankizumab | 0.13  (-0.54 to 0.85) | 0.05  (-0.35 to 0.45) | 0.13  (-0.53 to 0.85) | 0.08  (-0.59 to 0.77) | N/A | N/A | N/A | 0.04  (-0.21 to 0.30) |
| Risk of AEs in induction therapy | Adalimumab | Vedolizumab | -0.31  (-0.95 to 0.33) | -0.31  (-0.71 to 0.08) | -0.31  (-0.95 to 0.32) | -0.02  (-0.62 to 0.55) | N/A | N/A | N/A | -0.12  (-0.35 to 0.12) |
| Risk of AEs in induction therapy | Adalimumab | placebo | -0.25  (-0.71 to 0.25) | -0.30  (-0.61 to 0.00) | -0.25  (-0.70 to 0.25) | 0.02  (-0.47 to 0.49) | N/A | N/A | N/A | -0.10  (-0.27 to 0.08) |
| Risk of AEs in induction therapy | Certolizumab Pegol | Ustekinumab | 0.28  (-0.61 to 1.20) | 0.25  (-0.20 to 0.69) | 0.27  (-0.62 to 1.20) | 0.26  (-0.37 to 0.90) | N/A | N/A | N/A | 0.13  (-0.23 to 0.49) |
| Risk of AEs in induction therapy | Certolizumab Pegol | Risankizumab | 0.57  (-0.36 to 1.50) | 0.54  (0.09 to 1.00) | 0.56  (-0.36 to 1.49) | 0.25  (-0.45 to 0.99) | N/A | N/A | N/A | 0.23  (-0.13 to 0.59) |
| Risk of AEs in induction therapy | Certolizumab Pegol | Vedolizumab | 0.13  (-0.79 to 0.98) | 0.18  (-0.27 to 0.63) | 0.12  (-0.80 to 0.99) | 0.15  (-0.50 to 0.76) | N/A | N/A | N/A | 0.07  (-0.28 to 0.42) |
| Risk of AEs in induction therapy | Certolizumab Pegol | placebo | 0.19  (-0.59 to 0.96) | 0.19  (-0.19 to 0.56) | 0.18  (-0.60 to 0.96) | 0.19  (-0.36 to 0.73) | N/A | N/A | N/A | 0.09  (-0.22 to 0.40) |
| Risk of AEs in induction therapy | Ustekinumab | Risankizumab | 0.29  (-0.41 to 0.97) | 0.29  (-0.06 to 0.64) | 0.29  (-0.40 to 0.97) | -0.01  (-0.58 to 0.58) | N/A | N/A | N/A | 0.10  (-0.14 to 0.35) |
| Risk of AEs in induction therapy | Ustekinumab | Vedolizumab | -0.16  (-0.83 to 0.45) | -0.07  (-0.41 to 0.27) | -0.15  (-0.82 to 0.44) | -0.11  (-0.61 to 0.33) | N/A | N/A | N/A | -0.06  (-0.29 to 0.17) |
| Risk of AEs in induction therapy | Ustekinumab | placebo | -0.09  (-0.57 to 0.36) | -0.06  (-0.30 to 0.17) | -0.09  (-0.56 to 0.36) | -0.08  (-0.42 to 0.25) | N/A | N/A | N/A | -0.04  (-0.21 to 0.13) |
| Risk of AEs in induction therapy | Risankizumab | Vedolizumab | -0.44  (-1.13 to 0.19) | -0.36  (-0.72 to 0.00) | -0.44  (-1.13 to 0.19) | -0.10  (-0.72 to 0.44) | N/A | N/A | N/A | -0.16  (-0.40 to 0.07) |
| Risk of AEs in induction therapy | Risankizumab | placebo | -0.38  (-0.89 to 0.12) | -0.35  (-0.61 to -0.10) | -0.38  (-0.88 to 0.12) | -0.07  (-0.56 to 0.39) | N/A | N/A | N/A | -0.14  (-0.32 to 0.03) |
| Risk of AEs in induction therapy | Vedolizumab | placebo | 0.06  (-0.34 to 0.52) | 0.01  (-0.24 to 0.25) | 0.06  (-0.35 to 0.52) | 0.03  (-0.27 to 0.38) | N/A | N/A | N/A | 0.02  (-0.13 to 0.18) |
| Risk of SAEs in induction therapy | Adalimumab | Certolizumab Pegol | -1.02  (-2.57 to 0.46) | -1.05  (-2.38 to 0.23) | -1.06  (-2.57 to 0.48) | -0.56  (-2.26 to 1.17) | N/A | N/A | N/A | -1.13  (-2.56 to 0.24) |
| Risk of SAEs in induction therapy | Adalimumab | Ustekinumab | -0.47  (-1.67 to 0.73) | -0.49  (-1.55 to 0.51) | -0.46  (-1.60 to 0.71) | 0.04  (-1.36 to 1.47) | N/A | N/A | N/A | -0.62  (-1.71 to 0.42) |
| Risk of SAEs in induction therapy | Adalimumab | Risankizumab | 0.40  (-0.74 to 1.59) | 0.35  (-0.68 to 1.33) | 0.39  (-0.71 to 1.57) | 0.91  (-0.49 to 2.34) | N/A | N/A | N/A | 0.09  (-0.95 to 1.15) |
| Risk of SAEs in induction therapy | Adalimumab | Vedolizumab | -0.68  (-1.83 to 0.47) | -0.68  (-1.71 to 0.31) | -0.67  (-1.79 to 0.46) | -0.18  (-1.56 to 1.22) | N/A | N/A | N/A | -0.81  (-1.85 to 0.20) |
| Risk of SAEs in induction therapy | Adalimumab | placebo | -0.64  (-1.66 to 0.34) | -0.64  (-1.59 to 0.23) | -0.64  (-1.62 to 0.33) | -0.14  (-1.40 to 1.14) | N/A | N/A | N/A | -0.86  (-1.81 to 0.02) |
| Risk of SAEs in induction therapy | Certolizumab Pegol | Ustekinumab | 0.56  (-0.68 to 1.93) | 0.55  (-0.48 to 1.63) | 0.59  (-0.70 to 1.95) | 0.60  (-0.69 to 1.93) | N/A | N/A | N/A | 0.51  (-0.64 to 1.73) |
| Risk of SAEs in induction therapy | Certolizumab Pegol | Risankizumab | 1.44  (0.20 to 2.80) | 1.39  (0.39 to 2.45) | 1.45  (0.17 to 2.82) | 1.45  (0.21 to 2.81) | N/A | N/A | N/A | 1.23  (0.09 to 2.42) |
| Risk of SAEs in induction therapy | Certolizumab Pegol | Vedolizumab | 0.36  (-0.89 to 1.66) | 0.36  (-0.65 to 1.43) | 0.38  (-0.89 to 1.68) | 0.38  (-0.87 to 1.67) | N/A | N/A | N/A | 0.33  (-0.80 to 1.48) |
| Risk of SAEs in induction therapy | Certolizumab Pegol | placebo | 0.39  (-0.72 to 1.57) | 0.39  (-0.52 to 1.36) | 0.41  (-0.74 to 1.59) | 0.41  (-0.72 to 1.59) | N/A | N/A | N/A | 0.27  (-0.77 to 1.32) |
| Risk of SAEs in induction therapy | Ustekinumab | Risankizumab | 0.87  (0.01 to 1.76) | 0.84  (0.20 to 1.49) | 0.86  (0.01 to 1.75) | 0.86  (0.03 to 1.74) | N/A | N/A | N/A | 0.72  (-0.03 to 1.48) |
| Risk of SAEs in induction therapy | Ustekinumab | Vedolizumab | -0.21  (-1.08 to 0.63) | -0.19  (-0.84 to 0.46) | -0.21  (-1.06 to 0.61) | -0.21  (-1.07 to 0.60) | N/A | N/A | N/A | -0.18  (-0.93 to 0.54) |
| Risk of SAEs in induction therapy | Ustekinumab | placebo | -0.18  (-0.84 to 0.43) | -0.16  (-0.65 to 0.33) | -0.18  (-0.83 to 0.42) | -0.18  (-0.83 to 0.43) | N/A | N/A | N/A | -0.24  (-0.82 to 0.30) |
| Risk of SAEs in induction therapy | Risankizumab | Vedolizumab | -1.07  (-1.95 to -0.29) | -1.03  (-1.64 to -0.43) | -1.06  (-1.94 to -0.29) | -1.07  (-1.94 to -0.30) | N/A | N/A | N/A | -0.90  (-1.63 to -0.22) |
| Risk of SAEs in induction therapy | Risankizumab | placebo | -1.04  (-1.71 to -0.48) | -1.00  (-1.43 to -0.58) | -1.04  (-1.70 to -0.48) | -1.04  (-1.70 to -0.48) | N/A | N/A | N/A | -0.95  (-1.51 to -0.47) |
| Risk of SAEs in induction therapy | Vedolizumab | placebo | 0.03  (-0.53 to 0.59) | 0.03  (-0.40 to 0.47) | 0.02  (-0.53 to 0.60) | 0.03  (-0.52 to 0.59) | N/A | N/A | N/A | -0.06  (-0.54 to 0.42) |
| Risk of SIs in induction therapy | Adalimumab | Certolizumab Pegol | 0.18  (-4.52 to 4.94) | 0.06  (-2.65 to 2.78) | 0.23  (-4.43 to 5.08) | N/A | N/A | N/A | N/A | -0.18  (-4.23 to 3.78) |
| Risk of SIs in induction therapy | Adalimumab | Ustekinumab | -1.28  (-5.02 to 2.52) | -1.44  (-3.75 to 0.52) | -1.19  (-4.93 to 2.60) | N/A | N/A | N/A | N/A | -1.43  (-4.65 to 1.54) |
| Risk of SIs in induction therapy | Adalimumab | Risankizumab | 0.69  (-3.04 to 4.46) | 0.58  (-1.72 to 2.60) | 0.76  (-2.97 to 4.56) | N/A | N/A | N/A | N/A | 0.25  (-3.02 to 3.25) |
| Risk of SIs in induction therapy | Adalimumab | Vedolizumab | -1.22  (-5.77 to 3.02) | -1.04  (-3.96 to 1.56) | -1.11  (-5.63 to 3.13) | N/A | N/A | N/A | N/A | -0.76  (-4.46 to 2.65) |
| Risk of SIs in induction therapy | Adalimumab | placebo | -0.68  (-3.84 to 2.43) | -0.78  (-2.89 to 0.94) | -0.60  (-3.77 to 2.55) | N/A | N/A | N/A | N/A | -1.25  (-4.07 to 1.15) |
| Risk of SIs in induction therapy | Certolizumab Pegol | Ustekinumab | -1.46  (-5.54 to 2.60) | -1.50  (-3.82 to 0.47) | -1.43  (-5.55 to 2.62) | N/A | N/A | N/A | N/A | -1.25  (-4.79 to 2.18) |
| Risk of SIs in induction therapy | Certolizumab Pegol | Risankizumab | 0.51  (-3.58 to 4.55) | 0.52  (-1.81 to 2.53) | 0.53  (-3.56 to 4.57) | N/A | N/A | N/A | N/A | 0.44  (-3.13 to 3.84) |
| Risk of SIs in induction therapy | Certolizumab Pegol | Vedolizumab | -1.38  (-6.30 to 3.08) | -1.10  (-4.05 to 1.51) | -1.33  (-6.18 to 3.09) | N/A | N/A | N/A | N/A | -0.57  (-4.54 to 3.16) |
| Risk of SIs in induction therapy | Certolizumab Pegol | placebo | -0.86  (-4.45 to 2.61) | -0.83  (-2.96 to 0.88) | -0.84  (-4.43 to 2.62) | N/A | N/A | N/A | N/A | -1.06  (-4.24 to 1.84) |
| Risk of SIs in induction therapy | Ustekinumab | Risankizumab | 1.97  (-0.93 to 4.87) | 2.01  (0.68 to 3.45) | 1.95  (-0.91 to 4.86) | N/A | N/A | N/A | N/A | 1.69  (-0.76 to 4.08) |
| Risk of SIs in induction therapy | Ustekinumab | Vedolizumab | 0.08  (-3.86 to 3.56) | 0.43  (-1.90 to 2.55) | 0.10  (-3.79 to 3.56) | N/A | N/A | N/A | N/A | 0.68  (-2.30 to 3.55) |
| Risk of SIs in induction therapy | Ustekinumab | placebo | 0.59  (-1.49 to 2.62) | 0.65  (-0.26 to 1.64) | 0.58  (-1.48 to 2.63) | N/A | N/A | N/A | N/A | 0.18  (-1.58 to 1.80) |
| Risk of SIs in induction therapy | Risankizumab | Vedolizumab | -1.89  (-5.83 to 1.58) | -1.59  (-3.96 to 0.55) | -1.84  (-5.75 to 1.57) | N/A | N/A | N/A | N/A | -1.00  (-4.00 to 1.92) |
| Risk of SIs in induction therapy | Risankizumab | placebo | -1.37  (-3.47 to 0.63) | -1.35  (-2.42 to -0.40) | -1.36  (-3.45 to 0.64) | N/A | N/A | N/A | N/A | -1.51  (-3.29 to 0.17) |
| Risk of SIs in induction therapy | Vedolizumab | placebo | 0.51  (-2.36 to 3.84) | 0.22  (-1.67 to 2.36) | 0.48  (-2.37 to 3.74) | N/A | N/A | N/A | N/A | -0.18  (-4.23 to 3.78) |
| Maintaining clinical remission | IFX-5 | IFX-10 | -0.39  (-1.20 to 0.45) | -0.41  (-0.97 to 0.14) | -0.40  (-1.21 to 0.44) | -0.39  (-1.22 to 0.47) | -0.40  (-1.14 to 0.37) | -0.46  (-1.36 to 0.44) | N/A | -0.25  (-0.86 to 0.38) |
| Maintaining clinical remission | IFX-5 | Infliximab+azathioprine | -0.39  (-1.27 to 0.49) | -0.39  (-1.00 to 0.21) | -0.39  (-1.27 to 0.49) | N/A | N/A | -0.39  (-1.31 to 0.53) | N/A | -0.11  (-0.67 to 0.44) |
| Maintaining clinical remission | IFX-5 | CT-P13 | 0.08  (-0.76 to 0.92) | 0.08  (-0.46 to 0.61) | 0.08  (-0.75 to 0.92) | 0.08  (-0.78 to 0.94) | 0.08  (-0.68 to 0.84) | 0.08  (-0.80 to 0.96) | N/A | 0.03  (-0.54 to 0.60) |
| Maintaining clinical remission | IFX-5 | Adalimumab | -0.42  (-1.49 to 0.55) | -0.33  (-1.05 to 0.39) | -0.41  (-1.49 to 0.54) | -0.28  (-1.52 to 0.73) | -0.71  (-1.72 to 0.25) | -0.31  (-1.48 to 0.75) | N/A | -0.16  (-0.97 to 0.57) |
| Maintaining clinical remission | IFX-5 | Adalimumab+azathioprine | -0.25  (-1.65 to 1.06) | -0.16  (-1.10 to 0.79) | -0.24  (-1.64 to 1.04) | -0.10  (-1.67 to 1.22) | N/A | N/A | N/A | -0.08  (-1.08 to 0.82) |
| Maintaining clinical remission | IFX-5 | BI 695501 | -0.05  (-1.52 to 1.32) | 0.03  (-1.00 to 1.08) | -0.05  (-1.52 to 1.30) | 0.09  (-1.53 to 1.48) | -0.34  (-1.70 to 0.97) | 0.06  (-1.50 to 1.51) | N/A | -0.04  (-1.05 to 0.85) |
| Maintaining clinical remission | IFX-5 | Certolizumab Pegol | 0.09  (-0.95 to 1.11) | 0.10  (-0.59 to 0.80) | 0.09  (-0.94 to 1.10) | 0.09  (-0.97 to 1.13) | -0.01  (-1.06 to 1.05) | 0.19  (-0.92 to 1.33) | N/A | 0.07  (-0.72 to 0.86) |
| Maintaining clinical remission | IFX-5 | Ustekinumab | -0.18  (-1.19 to 0.85) | -0.18  (-0.89 to 0.55) | -0.17  (-1.20 to 0.84) | -0.11  (-1.19 to 0.91) | 0.14  (-0.84 to 1.13) | -0.07  (-1.17 to 1.07) | N/A | -0.01  (-0.77 to 0.77) |
| Maintaining clinical remission | IFX-5 | RZB-180 | 0.23  (-0.95 to 1.40) | 0.24  (-0.53 to 1.02) | 0.23  (-0.95 to 1.40) | 0.23  (-0.98 to 1.43) | 0.24  (-0.83 to 1.31) | 0.34  (-0.93 to 1.63) | N/A | 0.27  (-0.61 to 1.15) |
| Maintaining clinical remission | IFX-5 | RZB-360 | 0.35  (-0.83 to 1.52) | 0.36  (-0.42 to 1.15) | 0.35  (-0.83 to 1.52) | 0.35  (-0.86 to 1.55) | 0.36  (-0.72 to 1.43) | 0.46  (-0.82 to 1.75) | N/A | 0.32  (-0.56 to 1.21) |
| Maintaining clinical remission | IFX-5 | Vedolizumab | -0.07  (-1.19 to 1.00) | -0.04  (-0.83 to 0.74) | -0.07  (-1.19 to 1.00) | -0.07  (-1.22 to 1.02) | -0.06  (-1.09 to 0.93) | 0.04  (-1.17 to 1.21) | N/A | 0.02  (-0.81 to 0.85) |
| Maintaining clinical remission | IFX-5 | Vedolizumab SC | 0.25  (-0.93 to 1.42) | 0.26  (-0.51 to 1.03) | 0.25  (-0.93 to 1.41) | 0.25  (-0.96 to 1.44) | 0.26  (-0.81 to 1.32) | 0.36  (-0.92 to 1.64) | N/A | 0.24  (-0.65 to 1.12) |
| Maintaining clinical remission | IFX-5 | placebo | 0.82  (-0.05 to 1.70) | 0.83  (0.20 to 1.48) | 0.82  (-0.06 to 1.69) | 0.82  (-0.08 to 1.72) | 0.83  (0.02 to 1.64) | 0.93  (-0.04 to 1.92) | N/A | 0.56  (-0.11 to 1.25) |
| Maintaining clinical remission | IFX-10 | Infliximab+azathioprine | 0.00  (-1.22 to 1.19) | 0.02  (-0.80 to 0.84) | 0.00  (-1.22 to 1.20) | N/A | N/A | 0.07  (-1.22 to 1.35) | N/A | 0.14  (-0.71 to 0.95) |
| Maintaining clinical remission | IFX-10 | CT-P13 | 0.48  (-0.72 to 1.63) | 0.49  (-0.28 to 1.26) | 0.47  (-0.71 to 1.63) | 0.47  (-0.75 to 1.66) | 0.47  (-0.61 to 1.53) | 0.54  (-0.71 to 1.80) | N/A | 0.28  (-0.57 to 1.10) |
| Maintaining clinical remission | IFX-10 | Adalimumab | -0.03  (-1.00 to 0.83) | 0.08  (-0.58 to 0.75) | -0.02  (-0.99 to 0.83) | 0.10  (-1.03 to 1.01) | -0.31  (-1.23 to 0.56) | 0.15  (-1.01 to 1.20) | N/A | 0.09  (-0.63 to 0.73) |
| Maintaining clinical remission | IFX-10 | Adalimumab+azathioprine | 0.15  (-1.19 to 1.36) | 0.25  (-0.64 to 1.15) | 0.16  (-1.18 to 1.34) | 0.28  (-1.21 to 1.52) | N/A | N/A | N/A | 0.17  (-0.77 to 0.99) |
| Maintaining clinical remission | IFX-10 | BI 695501 | 0.34  (-1.06 to 1.62) | 0.44  (-0.54 to 1.45) | 0.35  (-1.05 to 1.62) | 0.48  (-1.08 to 1.80) | 0.05  (-1.24 to 1.30) | 0.52  (-1.03 to 1.96) | N/A | 0.21  (-0.73 to 1.02) |
| Maintaining clinical remission | IFX-10 | Certolizumab Pegol | 0.48  (-0.45 to 1.37) | 0.51  (-0.11 to 1.15) | 0.48  (-0.44 to 1.37) | 0.48  (-0.48 to 1.40) | 0.39  (-0.59 to 1.35) | 0.65  (-0.46 to 1.78) | N/A | 0.32  (-0.38 to 1.02) |
| Maintaining clinical remission | IFX-10 | Ustekinumab | 0.22  (-0.70 to 1.11) | 0.23  (-0.42 to 0.90) | 0.22  (-0.69 to 1.11) | 0.27  (-0.71 to 1.18) | 0.54  (-0.37 to 1.44) | 0.39  (-0.70 to 1.52) | N/A | 0.24  (-0.43 to 0.92) |
| Maintaining clinical remission | IFX-10 | RZB-180 | 0.62  (-0.48 to 1.68) | 0.65  (-0.06 to 1.37) | 0.63  (-0.47 to 1.69) | 0.62  (-0.51 to 1.71) | 0.64  (-0.36 to 1.62) | 0.80  (-0.46 to 2.08) | N/A | 0.52  (-0.29 to 1.31) |
| Maintaining clinical remission | IFX-10 | RZB-360 | 0.75  (-0.36 to 1.81) | 0.77  (0.05 to 1.50) | 0.75  (-0.36 to 1.80) | 0.74  (-0.39 to 1.83) | 0.76  (-0.25 to 1.74) | 0.92  (-0.35 to 2.20) | N/A | 0.58  (-0.24 to 1.37) |
| Maintaining clinical remission | IFX-10 | Vedolizumab | 0.32  (-0.71 to 1.29) | 0.36  (-0.36 to 1.10) | 0.32  (-0.69 to 1.28) | 0.32  (-0.74 to 1.29) | 0.34  (-0.61 to 1.23) | 0.49  (-0.70 to 1.66) | N/A | 0.27  (-0.48 to 1.01) |
| Maintaining clinical remission | IFX-10 | Vedolizumab SC | 0.64  (-0.45 to 1.70) | 0.67  (-0.04 to 1.38) | 0.65  (-0.45 to 1.69) | 0.64  (-0.49 to 1.72) | 0.66  (-0.34 to 1.62) | 0.82  (-0.44 to 2.09) | N/A | 0.49  (-0.33 to 1.28) |
| Maintaining clinical remission | IFX-10 | placebo | 1.21  (0.46 to 1.95) | 1.24  (0.69 to 1.82) | 1.22  (0.47 to 1.95) | 1.21  (0.44 to 1.96) | 1.23  (0.53 to 1.93) | 1.39  (0.44 to 2.37) | N/A | 0.82  (0.25 to 1.39) |
| Maintaining clinical remission | Infliximab+azathioprine | CT-P13 | 0.47  (-0.75 to 1.69) | 0.47  (-0.34 to 1.28) | 0.47  (-0.75 to 1.69) | N/A | N/A | 0.47  (-0.80 to 1.75) | N/A | 0.14  (-0.65 to 0.94) |
| Maintaining clinical remission | Infliximab+azathioprine | Adalimumab | -0.02  (-1.43 to 1.26) | 0.06  (-0.88 to 1.01) | -0.02  (-1.43 to 1.26) | N/A | N/A | 0.08  (-1.42 to 1.46) | N/A | -0.04  (-1.04 to 0.85) |
| Maintaining clinical remission | Infliximab+azathioprine | Adalimumab+azathioprine | 0.15  (-1.53 to 1.71) | 0.23  (-0.88 to 1.36) | 0.15  (-1.51 to 1.69) | N/A | N/A | N/A | N/A | 0.03  (-1.12 to 1.07) |
| Maintaining clinical remission | Infliximab+azathioprine | BI 695501 | 0.34  (-1.38 to 1.95) | 0.42  (-0.77 to 1.63) | 0.35  (-1.38 to 1.94) | N/A | N/A | 0.45  (-1.37 to 2.16) | N/A | 0.07  (-1.09 to 1.10) |
| Maintaining clinical remission | Infliximab+azathioprine | Certolizumab Pegol | 0.48  (-0.88 to 1.82) | 0.49  (-0.43 to 1.42) | 0.48  (-0.89 to 1.82) | N/A | N/A | 0.59  (-0.86 to 2.05) | N/A | 0.18  (-0.78 to 1.15) |
| Maintaining clinical remission | Infliximab+azathioprine | Ustekinumab | 0.22  (-1.13 to 1.57) | 0.21  (-0.72 to 1.16) | 0.22  (-1.13 to 1.56) | N/A | N/A | 0.33  (-1.11 to 1.79) | N/A | 0.10  (-0.83 to 1.06) |
| Maintaining clinical remission | Infliximab+azathioprine | RZB-180 | 0.63  (-0.85 to 2.09) | 0.63  (-0.35 to 1.62) | 0.62  (-0.86 to 2.09) | N/A | N/A | 0.73  (-0.84 to 2.32) | N/A | 0.38  (-0.66 to 1.42) |
| Maintaining clinical remission | Infliximab+azathioprine | RZB-360 | 0.74  (-0.74 to 2.21) | 0.75  (-0.24 to 1.74) | 0.74  (-0.74 to 2.21) | N/A | N/A | 0.85  (-0.72 to 2.44) | N/A | 0.43  (-0.61 to 1.48) |
| Maintaining clinical remission | Infliximab+azathioprine | Vedolizumab | 0.32  (-1.11 to 1.70) | 0.35  (-0.64 to 1.34) | 0.32  (-1.11 to 1.70) | N/A | N/A | 0.43  (-1.09 to 1.91) | N/A | 0.13  (-0.86 to 1.13) |
| Maintaining clinical remission | Infliximab+azathioprine | Vedolizumab SC | 0.65  (-0.83 to 2.10) | 0.65  (-0.33 to 1.63) | 0.64  (-0.84 to 2.10) | N/A | N/A | 0.75  (-0.82 to 2.33) | N/A | 0.35  (-0.69 to 1.39) |
| Maintaining clinical remission | Infliximab+azathioprine | placebo | 1.21  (-0.03 to 2.45) | 1.22  (0.35 to 2.11) | 1.21  (-0.04 to 2.45) | N/A | N/A | 1.32  (-0.01 to 2.67) | N/A | 0.68  (-0.19 to 1.55) |
| Maintaining clinical remission | CT-P13 | Adalimumab | -0.49  (-1.88 to 0.76) | -0.41  (-1.31 to 0.49) | -0.49  (-1.87 to 0.76) | -0.36  (-1.90 to 0.93) | -0.79  (-2.06 to 0.43) | -0.38  (-1.87 to 0.96) | N/A | -0.18  (-1.19 to 0.72) |
| Maintaining clinical remission | CT-P13 | Adalimumab+azathioprine | -0.32  (-1.98 to 1.21) | -0.24  (-1.32 to 0.85) | -0.31  (-1.96 to 1.19) | -0.18  (-1.99 to 1.36) | N/A | N/A | N/A | -0.11  (-1.27 to 0.94) |
| Maintaining clinical remission | CT-P13 | BI 695501 | -0.13  (-1.83 to 1.46) | -0.05  (-1.20 to 1.13) | -0.13  (-1.83 to 1.45) | 0.01  (-1.85 to 1.63) | -0.42  (-1.98 to 1.09) | -0.02  (-1.82 to 1.66) | N/A | -0.07  (-1.24 to 0.97) |
| Maintaining clinical remission | CT-P13 | Certolizumab Pegol | 0.01  (-1.32 to 1.32) | 0.02  (-0.85 to 0.90) | 0.01  (-1.33 to 1.32) | 0.00  (-1.36 to 1.36) | -0.08  (-1.38 to 1.22) | 0.12  (-1.31 to 1.55) | N/A | 0.04  (-0.93 to 1.02) |
| Maintaining clinical remission | CT-P13 | Ustekinumab | -0.26  (-1.57 to 1.07) | -0.26  (-1.15 to 0.65) | -0.25  (-1.58 to 1.06) | -0.20  (-1.58 to 1.14) | 0.06  (-1.18 to 1.31) | -0.14  (-1.55 to 1.29) | N/A | -0.04  (-0.98 to 0.93) |
| Maintaining clinical remission | CT-P13 | RZB-180 | 0.15  (-1.29 to 1.60) | 0.16  (-0.78 to 1.11) | 0.16  (-1.30 to 1.59) | 0.15  (-1.34 to 1.62) | 0.16  (-1.14 to 1.47) | 0.26  (-1.29 to 1.82) | N/A | 0.24  (-0.81 to 1.29) |
| Maintaining clinical remission | CT-P13 | RZB-360 | 0.27  (-1.18 to 1.72) | 0.28  (-0.66 to 1.23) | 0.27  (-1.18 to 1.71) | 0.27  (-1.22 to 1.75) | 0.28  (-1.04 to 1.59) | 0.38  (-1.17 to 1.94) | N/A | 0.30  (-0.75 to 1.34) |
| Maintaining clinical remission | CT-P13 | Vedolizumab | -0.15  (-1.55 to 1.20) | -0.12  (-1.07 to 0.83) | -0.15  (-1.55 to 1.20) | -0.16  (-1.60 to 1.23) | -0.14  (-1.42 to 1.11) | -0.04  (-1.54 to 1.42) | N/A | -0.01  (-1.01 to 1.00) |
| Maintaining clinical remission | CT-P13 | Vedolizumab SC | 0.17  (-1.28 to 1.61) | 0.18  (-0.75 to 1.12) | 0.17  (-1.28 to 1.60) | 0.17  (-1.32 to 1.64) | 0.18  (-1.13 to 1.49) | 0.28  (-1.27 to 1.83) | N/A | 0.21  (-0.85 to 1.26) |
| Maintaining clinical remission | CT-P13 | placebo | 0.74  (-0.47 to 1.96) | 0.75  (-0.07 to 1.59) | 0.75  (-0.48 to 1.95) | 0.74  (-0.51 to 1.98) | 0.75  (-0.35 to 1.86) | 0.85  (-0.46 to 2.17) | N/A | 0.54  (-0.34 to 1.42) |
| Maintaining clinical remission | Adalimumab | Adalimumab+azathioprine | 0.17  (-0.71 to 1.06) | 0.18  (-0.43 to 0.79) | 0.17  (-0.70 to 1.06) | 0.18  (-0.73 to 1.09) | N/A | N/A | N/A | 0.07  (-0.50 to 0.65) |
| Maintaining clinical remission | Adalimumab | BI 695501 | 0.36  (-0.61 to 1.35) | 0.36  (-0.37 to 1.12) | 0.36  (-0.61 to 1.35) | 0.37  (-0.63 to 1.37) | 0.36  (-0.54 to 1.29) | 0.37  (-0.65 to 1.38) | N/A | 0.11  (-0.45 to 0.67) |
| Maintaining clinical remission | Adalimumab | Certolizumab Pegol | 0.50  (-0.17 to 1.31) | 0.43  (0.00 to 0.87) | 0.49  (-0.17 to 1.30) | 0.36  (-0.37 to 1.37) | 0.70  (-0.14 to 1.60) | 0.50  (-0.19 to 1.36) | N/A | 0.22  (-0.26 to 0.83) |
| Maintaining clinical remission | Adalimumab | Ustekinumab | 0.24  (-0.27 to 0.90) | 0.16  (-0.18 to 0.50) | 0.23  (-0.27 to 0.89) | 0.16  (-0.38 to 0.93) | 0.85  (0.08 to 1.67) | 0.24  (-0.28 to 0.94) | N/A | 0.14  (-0.19 to 0.62) |
| Maintaining clinical remission | Adalimumab | RZB-180 | 0.65  (-0.22 to 1.66) | 0.57  (0.01 to 1.13) | 0.64  (-0.22 to 1.65) | 0.51  (-0.41 to 1.70) | 0.95  (0.08 to 1.86) | 0.65  (-0.25 to 1.71) | N/A | 0.42  (-0.19 to 1.15) |
| Maintaining clinical remission | Adalimumab | RZB-360 | 0.76  (-0.11 to 1.78) | 0.69  (0.12 to 1.26) | 0.76  (-0.11 to 1.77) | 0.63  (-0.29 to 1.82) | 1.06  (0.20 to 1.99) | 0.77  (-0.14 to 1.83) | N/A | 0.48  (-0.13 to 1.21) |
| Maintaining clinical remission | Adalimumab | Vedolizumab | 0.34  (-0.44 to 1.21) | 0.29  (-0.29 to 0.86) | 0.34  (-0.44 to 1.21) | 0.21  (-0.64 to 1.23) | 0.65  (-0.18 to 1.47) | 0.35  (-0.47 to 1.24) | N/A | 0.18  (-0.38 to 0.83) |
| Maintaining clinical remission | Adalimumab | Vedolizumab SC | 0.66  (-0.20 to 1.67) | 0.59  (0.04 to 1.14) | 0.66  (-0.20 to 1.66) | 0.53  (-0.37 to 1.70) | 0.96  (0.11 to 1.87) | 0.66  (-0.23 to 1.72) | N/A | 0.39  (-0.23 to 1.13) |
| Maintaining clinical remission | Adalimumab | placebo | 1.24  (0.78 to 1.81) | 1.16  (0.82 to 1.51) | 1.23  (0.78 to 1.80) | 1.11  (0.54 to 1.88) | 1.54  (1.01 to 2.12) | 1.24  (0.78 to 1.84) | N/A | 0.72  (0.41 to 1.13) |
| Maintaining clinical remission | Adalimumab+azathioprine | BI 695501 | 0.19  (-1.13 to 1.52) | 0.19  (-0.77 to 1.15) | 0.19  (-1.12 to 1.51) | 0.19  (-1.16 to 1.54) | N/A | N/A | N/A | 0.04  (-0.77 to 0.84) |
| Maintaining clinical remission | Adalimumab+azathioprine | Certolizumab Pegol | 0.33  (-0.76 to 1.55) | 0.26  (-0.49 to 1.00) | 0.32  (-0.76 to 1.53) | 0.19  (-0.94 to 1.57) | N/A | N/A | N/A | 0.15  (-0.57 to 1.00) |
| Maintaining clinical remission | Adalimumab+azathioprine | Ustekinumab | 0.06  (-0.93 to 1.20) | -0.02  (-0.72 to 0.67) | 0.06  (-0.92 to 1.18) | -0.01  (-1.03 to 1.21) | N/A | N/A | N/A | 0.06  (-0.56 to 0.84) |
| Maintaining clinical remission | Adalimumab+azathioprine | RZB-180 | 0.47  (-0.75 to 1.83) | 0.40  (-0.43 to 1.22) | 0.47  (-0.75 to 1.82) | 0.33  (-0.92 to 1.85) | N/A | N/A | N/A | 0.34  (-0.47 to 1.29) |
| Maintaining clinical remission | Adalimumab+azathioprine | RZB-360 | 0.59  (-0.64 to 1.95) | 0.52  (-0.32 to 1.35) | 0.59  (-0.64 to 1.94) | 0.45  (-0.81 to 1.97) | N/A | N/A | N/A | 0.40  (-0.41 to 1.35) |
| Maintaining clinical remission | Adalimumab+azathioprine | Vedolizumab | 0.17  (-1.00 to 1.42) | 0.11  (-0.73 to 0.94) | 0.17  (-1.00 to 1.41) | 0.03  (-1.18 to 1.43) | N/A | N/A | N/A | 0.10  (-0.67 to 0.98) |
| Maintaining clinical remission | Adalimumab+azathioprine | Vedolizumab SC | 0.49  (-0.73 to 1.85) | 0.41  (-0.41 to 1.23) | 0.49  (-0.73 to 1.83) | 0.35  (-0.90 to 1.86) | N/A | N/A | N/A | 0.31  (-0.51 to 1.27) |
| Maintaining clinical remission | Adalimumab+azathioprine | placebo | 1.06  (0.10 to 2.15) | 0.99  (0.29 to 1.68) | 1.06  (0.10 to 2.13) | 0.92  (-0.08 to 2.17) | N/A | N/A | N/A | 0.64  (0.04 to 1.38) |
| Maintaining clinical remission | BI 695501 | Certolizumab Pegol | 0.14  (-1.04 to 1.42) | 0.07  (-0.80 to 0.92) | 0.13  (-1.04 to 1.41) | 0.00  (-1.22 to 1.44) | 0.34  (-0.90 to 1.60) | 0.14  (-1.08 to 1.48) | N/A | 0.11  (-0.60 to 0.96) |
| Maintaining clinical remission | BI 695501 | Ustekinumab | -0.12  (-1.21 to 1.07) | -0.21  (-1.03 to 0.60) | -0.13  (-1.21 to 1.07) | -0.21  (-1.31 to 1.09) | 0.49  (-0.70 to 1.71) | -0.12  (-1.24 to 1.13) | N/A | 0.03  (-0.58 to 0.80) |
| Maintaining clinical remission | BI 695501 | RZB-180 | 0.28  (-1.01 to 1.69) | 0.21  (-0.73 to 1.13) | 0.28  (-1.02 to 1.69) | 0.14  (-1.19 to 1.71) | 0.58  (-0.68 to 1.87) | 0.28  (-1.06 to 1.76) | N/A | 0.31  (-0.50 to 1.25) |
| Maintaining clinical remission | BI 695501 | RZB-360 | 0.40  (-0.90 to 1.81) | 0.33  (-0.62 to 1.26) | 0.40  (-0.91 to 1.81) | 0.26  (-1.07 to 1.83) | 0.70  (-0.56 to 1.99) | 0.40  (-0.95 to 1.88) | N/A | 0.36  (-0.44 to 1.31) |
| Maintaining clinical remission | BI 695501 | Vedolizumab | -0.02  (-1.27 to 1.30) | -0.08  (-1.02 to 0.86) | -0.02  (-1.27 to 1.29) | -0.16  (-1.45 to 1.29) | 0.28  (-0.95 to 1.51) | -0.02  (-1.31 to 1.34) | N/A | 0.06  (-0.70 to 0.94) |
| Maintaining clinical remission | BI 695501 | Vedolizumab SC | 0.30  (-0.99 to 1.71) | 0.22  (-0.71 to 1.14) | 0.30  (-1.00 to 1.70) | 0.16  (-1.17 to 1.73) | 0.60  (-0.65 to 1.88) | 0.30  (-1.04 to 1.77) | N/A | 0.27  (-0.54 to 1.22) |
| Maintaining clinical remission | BI 695501 | placebo | 0.88  (-0.19 to 2.03) | 0.80  (-0.03 to 1.61) | 0.87  (-0.19 to 2.02) | 0.74  (-0.37 to 2.04) | 1.17  (0.12 to 2.25) | 0.87  (-0.22 to 2.08) | N/A | 0.60  (0.01 to 1.34) |
| Maintaining clinical remission | Certolizumab Pegol | Ustekinumab | -0.26  (-1.00 to 0.50) | -0.28  (-0.71 to 0.16) | -0.26  (-1.00 to 0.49) | -0.20  (-1.01 to 0.56) | 0.15  (-0.73 to 1.03) | -0.26  (-1.04 to 0.53) | N/A | -0.08  (-0.63 to 0.48) |
| Maintaining clinical remission | Certolizumab Pegol | RZB-180 | 0.14  (-0.81 to 1.10) | 0.14  (-0.38 to 0.66) | 0.14  (-0.80 to 1.10) | 0.15  (-0.85 to 1.13) | 0.25  (-0.73 to 1.22) | 0.14  (-0.86 to 1.16) | N/A | 0.20  (-0.50 to 0.90) |
| Maintaining clinical remission | Certolizumab Pegol | RZB-360 | 0.26  (-0.70 to 1.23) | 0.26  (-0.27 to 0.79) | 0.26  (-0.69 to 1.22) | 0.26  (-0.72 to 1.25) | 0.36  (-0.62 to 1.34) | 0.26  (-0.75 to 1.28) | N/A | 0.25  (-0.45 to 0.95) |
| Maintaining clinical remission | Certolizumab Pegol | Vedolizumab | -0.16  (-1.03 to 0.67) | -0.14  (-0.68 to 0.39) | -0.16  (-1.02 to 0.66) | -0.16  (-1.06 to 0.68) | -0.05  (-0.98 to 0.83) | -0.16  (-1.07 to 0.70) | N/A | -0.05  (-0.69 to 0.58) |
| Maintaining clinical remission | Certolizumab Pegol | Vedolizumab SC | 0.16  (-0.78 to 1.11) | 0.16  (-0.36 to 0.66) | 0.16  (-0.78 to 1.10) | 0.16  (-0.82 to 1.14) | 0.26  (-0.71 to 1.23) | 0.16  (-0.84 to 1.16) | N/A | 0.16  (-0.55 to 0.87) |
| Maintaining clinical remission | Certolizumab Pegol | placebo | 0.73  (0.20 to 1.28) | 0.73  (0.46 to 1.00) | 0.73  (0.20 to 1.27) | 0.74  (0.18 to 1.30) | 0.84  (0.16 to 1.51) | 0.73  (0.16 to 1.31) | N/A | 0.49  (0.08 to 0.91) |
| Maintaining clinical remission | Ustekinumab | RZB-180 | 0.41  (-0.55 to 1.35) | 0.42  (-0.14 to 0.97) | 0.40  (-0.54 to 1.34) | 0.35  (-0.62 to 1.36) | 0.10  (-0.80 to 0.99) | 0.40  (-0.60 to 1.39) | N/A | 0.28  (-0.41 to 0.95) |
| Maintaining clinical remission | Ustekinumab | RZB-360 | 0.53  (-0.43 to 1.47) | 0.53  (-0.03 to 1.10) | 0.52  (-0.42 to 1.46) | 0.46  (-0.50 to 1.48) | 0.21  (-0.70 to 1.11) | 0.52  (-0.48 to 1.51) | N/A | 0.34  (-0.36 to 1.00) |
| Maintaining clinical remission | Ustekinumab | Vedolizumab | 0.10  (-0.76 to 0.92) | 0.13  (-0.44 to 0.70) | 0.10  (-0.76 to 0.92) | 0.04  (-0.83 to 0.90) | -0.20  (-1.06 to 0.60) | 0.10  (-0.80 to 0.94) | N/A | 0.03  (-0.59 to 0.63) |
| Maintaining clinical remission | Ustekinumab | Vedolizumab SC | 0.43  (-0.52 to 1.35) | 0.43  (-0.12 to 0.98) | 0.43  (-0.52 to 1.35) | 0.37  (-0.59 to 1.37) | 0.11  (-0.78 to 1.00) | 0.42  (-0.57 to 1.40) | N/A | 0.25  (-0.45 to 0.92) |
| Maintaining clinical remission | Ustekinumab | placebo | 1.00  (0.47 to 1.51) | 1.01  (0.67 to 1.35) | 1.00  (0.47 to 1.51) | 0.94  (0.41 to 1.51) | 0.69  (0.12 to 1.26) | 0.99  (0.45 to 1.53) | N/A | 0.57  (0.20 to 0.94) |
| Maintaining clinical remission | RZB-180 | RZB-360 | 0.12  (-0.68 to 0.91) | 0.12  (-0.34 to 0.58) | 0.12  (-0.68 to 0.91) | 0.12  (-0.70 to 0.94) | 0.12  (-0.59 to 0.83) | 0.12  (-0.72 to 0.96) | N/A | 0.06  (-0.51 to 0.62) |
| Maintaining clinical remission | RZB-180 | Vedolizumab | -0.30  (-1.35 to 0.70) | -0.28  (-0.92 to 0.35) | -0.30  (-1.35 to 0.70) | -0.30  (-1.39 to 0.73) | -0.30  (-1.24 to 0.60) | -0.30  (-1.40 to 0.74) | N/A | -0.25  (-0.99 to 0.49) |
| Maintaining clinical remission | RZB-180 | Vedolizumab SC | 0.02  (-1.09 to 1.13) | 0.02  (-0.60 to 0.63) | 0.02  (-1.09 to 1.12) | 0.02  (-1.13 to 1.17) | 0.02  (-0.97 to 1.00) | 0.02  (-1.16 to 1.19) | N/A | -0.03  (-0.84 to 0.78) |
| Maintaining clinical remission | RZB-180 | placebo | 0.59  (-0.20 to 1.38) | 0.59  (0.15 to 1.04) | 0.59  (-0.20 to 1.37) | 0.59  (-0.22 to 1.41) | 0.59  (-0.11 to 1.29) | 0.59  (-0.24 to 1.43) | N/A | 0.30  (-0.27 to 0.87) |
| Maintaining clinical remission | RZB-360 | Vedolizumab | -0.42  (-1.48 to 0.59) | -0.40  (-1.05 to 0.24) | -0.42  (-1.47 to 0.59) | -0.42  (-1.51 to 0.61) | -0.42  (-1.37 to 0.49) | -0.42  (-1.53 to 0.63) | N/A | -0.30  (-1.05 to 0.44) |
| Maintaining clinical remission | RZB-360 | Vedolizumab SC | -0.10  (-1.22 to 1.02) | -0.10  (-0.73 to 0.53) | -0.10  (-1.21 to 1.01) | -0.10  (-1.25 to 1.05) | -0.10  (-1.09 to 0.89) | -0.11  (-1.28 to 1.07) | N/A | -0.09  (-0.90 to 0.72) |
| Maintaining clinical remission | RZB-360 | placebo | 0.47  (-0.32 to 1.27) | 0.47  (0.02 to 0.93) | 0.47  (-0.32 to 1.26) | 0.47  (-0.35 to 1.29) | 0.47  (-0.23 to 1.18) | 0.47  (-0.36 to 1.31) | N/A | 0.24  (-0.33 to 0.81) |
| Maintaining clinical remission | Vedolizumab | Vedolizumab SC | 0.32  (-0.68 to 1.36) | 0.30  (-0.33 to 0.93) | 0.32  (-0.68 to 1.36) | 0.32  (-0.69 to 1.40) | 0.32  (-0.58 to 1.26) | 0.32  (-0.72 to 1.41) | N/A | 0.21  (-0.53 to 0.96) |
| Maintaining clinical remission | Vedolizumab | placebo | 0.89  (0.26 to 1.57) | 0.87  (0.42 to 1.34) | 0.89  (0.26 to 1.57) | 0.89  (0.25 to 1.59) | 0.89  (0.31 to 1.52) | 0.89  (0.24 to 1.60) | N/A | 0.54  (0.07 to 1.03) |
| Maintaining clinical remission | Vedolizumab SC | placebo | 0.57  (-0.21 to 1.36) | 0.57  (0.15 to 1.01) | 0.57  (-0.20 to 1.35) | 0.57  (-0.23 to 1.38) | 0.57  (-0.12 to 1.27) | 0.57  (-0.25 to 1.40) | N/A | 0.33  (-0.24 to 0.91) |
| Maintaining CDAI-70 | IFX-5 | IFX-10 | -0.30  (-1.51 to 1.01) | -0.32  (-0.84 to 0.20) | -0.30  (-1.50 to 0.98) | N/A | -0.29  (-1.48 to 0.98) | -0.38  (-1.97 to 1.20) | N/A | -0.11  (-1.36 to 1.20) |
| Maintaining CDAI-70 | IFX-5 | Infliximab+azathioprine | -0.26  (-1.63 to 1.14) | -0.26  (-0.95 to 0.43) | -0.26  (-1.64 to 1.12) | N/A | N/A | -0.26  (-1.89 to 1.37) | N/A | -0.05  (-1.39 to 1.28) |
| Maintaining CDAI-70 | IFX-5 | CT-P13 | -0.08  (-1.44 to 1.29) | -0.07  (-0.70 to 0.55) | -0.07  (-1.41 to 1.28) | N/A | -0.08  (-1.41 to 1.26) | -0.07  (-1.69 to 1.52) | N/A | -0.02  (-1.36 to 1.31) |
| Maintaining CDAI-70 | IFX-5 | Adalimumab | -0.22  (-1.77 to 1.25) | -0.20  (-0.91 to 0.53) | -0.22  (-1.74 to 1.22) | N/A | -0.22  (-1.73 to 1.23) | -0.08  (-1.91 to 1.74) | N/A | -0.01  (-1.54 to 1.43) |
| Maintaining CDAI-70 | IFX-5 | Adalimumab+azathioprine | 0.02  (-2.07 to 2.01) | 0.04  (-0.90 to 0.99) | 0.02  (-2.01 to 1.98) | N/A | N/A | N/A | N/A | 0.09  (-1.98 to 2.05) |
| Maintaining CDAI-70 | IFX-5 | BI 695501 | -0.16  (-2.30 to 1.90) | -0.12  (-1.25 to 1.00) | -0.15  (-2.27 to 1.89) | N/A | N/A | -0.01  (-2.48 to 2.48) | N/A | 0.01  (-2.04 to 1.97) |
| Maintaining CDAI-70 | IFX-5 | Certolizumab Pegol | 0.47  (-1.38 to 2.26) | 0.49  (-0.18 to 1.17) | 0.47  (-1.33 to 2.23) | N/A | N/A | 0.60  (-1.59 to 2.85) | N/A | 0.31  (-1.55 to 2.19) |
| Maintaining CDAI-70 | IFX-5 | placebo | 1.09  (-0.22 to 2.36) | 1.11  (0.52 to 1.72) | 1.09  (-0.20 to 2.33) | N/A | 1.09  (-0.17 to 2.34) | 1.22  (-0.35 to 2.83) | N/A | 0.70  (-0.59 to 2.00) |
| Maintaining CDAI-70 | IFX-10 | Infliximab+azathioprine | 0.04  (-1.86 to 1.88) | 0.06  (-0.80 to 0.92) | 0.04  (-1.85 to 1.85) | N/A | N/A | 0.13  (-2.15 to 2.40) | N/A | 0.05  (-1.83 to 1.88) |
| Maintaining CDAI-70 | IFX-10 | CT-P13 | 0.21  (-1.69 to 2.05) | 0.24  (-0.56 to 1.06) | 0.22  (-1.64 to 2.02) | N/A | 0.21  (-1.63 to 1.98) | 0.31  (-1.97 to 2.56) | N/A | 0.09  (-1.79 to 1.92) |
| Maintaining CDAI-70 | IFX-10 | Adalimumab | 0.08  (-1.29 to 1.28) | 0.12  (-0.53 to 0.79) | 0.08  (-1.25 to 1.27) | N/A | 0.06  (-1.25 to 1.27) | 0.30  (-1.55 to 2.13) | N/A | 0.09  (-1.21 to 1.28) |
| Maintaining CDAI-70 | IFX-10 | Adalimumab+azathioprine | 0.32  (-1.64 to 2.11) | 0.36  (-0.54 to 1.27) | 0.32  (-1.58 to 2.09) | N/A | N/A | N/A | N/A | 0.19  (-1.72 to 1.95) |
| Maintaining CDAI-70 | IFX-10 | BI 695501 | 0.13  (-1.88 to 2.02) | 0.19  (-0.90 to 1.28) | 0.14  (-1.85 to 2.00) | N/A | 0.15  (-1.82 to 2.00) | 0.37  (-2.13 to 2.86) | N/A | 0.12  (-1.79 to 1.86) |
| Maintaining CDAI-70 | IFX-10 | Certolizumab Pegol | 0.76  (-0.93 to 2.33) | 0.80  (0.19 to 1.43) | 0.76  (-0.91 to 2.30) | N/A | N/A | 0.99  (-1.24 to 3.23) | N/A | 0.41  (-1.28 to 2.07) |
| Maintaining CDAI-70 | IFX-10 | placebo | 1.38  (0.32 to 2.35) | 1.42  (0.91 to 1.96) | 1.39  (0.34 to 2.34) | N/A | 1.38  (0.34 to 2.34) | 1.61  (0.01 to 3.23) | N/A | 0.80  (-0.21 to 1.80) |
| Maintaining CDAI-70 | Infliximab+azathioprine | CT-P13 | 0.17  (-1.76 to 2.11) | 0.18  (-0.74 to 1.12) | 0.18  (-1.74 to 2.12) | N/A | N/A | 0.18  (-2.12 to 2.47) | N/A | 0.03  (-1.86 to 1.93) |
| Maintaining CDAI-70 | Infliximab+azathioprine | Adalimumab | 0.04  (-2.06 to 2.03) | 0.06  (-0.92 to 1.06) | 0.04  (-2.01 to 2.03) | N/A | N/A | 0.17  (-2.27 to 2.62) | N/A | 0.05  (-2.00 to 1.99) |
| Maintaining CDAI-70 | Infliximab+azathioprine | Adalimumab+azathioprine | 0.28  (-2.24 to 2.69) | 0.30  (-0.87 to 1.47) | 0.27  (-2.16 to 2.67) | N/A | N/A | N/A | N/A | 0.14  (-2.33 to 2.51) |
| Maintaining CDAI-70 | Infliximab+azathioprine | BI 695501 | 0.10  (-2.47 to 2.57) | 0.13  (-1.19 to 1.45) | 0.10  (-2.42 to 2.55) | N/A | N/A | 0.25  (-2.71 to 3.23) | N/A | 0.06  (-2.40 to 2.42) |
| Maintaining CDAI-70 | Infliximab+azathioprine | Certolizumab Pegol | 0.72  (-1.60 to 2.99) | 0.74  (-0.22 to 1.72) | 0.72  (-1.53 to 2.95) | N/A | N/A | 0.85  (-1.87 to 3.64) | N/A | 0.36  (-1.93 to 2.66) |
| Maintaining CDAI-70 | Infliximab+azathioprine | placebo | 1.34  (-0.57 to 3.22) | 1.37  (0.46 to 2.28) | 1.35  (-0.54 to 3.19) | N/A | N/A | 1.48  (-0.78 to 3.79) | N/A | 0.75  (-1.10 to 2.62) |
| Maintaining CDAI-70 | CT-P13 | Adalimumab | -0.14  (-2.24 to 1.86) | -0.12  (-1.08 to 0.84) | -0.14  (-2.19 to 1.82) | N/A | -0.14  (-2.14 to 1.82) | 0.00  (-2.42 to 2.43) | N/A | 0.02  (-2.04 to 1.96) |
| Maintaining CDAI-70 | CT-P13 | Adalimumab+azathioprine | 0.10  (-2.41 to 2.51) | 0.11  (-1.03 to 1.26) | 0.09  (-2.36 to 2.48) | N/A | N/A | N/A | N/A | 0.11  (-2.35 to 2.45) |
| Maintaining CDAI-70 | CT-P13 | BI 695501 | -0.08  (-2.64 to 2.38) | -0.05  (-1.35 to 1.23) | -0.07  (-2.60 to 2.36) | N/A | -0.06  (-2.53 to 2.36) | 0.07  (-2.86 to 3.04) | N/A | 0.03  (-2.42 to 2.38) |
| Maintaining CDAI-70 | CT-P13 | Certolizumab Pegol | 0.55  (-1.78 to 2.80) | 0.56  (-0.36 to 1.49) | 0.54  (-1.72 to 2.74) | N/A | N/A | 0.68  (-2.03 to 3.46) | N/A | 0.33  (-1.96 to 2.64) |
| Maintaining CDAI-70 | CT-P13 | placebo | 1.17  (-0.75 to 3.03) | 1.18  (0.32 to 2.05) | 1.17  (-0.72 to 2.99) | N/A | 1.18  (-0.67 to 2.99) | 1.30  (-0.92 to 3.59) | N/A | 0.72  (-1.14 to 2.59) |
| Maintaining CDAI-70 | Adalimumab | Adalimumab+azathioprine | 0.24  (-1.13 to 1.59) | 0.24  (-0.38 to 0.86) | 0.24  (-1.11 to 1.58) | N/A | N/A | N/A | N/A | 0.09  (-1.26 to 1.45) |
| Maintaining CDAI-70 | Adalimumab | BI 695501 | 0.06  (-1.41 to 1.56) | 0.07  (-0.80 to 0.93) | 0.07  (-1.38 to 1.52) | N/A | 0.08  (-1.37 to 1.52) | 0.07  (-1.62 to 1.77) | N/A | 0.02  (-1.33 to 1.36) |
| Maintaining CDAI-70 | Adalimumab | Certolizumab Pegol | 0.68  (-0.81 to 2.20) | 0.68  (0.17 to 1.20) | 0.68  (-0.78 to 2.18) | N/A | N/A | 0.69  (-1.07 to 2.50) | N/A | 0.31  (-1.16 to 1.90) |
| Maintaining CDAI-70 | Adalimumab | placebo | 1.30  (0.55 to 2.11) | 1.30  (0.91 to 1.71) | 1.31  (0.55 to 2.10) | N/A | 1.32  (0.56 to 2.10) | 1.31  (0.42 to 2.23) | N/A | 0.71  (0.03 to 1.49) |
| Maintaining CDAI-70 | Adalimumab+azathioprine | BI 695501 | -0.18  (-2.18 to 1.84) | -0.16  (-1.23 to 0.89) | -0.17  (-2.16 to 1.80) | N/A | N/A | N/A | N/A | -0.08  (-1.97 to 1.84) |
| Maintaining CDAI-70 | Adalimumab+azathioprine | Certolizumab Pegol | 0.44  (-1.58 to 2.49) | 0.44  (-0.36 to 1.25) | 0.44  (-1.53 to 2.45) | N/A | N/A | N/A | N/A | 0.22  (-1.76 to 2.31) |
| Maintaining CDAI-70 | Adalimumab+azathioprine | placebo | 1.07  (-0.48 to 2.67) | 1.07  (0.33 to 1.80) | 1.07  (-0.46 to 2.63) | N/A | N/A | N/A | N/A | 0.61  (-0.87 to 2.22) |
| Maintaining CDAI-70 | BI 695501 | Certolizumab Pegol | 0.62  (-1.45 to 2.74) | 0.61  (-0.39 to 1.62) | 0.61  (-1.45 to 2.71) | N/A | N/A | 0.61  (-1.81 to 3.08) | N/A | 0.30  (-1.69 to 2.40) |
| Maintaining CDAI-70 | BI 695501 | placebo | 1.25  (-0.40 to 2.93) | 1.23  (0.28 to 2.19) | 1.24  (-0.39 to 2.92) | N/A | 1.24  (-0.39 to 2.89) | 1.24  (-0.68 to 3.17) | N/A | 0.69  (-0.78 to 2.27) |
| Maintaining CDAI-70 | Certolizumab Pegol | placebo | 0.62  (-0.66 to 1.91) | 0.62  (0.30 to 0.95) | 0.63  (-0.64 to 1.89) | N/A | N/A | 0.62  (-0.94 to 2.16) | N/A | 0.39  (-0.95 to 1.75) |
| Maintaining CDAI-100 | Adalimumab | BI 695501 | 0.34  (-0.96 to 1.65) | 0.35  (-0.49 to 1.19) | 0.34  (-0.95 to 1.65) | 0.34  (-0.93 to 1.63) | 0.33  (-0.93 to 1.60) | N/A | N/A | 0.07  (-0.53 to 0.67) |
| Maintaining CDAI-100 | Adalimumab | Certolizumab Pegol | 0.36  (-0.58 to 1.40) | 0.31  (-0.11 to 0.73) | 0.36  (-0.58 to 1.40) | 0.26  (-0.71 to 1.43) | 0.22  (-0.99 to 1.52) | N/A | N/A | 0.15  (-0.36 to 0.76) |
| Maintaining CDAI-100 | Adalimumab | Ustekinumab | 0.07  (-0.66 to 0.89) | 0.00  (-0.35 to 0.35) | 0.06  (-0.66 to 0.88) | 0.01  (-0.73 to 0.89) | 0.49  (-0.55 to 1.55) | N/A | N/A | 0.09  (-0.29 to 0.56) |
| Maintaining CDAI-100 | Adalimumab | RZB-180 | 0.35  (-0.87 to 1.67) | 0.27  (-0.29 to 0.83) | 0.34  (-0.86 to 1.69) | 0.25  (-0.97 to 1.70) | 0.53  (-0.70 to 1.86) | N/A | N/A | 0.26  (-0.39 to 1.02) |
| Maintaining CDAI-100 | Adalimumab | RZB-360 | 0.57  (-0.63 to 1.91) | 0.50  (-0.07 to 1.06) | 0.57  (-0.65 to 1.90) | 0.47  (-0.75 to 1.93) | 0.76  (-0.47 to 2.10) | N/A | N/A | 0.34  (-0.31 to 1.11) |
| Maintaining CDAI-100 | Adalimumab | Vedolizumab | 0.34  (-0.74 to 1.32) | 0.38  (-0.17 to 0.92) | 0.33  (-0.74 to 1.31) | 0.48  (-0.57 to 1.76) | 0.56  (-0.60 to 1.49) | N/A | N/A | 0.17  (-0.41 to 0.75) |
| Maintaining CDAI-100 | Adalimumab | Vedolizumab SC | 0.84  (-0.36 to 2.18) | 0.76  (0.22 to 1.29) | 0.83  (-0.37 to 2.15) | 0.74  (-0.46 to 2.18) | 1.03  (-0.19 to 2.32) | N/A | N/A | 0.44  (-0.22 to 1.20) |
| Maintaining CDAI-100 | Adalimumab | placebo | 1.13  (0.55 to 1.82) | 1.05  (0.72 to 1.38) | 1.13  (0.55 to 1.81) | 1.04  (0.37 to 1.89) | 1.32  (0.67 to 2.03) | N/A | N/A | 0.59  (0.27 to 0.99) |
| Maintaining CDAI-100 | BI 695501 | Certolizumab Pegol | 0.03  (-1.56 to 1.70) | -0.03  (-0.97 to 0.91) | 0.02  (-1.57 to 1.68) | -0.08  (-1.67 to 1.67) | -0.11  (-1.85 to 1.69) | N/A | N/A | 0.07  (-0.68 to 0.96) |
| Maintaining CDAI-100 | BI 695501 | Ustekinumab | -0.27  (-1.76 to 1.28) | -0.34  (-1.25 to 0.56) | -0.28  (-1.76 to 1.26) | -0.33  (-1.80 to 1.24) | 0.16  (-1.48 to 1.79) | N/A | N/A | 0.01  (-0.67 to 0.80) |
| Maintaining CDAI-100 | BI 695501 | RZB-180 | 0.01  (-1.77 to 1.87) | -0.08  (-1.09 to 0.93) | 0.00  (-1.76 to 1.87) | -0.09  (-1.85 to 1.85) | 0.20  (-1.55 to 2.03) | N/A | N/A | 0.18  (-0.68 to 1.18) |
| Maintaining CDAI-100 | BI 695501 | RZB-360 | 0.23  (-1.53 to 2.11) | 0.15  (-0.86 to 1.16) | 0.23  (-1.54 to 2.09) | 0.14  (-1.62 to 2.08) | 0.43  (-1.32 to 2.25) | N/A | N/A | 0.27  (-0.60 to 1.26) |
| Maintaining CDAI-100 | BI 695501 | Vedolizumab | 0.01  (-1.72 to 1.60) | 0.03  (-0.97 to 1.03) | -0.01  (-1.72 to 1.59) | 0.14  (-1.51 to 1.96) | 0.22  (-1.53 to 1.76) | N/A | N/A | 0.10  (-0.73 to 0.93) |
| Maintaining CDAI-100 | BI 695501 | Vedolizumab SC | 0.50  (-1.26 to 2.37) | 0.41  (-0.58 to 1.40) | 0.50  (-1.26 to 2.34) | 0.40  (-1.34 to 2.33) | 0.69  (-1.06 to 2.50) | N/A | N/A | 0.36  (-0.51 to 1.35) |
| Maintaining CDAI-100 | BI 695501 | placebo | 0.79  (-0.61 to 2.30) | 0.70  (-0.20 to 1.60) | 0.78  (-0.61 to 2.27) | 0.69  (-0.72 to 2.26) | 0.98  (-0.43 to 2.45) | N/A | N/A | 0.51  (-0.13 to 1.26) |
| Maintaining CDAI-100 | Certolizumab Pegol | Ustekinumab | -0.29  (-1.34 to 0.73) | -0.31  (-0.74 to 0.11) | -0.29  (-1.34 to 0.73) | -0.26  (-1.32 to 0.76) | 0.27  (-1.09 to 1.57) | N/A | N/A | -0.06  (-0.64 to 0.50) |
| Maintaining CDAI-100 | Certolizumab Pegol | RZB-180 | -0.01  (-1.37 to 1.33) | -0.04  (-0.57 to 0.47) | -0.01  (-1.35 to 1.35) | -0.02  (-1.34 to 1.34) | 0.31  (-1.20 to 1.83) | N/A | N/A | 0.11  (-0.64 to 0.85) |
| Maintaining CDAI-100 | Certolizumab Pegol | RZB-360 | 0.21  (-1.14 to 1.57) | 0.18  (-0.34 to 0.71) | 0.22  (-1.13 to 1.57) | 0.21  (-1.11 to 1.58) | 0.54  (-0.98 to 2.07) | N/A | N/A | 0.20  (-0.56 to 0.94) |
| Maintaining CDAI-100 | Certolizumab Pegol | Vedolizumab | -0.01  (-1.27 to 1.00) | 0.06  (-0.44 to 0.56) | -0.02  (-1.26 to 0.99) | 0.21  (-0.91 to 1.41) | 0.35  (-1.17 to 1.49) | N/A | N/A | 0.02  (-0.67 to 0.61) |
| Maintaining CDAI-100 | Certolizumab Pegol | Vedolizumab SC | 0.48  (-0.86 to 1.84) | 0.45  (-0.04 to 0.93) | 0.47  (-0.85 to 1.82) | 0.47  (-0.83 to 1.83) | 0.81  (-0.71 to 2.30) | N/A | N/A | 0.29  (-0.47 to 1.03) |
| Maintaining CDAI-100 | Certolizumab Pegol | placebo | 0.77  (0.01 to 1.55) | 0.74  (0.48 to 0.99) | 0.77  (0.01 to 1.54) | 0.77  (0.02 to 1.55) | 1.09  (0.03 to 2.16) | N/A | N/A | 0.43  (0.00 to 0.86) |
| Maintaining CDAI-100 | Ustekinumab | RZB-180 | 0.28  (-1.02 to 1.60) | 0.27  (-0.30 to 0.83) | 0.28  (-1.02 to 1.61) | 0.24  (-1.03 to 1.60) | 0.04  (-1.25 to 1.43) | N/A | N/A | 0.17  (-0.53 to 0.90) |
| Maintaining CDAI-100 | Ustekinumab | RZB-360 | 0.50  (-0.79 to 1.84) | 0.49  (-0.07 to 1.06) | 0.51  (-0.79 to 1.84) | 0.47  (-0.81 to 1.83) | 0.27  (-1.04 to 1.66) | N/A | N/A | 0.26  (-0.45 to 0.99) |
| Maintaining CDAI-100 | Ustekinumab | Vedolizumab | 0.28  (-0.91 to 1.26) | 0.37  (-0.18 to 0.92) | 0.27  (-0.92 to 1.25) | 0.47  (-0.60 to 1.64) | 0.07  (-1.20 to 1.06) | N/A | N/A | 0.08  (-0.56 to 0.65) |
| Maintaining CDAI-100 | Ustekinumab | Vedolizumab SC | 0.77  (-0.51 to 2.10) | 0.76  (0.22 to 1.29) | 0.77  (-0.52 to 2.09) | 0.73  (-0.53 to 2.07) | 0.54  (-0.77 to 1.91) | N/A | N/A | 0.35  (-0.37 to 1.08) |
| Maintaining CDAI-100 | Ustekinumab | placebo | 1.06  (0.38 to 1.78) | 1.05  (0.71 to 1.39) | 1.06  (0.38 to 1.78) | 1.02  (0.35 to 1.77) | 0.82  (0.06 to 1.65) | N/A | N/A | 0.50  (0.13 to 0.88) |
| Maintaining CDAI-100 | RZB-180 | RZB-360 | 0.23  (-0.89 to 1.35) | 0.23  (-0.25 to 0.70) | 0.23  (-0.90 to 1.34) | 0.23  (-0.89 to 1.34) | 0.23  (-0.86 to 1.31) | N/A | N/A | 0.08  (-0.52 to 0.68) |
| Maintaining CDAI-100 | RZB-180 | Vedolizumab | 0.00  (-1.51 to 1.26) | 0.11  (-0.52 to 0.73) | 0.00  (-1.52 to 1.25) | 0.23  (-1.17 to 1.65) | 0.03  (-1.51 to 1.19) | N/A | N/A | -0.09  (-0.91 to 0.63) |
| Maintaining CDAI-100 | RZB-180 | Vedolizumab SC | 0.49  (-1.06 to 2.06) | 0.49  (-0.13 to 1.11) | 0.49  (-1.08 to 2.04) | 0.49  (-1.07 to 2.04) | 0.49  (-1.04 to 2.00) | N/A | N/A | 0.18  (-0.69 to 1.04) |
| Maintaining CDAI-100 | RZB-180 | placebo | 0.78  (-0.32 to 1.90) | 0.78  (0.33 to 1.24) | 0.78  (-0.34 to 1.90) | 0.78  (-0.33 to 1.89) | 0.78  (-0.31 to 1.85) | N/A | N/A | 0.32  (-0.28 to 0.93) |
| Maintaining CDAI-100 | RZB-360 | Vedolizumab | -0.22  (-1.75 to 1.03) | -0.12  (-0.74 to 0.51) | -0.23  (-1.74 to 1.02) | 0.00  (-1.40 to 1.42) | -0.20  (-1.74 to 0.96) | N/A | N/A | -0.17  (-1.00 to 0.55) |
| Maintaining CDAI-100 | RZB-360 | Vedolizumab SC | 0.27  (-1.30 to 1.84) | 0.26  (-0.35 to 0.88) | 0.26  (-1.30 to 1.83) | 0.26  (-1.29 to 1.82) | 0.27  (-1.28 to 1.78) | N/A | N/A | 0.09  (-0.78 to 0.96) |
| Maintaining CDAI-100 | RZB-360 | placebo | 0.56  (-0.56 to 1.67) | 0.55  (0.10 to 1.01) | 0.55  (-0.55 to 1.67) | 0.55  (-0.55 to 1.66) | 0.56  (-0.54 to 1.62) | N/A | N/A | 0.24  (-0.37 to 0.85) |
| Maintaining CDAI-100 | Vedolizumab | Vedolizumab SC | 0.49  (-0.75 to 2.01) | 0.38  (-0.21 to 0.98) | 0.49  (-0.75 to 2.00) | 0.27  (-1.16 to 1.65) | 0.46  (-0.69 to 1.98) | N/A | N/A | 0.26  (-0.46 to 1.08) |
| Maintaining CDAI-100 | Vedolizumab | placebo | 0.79  (0.06 to 1.73) | 0.67  (0.25 to 1.10) | 0.79  (0.07 to 1.73) | 0.55  (-0.34 to 1.42) | 0.75  (0.10 to 1.72) | N/A | N/A | 0.42  (-0.01 to 0.93) |
| Maintaining CDAI-100 | Vedolizumab SC | placebo | 0.29  (-0.81 to 1.39) | 0.29  (-0.13 to 0.71) | 0.29  (-0.80 to 1.39) | 0.29  (-0.80 to 1.38) | 0.29  (-0.77 to 1.37) | N/A | N/A | 0.15  (-0.46 to 0.76) |
| Risk of AEs in maintenance therapy | IFX-10 | Adalimumab | -1.11  (-4.56 to 1.71) | -1.04  (-4.43 to 1.58) | -1.08  (-4.61 to 1.70) | -0.84  (-4.14 to 1.89) | -1.31  (-4.80 to 1.62) | N/A | N/A | -0.06  (-0.24 to 0.12) |
| Risk of AEs in maintenance therapy | IFX-10 | BI 695501 | -1.02  (-4.62 to 2.00) | -0.95  (-4.39 to 1.75) | -1.00  (-4.69 to 2.01) | -0.75  (-4.18 to 2.11) | -1.23  (-4.85 to 1.94) | N/A | N/A | 0.01  (-0.42 to 0.43) |
| Risk of AEs in maintenance therapy | IFX-10 | Certolizumab Pegol | -0.92  (-4.37 to 1.92) | -0.87  (-4.26 to 1.74) | -0.87  (-4.40 to 1.94) | -0.83  (-4.13 to 1.91) | -0.73  (-4.27 to 2.30) | N/A | N/A | -0.03  (-0.23 to 0.16) |
| Risk of AEs in maintenance therapy | IFX-10 | Ustekinumab | -0.86  (-4.30 to 2.00) | -0.86  (-4.25 to 1.77) | -0.81  (-4.34 to 1.98) | -0.70  (-4.01 to 2.02) | -0.51  (-4.04 to 2.48) | N/A | N/A | -0.02  (-0.21 to 0.18) |
| Risk of AEs in maintenance therapy | IFX-10 | RZB-180 | -0.80  (-4.32 to 2.10) | -0.76  (-4.16 to 1.87) | -0.77  (-4.41 to 2.12) | -0.73  (-4.07 to 2.05) | -0.76  (-4.30 to 2.29) | N/A | N/A | 0.00  (-0.24 to 0.23) |
| Risk of AEs in maintenance therapy | IFX-10 | RZB-360 | -0.83  (-4.35 to 2.07) | -0.79  (-4.19 to 1.85) | -0.79  (-4.41 to 2.09) | -0.76  (-4.09 to 2.05) | -0.79  (-4.33 to 2.25) | N/A | N/A | -0.01  (-0.25 to 0.22) |
| Risk of AEs in maintenance therapy | IFX-10 | Vedolizumab | -1.05  (-4.53 to 1.86) | -1.08  (-4.50 to 1.60) | -1.01  (-4.59 to 1.86) | -0.98  (-4.36 to 1.79) | -0.96  (-4.49 to 2.04) | N/A | N/A | -0.05  (-0.25 to 0.15) |
| Risk of AEs in maintenance therapy | IFX-10 | Vedolizumab SC | -0.75  (-4.26 to 2.17) | -0.71  (-4.10 to 1.93) | -0.72  (-4.37 to 2.18) | -0.67  (-4.00 to 2.14) | -0.70  (-4.22 to 2.35) | N/A | N/A | 0.01  (-0.22 to 0.23) |
| Risk of AEs in maintenance therapy | IFX-10 | placebo | -0.90  (-4.28 to 1.84) | -0.85  (-4.22 to 1.74) | -0.85  (-4.34 to 1.86) | -0.81  (-4.08 to 1.88) | -0.84  (-4.23 to 1.97) | N/A | N/A | -0.03  (-0.20 to 0.13) |
| Risk of AEs in maintenance therapy | Adalimumab | BI 695501 | 0.09  (-1.02 to 1.20) | 0.09  (-0.56 to 0.75) | 0.09  (-1.02 to 1.21) | 0.09  (-0.80 to 0.99) | 0.09  (-1.15 to 1.35) | 0.10  (-1.03 to 1.21) | N/A | 0.07  (-0.32 to 0.45) |
| Risk of AEs in maintenance therapy | Adalimumab | Certolizumab Pegol | 0.18  (-0.73 to 1.21) | 0.16  (-0.31 to 0.63) | 0.19  (-0.73 to 1.20) | 0.02  (-0.80 to 0.76) | 0.58  (-0.81 to 2.03) | 0.19  (-0.74 to 1.20) | N/A | 0.03  (-0.11 to 0.16) |
| Risk of AEs in maintenance therapy | Adalimumab | Ustekinumab | 0.24  (-0.43 to 1.09) | 0.17  (-0.22 to 0.57) | 0.25  (-0.44 to 1.09) | 0.14  (-0.46 to 0.76) | 0.80  (-0.38 to 2.08) | 0.25  (-0.44 to 1.10) | N/A | 0.04  (-0.06 to 0.15) |
| Risk of AEs in maintenance therapy | Adalimumab | RZB-180 | 0.30  (-0.87 to 1.58) | 0.28  (-0.33 to 0.87) | 0.30  (-0.88 to 1.56) | 0.12  (-0.88 to 1.06) | 0.56  (-0.84 to 2.02) | 0.29  (-0.88 to 1.57) | N/A | 0.06  (-0.13 to 0.24) |
| Risk of AEs in maintenance therapy | Adalimumab | RZB-360 | 0.27  (-0.90 to 1.55) | 0.25  (-0.35 to 0.85) | 0.28  (-0.91 to 1.53) | 0.09  (-0.90 to 1.03) | 0.53  (-0.87 to 1.99) | 0.27  (-0.90 to 1.54) | N/A | 0.05  (-0.14 to 0.24) |
| Risk of AEs in maintenance therapy | Adalimumab | Vedolizumab | 0.04  (-0.95 to 1.29) | -0.04  (-0.74 to 0.66) | 0.05  (-0.95 to 1.28) | -0.15  (-1.10 to 0.83) | 0.32  (-0.83 to 1.74) | 0.04  (-0.97 to 1.30) | N/A | 0.01  (-0.13 to 0.15) |
| Risk of AEs in maintenance therapy | Adalimumab | Vedolizumab SC | 0.34  (-0.82 to 1.64) | 0.33  (-0.28 to 0.94) | 0.35  (-0.83 to 1.62) | 0.17  (-0.84 to 1.12) | 0.61  (-0.79 to 2.08) | 0.35  (-0.85 to 1.63) | N/A | 0.07  (-0.12 to 0.24) |
| Risk of AEs in maintenance therapy | Adalimumab | placebo | 0.20  (-0.40 to 0.90) | 0.18  (-0.20 to 0.56) | 0.21  (-0.41 to 0.90) | 0.03  (-0.59 to 0.58) | 0.46  (-0.34 to 1.32) | 0.21  (-0.41 to 0.89) | N/A | 0.03  (-0.05 to 0.11) |
| Risk of AEs in maintenance therapy | BI 695501 | Certolizumab Pegol | 0.09  (-1.33 to 1.62) | 0.07  (-0.74 to 0.88) | 0.11  (-1.33 to 1.61) | -0.07  (-1.30 to 1.08) | 0.49  (-1.37 to 2.40) | 0.09  (-1.34 to 1.62) | N/A | -0.04  (-0.44 to 0.37) |
| Risk of AEs in maintenance therapy | BI 695501 | Ustekinumab | 0.15  (-1.12 to 1.58) | 0.08  (-0.69 to 0.85) | 0.16  (-1.13 to 1.58) | 0.05  (-1.04 to 1.14) | 0.71  (-1.00 to 2.51) | 0.15  (-1.13 to 1.59) | N/A | -0.02  (-0.42 to 0.38) |
| Risk of AEs in maintenance therapy | BI 695501 | RZB-180 | 0.20  (-1.40 to 1.92) | 0.18  (-0.71 to 1.07) | 0.22  (-1.40 to 1.89) | 0.03  (-1.32 to 1.32) | 0.47  (-1.41 to 2.38) | 0.19  (-1.40 to 1.91) | N/A | 0.00  (-0.44 to 0.43) |
| Risk of AEs in maintenance therapy | BI 695501 | RZB-360 | 0.17  (-1.43 to 1.88) | 0.15  (-0.73 to 1.04) | 0.20  (-1.44 to 1.87) | 0.01  (-1.35 to 1.29) | 0.44  (-1.42 to 2.36) | 0.17  (-1.43 to 1.88) | N/A | -0.01  (-0.45 to 0.41) |
| Risk of AEs in maintenance therapy | BI 695501 | Vedolizumab | -0.05  (-1.50 to 1.68) | -0.13  (-1.09 to 0.82) | -0.04  (-1.50 to 1.65) | -0.24  (-1.55 to 1.09) | 0.23  (-1.42 to 2.16) | -0.07  (-1.51 to 1.67) | N/A | -0.06  (-0.46 to 0.36) |
| Risk of AEs in maintenance therapy | BI 695501 | Vedolizumab SC | 0.25  (-1.35 to 1.98) | 0.23  (-0.66 to 1.13) | 0.27  (-1.36 to 1.95) | 0.09  (-1.27 to 1.38) | 0.53  (-1.35 to 2.45) | 0.25  (-1.37 to 1.96) | N/A | 0.00  (-0.43 to 0.42) |
| Risk of AEs in maintenance therapy | BI 695501 | placebo | 0.11  (-1.14 to 1.45) | 0.09  (-0.67 to 0.85) | 0.13  (-1.14 to 1.43) | -0.06  (-1.16 to 0.98) | 0.38  (-1.10 to 1.88) | 0.11  (-1.14 to 1.45) | N/A | -0.03  (-0.43 to 0.36) |
| Risk of AEs in maintenance therapy | Certolizumab Pegol | Ustekinumab | 0.06  (-0.90 to 1.09) | 0.01  (-0.48 to 0.50) | 0.05  (-0.90 to 1.08) | 0.12  (-0.61 to 0.93) | 0.22  (-1.22 to 1.71) | 0.06  (-0.90 to 1.09) | N/A | 0.02  (-0.13 to 0.16) |
| Risk of AEs in maintenance therapy | Certolizumab Pegol | RZB-180 | 0.11  (-1.14 to 1.36) | 0.11  (-0.43 to 0.65) | 0.11  (-1.14 to 1.36) | 0.11  (-0.83 to 1.04) | -0.02  (-1.68 to 1.62) | 0.10  (-1.14 to 1.37) | N/A | 0.03  (-0.17 to 0.23) |
| Risk of AEs in maintenance therapy | Certolizumab Pegol | RZB-360 | 0.09  (-1.18 to 1.34) | 0.08  (-0.45 to 0.63) | 0.09  (-1.17 to 1.33) | 0.08  (-0.86 to 1.02) | -0.05  (-1.69 to 1.59) | 0.08  (-1.17 to 1.34) | N/A | 0.03  (-0.18 to 0.22) |
| Risk of AEs in maintenance therapy | Certolizumab Pegol | Vedolizumab | -0.14  (-1.19 to 1.09) | -0.20  (-0.86 to 0.44) | -0.15  (-1.20 to 1.09) | -0.17  (-1.06 to 0.80) | -0.27  (-1.65 to 1.37) | -0.15  (-1.20 to 1.10) | N/A | -0.02  (-0.17 to 0.13) |
| Risk of AEs in maintenance therapy | Certolizumab Pegol | Vedolizumab SC | 0.16  (-1.10 to 1.43) | 0.16  (-0.39 to 0.72) | 0.17  (-1.09 to 1.41) | 0.16  (-0.79 to 1.11) | 0.03  (-1.62 to 1.68) | 0.16  (-1.11 to 1.42) | N/A | 0.04  (-0.16 to 0.23) |
| Risk of AEs in maintenance therapy | Certolizumab Pegol | placebo | 0.02  (-0.71 to 0.73) | 0.02  (-0.26 to 0.30) | 0.02  (-0.71 to 0.72) | 0.01  (-0.51 to 0.54) | -0.11  (-1.28 to 1.04) | 0.01  (-0.70 to 0.73) | N/A | 0.01  (-0.11 to 0.11) |
| Risk of AEs in maintenance therapy | Ustekinumab | RZB-180 | 0.06  (-1.22 to 1.26) | 0.10  (-0.51 to 0.71) | 0.05  (-1.22 to 1.25) | -0.02  (-1.02 to 0.90) | -0.24  (-1.76 to 1.21) | 0.05  (-1.23 to 1.26) | N/A | 0.02  (-0.18 to 0.21) |
| Risk of AEs in maintenance therapy | Ustekinumab | RZB-360 | 0.03  (-1.25 to 1.23) | 0.07  (-0.54 to 0.69) | 0.03  (-1.25 to 1.23) | -0.05  (-1.04 to 0.88) | -0.27  (-1.78 to 1.19) | 0.02  (-1.25 to 1.22) | N/A | 0.01  (-0.19 to 0.20) |
| Risk of AEs in maintenance therapy | Ustekinumab | Vedolizumab | -0.20  (-1.28 to 0.98) | -0.22  (-0.94 to 0.49) | -0.20  (-1.28 to 0.98) | -0.29  (-1.24 to 0.66) | -0.49  (-1.73 to 0.94) | -0.21  (-1.29 to 0.99) | N/A | -0.03  (-0.18 to 0.11) |
| Risk of AEs in maintenance therapy | Ustekinumab | Vedolizumab SC | 0.10  (-1.18 to 1.33) | 0.15  (-0.47 to 0.79) | 0.11  (-1.19 to 1.32) | 0.03  (-0.97 to 0.97) | -0.18  (-1.70 to 1.26) | 0.10  (-1.21 to 1.31) | N/A | 0.02  (-0.17 to 0.21) |
| Risk of AEs in maintenance therapy | Ustekinumab | placebo | -0.05  (-0.77 to 0.62) | 0.01  (-0.40 to 0.41) | -0.04  (-0.77 to 0.62) | -0.11  (-0.71 to 0.42) | -0.34  (-1.27 to 0.55) | -0.04  (-0.77 to 0.61) | N/A | -0.01  (-0.12 to 0.08) |
| Risk of AEs in maintenance therapy | RZB-180 | RZB-360 | -0.03  (-1.06 to 1.01) | -0.03  (-0.49 to 0.43) | -0.02  (-1.06 to 1.00) | -0.03  (-0.80 to 0.75) | -0.03  (-1.20 to 1.15) | -0.03  (-1.07 to 1.02) | N/A | -0.01  (-0.18 to 0.16) |
| Risk of AEs in maintenance therapy | RZB-180 | Vedolizumab | -0.26  (-1.53 to 1.22) | -0.32  (-1.07 to 0.43) | -0.26  (-1.52 to 1.20) | -0.27  (-1.32 to 0.85) | -0.25  (-1.63 to 1.41) | -0.26  (-1.53 to 1.22) | N/A | -0.05  (-0.25 to 0.15) |
| Risk of AEs in maintenance therapy | RZB-180 | Vedolizumab SC | 0.04  (-1.41 to 1.53) | 0.05  (-0.61 to 0.72) | 0.05  (-1.41 to 1.51) | 0.06  (-1.05 to 1.16) | 0.05  (-1.60 to 1.72) | 0.05  (-1.42 to 1.52) | N/A | 0.01  (-0.22 to 0.24) |
| Risk of AEs in maintenance therapy | RZB-180 | placebo | -0.10  (-1.13 to 0.93) | -0.10  (-0.56 to 0.37) | -0.09  (-1.12 to 0.93) | -0.09  (-0.87 to 0.68) | -0.10  (-1.27 to 1.08) | -0.09  (-1.14 to 0.94) | N/A | -0.03  (-0.20 to 0.14) |
| Risk of AEs in maintenance therapy | RZB-360 | Vedolizumab | -0.23  (-1.50 to 1.24) | -0.29  (-1.04 to 0.46) | -0.23  (-1.50 to 1.24) | -0.24  (-1.30 to 0.88) | -0.22  (-1.60 to 1.44) | -0.23  (-1.50 to 1.24) | N/A | -0.04  (-0.24 to 0.16) |
| Risk of AEs in maintenance therapy | RZB-360 | Vedolizumab SC | 0.07  (-1.38 to 1.55) | 0.08  (-0.59 to 0.75) | 0.08  (-1.39 to 1.54) | 0.08  (-1.03 to 1.19) | 0.09  (-1.58 to 1.75) | 0.08  (-1.40 to 1.54) | N/A | 0.01  (-0.22 to 0.25) |
| Risk of AEs in maintenance therapy | RZB-360 | placebo | -0.08  (-1.11 to 0.97) | -0.07  (-0.53 to 0.39) | -0.07  (-1.10 to 0.96) | -0.06  (-0.85 to 0.71) | -0.06  (-1.23 to 1.10) | -0.06  (-1.11 to 0.96) | N/A | -0.02  (-0.19 to 0.15) |
| Risk of AEs in maintenance therapy | Vedolizumab | Vedolizumab SC | 0.31  (-1.16 to 1.60) | 0.37  (-0.39 to 1.14) | 0.31  (-1.16 to 1.58) | 0.33  (-0.81 to 1.39) | 0.30  (-1.36 to 1.68) | 0.32  (-1.18 to 1.58) | N/A | 0.06  (-0.14 to 0.25) |
| Risk of AEs in maintenance therapy | Vedolizumab | placebo | 0.15  (-0.83 to 0.98) | 0.22  (-0.36 to 0.82) | 0.16  (-0.82 to 0.98) | 0.17  (-0.62 to 0.92) | 0.15  (-0.95 to 1.01) | 0.16  (-0.84 to 0.99) | N/A | 0.02  (-0.09 to 0.13) |
| Risk of AEs in maintenance therapy | Vedolizumab SC | placebo | -0.15  (-1.20 to 0.88) | -0.14  (-0.63 to 0.33) | -0.15  (-1.18 to 0.88) | -0.15  (-0.93 to 0.64) | -0.15  (-1.32 to 1.03) | -0.15  (-1.19 to 0.90) | N/A | -0.03  (-0.19 to 0.13) |
| Risk of SAEs in maintenance therapy | IFX-5 | IFX-10 | 0.30  (-0.55 to 1.15) | 0.30  (-0.16 to 0.77) | 0.30  (-0.56 to 1.15) | N/A | 0.30  (-0.75 to 1.34) | N/A | N/A | 0.22  (-0.49 to 0.93) |
| Risk of SAEs in maintenance therapy | IFX-5 | Infliximab+azathioprine | 0.57  (-0.31 to 1.48) | 0.58  (0.03 to 1.13) | 0.57  (-0.33 to 1.47) | N/A | N/A | N/A | N/A | 0.48  (-0.26 to 1.24) |
| Risk of SAEs in maintenance therapy | IFX-5 | CT-P13 | 0.46  (-0.81 to 1.80) | 0.48  (-0.60 to 1.62) | 0.45  (-0.82 to 1.80) | N/A | 0.49  (-0.92 to 1.92) | N/A | N/A | 0.29  (-0.86 to 1.43) |
| Risk of SAEs in maintenance therapy | IFX-5 | Adalimumab | 0.46  (-0.54 to 1.50) | 0.44  (-0.16 to 1.04) | 0.46  (-0.54 to 1.52) | N/A | 0.58  (-0.64 to 1.90) | N/A | N/A | 0.46  (-0.34 to 1.35) |
| Risk of SAEs in maintenance therapy | IFX-5 | BI 695501 | 2.18  (0.28 to 4.55) | 2.14  (0.56 to 4.24) | 2.15  (0.24 to 4.50) | N/A | 2.31  (0.20 to 4.83) | N/A | N/A | 2.16  (0.50 to 4.34) |
| Risk of SAEs in maintenance therapy | IFX-5 | Certolizumab Pegol | -0.26  (-1.31 to 0.86) | -0.29  (-0.93 to 0.34) | -0.26  (-1.31 to 0.86) | N/A | 0.13  (-1.46 to 1.73) | N/A | N/A | -0.20  (-1.08 to 0.75) |
| Risk of SAEs in maintenance therapy | IFX-5 | Ustekinumab | 0.53  (-0.54 to 1.56) | 0.54  (-0.10 to 1.18) | 0.53  (-0.53 to 1.58) | N/A | 0.35  (-1.00 to 1.71) | N/A | N/A | 0.53  (-0.35 to 1.39) |
| Risk of SAEs in maintenance therapy | IFX-5 | RZB-180 | -0.03  (-1.31 to 1.22) | -0.04  (-0.82 to 0.74) | -0.03  (-1.30 to 1.23) | N/A | -0.04  (-1.56 to 1.47) | N/A | N/A | 0.00  (-1.05 to 1.06) |
| Risk of SAEs in maintenance therapy | IFX-5 | RZB-360 | -0.13  (-1.40 to 1.12) | -0.14  (-0.91 to 0.62) | -0.13  (-1.40 to 1.12) | N/A | -0.14  (-1.66 to 1.38) | N/A | N/A | -0.09  (-1.14 to 0.96) |
| Risk of SAEs in maintenance therapy | IFX-5 | Vedolizumab | -0.31  (-1.39 to 0.81) | -0.31  (-1.00 to 0.38) | -0.30  (-1.39 to 0.82) | N/A | -0.30  (-1.60 to 1.04) | N/A | N/A | -0.15  (-1.02 to 0.76) |
| Risk of SAEs in maintenance therapy | IFX-5 | Vedolizumab SC | 0.19  (-1.14 to 1.48) | 0.18  (-0.68 to 1.00) | 0.18  (-1.13 to 1.48) | N/A | 0.18  (-1.40 to 1.71) | N/A | N/A | 0.22  (-0.89 to 1.31) |
| Risk of SAEs in maintenance therapy | IFX-5 | placebo | -0.06  (-0.90 to 0.78) | -0.06  (-0.51 to 0.39) | -0.05  (-0.90 to 0.79) | N/A | -0.06  (-1.11 to 0.98) | N/A | N/A | -0.06  (-0.75 to 0.63) |
| Risk of SAEs in maintenance therapy | IFX-10 | Infliximab+azathioprine | 0.27  (-0.94 to 1.52) | 0.28  (-0.44 to 1.00) | 0.27  (-0.96 to 1.51) | N/A | N/A | N/A | N/A | 0.26  (-0.77 to 1.30) |
| Risk of SAEs in maintenance therapy | IFX-10 | CT-P13 | 0.16  (-1.38 to 1.74) | 0.18  (-1.00 to 1.41) | 0.15  (-1.36 to 1.75) | N/A | 0.19  (-1.55 to 1.94) | N/A | N/A | 0.06  (-1.27 to 1.40) |
| Risk of SAEs in maintenance therapy | IFX-10 | Adalimumab | 0.16  (-0.84 to 1.21) | 0.14  (-0.48 to 0.75) | 0.16  (-0.85 to 1.22) | N/A | 0.28  (-0.95 to 1.62) | N/A | N/A | 0.23  (-0.57 to 1.14) |
| Risk of SAEs in maintenance therapy | IFX-10 | BI 695501 | 1.88  (-0.01 to 4.24) | 1.84  (0.26 to 3.95) | 1.85  (-0.05 to 4.20) | N/A | 2.01  (-0.13 to 4.57) | N/A | N/A | 1.94  (0.27 to 4.12) |
| Risk of SAEs in maintenance therapy | IFX-10 | Certolizumab Pegol | -0.56  (-1.61 to 0.56) | -0.59  (-1.24 to 0.06) | -0.56  (-1.61 to 0.57) | N/A | -0.18  (-1.77 to 1.44) | N/A | N/A | -0.43  (-1.31 to 0.54) |
| Risk of SAEs in maintenance therapy | IFX-10 | Ustekinumab | 0.23  (-0.85 to 1.27) | 0.24  (-0.42 to 0.89) | 0.23  (-0.84 to 1.28) | N/A | 0.05  (-1.30 to 1.42) | N/A | N/A | 0.30  (-0.58 to 1.18) |
| Risk of SAEs in maintenance therapy | IFX-10 | RZB-180 | -0.33  (-1.61 to 0.93) | -0.34  (-1.13 to 0.45) | -0.33  (-1.61 to 0.94) | N/A | -0.34  (-1.87 to 1.19) | N/A | N/A | -0.22  (-1.29 to 0.85) |
| Risk of SAEs in maintenance therapy | IFX-10 | RZB-360 | -0.44  (-1.70 to 0.82) | -0.44  (-1.22 to 0.34) | -0.44  (-1.71 to 0.83) | N/A | -0.44  (-1.96 to 1.10) | N/A | N/A | -0.31  (-1.37 to 0.76) |
| Risk of SAEs in maintenance therapy | IFX-10 | Vedolizumab | -0.61  (-1.69 to 0.51) | -0.61  (-1.31 to 0.09) | -0.60  (-1.70 to 0.53) | N/A | -0.60  (-1.90 to 0.74) | N/A | N/A | -0.38  (-1.26 to 0.55) |
| Risk of SAEs in maintenance therapy | IFX-10 | Vedolizumab SC | -0.11  (-1.45 to 1.18) | -0.13  (-0.98 to 0.71) | -0.12  (-1.44 to 1.17) | N/A | -0.13  (-1.70 to 1.43) | N/A | N/A | -0.01  (-1.12 to 1.11) |
| Risk of SAEs in maintenance therapy | IFX-10 | placebo | -0.36  (-1.21 to 0.50) | -0.36  (-0.83 to 0.10) | -0.36  (-1.21 to 0.49) | N/A | -0.36  (-1.40 to 0.69) | N/A | N/A | -0.28  (-0.99 to 0.43) |
| Risk of SAEs in maintenance therapy | Infliximab+azathioprine | CT-P13 | -0.12  (-1.67 to 1.46) | -0.10  (-1.31 to 1.17) | -0.12  (-1.67 to 1.51) | N/A | N/A | N/A | N/A | -0.20  (-1.56 to 1.17) |
| Risk of SAEs in maintenance therapy | Infliximab+azathioprine | Adalimumab | -0.12  (-1.45 to 1.26) | -0.14  (-0.96 to 0.67) | -0.12  (-1.45 to 1.27) | N/A | N/A | N/A | N/A | -0.03  (-1.11 to 1.15) |
| Risk of SAEs in maintenance therapy | Infliximab+azathioprine | BI 695501 | 1.60  (-0.51 to 4.13) | 1.57  (-0.12 to 3.72) | 1.58  (-0.54 to 4.10) | N/A | N/A | N/A | N/A | 1.68  (-0.14 to 3.98) |
| Risk of SAEs in maintenance therapy | Infliximab+azathioprine | Certolizumab Pegol | -0.83  (-2.21 to 0.59) | -0.87  (-1.71 to -0.03) | -0.83  (-2.21 to 0.61) | N/A | N/A | N/A | N/A | -0.68  (-1.83 to 0.53) |
| Risk of SAEs in maintenance therapy | Infliximab+azathioprine | Ustekinumab | -0.05  (-1.45 to 1.31) | -0.04  (-0.89 to 0.80) | -0.04  (-1.44 to 1.32) | N/A | N/A | N/A | N/A | 0.04  (-1.12 to 1.18) |
| Risk of SAEs in maintenance therapy | Infliximab+azathioprine | RZB-180 | -0.61  (-2.17 to 0.91) | -0.62  (-1.57 to 0.34) | -0.60  (-2.15 to 0.94) | N/A | N/A | N/A | N/A | -0.48  (-1.78 to 0.82) |
| Risk of SAEs in maintenance therapy | Infliximab+azathioprine | RZB-360 | -0.71  (-2.26 to 0.82) | -0.72  (-1.66 to 0.22) | -0.70  (-2.26 to 0.82) | N/A | N/A | N/A | N/A | -0.58  (-1.87 to 0.72) |
| Risk of SAEs in maintenance therapy | Infliximab+azathioprine | Vedolizumab | -0.89  (-2.29 to 0.54) | -0.89  (-1.78 to -0.01) | -0.87  (-2.28 to 0.56) | N/A | N/A | N/A | N/A | -0.64  (-1.79 to 0.55) |
| Risk of SAEs in maintenance therapy | Infliximab+azathioprine | Vedolizumab SC | -0.39  (-1.99 to 1.16) | -0.40  (-1.42 to 0.59) | -0.39  (-1.99 to 1.18) | N/A | N/A | N/A | N/A | -0.26  (-1.60 to 1.07) |
| Risk of SAEs in maintenance therapy | Infliximab+azathioprine | placebo | -0.63  (-1.87 to 0.57) | -0.64  (-1.35 to 0.07) | -0.62  (-1.85 to 0.60) | N/A | N/A | N/A | N/A | -0.54  (-1.56 to 0.48) |
| Risk of SAEs in maintenance therapy | CT-P13 | Adalimumab | -0.01  (-1.66 to 1.66) | -0.04  (-1.33 to 1.19) | 0.02  (-1.67 to 1.63) | N/A | 0.09  (-1.77 to 2.01) | N/A | N/A | 0.18  (-1.22 to 1.62) |
| Risk of SAEs in maintenance therapy | CT-P13 | BI 695501 | 1.73  (-0.59 to 4.44) | 1.68  (-0.30 to 4.01) | 1.74  (-0.69 to 4.35) | N/A | 1.82  (-0.79 to 4.71) | N/A | N/A | 1.90  (-0.16 to 4.32) |
| Risk of SAEs in maintenance therapy | CT-P13 | Certolizumab Pegol | -0.71  (-2.42 to 0.98) | -0.77  (-2.07 to 0.48) | -0.71  (-2.43 to 0.97) | N/A | -0.37  (-2.49 to 1.75) | N/A | N/A | -0.49  (-1.92 to 0.99) |
| Risk of SAEs in maintenance therapy | CT-P13 | Ustekinumab | 0.06  (-1.63 to 1.73) | 0.06  (-1.25 to 1.31) | 0.09  (-1.64 to 1.69) | N/A | -0.15  (-2.08 to 1.80) | N/A | N/A | 0.24  (-1.20 to 1.66) |
| Risk of SAEs in maintenance therapy | CT-P13 | RZB-180 | -0.50  (-2.34 to 1.28) | -0.52  (-1.90 to 0.81) | -0.48  (-2.35 to 1.29) | N/A | -0.54  (-2.60 to 1.52) | N/A | N/A | -0.29  (-1.84 to 1.26) |
| Risk of SAEs in maintenance therapy | CT-P13 | RZB-360 | -0.60  (-2.43 to 1.20) | -0.62  (-2.00 to 0.70) | -0.58  (-2.45 to 1.17) | N/A | -0.63  (-2.69 to 1.42) | N/A | N/A | -0.37  (-1.92 to 1.16) |
| Risk of SAEs in maintenance therapy | CT-P13 | Vedolizumab | -0.77  (-2.48 to 0.94) | -0.79  (-2.12 to 0.49) | -0.74  (-2.49 to 0.91) | N/A | -0.80  (-2.70 to 1.16) | N/A | N/A | -0.44  (-1.87 to 1.01) |
| Risk of SAEs in maintenance therapy | CT-P13 | Vedolizumab SC | -0.29  (-2.13 to 1.54) | -0.31  (-1.72 to 1.05) | -0.28  (-2.15 to 1.51) | N/A | -0.32  (-2.41 to 1.75) | N/A | N/A | -0.07  (-1.66 to 1.49) |
| Risk of SAEs in maintenance therapy | CT-P13 | placebo | -0.53  (-2.09 to 1.01) | -0.54  (-1.76 to 0.62) | -0.50  (-2.10 to 0.99) | N/A | -0.56  (-2.30 to 1.18) | N/A | N/A | -0.35  (-1.67 to 0.98) |
| Risk of SAEs in maintenance therapy | Adalimumab | BI 695501 | 1.71  (0.10 to 3.84) | 1.70  (0.24 to 3.72) | 1.68  (0.07 to 3.80) | N/A | 1.71  (-0.02 to 3.87) | N/A | N/A | 1.69  (0.24 to 3.71) |
| Risk of SAEs in maintenance therapy | Adalimumab | Certolizumab Pegol | -0.71  (-1.60 to 0.17) | -0.72  (-1.34 to -0.12) | -0.71  (-1.60 to 0.18) | N/A | -0.46  (-1.91 to 0.94) | N/A | N/A | -0.66  (-1.44 to 0.11) |
| Risk of SAEs in maintenance therapy | Adalimumab | Ustekinumab | 0.08  (-0.64 to 0.72) | 0.10  (-0.35 to 0.56) | 0.08  (-0.64 to 0.72) | N/A | -0.23  (-1.41 to 0.89) | N/A | N/A | 0.07  (-0.56 to 0.61) |
| Risk of SAEs in maintenance therapy | Adalimumab | RZB-180 | -0.48  (-1.63 to 0.57) | -0.48  (-1.23 to 0.27) | -0.49  (-1.62 to 0.60) | N/A | -0.62  (-2.00 to 0.68) | N/A | N/A | -0.46  (-1.45 to 0.46) |
| Risk of SAEs in maintenance therapy | Adalimumab | RZB-360 | -0.59  (-1.73 to 0.48) | -0.58  (-1.32 to 0.17) | -0.59  (-1.73 to 0.47) | N/A | -0.71  (-2.10 to 0.56) | N/A | N/A | -0.54  (-1.53 to 0.36) |
| Risk of SAEs in maintenance therapy | Adalimumab | Vedolizumab | -0.76  (-1.70 to 0.14) | -0.75  (-1.42 to -0.08) | -0.76  (-1.69 to 0.16) | N/A | -0.88  (-2.01 to 0.19) | N/A | N/A | -0.61  (-1.39 to 0.11) |
| Risk of SAEs in maintenance therapy | Adalimumab | Vedolizumab SC | -0.27  (-1.46 to 0.83) | -0.26  (-1.08 to 0.54) | -0.28  (-1.47 to 0.84) | N/A | -0.41  (-1.83 to 0.94) | N/A | N/A | -0.24  (-1.28 to 0.72) |
| Risk of SAEs in maintenance therapy | Adalimumab | placebo | -0.51  (-1.12 to 0.04) | -0.50  (-0.90 to -0.09) | -0.51  (-1.12 to 0.04) | N/A | -0.64  (-1.43 to 0.06) | N/A | N/A | -0.52  (-1.05 to -0.06) |
| Risk of SAEs in maintenance therapy | BI 695501 | Certolizumab Pegol | -2.42  (-4.73 to -0.61) | -2.43  (-4.52 to -0.84) | -2.41  (-4.68 to -0.55) | N/A | -2.17  (-4.80 to 0.05) | N/A | N/A | -2.36  (-4.51 to -0.69) |
| Risk of SAEs in maintenance therapy | BI 695501 | Ustekinumab | -1.64  (-3.91 to 0.10) | -1.60  (-3.66 to -0.07) | -1.61  (-3.83 to 0.12) | N/A | -1.95  (-4.41 to 0.11) | N/A | N/A | -1.63  (-3.74 to -0.08) |
| Risk of SAEs in maintenance therapy | BI 695501 | RZB-180 | -2.22  (-4.61 to -0.27) | -2.19  (-4.32 to -0.53) | -2.18  (-4.55 to -0.21) | N/A | -2.35  (-4.92 to -0.18) | N/A | N/A | -2.17  (-4.40 to -0.43) |
| Risk of SAEs in maintenance therapy | BI 695501 | RZB-360 | -2.32  (-4.73 to -0.38) | -2.29  (-4.41 to -0.64) | -2.29  (-4.67 to -0.32) | N/A | -2.44  (-5.01 to -0.29) | N/A | N/A | -2.25  (-4.48 to -0.53) |
| Risk of SAEs in maintenance therapy | BI 695501 | Vedolizumab | -2.50  (-4.79 to -0.62) | -2.46  (-4.56 to -0.84) | -2.45  (-4.76 to -0.59) | N/A | -2.61  (-5.05 to -0.57) | N/A | N/A | -2.32  (-4.46 to -0.68) |
| Risk of SAEs in maintenance therapy | BI 695501 | Vedolizumab SC | -2.00  (-4.47 to -0.02) | -1.98  (-4.12 to -0.29) | -1.97  (-4.38 to 0.00) | N/A | -2.14  (-4.70 to 0.08) | N/A | N/A | -1.96  (-4.19 to -0.19) |
| Risk of SAEs in maintenance therapy | BI 695501 | placebo | -2.23  (-4.45 to -0.53) | -2.20  (-4.25 to -0.68) | -2.20  (-4.38 to -0.48) | N/A | -2.37  (-4.68 to -0.49) | N/A | N/A | -2.21  (-4.31 to -0.69) |
| Risk of SAEs in maintenance therapy | Certolizumab Pegol | Ustekinumab | 0.78  (-0.17 to 1.70) | 0.83  (0.18 to 1.48) | 0.79  (-0.18 to 1.68) | N/A | 0.22  (-1.28 to 1.71) | N/A | N/A | 0.73  (-0.11 to 1.51) |
| Risk of SAEs in maintenance therapy | Certolizumab Pegol | RZB-180 | 0.23  (-0.97 to 1.35) | 0.25  (-0.53 to 1.03) | 0.23  (-0.98 to 1.36) | N/A | -0.17  (-1.82 to 1.47) | N/A | N/A | 0.20  (-0.85 to 1.18) |
| Risk of SAEs in maintenance therapy | Certolizumab Pegol | RZB-360 | 0.12  (-1.07 to 1.25) | 0.15  (-0.63 to 0.92) | 0.12  (-1.08 to 1.26) | N/A | -0.26  (-1.91 to 1.35) | N/A | N/A | 0.11  (-0.92 to 1.08) |
| Risk of SAEs in maintenance therapy | Certolizumab Pegol | Vedolizumab | -0.06  (-1.05 to 0.93) | -0.02  (-0.71 to 0.67) | -0.04  (-1.05 to 0.93) | N/A | -0.42  (-1.88 to 1.04) | N/A | N/A | 0.05  (-0.79 to 0.86) |
| Risk of SAEs in maintenance therapy | Certolizumab Pegol | Vedolizumab SC | 0.44  (-0.80 to 1.60) | 0.46  (-0.38 to 1.29) | 0.44  (-0.82 to 1.61) | N/A | 0.05  (-1.63 to 1.70) | N/A | N/A | 0.42  (-0.67 to 1.44) |
| Risk of SAEs in maintenance therapy | Certolizumab Pegol | placebo | 0.20  (-0.51 to 0.86) | 0.23  (-0.22 to 0.68) | 0.20  (-0.51 to 0.85) | N/A | -0.19  (-1.41 to 1.01) | N/A | N/A | 0.14  (-0.49 to 0.72) |
| Risk of SAEs in maintenance therapy | Ustekinumab | RZB-180 | -0.56  (-1.69 to 0.57) | -0.58  (-1.36 to 0.21) | -0.56  (-1.70 to 0.59) | N/A | -0.39  (-1.81 to 1.04) | N/A | N/A | -0.53  (-1.50 to 0.46) |
| Risk of SAEs in maintenance therapy | Ustekinumab | RZB-360 | -0.67  (-1.79 to 0.47) | -0.68  (-1.45 to 0.10) | -0.67  (-1.79 to 0.47) | N/A | -0.49  (-1.90 to 0.92) | N/A | N/A | -0.61  (-1.59 to 0.37) |
| Risk of SAEs in maintenance therapy | Ustekinumab | Vedolizumab | -0.84  (-1.78 to 0.14) | -0.85  (-1.55 to -0.15) | -0.83  (-1.77 to 0.16) | N/A | -0.66  (-1.83 to 0.56) | N/A | N/A | -0.68  (-1.45 to 0.13) |
| Risk of SAEs in maintenance therapy | Ustekinumab | Vedolizumab SC | -0.34  (-1.53 to 0.83) | -0.36  (-1.21 to 0.48) | -0.36  (-1.53 to 0.82) | N/A | -0.18  (-1.63 to 1.25) | N/A | N/A | -0.31  (-1.34 to 0.72) |
| Risk of SAEs in maintenance therapy | Ustekinumab | placebo | -0.59  (-1.22 to 0.05) | -0.60  (-1.06 to -0.14) | -0.59  (-1.22 to 0.05) | N/A | -0.42  (-1.29 to 0.46) | N/A | N/A | -0.58  (-1.13 to -0.03) |
| Risk of SAEs in maintenance therapy | RZB-180 | RZB-360 | -0.11  (-1.04 to 0.84) | -0.10  (-0.73 to 0.52) | -0.11  (-1.05 to 0.84) | N/A | -0.09  (-1.21 to 1.00) | N/A | N/A | -0.09  (-0.90 to 0.72) |
| Risk of SAEs in maintenance therapy | RZB-180 | Vedolizumab | -0.29  (-1.43 to 0.92) | -0.27  (-1.09 to 0.55) | -0.27  (-1.44 to 0.94) | N/A | -0.26  (-1.61 to 1.14) | N/A | N/A | -0.16  (-1.13 to 0.86) |
| Risk of SAEs in maintenance therapy | RZB-180 | Vedolizumab SC | 0.21  (-1.16 to 1.58) | 0.21  (-0.75 to 1.16) | 0.21  (-1.18 to 1.57) | N/A | 0.22  (-1.40 to 1.81) | N/A | N/A | 0.22  (-0.98 to 1.40) |
| Risk of SAEs in maintenance therapy | RZB-180 | placebo | -0.03  (-0.96 to 0.92) | -0.02  (-0.65 to 0.62) | -0.03  (-0.97 to 0.93) | N/A | -0.02  (-1.14 to 1.09) | N/A | N/A | -0.06  (-0.87 to 0.75) |
| Risk of SAEs in maintenance therapy | RZB-360 | Vedolizumab | -0.18  (-1.33 to 1.02) | -0.17  (-0.98 to 0.65) | -0.17  (-1.33 to 1.04) | N/A | -0.16  (-1.51 to 1.22) | N/A | N/A | -0.07  (-1.04 to 0.94) |
| Risk of SAEs in maintenance therapy | RZB-360 | Vedolizumab SC | 0.32  (-1.05 to 1.68) | 0.32  (-0.64 to 1.25) | 0.32  (-1.07 to 1.68) | N/A | 0.32  (-1.30 to 1.91) | N/A | N/A | 0.30  (-0.88 to 1.48) |
| Risk of SAEs in maintenance therapy | RZB-360 | placebo | 0.08  (-0.85 to 1.01) | 0.08  (-0.54 to 0.71) | 0.08  (-0.85 to 1.03) | N/A | 0.07  (-1.03 to 1.19) | N/A | N/A | 0.03  (-0.77 to 0.83) |
| Risk of SAEs in maintenance therapy | Vedolizumab | Vedolizumab SC | 0.49  (-0.77 to 1.69) | 0.49  (-0.41 to 1.36) | 0.48  (-0.77 to 1.68) | N/A | 0.48  (-0.96 to 1.86) | N/A | N/A | 0.37  (-0.69 to 1.39) |
| Risk of SAEs in maintenance therapy | Vedolizumab | placebo | 0.25  (-0.47 to 0.96) | 0.25  (-0.28 to 0.78) | 0.24  (-0.48 to 0.95) | N/A | 0.24  (-0.58 to 1.03) | N/A | N/A | 0.10  (-0.48 to 0.65) |
| Risk of SAEs in maintenance therapy | Vedolizumab SC | placebo | -0.24  (-1.22 to 0.77) | -0.24  (-0.93 to 0.48) | -0.24  (-1.22 to 0.78) | N/A | -0.24  (-1.38 to 0.93) | N/A | N/A | -0.27  (-1.13 to 0.60) |
| Risk of SIs in maintenance therapy | IFX-5 | IFX-10 | 0.30  (-1.19 to 1.82) | 0.30  (-0.79 to 1.46) | 0.32  (-1.15 to 1.81) | N/A | 0.31  (-1.30 to 1.95) | N/A | N/A | 0.29  (-1.12 to 1.74) |
| Risk of SIs in maintenance therapy | IFX-5 | Infliximab+azathioprine | 0.24  (-1.19 to 1.73) | 0.25  (-0.81 to 1.34) | 0.25  (-1.17 to 1.69) | N/A | N/A | N/A | N/A | 0.35  (-1.01 to 1.75) |
| Risk of SIs in maintenance therapy | IFX-5 | Adalimumab | 0.23  (-1.51 to 2.08) | 0.24  (-1.10 to 1.57) | 0.26  (-1.55 to 2.06) | N/A | 0.36  (-1.68 to 2.49) | N/A | N/A | 0.27  (-1.38 to 1.98) |
| Risk of SIs in maintenance therapy | IFX-5 | BI 695501 | 0.22  (-2.82 to 3.26) | 0.20  (-2.43 to 2.80) | 0.28  (-2.68 to 3.30) | N/A | 0.34  (-2.86 to 3.67) | N/A | N/A | 0.71  (-1.98 to 3.56) |
| Risk of SIs in maintenance therapy | IFX-5 | Certolizumab Pegol | -1.08  (-3.04 to 0.84) | -1.03  (-2.57 to 0.40) | -1.03  (-3.01 to 0.83) | N/A | -1.24  (-4.03 to 1.22) | N/A | N/A | -0.81  (-2.58 to 0.94) |
| Risk of SIs in maintenance therapy | IFX-5 | Ustekinumab | 0.28  (-1.55 to 2.17) | 0.30  (-1.14 to 1.74) | 0.32  (-1.53 to 2.15) | N/A | 0.17  (-2.00 to 2.37) | N/A | N/A | 0.48  (-1.24 to 2.28) |
| Risk of SIs in maintenance therapy | IFX-5 | RZB-180 | 0.29  (-1.84 to 2.42) | 0.32  (-1.27 to 1.93) | 0.33  (-1.76 to 2.42) | N/A | 0.32  (-1.97 to 2.62) | N/A | N/A | 0.30  (-1.67 to 2.33) |
| Risk of SIs in maintenance therapy | IFX-5 | RZB-360 | -0.23  (-2.30 to 1.83) | -0.20  (-1.70 to 1.28) | -0.20  (-2.21 to 1.81) | N/A | -0.19  (-2.45 to 2.02) | N/A | N/A | -0.20  (-2.13 to 1.71) |
| Risk of SIs in maintenance therapy | IFX-5 | Vedolizumab | -0.26  (-2.39 to 1.92) | -0.22  (-1.87 to 1.41) | -0.22  (-2.34 to 1.91) | N/A | -0.20  (-2.58 to 2.09) | N/A | N/A | -0.16  (-2.16 to 1.81) |
| Risk of SIs in maintenance therapy | IFX-5 | Vedolizumab SC | 1.12  (-1.04 to 3.38) | 1.17  (-0.51 to 2.91) | 1.18  (-1.01 to 3.35) | N/A | 1.17  (-1.19 to 3.57) | N/A | N/A | 1.16  (-0.87 to 3.27) |
| Risk of SIs in maintenance therapy | IFX-5 | placebo | -0.05  (-1.49 to 1.41) | -0.03  (-1.06 to 1.00) | -0.03  (-1.45 to 1.38) | N/A | -0.04  (-1.60 to 1.54) | N/A | N/A | -0.16  (-1.50 to 1.19) |
| Risk of SIs in maintenance therapy | IFX-10 | Infliximab+azathioprine | -0.06  (-2.16 to 2.02) | -0.06  (-1.61 to 1.49) | -0.05  (-2.12 to 2.02) | N/A | N/A | N/A | N/A | 0.07  (-1.92 to 2.03) |
| Risk of SIs in maintenance therapy | IFX-10 | Adalimumab | -0.06  (-1.86 to 1.79) | -0.07  (-1.50 to 1.32) | -0.06  (-1.88 to 1.78) | N/A | 0.05  (-2.05 to 2.22) | N/A | N/A | -0.02  (-1.76 to 1.73) |
| Risk of SIs in maintenance therapy | IFX-10 | BI 695501 | -0.09  (-3.13 to 2.96) | -0.11  (-2.78 to 2.51) | -0.04  (-3.04 to 2.97) | N/A | 0.03  (-3.20 to 3.38) | N/A | N/A | 0.41  (-2.25 to 3.28) |
| Risk of SIs in maintenance therapy | IFX-10 | Certolizumab Pegol | -1.39  (-3.39 to 0.56) | -1.34  (-2.96 to 0.15) | -1.35  (-3.34 to 0.55) | N/A | -1.56  (-4.36 to 0.94) | N/A | N/A | -1.09  (-2.96 to 0.68) |
| Risk of SIs in maintenance therapy | IFX-10 | Ustekinumab | -0.01  (-1.90 to 1.87) | -0.01  (-1.52 to 1.48) | 0.00  (-1.89 to 1.87) | N/A | -0.14  (-2.35 to 2.09) | N/A | N/A | 0.19  (-1.61 to 1.97) |
| Risk of SIs in maintenance therapy | IFX-10 | RZB-180 | -0.02  (-2.17 to 2.15) | 0.01  (-1.64 to 1.67) | 0.01  (-2.12 to 2.13) | N/A | 0.02  (-2.34 to 2.35) | N/A | N/A | 0.02  (-2.04 to 2.07) |
| Risk of SIs in maintenance therapy | IFX-10 | RZB-360 | -0.53  (-2.63 to 1.54) | -0.51  (-2.09 to 1.01) | -0.52  (-2.59 to 1.53) | N/A | -0.50  (-2.82 to 1.75) | N/A | N/A | -0.49  (-2.47 to 1.45) |
| Risk of SIs in maintenance therapy | IFX-10 | Vedolizumab | -0.56  (-2.74 to 1.65) | -0.53  (-2.25 to 1.14) | -0.52  (-2.68 to 1.61) | N/A | -0.51  (-2.93 to 1.81) | N/A | N/A | -0.45  (-2.51 to 1.56) |
| Risk of SIs in maintenance therapy | IFX-10 | Vedolizumab SC | 0.82  (-1.37 to 3.11) | 0.86  (-0.89 to 2.65) | 0.86  (-1.32 to 3.06) | N/A | 0.86  (-1.54 to 3.29) | N/A | N/A | 0.88  (-1.25 to 3.00) |
| Risk of SIs in maintenance therapy | IFX-10 | placebo | -0.35  (-1.85 to 1.13) | -0.33  (-1.49 to 0.76) | -0.33  (-1.83 to 1.12) | N/A | -0.34  (-1.99 to 1.28) | N/A | N/A | -0.45  (-1.90 to 0.93) |
| Risk of SIs in maintenance therapy | Infliximab+azathioprine | Adalimumab | -0.01  (-2.28 to 2.33) | -0.01  (-1.73 to 1.68) | 0.00  (-2.30 to 2.28) | N/A | N/A | N/A | N/A | -0.09  (-2.23 to 2.11) |
| Risk of SIs in maintenance therapy | Infliximab+azathioprine | BI 695501 | -0.03  (-3.36 to 3.34) | -0.05  (-2.89 to 2.75) | 0.01  (-3.31 to 3.34) | N/A | N/A | N/A | N/A | 0.36  (-2.60 to 3.46) |
| Risk of SIs in maintenance therapy | Infliximab+azathioprine | Certolizumab Pegol | -1.32  (-3.78 to 1.08) | -1.29  (-3.16 to 0.50) | -1.29  (-3.72 to 1.06) | N/A | N/A | N/A | N/A | -1.17  (-3.40 to 1.06) |
| Risk of SIs in maintenance therapy | Infliximab+azathioprine | Ustekinumab | 0.04  (-2.32 to 2.43) | 0.05  (-1.75 to 1.83) | 0.04  (-2.28 to 2.36) | N/A | N/A | N/A | N/A | 0.13  (-2.07 to 2.34) |
| Risk of SIs in maintenance therapy | Infliximab+azathioprine | RZB-180 | 0.04  (-2.54 to 2.61) | 0.07  (-1.85 to 1.98) | 0.07  (-2.45 to 2.57) | N/A | N/A | N/A | N/A | -0.06  (-2.44 to 2.40) |
| Risk of SIs in maintenance therapy | Infliximab+azathioprine | RZB-360 | -0.47  (-3.00 to 2.02) | -0.46  (-2.31 to 1.36) | -0.46  (-2.94 to 1.99) | N/A | N/A | N/A | N/A | -0.56  (-2.91 to 1.78) |
| Risk of SIs in maintenance therapy | Infliximab+azathioprine | Vedolizumab | -0.50  (-3.08 to 2.11) | -0.47  (-2.45 to 1.45) | -0.48  (-2.99 to 2.08) | N/A | N/A | N/A | N/A | -0.52  (-2.95 to 1.89) |
| Risk of SIs in maintenance therapy | Infliximab+azathioprine | Vedolizumab SC | 0.89  (-1.74 to 3.54) | 0.92  (-1.10 to 2.95) | 0.93  (-1.67 to 3.55) | N/A | N/A | N/A | N/A | 0.79  (-1.64 to 3.32) |
| Risk of SIs in maintenance therapy | Infliximab+azathioprine | placebo | -0.29  (-2.37 to 1.75) | -0.28  (-1.78 to 1.20) | -0.28  (-2.31 to 1.70) | N/A | N/A | N/A | N/A | -0.52  (-2.43 to 1.41) |
| Risk of SIs in maintenance therapy | Adalimumab | BI 695501 | -0.03  (-2.51 to 2.41) | -0.04  (-2.30 to 2.22) | 0.02  (-2.42 to 2.47) | N/A | -0.02  (-2.56 to 2.51) | N/A | N/A | 0.42  (-1.61 to 2.70) |
| Risk of SIs in maintenance therapy | Adalimumab | Certolizumab Pegol | -1.32  (-3.05 to 0.33) | -1.27  (-2.70 to 0.05) | -1.29  (-3.01 to 0.27) | N/A | -1.61  (-4.30 to 0.73) | N/A | N/A | -1.08  (-2.65 to 0.42) |
| Risk of SIs in maintenance therapy | Adalimumab | Ustekinumab | 0.04  (-1.21 to 1.29) | 0.06  (-0.96 to 1.10) | 0.05  (-1.18 to 1.27) | N/A | -0.19  (-2.27 to 1.85) | N/A | N/A | 0.21  (-0.98 to 1.42) |
| Risk of SIs in maintenance therapy | Adalimumab | RZB-180 | 0.04  (-1.87 to 1.91) | 0.08  (-1.38 to 1.58) | 0.06  (-1.81 to 1.95) | N/A | -0.04  (-2.26 to 2.08) | N/A | N/A | 0.03  (-1.78 to 1.83) |
| Risk of SIs in maintenance therapy | Adalimumab | RZB-360 | -0.48  (-2.32 to 1.31) | -0.44  (-1.82 to 0.91) | -0.46  (-2.27 to 1.29) | N/A | -0.56  (-2.70 to 1.48) | N/A | N/A | -0.47  (-2.19 to 1.20) |
| Risk of SIs in maintenance therapy | Adalimumab | Vedolizumab | -0.49  (-2.44 to 1.42) | -0.46  (-1.99 to 1.05) | -0.46  (-2.39 to 1.41) | N/A | -0.57  (-2.83 to 1.57) | N/A | N/A | -0.44  (-2.25 to 1.34) |
| Risk of SIs in maintenance therapy | Adalimumab | Vedolizumab SC | 0.88  (-1.06 to 2.89) | 0.93  (-0.63 to 2.56) | 0.91  (-1.03 to 2.93) | N/A | 0.82  (-1.47 to 3.06) | N/A | N/A | 0.88  (-0.96 to 2.78) |
| Risk of SIs in maintenance therapy | Adalimumab | placebo | -0.29  (-1.40 to 0.75) | -0.26  (-1.12 to 0.57) | -0.27  (-1.39 to 0.76) | N/A | -0.39  (-1.81 to 0.93) | N/A | N/A | -0.43  (-1.46 to 0.55) |
| Risk of SIs in maintenance therapy | BI 695501 | Certolizumab Pegol | -1.29  (-4.31 to 1.64) | -1.24  (-3.88 to 1.38) | -1.31  (-4.29 to 1.58) | N/A | -1.63  (-5.23 to 1.86) | N/A | N/A | -1.50  (-4.24 to 1.03) |
| Risk of SIs in maintenance therapy | BI 695501 | Ustekinumab | 0.08  (-2.67 to 2.85) | 0.10  (-2.37 to 2.59) | 0.04  (-2.68 to 2.74) | N/A | -0.16  (-3.47 to 3.05) | N/A | N/A | -0.22  (-2.80 to 2.17) |
| Risk of SIs in maintenance therapy | BI 695501 | RZB-180 | 0.07  (-3.02 to 3.13) | 0.12  (-2.56 to 2.83) | 0.07  (-3.04 to 3.13) | N/A | -0.02  (-3.40 to 3.27) | N/A | N/A | -0.41  (-3.28 to 2.34) |
| Risk of SIs in maintenance therapy | BI 695501 | RZB-360 | -0.44  (-3.51 to 2.57) | -0.41  (-3.03 to 2.23) | -0.47  (-3.50 to 2.56) | N/A | -0.53  (-3.88 to 2.70) | N/A | N/A | -0.91  (-3.71 to 1.75) |
| Risk of SIs in maintenance therapy | BI 695501 | Vedolizumab | -0.47  (-3.57 to 2.64) | -0.42  (-3.15 to 2.30) | -0.47  (-3.60 to 2.56) | N/A | -0.57  (-3.95 to 2.73) | N/A | N/A | -0.86  (-3.74 to 1.86) |
| Risk of SIs in maintenance therapy | BI 695501 | Vedolizumab SC | 0.91  (-2.21 to 4.06) | 0.97  (-1.76 to 3.74) | 0.90  (-2.16 to 4.08) | N/A | 0.83  (-2.58 to 4.18) | N/A | N/A | 0.46  (-2.43 to 3.21) |
| Risk of SIs in maintenance therapy | BI 695501 | placebo | -0.27  (-2.92 to 2.42) | -0.23  (-2.63 to 2.19) | -0.30  (-2.96 to 2.33) | N/A | -0.37  (-3.31 to 2.47) | N/A | N/A | -0.86  (-3.36 to 1.39) |
| Risk of SIs in maintenance therapy | Certolizumab Pegol | Ustekinumab | 1.37  (-0.36 to 3.15) | 1.33  (-0.09 to 2.87) | 1.34  (-0.33 to 3.10) | N/A | 1.42  (-1.08 to 4.19) | N/A | N/A | 1.28  (-0.27 to 2.91) |
| Risk of SIs in maintenance therapy | Certolizumab Pegol | RZB-180 | 1.37  (-0.63 to 3.44) | 1.35  (-0.22 to 3.04) | 1.35  (-0.59 to 3.43) | N/A | 1.57  (-0.99 to 4.38) | N/A | N/A | 1.10  (-0.72 to 3.04) |
| Risk of SIs in maintenance therapy | Certolizumab Pegol | RZB-360 | 0.85  (-1.08 to 2.81) | 0.83  (-0.66 to 2.38) | 0.83  (-1.07 to 2.79) | N/A | 1.06  (-1.44 to 3.80) | N/A | N/A | 0.60  (-1.16 to 2.41) |
| Risk of SIs in maintenance therapy | Certolizumab Pegol | Vedolizumab | 0.83  (-1.21 to 2.90) | 0.81  (-0.82 to 2.50) | 0.83  (-1.23 to 2.88) | N/A | 1.04  (-1.56 to 3.82) | N/A | N/A | 0.64  (-1.23 to 2.55) |
| Risk of SIs in maintenance therapy | Certolizumab Pegol | Vedolizumab SC | 2.21  (0.16 to 4.39) | 2.20  (0.53 to 4.01) | 2.20  (0.22 to 4.34) | N/A | 2.45  (-0.22 to 5.27) | N/A | N/A | 1.96  (0.08 to 3.96) |
| Risk of SIs in maintenance therapy | Certolizumab Pegol | placebo | 1.02  (-0.22 to 2.36) | 0.99  (0.00 to 2.16) | 1.00  (-0.21 to 2.35) | N/A | 1.21  (-0.70 to 3.51) | N/A | N/A | 0.64  (-0.47 to 1.83) |
| Risk of SIs in maintenance therapy | Ustekinumab | RZB-180 | 0.00  (-1.97 to 1.97) | 0.02  (-1.54 to 1.61) | 0.02  (-1.90 to 1.95) | N/A | 0.15  (-2.15 to 2.41) | N/A | N/A | -0.18  (-2.03 to 1.71) |
| Risk of SIs in maintenance therapy | Ustekinumab | RZB-360 | -0.52  (-2.41 to 1.34) | -0.50  (-1.98 to 0.95) | -0.51  (-2.36 to 1.31) | N/A | -0.38  (-2.58 to 1.80) | N/A | N/A | -0.68  (-2.53 to 1.06) |
| Risk of SIs in maintenance therapy | Ustekinumab | Vedolizumab | -0.53  (-2.56 to 1.45) | -0.52  (-2.17 to 1.07) | -0.51  (-2.50 to 1.44) | N/A | -0.38  (-2.73 to 1.86) | N/A | N/A | -0.64  (-2.52 to 1.19) |
| Risk of SIs in maintenance therapy | Ustekinumab | Vedolizumab SC | 0.84  (-1.17 to 2.92) | 0.86  (-0.79 to 2.58) | 0.87  (-1.13 to 2.97) | N/A | 1.00  (-1.31 to 3.34) | N/A | N/A | 0.68  (-1.21 to 2.63) |
| Risk of SIs in maintenance therapy | Ustekinumab | placebo | -0.33  (-1.54 to 0.83) | -0.33  (-1.34 to 0.66) | -0.34  (-1.50 to 0.82) | N/A | -0.20  (-1.74 to 1.30) | N/A | N/A | -0.63  (-1.81 to 0.45) |
| Risk of SIs in maintenance therapy | RZB-180 | RZB-360 | -0.51  (-2.10 to 0.99) | -0.51  (-1.75 to 0.62) | -0.52  (-2.08 to 0.97) | N/A | -0.52  (-2.20 to 1.11) | N/A | N/A | -0.50  (-2.02 to 0.95) |
| Risk of SIs in maintenance therapy | RZB-180 | Vedolizumab | -0.53  (-2.80 to 1.73) | -0.54  (-2.32 to 1.20) | -0.54  (-2.80 to 1.65) | N/A | -0.52  (-2.94 to 1.85) | N/A | N/A | -0.47  (-2.57 to 1.61) |
| Risk of SIs in maintenance therapy | RZB-180 | Vedolizumab SC | 0.85  (-1.39 to 3.16) | 0.85  (-0.95 to 2.69) | 0.84  (-1.36 to 3.13) | N/A | 0.85  (-1.57 to 3.33) | N/A | N/A | 0.86  (-1.28 to 3.01) |
| Risk of SIs in maintenance therapy | RZB-180 | placebo | -0.33  (-1.92 to 1.22) | -0.34  (-1.60 to 0.85) | -0.34  (-1.91 to 1.18) | N/A | -0.35  (-2.04 to 1.34) | N/A | N/A | -0.46  (-1.99 to 0.98) |
| Risk of SIs in maintenance therapy | RZB-360 | Vedolizumab | -0.01  (-2.18 to 2.18) | -0.02  (-1.68 to 1.64) | 0.00  (-2.17 to 2.12) | N/A | 0.00  (-2.37 to 2.32) | N/A | N/A | 0.03  (-1.97 to 2.06) |
| Risk of SIs in maintenance therapy | RZB-360 | Vedolizumab SC | 1.37  (-0.80 to 3.59) | 1.37  (-0.31 to 3.14) | 1.37  (-0.77 to 3.58) | N/A | 1.37  (-1.01 to 3.80) | N/A | N/A | 1.36  (-0.69 to 3.48) |
| Risk of SIs in maintenance therapy | RZB-360 | placebo | 0.18  (-1.27 to 1.66) | 0.17  (-0.88 to 1.26) | 0.16  (-1.25 to 1.62) | N/A | 0.16  (-1.42 to 1.77) | N/A | N/A | 0.04  (-1.33 to 1.41) |
| Risk of SIs in maintenance therapy | Vedolizumab | Vedolizumab SC | 1.38  (-0.91 to 3.76) | 1.39  (-0.43 to 3.29) | 1.39  (-0.84 to 3.71) | N/A | 1.38  (-1.06 to 3.93) | N/A | N/A | 1.33  (-0.78 to 3.51) |
| Risk of SIs in maintenance therapy | Vedolizumab | placebo | 0.21  (-1.41 to 1.82) | 0.19  (-1.06 to 1.49) | 0.18  (-1.37 to 1.78) | N/A | 0.17  (-1.52 to 1.94) | N/A | N/A | 0.00  (-1.47 to 1.48) |
| Risk of SIs in maintenance therapy | Vedolizumab SC | placebo | -1.17  (-2.90 to 0.44) | -1.19  (-2.61 to 0.11) | -1.19  (-2.89 to 0.40) | N/A | -1.20  (-3.03 to 0.55) | N/A | N/A | -1.31  (-2.94 to 0.21) |
| **TNF antagonists-naïve CD patients** | | | | | | | | | | |
| Inducing clinical remission | Infliximab | Infliximab+azathioprine | -0.12  (-1.53 to 1.31) | -0.12  (-0.55 to 0.31) | -0.12  (-1.54 to 1.29) | N/A | N/A | -0.12  (-1.49 to 1.25) | N/A | -0.05  (-1.21 to 1.11) |
| Inducing clinical remission | Infliximab | CT-P13 | 0.10  (-1.34 to 1.56) | 0.11  (-0.43 to 0.65) | 0.11  (-1.31 to 1.56) | N/A | N/A | 0.11  (-1.30 to 1.51) | N/A | 0.05  (-1.12 to 1.23) |
| Inducing clinical remission | Infliximab | Adalimumab | 1.73  (-0.80 to 5.48) | 1.78  (-0.32 to 5.41) | 1.72  (-0.75 to 5.39) | N/A | N/A | 1.73  (-0.83 to 5.31) | N/A | 0.77  (-1.00 to 3.22) |
| Inducing clinical remission | Infliximab | Adalimumab+azathioprine | 2.49  (-0.43 to 6.47) | 2.55  (0.31 to 6.20) | 2.48  (-0.39 to 6.39) | N/A | N/A | N/A | N/A | 0.99  (-1.08 to 3.70) |
| Inducing clinical remission | Infliximab | Certolizumab Pegol | 3.13  (0.43 to 6.99) | 3.19  (1.15 to 6.83) | 3.12  (0.46 to 6.86) | N/A | N/A | 3.13  (0.43 to 6.87) | N/A | 1.71  (-0.25 to 4.25) |
| Inducing clinical remission | Infliximab | Ustekinumab | 1.68  (-1.18 to 5.68) | 1.73  (-0.42 to 5.36) | 1.67  (-1.13 to 5.56) | N/A | N/A | 1.69  (-1.15 to 5.52) | N/A | 0.75  (-1.34 to 3.46) |
| Inducing clinical remission | Infliximab | Vedolizumab | 2.40  (-0.15 to 6.11) | 2.48  (0.37 to 6.10) | 2.41  (-0.14 to 6.01) | N/A | N/A | 2.39  (-0.22 to 5.99) | N/A | 1.26  (-0.56 to 3.64) |
| Inducing clinical remission | Infliximab | placebo | 3.42  (1.09 to 7.00) | 3.49  (1.51 to 7.10) | 3.42  (1.12 to 6.95) | N/A | N/A | 3.43  (1.05 to 6.87) | N/A | 1.92  (0.33 to 4.17) |
| Inducing clinical remission | Infliximab+azathioprine | CT-P13 | 0.22  (-1.81 to 2.25) | 0.23  (-0.46 to 0.92) | 0.24  (-1.76 to 2.27) | N/A | N/A | 0.23  (-1.74 to 2.19) | N/A | 0.10  (-1.56 to 1.76) |
| Inducing clinical remission | Infliximab+azathioprine | Adalimumab | 1.86  (-1.00 to 5.84) | 1.91  (-0.25 to 5.55) | 1.86  (-0.97 to 5.73) | N/A | N/A | 1.86  (-1.01 to 5.69) | N/A | 0.82  (-1.25 to 3.51) |
| Inducing clinical remission | Infliximab+azathioprine | Adalimumab+azathioprine | 2.61  (-0.63 to 6.82) | 2.67  (0.37 to 6.35) | 2.61  (-0.57 to 6.71) | N/A | N/A | N/A | N/A | 1.04  (-1.29 to 3.99) |
| Inducing clinical remission | Infliximab+azathioprine | Certolizumab Pegol | 3.25  (0.19 to 7.33) | 3.32  (1.22 to 6.97) | 3.26  (0.26 to 7.22) | N/A | N/A | 3.25  (0.25 to 7.20) | N/A | 1.77  (-0.51 to 4.56) |
| Inducing clinical remission | Infliximab+azathioprine | Ustekinumab | 1.81  (-1.37 to 6.01) | 1.86  (-0.35 to 5.49) | 1.80  (-1.33 to 5.91) | N/A | N/A | 1.81  (-1.31 to 5.87) | N/A | 0.80  (-1.58 to 3.75) |
| Inducing clinical remission | Infliximab+azathioprine | Vedolizumab | 2.52  (-0.39 to 6.46) | 2.60  (0.44 to 6.24) | 2.54  (-0.36 to 6.38) | N/A | N/A | 2.51  (-0.40 to 6.34) | N/A | 1.31  (-0.83 to 3.97) |
| Inducing clinical remission | Infliximab+azathioprine | placebo | 3.55  (0.84 to 7.40) | 3.62  (1.58 to 7.24) | 3.55  (0.88 to 7.30) | N/A | N/A | 3.55  (0.86 to 7.26) | N/A | 1.98  (0.03 to 4.50) |
| Inducing clinical remission | CT-P13 | Adalimumab | 1.64  (-1.25 to 5.64) | 1.68  (-0.50 to 5.34) | 1.62  (-1.23 to 5.50) | N/A | N/A | 1.64  (-1.23 to 5.47) | N/A | 0.72  (-1.36 to 3.44) |
| Inducing clinical remission | CT-P13 | Adalimumab+azathioprine | 2.39  (-0.85 to 6.62) | 2.44  (0.13 to 6.12) | 2.37  (-0.83 to 6.51) | N/A | N/A | N/A | N/A | 0.95  (-1.42 to 3.90) |
| Inducing clinical remission | CT-P13 | Certolizumab Pegol | 3.04  (-0.02 to 7.13) | 3.09  (0.97 to 6.76) | 3.03  (0.00 to 6.99) | N/A | N/A | 3.03  (0.01 to 7.00) | N/A | 1.67  (-0.61 to 4.47) |
| Inducing clinical remission | CT-P13 | Ustekinumab | 1.59  (-1.61 to 5.82) | 1.63  (-0.60 to 5.29) | 1.56  (-1.56 to 5.67) | N/A | N/A | 1.59  (-1.54 to 5.64) | N/A | 0.70  (-1.68 to 3.67) |
| Inducing clinical remission | CT-P13 | Vedolizumab | 2.31  (-0.62 to 6.27) | 2.38  (0.19 to 6.03) | 2.31  (-0.62 to 6.13) | N/A | N/A | 2.30  (-0.63 to 6.12) | N/A | 1.21  (-0.94 to 3.87) |
| Inducing clinical remission | CT-P13 | placebo | 3.33  (0.61 to 7.20) | 3.39  (1.33 to 7.04) | 3.32  (0.63 to 7.08) | N/A | N/A | 3.33  (0.63 to 7.05) | N/A | 1.88  (-0.09 to 4.42) |
| Inducing clinical remission | Adalimumab | Adalimumab+azathioprine | 0.74  (-0.76 to 2.22) | 0.74  (0.08 to 1.42) | 0.74  (-0.73 to 2.22) | N/A | N/A | N/A | N/A | 0.22  (-0.94 to 1.39) |
| Inducing clinical remission | Adalimumab | Certolizumab Pegol | 1.40  (-0.36 to 3.08) | 1.42  (0.71 to 2.14) | 1.41  (-0.37 to 3.05) | N/A | N/A | 1.40  (-0.33 to 3.02) | N/A | 0.96  (-0.52 to 2.30) |
| Inducing clinical remission | Adalimumab | Ustekinumab | -0.06  (-1.46 to 1.34) | -0.06  (-0.46 to 0.34) | -0.06  (-1.45 to 1.35) | N/A | N/A | -0.06  (-1.41 to 1.33) | N/A | -0.03  (-1.19 to 1.14) |
| Inducing clinical remission | Adalimumab | Vedolizumab | 0.67  (-0.79 to 2.02) | 0.70  (-0.16 to 1.56) | 0.67  (-0.79 to 2.01) | N/A | N/A | 0.67  (-0.77 to 1.99) | N/A | 0.49  (-0.70 to 1.56) |
| Inducing clinical remission | Adalimumab | placebo | 1.70  (0.69 to 2.66) | 1.72  (1.17 to 2.32) | 1.71  (0.68 to 2.65) | N/A | N/A | 1.70  (0.71 to 2.65) | N/A | 1.16  (0.32 to 1.92) |
| Inducing clinical remission | Adalimumab+azathioprine | Certolizumab Pegol | 0.66  (-1.66 to 2.90) | 0.68  (-0.31 to 1.66) | 0.68  (-1.65 to 2.86) | N/A | N/A | N/A | N/A | 0.74  (-1.18 to 2.49) |
| Inducing clinical remission | Adalimumab+azathioprine | Ustekinumab | -0.80  (-2.83 to 1.24) | -0.80  (-1.59 to -0.03) | -0.80  (-2.83 to 1.23) | N/A | N/A | N/A | N/A | -0.25  (-1.89 to 1.40) |
| Inducing clinical remission | Adalimumab+azathioprine | Vedolizumab | -0.07  (-2.16 to 1.91) | -0.04  (-1.14 to 1.05) | -0.06  (-2.17 to 1.89) | N/A | N/A | N/A | N/A | 0.27  (-1.41 to 1.80) |
| Inducing clinical remission | Adalimumab+azathioprine | placebo | 0.97  (-0.83 to 2.71) | 0.99  (0.10 to 1.88) | 0.97  (-0.85 to 2.69) | N/A | N/A | N/A | N/A | 0.95  (-0.52 to 2.28) |
| Inducing clinical remission | Certolizumab Pegol | Ustekinumab | -1.46  (-3.64 to 0.81) | -1.48  (-2.31 to -0.67) | -1.48  (-3.59 to 0.82) | N/A | N/A | -1.46  (-3.55 to 0.78) | N/A | -0.99  (-2.76 to 0.92) |
| Inducing clinical remission | Certolizumab Pegol | Vedolizumab | -0.72  (-2.48 to 0.97) | -0.71  (-1.50 to 0.05) | -0.73  (-2.46 to 0.97) | N/A | N/A | -0.74  (-2.41 to 0.91) | N/A | -0.47  (-1.89 to 0.94) |
| Inducing clinical remission | Certolizumab Pegol | placebo | 0.31  (-1.11 to 1.72) | 0.31  (-0.12 to 0.74) | 0.30  (-1.10 to 1.72) | N/A | N/A | 0.30  (-1.06 to 1.67) | N/A | 0.21  (-0.95 to 1.38) |
| Inducing clinical remission | Ustekinumab | Vedolizumab | 0.74  (-1.31 to 2.65) | 0.76  (-0.19 to 1.71) | 0.74  (-1.32 to 2.61) | N/A | N/A | 0.72  (-1.27 to 2.59) | N/A | 0.52  (-1.17 to 2.05) |
| Inducing clinical remission | Ustekinumab | placebo | 1.77  (0.00 to 3.45) | 1.79  (1.10 to 2.50) | 1.77  (0.01 to 3.40) | N/A | N/A | 1.76  (0.04 to 3.38) | N/A | 1.19  (-0.28 to 2.54) |
| Inducing clinical remission | Vedolizumab | placebo | 1.03  (0.05 to 2.06) | 1.02  (0.40 to 1.69) | 1.03  (0.07 to 2.06) | N/A | N/A | 1.04  (0.08 to 2.05) | N/A | 0.67  (-0.10 to 1.49) |
| Inducing CDAI-70 | Infliximab | Infliximab+azathioprine | -0.45  (-3.42 to 2.54) | -0.45  (-0.92 to 0.02) | -0.44  (-3.38 to 2.53) | -0.45  (-3.05 to 2.16) | N/A | -0.44  (-3.45 to 2.57) | N/A | -0.13  (-1.48 to 1.22) |
| Inducing CDAI-70 | Infliximab | CT-P13 | 0.24  (-2.75 to 3.27) | 0.25  (-0.35 to 0.84) | 0.25  (-2.72 to 3.25) | 0.25  (-2.38 to 2.87) | N/A | 0.26  (-2.76 to 3.30) | N/A | 0.07  (-1.27 to 1.42) |
| Inducing CDAI-70 | Infliximab | Adalimumab | 1.63  (-2.22 to 5.19) | 1.80  (0.33 to 3.52) | 1.61  (-2.26 to 5.08) | -0.44  (-38.14 to 40.02) | N/A | 1.65  (-2.26 to 5.17) | N/A | 0.70  (-1.05 to 2.44) |
| Inducing CDAI-70 | Infliximab | Adalimumab+azathioprine | 2.39  (-2.56 to 7.01) | 2.55  (0.81 to 4.51) | 2.37  (-2.62 to 6.92) | 0.31  (-37.48 to 40.83) | N/A | N/A | N/A | 0.81  (-1.44 to 2.97) |
| Inducing CDAI-70 | Infliximab | placebo | 3.23  (0.05 to 6.49) | 3.26  (1.86 to 4.94) | 3.23  (0.02 to 6.43) | 1.18  (-36.52 to 41.54) | N/A | 3.26  (-0.01 to 6.50) | N/A | 1.45  (-0.04 to 3.03) |
| Inducing CDAI-70 | Infliximab+azathioprine | CT-P13 | 0.69  (-3.54 to 4.93) | 0.70  (-0.05 to 1.46) | 0.69  (-3.51 to 4.88) | 0.70  (-3.06 to 4.38) | N/A | 0.71  (-3.55 to 4.95) | N/A | 0.20  (-1.69 to 2.12) |
| Inducing CDAI-70 | Infliximab+azathioprine | Adalimumab | 2.09  (-2.84 to 6.65) | 2.25  (0.71 to 4.03) | 2.07  (-2.84 to 6.53) | 0.03  (-37.76 to 40.55) | N/A | 2.11  (-2.90 to 6.65) | N/A | 0.84  (-1.38 to 2.99) |
| Inducing CDAI-70 | Infliximab+azathioprine | Adalimumab+azathioprine | 2.84  (-2.96 to 8.32) | 3.01  (1.20 to 5.02) | 2.82  (-3.01 to 8.17) | 0.76  (-37.01 to 41.34) | N/A | N/A | N/A | 0.95  (-1.67 to 3.48) |
| Inducing CDAI-70 | Infliximab+azathioprine | placebo | 3.68  (-0.69 to 8.09) | 3.71  (2.23 to 5.45) | 3.68  (-0.69 to 8.00) | 1.66  (-36.14 to 42.04) | N/A | 3.71  (-0.75 to 8.11) | N/A | 1.59  (-0.42 to 3.64) |
| Inducing CDAI-70 | CT-P13 | Adalimumab | 1.40  (-3.55 to 5.97) | 1.55  (-0.03 to 3.37) | 1.37  (-3.58 to 5.86) | -0.68  (-38.35 to 39.74) | N/A | 1.40  (-3.56 to 5.95) | N/A | 0.64  (-1.60 to 2.79) |
| Inducing CDAI-70 | CT-P13 | Adalimumab+azathioprine | 2.15  (-3.71 to 7.61) | 2.31  (0.47 to 4.36) | 2.12  (-3.73 to 7.50) | 0.04  (-37.71 to 40.59) | N/A | N/A | N/A | 0.74  (-1.89 to 3.27) |
| Inducing CDAI-70 | CT-P13 | placebo | 3.00  (-1.39 to 7.38) | 3.01  (1.49 to 4.80) | 2.98  (-1.42 to 7.32) | 0.95  (-36.74 to 41.33) | N/A | 3.00  (-1.43 to 7.42) | N/A | 1.39  (-0.63 to 3.43) |
| Inducing CDAI-70 | Adalimumab | Adalimumab+azathioprine | 0.75  (-2.28 to 3.82) | 0.74  (-0.15 to 1.72) | 0.75  (-2.30 to 3.79) | 0.74  (-1.96 to 3.45) | N/A | N/A | N/A | 0.10  (-1.25 to 1.46) |
| Inducing CDAI-70 | Adalimumab | placebo | 1.59  (-0.01 to 3.60) | 1.46  (1.03 to 1.90) | 1.59  (0.02 to 3.59) | 1.58  (0.16 to 3.34) | N/A | 1.59  (-0.01 to 3.62) | N/A | 0.74  (-0.02 to 1.63) |
| Inducing CDAI-70 | Adalimumab+azathioprine | placebo | 0.85  (-2.50 to 4.58) | 0.72  (-0.35 to 1.71) | 0.85  (-2.47 to 4.57) | 0.85  (-2.14 to 4.13) | N/A | N/A | N/A | 0.64  (-0.88 to 2.28) |
| Inducing CDAI-100 | Adalimumab | Certolizumab Pegol | 0.65  (-0.11 to 1.49) | 0.63  (0.10 to 1.17) | 0.65  (-0.12 to 1.46) | N/A | N/A | N/A | N/A | 0.33  (-0.13 to 0.83) |
| Inducing CDAI-100 | Adalimumab | Ustekinumab | -0.02  (-0.60 to 0.59) | -0.04  (-0.41 to 0.34) | -0.03  (-0.60 to 0.57) | N/A | N/A | N/A | N/A | -0.01  (-0.32 to 0.34) |
| Inducing CDAI-100 | Adalimumab | Vedolizumab | 0.46  (-0.40 to 1.34) | 0.45  (-0.22 to 1.11) | 0.45  (-0.39 to 1.32) | N/A | N/A | N/A | N/A | 0.24  (-0.31 to 0.79) |
| Inducing CDAI-100 | Adalimumab | placebo | 1.03  (0.43 to 1.69) | 1.01  (0.55 to 1.47) | 1.02  (0.43 to 1.68) | N/A | N/A | N/A | N/A | 0.56  (0.21 to 0.95) |
| Inducing CDAI-100 | Certolizumab Pegol | Ustekinumab | -0.67  (-1.49 to 0.09) | -0.67  (-1.17 to -0.18) | -0.68  (-1.47 to 0.09) | N/A | N/A | N/A | N/A | -0.34  (-0.82 to 0.14) |
| Inducing CDAI-100 | Certolizumab Pegol | Vedolizumab | -0.20  (-0.99 to 0.56) | -0.18  (-0.74 to 0.36) | -0.20  (-0.97 to 0.55) | N/A | N/A | N/A | N/A | -0.10  (-0.59 to 0.39) |
| Inducing CDAI-100 | Certolizumab Pegol | placebo | 0.38  (-0.12 to 0.87) | 0.38  (0.10 to 0.66) | 0.37  (-0.11 to 0.86) | N/A | N/A | N/A | N/A | 0.23  (-0.07 to 0.54) |
| Inducing CDAI-100 | Ustekinumab | Vedolizumab | 0.48  (-0.38 to 1.33) | 0.49  (-0.15 to 1.12) | 0.48  (-0.36 to 1.33) | N/A | N/A | N/A | N/A | 0.24  (-0.30 to 0.78) |
| Inducing CDAI-100 | Ustekinumab | placebo | 1.05  (0.46 to 1.68) | 1.05  (0.64 to 1.47) | 1.05  (0.46 to 1.67) | N/A | N/A | N/A | N/A | 0.57  (0.21 to 0.95) |
| Inducing CDAI-100 | Vedolizumab | placebo | 0.57  (-0.01 to 1.19) | 0.56  (0.09 to 1.05) | 0.57  (-0.01 to 1.18) | N/A | N/A | N/A | N/A | 0.33  (-0.05 to 0.74) |
| Maintaining clinical remission | IFX-5 | IFX-10 | -0.38  (-1.78 to 1.10) | -0.41  (-0.96 to 0.14) | -0.38  (-1.79 to 1.11) | N/A | -0.39  (-1.83 to 1.15) | -0.46  (-2.20 to 1.26) | N/A | -0.23  (-1.37 to 0.96) |
| Maintaining clinical remission | IFX-5 | Infliximab+azathioprine | -0.39  (-1.93 to 1.14) | -0.39  (-1.00 to 0.21) | -0.39  (-1.93 to 1.16) | N/A | N/A | -0.39  (-2.12 to 1.36) | N/A | -0.11  (-1.30 to 1.08) |
| Maintaining clinical remission | IFX-5 | CT-P13 | 0.08  (-1.43 to 1.61) | 0.08  (-0.46 to 0.61) | 0.08  (-1.44 to 1.61) | N/A | 0.08  (-1.49 to 1.66) | 0.08  (-1.64 to 1.80) | N/A | 0.03  (-1.16 to 1.22) |
| Maintaining clinical remission | IFX-5 | Adalimumab | -0.33  (-2.30 to 1.55) | -0.30  (-1.15 to 0.55) | -0.34  (-2.32 to 1.55) | N/A | -0.73  (-2.97 to 1.47) | -0.24  (-2.55 to 2.01) | N/A | -0.12  (-1.75 to 1.36) |
| Maintaining clinical remission | IFX-5 | Adalimumab+azathioprine | -0.15  (-2.66 to 2.26) | -0.13  (-1.16 to 0.92) | -0.16  (-2.69 to 2.26) | N/A | N/A | N/A | N/A | -0.05  (-2.08 to 1.84) |
| Maintaining clinical remission | IFX-5 | Ustekinumab | -0.27  (-2.19 to 1.70) | -0.34  (-1.19 to 0.52) | -0.29  (-2.20 to 1.71) | N/A | 0.12  (-2.12 to 2.34) | -0.16  (-2.41 to 2.18) | N/A | 0.02  (-1.53 to 1.56) |
| Maintaining clinical remission | IFX-5 | Vedolizumab | -0.01  (-2.00 to 2.00) | -0.02  (-0.94 to 0.89) | -0.01  (-1.99 to 2.01) | N/A | -0.03  (-2.03 to 2.06) | 0.10  (-2.19 to 2.46) | N/A | 0.20  (-1.27 to 1.81) |
| Maintaining clinical remission | IFX-5 | Vedolizumab SC | 0.59  (-1.57 to 2.68) | 0.60  (-0.29 to 1.50) | 0.58  (-1.56 to 2.71) | N/A | 0.57  (-1.63 to 2.77) | 0.69  (-1.80 to 3.18) | N/A | 0.46  (-1.24 to 2.15) |
| Maintaining clinical remission | IFX-5 | placebo | 0.83  (-0.66 to 2.25) | 0.83  (0.21 to 1.48) | 0.82  (-0.67 to 2.25) | N/A | 0.81  (-0.71 to 2.30) | 0.93  (-0.87 to 2.69) | N/A | 0.57  (-0.62 to 1.75) |
| Maintaining clinical remission | IFX-10 | Infliximab+azathioprine | -0.01  (-2.17 to 2.05) | 0.02  (-0.80 to 0.84) | -0.01  (-2.16 to 2.08) | N/A | N/A | 0.06  (-2.37 to 2.54) | N/A | 0.12  (-1.58 to 1.76) |
| Maintaining clinical remission | IFX-10 | CT-P13 | 0.45  (-1.66 to 2.50) | 0.48  (-0.28 to 1.25) | 0.46  (-1.65 to 2.54) | N/A | 0.47  (-1.73 to 2.58) | 0.54  (-1.90 to 2.98) | N/A | 0.26  (-1.44 to 1.90) |
| Maintaining clinical remission | IFX-10 | Adalimumab | 0.04  (-1.75 to 1.68) | 0.11  (-0.69 to 0.91) | 0.04  (-1.76 to 1.69) | N/A | -0.35  (-2.41 to 1.61) | 0.23  (-2.07 to 2.48) | N/A | 0.10  (-1.37 to 1.38) |
| Maintaining clinical remission | IFX-10 | Adalimumab+azathioprine | 0.22  (-2.14 to 2.45) | 0.28  (-0.73 to 1.29) | 0.22  (-2.17 to 2.45) | N/A | N/A | N/A | N/A | 0.18  (-1.74 to 1.89) |
| Maintaining clinical remission | IFX-10 | Ustekinumab | 0.11  (-1.62 to 1.83) | 0.07  (-0.74 to 0.88) | 0.09  (-1.64 to 1.85) | N/A | 0.50  (-1.57 to 2.49) | 0.30  (-1.93 to 2.64) | N/A | 0.25  (-1.14 to 1.56) |
| Maintaining clinical remission | IFX-10 | Vedolizumab | 0.37  (-1.45 to 2.13) | 0.39  (-0.48 to 1.26) | 0.36  (-1.44 to 2.14) | N/A | 0.35  (-1.46 to 2.19) | 0.57  (-1.71 to 2.92) | N/A | 0.43  (-0.89 to 1.83) |
| Maintaining clinical remission | IFX-10 | Vedolizumab SC | 0.96  (-1.03 to 2.84) | 1.01  (0.17 to 1.86) | 0.95  (-1.03 to 2.86) | N/A | 0.96  (-1.07 to 2.91) | 1.16  (-1.31 to 3.64) | N/A | 0.68  (-0.88 to 2.19) |
| Maintaining clinical remission | IFX-10 | placebo | 1.20  (-0.02 to 2.31) | 1.24  (0.69 to 1.83) | 1.19  (-0.03 to 2.31) | N/A | 1.19  (-0.04 to 2.33) | 1.40  (-0.37 to 3.15) | N/A | 0.79  (-0.15 to 1.71) |
| Maintaining clinical remission | Infliximab+azathioprine | CT-P13 | 0.46  (-1.69 to 2.64) | 0.47  (-0.34 to 1.27) | 0.47  (-1.69 to 2.63) | N/A | N/A | 0.48  (-1.98 to 2.92) | N/A | 0.14  (-1.55 to 1.82) |
| Maintaining clinical remission | Infliximab+azathioprine | Adalimumab | 0.06  (-2.44 to 2.49) | 0.09  (-0.94 to 1.14) | 0.05  (-2.46 to 2.47) | N/A | N/A | 0.16  (-2.75 to 3.00) | N/A | -0.01  (-2.04 to 1.86) |
| Maintaining clinical remission | Infliximab+azathioprine | Adalimumab+azathioprine | 0.23  (-2.69 to 3.11) | 0.26  (-0.94 to 1.48) | 0.23  (-2.73 to 3.09) | N/A | N/A | N/A | N/A | 0.07  (-2.31 to 2.29) |
| Maintaining clinical remission | Infliximab+azathioprine | Ustekinumab | 0.12  (-2.31 to 2.63) | 0.05  (-0.99 to 1.10) | 0.10  (-2.33 to 2.62) | N/A | N/A | 0.23  (-2.61 to 3.15) | N/A | 0.14  (-1.83 to 2.07) |
| Maintaining clinical remission | Infliximab+azathioprine | Vedolizumab | 0.38  (-2.10 to 2.92) | 0.37  (-0.72 to 1.46) | 0.37  (-2.11 to 2.92) | N/A | N/A | 0.50  (-2.38 to 3.43) | N/A | 0.31  (-1.56 to 2.33) |
| Maintaining clinical remission | Infliximab+azathioprine | Vedolizumab SC | 0.98  (-1.66 to 3.58) | 1.00  (-0.09 to 2.08) | 0.97  (-1.68 to 3.58) | N/A | N/A | 1.09  (-1.96 to 4.12) | N/A | 0.57  (-1.51 to 2.65) |
| Maintaining clinical remission | Infliximab+azathioprine | placebo | 1.21  (-0.91 to 3.31) | 1.23  (0.36 to 2.11) | 1.20  (-0.93 to 3.29) | N/A | N/A | 1.33  (-1.19 to 3.80) | N/A | 0.69  (-1.01 to 2.35) |
| Maintaining clinical remission | CT-P13 | Adalimumab | -0.41  (-2.90 to 1.99) | -0.38  (-1.38 to 0.63) | -0.42  (-2.92 to 1.98) | N/A | -0.81  (-3.55 to 1.89) | -0.31  (-3.20 to 2.52) | N/A | -0.15  (-2.20 to 1.73) |
| Maintaining clinical remission | CT-P13 | Adalimumab+azathioprine | -0.23  (-3.15 to 2.60) | -0.21  (-1.38 to 0.97) | -0.24  (-3.21 to 2.60) | N/A | N/A | N/A | N/A | -0.07  (-2.46 to 2.14) |
| Maintaining clinical remission | CT-P13 | Ustekinumab | -0.35  (-2.76 to 2.12) | -0.41  (-1.42 to 0.59) | -0.37  (-2.81 to 2.13) | N/A | 0.04  (-2.71 to 2.75) | -0.25  (-3.04 to 2.68) | N/A | 0.00  (-1.97 to 1.94) |
| Maintaining clinical remission | CT-P13 | Vedolizumab | -0.09  (-2.58 to 2.43) | -0.10  (-1.16 to 0.96) | -0.10  (-2.58 to 2.43) | N/A | -0.11  (-2.65 to 2.53) | 0.02  (-2.85 to 2.97) | N/A | 0.18  (-1.71 to 2.19) |
| Maintaining clinical remission | CT-P13 | Vedolizumab SC | 0.52  (-2.12 to 3.08) | 0.53  (-0.51 to 1.56) | 0.49  (-2.15 to 3.10) | N/A | 0.49  (-2.22 to 3.20) | 0.61  (-2.40 to 3.64) | N/A | 0.43  (-1.65 to 2.49) |
| Maintaining clinical remission | CT-P13 | placebo | 0.75  (-1.38 to 2.81) | 0.76  (-0.06 to 1.59) | 0.73  (-1.40 to 2.80) | N/A | 0.73  (-1.46 to 2.89) | 0.85  (-1.65 to 3.31) | N/A | 0.54  (-1.15 to 2.22) |
| Maintaining clinical remission | Adalimumab | Adalimumab+azathioprine | 0.18  (-1.34 to 1.71) | 0.17  (-0.44 to 0.79) | 0.18  (-1.36 to 1.72) | N/A | N/A | N/A | N/A | 0.07  (-1.12 to 1.28) |
| Maintaining clinical remission | Adalimumab | Ustekinumab | 0.06  (-1.10 to 1.39) | -0.04  (-0.43 to 0.35) | 0.05  (-1.11 to 1.38) | N/A | 0.86  (-1.46 to 3.17) | 0.06  (-1.25 to 1.56) | N/A | 0.14  (-0.78 to 1.19) |
| Maintaining clinical remission | Adalimumab | Vedolizumab | 0.32  (-1.50 to 2.25) | 0.28  (-0.59 to 1.15) | 0.32  (-1.49 to 2.27) | N/A | 0.72  (-1.38 to 2.91) | 0.34  (-1.68 to 2.49) | N/A | 0.33  (-0.96 to 1.88) |
| Maintaining clinical remission | Adalimumab | Vedolizumab SC | 0.93  (-1.07 to 2.93) | 0.90  (0.06 to 1.76) | 0.91  (-1.07 to 2.95) | N/A | 1.32  (-0.96 to 3.59) | 0.93  (-1.31 to 3.20) | N/A | 0.58  (-0.94 to 2.23) |
| Maintaining clinical remission | Adalimumab | placebo | 1.16  (-0.11 to 2.45) | 1.13  (0.58 to 1.71) | 1.15  (-0.10 to 2.45) | N/A | 1.54  (-0.08 to 3.18) | 1.16  (-0.26 to 2.61) | N/A | 0.69  (-0.25 to 1.78) |
| Maintaining clinical remission | Adalimumab+azathioprine | Ustekinumab | -0.12  (-2.02 to 1.93) | -0.21  (-0.94 to 0.51) | -0.13  (-2.01 to 1.93) | N/A | N/A | N/A | N/A | 0.06  (-1.43 to 1.68) |
| Maintaining clinical remission | Adalimumab+azathioprine | Vedolizumab | 0.15  (-2.22 to 2.61) | 0.11  (-0.96 to 1.17) | 0.14  (-2.20 to 2.62) | N/A | N/A | N/A | N/A | 0.25  (-1.47 to 2.25) |
| Maintaining clinical remission | Adalimumab+azathioprine | Vedolizumab SC | 0.75  (-1.77 to 3.27) | 0.73  (-0.31 to 1.78) | 0.74  (-1.76 to 3.30) | N/A | N/A | N/A | N/A | 0.50  (-1.41 to 2.57) |
| Maintaining clinical remission | Adalimumab+azathioprine | placebo | 0.98  (-1.00 to 2.98) | 0.96  (0.13 to 1.80) | 0.97  (-1.01 to 3.00) | N/A | N/A | N/A | N/A | 0.61  (-0.89 to 2.27) |
| Maintaining clinical remission | Ustekinumab | Vedolizumab | 0.26  (-1.64 to 2.13) | 0.32  (-0.57 to 1.20) | 0.27  (-1.63 to 2.14) | N/A | -0.15  (-2.24 to 2.06) | 0.27  (-1.83 to 2.34) | N/A | 0.18  (-1.14 to 1.66) |
| Maintaining clinical remission | Ustekinumab | Vedolizumab SC | 0.86  (-1.22 to 2.81) | 0.94  (0.08 to 1.81) | 0.86  (-1.21 to 2.83) | N/A | 0.45  (-1.83 to 2.75) | 0.86  (-1.46 to 3.06) | N/A | 0.43  (-1.13 to 2.02) |
| Maintaining clinical remission | Ustekinumab | placebo | 1.09  (-0.25 to 2.33) | 1.17  (0.60 to 1.76) | 1.10  (-0.27 to 2.33) | N/A | 0.69  (-0.96 to 2.34) | 1.09  (-0.43 to 2.48) | N/A | 0.54  (-0.44 to 1.55) |
| Maintaining clinical remission | Vedolizumab | Vedolizumab SC | 0.61  (-1.50 to 2.61) | 0.62  (-0.28 to 1.54) | 0.59  (-1.51 to 2.63) | N/A | 0.60  (-1.57 to 2.67) | 0.58  (-1.75 to 2.85) | N/A | 0.25  (-1.39 to 1.74) |
| Maintaining clinical remission | Vedolizumab | placebo | 0.84  (-0.56 to 2.16) | 0.85  (0.21 to 1.53) | 0.83  (-0.59 to 2.15) | N/A | 0.83  (-0.61 to 2.17) | 0.82  (-0.73 to 2.28) | N/A | 0.36  (-0.70 to 1.29) |
| Maintaining clinical remission | Vedolizumab SC | placebo | 0.23  (-1.30 to 1.78) | 0.23  (-0.39 to 0.87) | 0.24  (-1.32 to 1.78) | N/A | 0.24  (-1.37 to 1.83) | 0.24  (-1.51 to 1.96) | N/A | 0.11  (-1.10 to 1.33) |
| **TNF antagonists-experienced CD patients** | | | | | | | | | | |
| Inducing clinical remission | Adalimumab | Ustekinumab | 0.75  (-0.68 to 2.37) | 0.66  (-0.13 to 1.49) | 0.75  (-0.70 to 2.38) | 1.16  (-0.74 to 3.15) | N/A | N/A | N/A | 0.49  (-0.77 to 1.80) |
| Inducing clinical remission | Adalimumab | Risankizumab | 0.18  (-2.09 to 2.36) | 0.15  (-1.60 to 1.69) | 0.16  (-2.13 to 2.34) | 0.19  (-2.14 to 2.44) | N/A | N/A | N/A | 0.21  (-1.66 to 1.99) |
| Inducing clinical remission | Adalimumab | Vedolizumab | 1.08  (-0.34 to 2.64) | 1.04  (0.19 to 1.91) | 1.08  (-0.37 to 2.63) | 1.08  (-0.42 to 2.70) | N/A | N/A | N/A | 0.84  (-0.40 to 2.10) |
| Inducing clinical remission | Adalimumab | placebo | 1.32  (0.19 to 2.57) | 1.29  (0.64 to 2.00) | 1.32  (0.17 to 2.57) | 1.32  (0.14 to 2.63) | N/A | N/A | N/A | 0.94  (-0.05 to 1.93) |
| Inducing clinical remission | Ustekinumab | Risankizumab | -0.58  (-2.80 to 1.41) | -0.52  (-2.19 to 0.92) | -0.60  (-2.82 to 1.41) | -0.98  (-3.50 to 1.38) | N/A | N/A | N/A | -0.28  (-2.10 to 1.37) |
| Inducing clinical remission | Ustekinumab | Vedolizumab | 0.34  (-1.00 to 1.61) | 0.38  (-0.30 to 1.06) | 0.33  (-1.01 to 1.62) | -0.08  (-1.84 to 1.70) | N/A | N/A | N/A | 0.35  (-0.79 to 1.45) |
| Inducing clinical remission | Ustekinumab | placebo | 0.57  (-0.43 to 1.49) | 0.63  (0.19 to 1.07) | 0.57  (-0.44 to 1.49) | 0.17  (-1.33 to 1.65) | N/A | N/A | N/A | 0.45  (-0.41 to 1.23) |
| Inducing clinical remission | Risankizumab | Vedolizumab | 0.91  (-1.06 to 3.10) | 0.90  (-0.57 to 2.61) | 0.93  (-1.07 to 3.10) | 0.89  (-1.15 to 3.15) | N/A | N/A | N/A | 0.62  (-1.03 to 2.40) |
| Inducing clinical remission | Risankizumab | placebo | 1.15  (-0.63 to 3.13) | 1.14  (-0.22 to 2.77) | 1.16  (-0.63 to 3.15) | 1.13  (-0.69 to 3.18) | N/A | N/A | N/A | 0.72  (-0.75 to 2.34) |
| Inducing clinical remission | Vedolizumab | placebo | 0.23  (-0.66 to 1.12) | 0.25  (-0.27 to 0.77) | 0.24  (-0.66 to 1.12) | 0.24  (-0.71 to 1.17) | N/A | N/A | N/A | 0.10  (-0.68 to 0.85) |
| Inducing CDAI-70 | Adalimumab | Ustekinumab | 0.11  (-0.91 to 1.21) | 0.11  (-0.41 to 0.63) | 0.11  (-0.93 to 1.22) | N/A | N/A | N/A | N/A | 0.00  (-0.57 to 0.56) |
| Inducing CDAI-70 | Adalimumab | placebo | 0.79  (0.02 to 1.65) | 0.77  (0.34 to 1.20) | 0.79  (0.00 to 1.66) | N/A | N/A | N/A | N/A | 0.41  (-0.01 to 0.84) |
| Inducing CDAI-70 | Ustekinumab | placebo | 0.67  (0.00 to 1.38) | 0.66  (0.36 to 0.96) | 0.67  (0.00 to 1.38) | N/A | N/A | N/A | N/A | 0.41  (0.05 to 0.79) |
| Inducing CDAI-100 | Adalimumab | Certolizumab Pegol | 0.58  (-1.50 to 2.84) | 0.44  (-0.41 to 1.29) | 0.59  (-1.47 to 2.84) | 0.52  (-1.11 to 2.38) | N/A | N/A | N/A | 0.32  (-0.88 to 1.60) |
| Inducing CDAI-100 | Adalimumab | Ustekinumab | -0.61  (-2.12 to 1.15) | -0.88  (-1.43 to -0.33) | -0.60  (-2.10 to 1.15) | -0.27  (-1.62 to 1.24) | N/A | N/A | N/A | -0.39  (-1.23 to 0.60) |
| Inducing CDAI-100 | Adalimumab | Risankizumab | -0.12  (-2.39 to 2.24) | -0.27  (-1.52 to 0.89) | -0.13  (-2.36 to 2.25) | -0.21  (-2.05 to 1.79) | N/A | N/A | N/A | -0.13  (-1.51 to 1.26) |
| Inducing CDAI-100 | Adalimumab | Vedolizumab | 0.27  (-1.29 to 2.02) | 0.09  (-0.51 to 0.69) | 0.28  (-1.27 to 2.02) | 0.19  (-1.01 to 1.68) | N/A | N/A | N/A | 0.10  (-0.78 to 1.10) |
| Inducing CDAI-100 | Adalimumab | placebo | 0.84  (-0.38 to 2.26) | 0.70  (0.25 to 1.17) | 0.85  (-0.36 to 2.27) | 0.78  (-0.17 to 1.98) | N/A | N/A | N/A | 0.48  (-0.22 to 1.27) |
| Inducing CDAI-100 | Certolizumab Pegol | Ustekinumab | -1.18  (-3.13 to 0.81) | -1.32  (-2.08 to -0.54) | -1.19  (-3.12 to 0.79) | -0.79  (-2.50 to 0.84) | N/A | N/A | N/A | -0.71  (-1.80 to 0.44) |
| Inducing CDAI-100 | Certolizumab Pegol | Risankizumab | -0.71  (-3.30 to 1.83) | -0.71  (-2.08 to 0.58) | -0.72  (-3.27 to 1.80) | -0.73  (-2.85 to 1.37) | N/A | N/A | N/A | -0.45  (-2.01 to 1.05) |
| Inducing CDAI-100 | Certolizumab Pegol | Vedolizumab | -0.31  (-2.29 to 1.67) | -0.35  (-1.15 to 0.46) | -0.31  (-2.29 to 1.67) | -0.32  (-1.89 to 1.30) | N/A | N/A | N/A | -0.22  (-1.35 to 0.94) |
| Inducing CDAI-100 | Certolizumab Pegol | placebo | 0.27  (-1.45 to 1.97) | 0.27  (-0.44 to 0.99) | 0.26  (-1.45 to 1.97) | 0.26  (-1.10 to 1.64) | N/A | N/A | N/A | 0.16  (-0.83 to 1.16) |
| Inducing CDAI-100 | Ustekinumab | Risankizumab | 0.48  (-1.71 to 2.56) | 0.61  (-0.60 to 1.72) | 0.47  (-1.69 to 2.56) | 0.06  (-1.77 to 1.95) | N/A | N/A | N/A | 0.25  (-1.08 to 1.49) |
| Inducing CDAI-100 | Ustekinumab | Vedolizumab | 0.87  (-0.53 to 2.23) | 0.97  (0.49 to 1.45) | 0.88  (-0.52 to 2.24) | 0.47  (-0.71 to 1.78) | N/A | N/A | N/A | 0.49  (-0.30 to 1.26) |
| Inducing CDAI-100 | Ustekinumab | placebo | 1.45  (0.47 to 2.39) | 1.58  (1.29 to 1.88) | 1.45  (0.48 to 2.38) | 1.05  (0.15 to 2.05) | N/A | N/A | N/A | 0.87  (0.32 to 1.38) |
| Inducing CDAI-100 | Risankizumab | Vedolizumab | 0.40  (-1.73 to 2.59) | 0.36  (-0.77 to 1.58) | 0.41  (-1.71 to 2.56) | 0.41  (-1.35 to 2.23) | N/A | N/A | N/A | 0.24  (-1.03 to 1.58) |
| Inducing CDAI-100 | Risankizumab | placebo | 0.97  (-0.91 to 2.91) | 0.98  (-0.09 to 2.14) | 0.98  (-0.89 to 2.91) | 0.99  (-0.58 to 2.61) | N/A | N/A | N/A | 0.61  (-0.52 to 1.82) |
| Inducing CDAI-100 | Vedolizumab | placebo | 0.57  (-0.42 to 1.57) | 0.61  (0.24 to 1.00) | 0.57  (-0.43 to 1.57) | 0.59  (-0.23 to 1.37) | N/A | N/A | N/A | 0.38  (-0.21 to 0.95) |
| Maintaining clinical remission | Adalimumab | Ustekinumab | 0.71  (-1.20 to 2.65) | 0.71  (-0.25 to 1.76) | 0.71  (-1.18 to 2.64) | N/A | N/A | N/A | N/A | 0.64  (-0.86 to 2.14) |
| Maintaining clinical remission | Adalimumab | Vedolizumab | 0.44  (-1.78 to 2.66) | 0.43  (-0.72 to 1.61) | 0.43  (-1.78 to 2.65) | N/A | N/A | N/A | N/A | 0.34  (-1.40 to 2.08) |
| Maintaining clinical remission | Adalimumab | Vedolizumab SC | 0.49  (-1.70 to 2.69) | 0.49  (-0.55 to 1.60) | 0.49  (-1.70 to 2.67) | N/A | N/A | N/A | N/A | 0.50  (-1.19 to 2.21) |
| Maintaining clinical remission | Adalimumab | placebo | 1.41  (-0.17 to 3.03) | 1.41  (0.59 to 2.35) | 1.41  (-0.16 to 3.01) | N/A | N/A | N/A | N/A | 1.06  (-0.18 to 2.33) |
| Maintaining clinical remission | Ustekinumab | Vedolizumab | -0.27  (-2.17 to 1.59) | -0.28  (-1.21 to 0.59) | -0.28  (-2.18 to 1.57) | N/A | N/A | N/A | N/A | -0.30  (-1.76 to 1.17) |
| Maintaining clinical remission | Ustekinumab | Vedolizumab SC | -0.22  (-2.07 to 1.61) | -0.22  (-1.01 to 0.55) | -0.23  (-2.06 to 1.61) | N/A | N/A | N/A | N/A | -0.14  (-1.54 to 1.28) |
| Maintaining clinical remission | Ustekinumab | placebo | 0.71  (-0.37 to 1.77) | 0.71  (0.23 to 1.19) | 0.71  (-0.36 to 1.78) | N/A | N/A | N/A | N/A | 0.42  (-0.39 to 1.25) |
| Maintaining clinical remission | Vedolizumab | Vedolizumab SC | 0.05  (-2.09 to 2.20) | 0.06  (-0.90 to 1.06) | 0.05  (-2.08 to 2.21) | N/A | N/A | N/A | N/A | 0.16  (-1.51 to 1.82) |
| Maintaining clinical remission | Vedolizumab | placebo | 0.97  (-0.55 to 2.53) | 0.98  (0.26 to 1.79) | 0.98  (-0.53 to 2.55) | N/A | N/A | N/A | N/A | 0.72  (-0.49 to 1.94) |
| Maintaining clinical remission | Vedolizumab SC | placebo | 0.92  (-0.56 to 2.42) | 0.92  (0.33 to 1.55) | 0.93  (-0.56 to 2.42) | N/A | N/A | N/A | N/A | 0.56  (-0.59 to 1.70) |
| Abbreviation: IFX-5/10-5/10 mg/kg Infliximab maintenance regimen, RZB-180/360-180/360 mg Risankizumab maintenance regimen, Vedolizumab SC-subcutaneous injections formulation of Vedolizumab. | | | | | | | | | | |
