# Supplementary material for: Systematic review and bayesian network meta-analysis: comparative efficacy and safety of six commonly used biologic therapies for moderate-to-severe Crohn’s disease
Source: Front Pharmacol. 2025 Jan 9;15:1475222. doi: 10.3389/fphar.2024.1475222 (PMC11794990; doi:10.3389/fphar.2024.1475222)
Supplement: Supplementary file 2 [file DataSheet1.docx]

**SUPPLEMENTARY FILE 1**

**Table of Contents**

[**Table of Contents** 1](#_Toc183973811)

[**Supplementary Table 1.** Search strategy 7](#_Toc183973812)

[**Supplementary Information.** detailed PICOS. 11](#_Toc183973813)

[**Supplementary Figure 1.** Traffic light plot of the risk of bias assessment. 12](#_Toc183973814)

[**Supplementary Figure 2A.** Network plots of the available direct comparisons of (a) induction of clinical remission, (b) induction of CDAI-70, (c) induction of CDAI-100, and (d) risk of adverse events in induction therapy, (e) risk of serious adverse events in induction therapy, (f) risk of serious infections in induction therapy, in patients with moderate-to-severe Crohn’s disease in included studies. The size of the nodes and the thickness of the edges are weighted according to the number of studies evaluating each treatment and of directly comparative arms, respectively. 13](#_Toc183973815)

[**Supplementary Figure 2B.** Network plots of the available direct comparisons of (a) maintenance of clinical remission, (b) maintenance of CDAI-70, (c) maintenance of CDAI-100, and (d) risk of adverse events in maintenance therapy, (e) risk of serious adverse events in maintenance therapy, (f) risk of serious infections in maintenance therapy, in patients with moderate-to-severe Crohn’s disease in included studies. The size of the nodes and the thickness of the edges are weighted according to the number of studies evaluating each treatment and of directly comparative arms, respectively. 14](#_Toc183973816)

[**Supplementary Figure 2C.** Network plots of the available direct comparisons of (a) induction of clinical remission, (b) induction of CDAI-70, (c) induction of CDAI-100, in tumor necrosis factor antagonist-naïve patients with moderate-to-severe Crohn’s disease in included studies. The size of the nodes and the thickness of the edges are weighted according to the number of studies evaluating each treatment and of directly comparative arms, respectively. 16](#_Toc183973817)

[**Supplementary Figure 2D.** Network plots of the available direct comparisons of (a) induction of clinical remission, (b) induction of CDAI-70, (c) induction of CDAI-100, in tumor necrosis factor antagonist-experienced patients with moderate-to-severe Crohn’s disease in included studies. The size of the nodes and the thickness of the edges are weighted according to the number of studies evaluating each treatment and of directly comparative arms, respectively. 17](#_Toc183973818)

[**Supplementary Figure 2E.** Network plots of the available direct comparisons of maintenance of clinical remission in (a) tumor necrosis factor antagonist-naïve patients and (b) tumor necrosis factor antagonist-experienced patients, with moderate-to-severe Crohn’s disease in included studies. The size of the nodes and the thickness of the edges are weighted according to the number of studies evaluating each treatment and of directly comparative arms, respectively. 18](#_Toc183973819)

[**Supplementary Figure 3.** Forest plots of direct comparisons of (A) induction of clinical remission, (B) induction of CDAI-70, (C) induction of CDAI-100, and (D) risk of adverse events in induction therapy, (E) risk of serious adverse events in induction therapy, (F) risk of serious infections in induction therapy, between included biologic agents in overall patients with moderate-to-severe Crohn’s disease. 19](#_Toc183973820)

[**Supplementary Figure 4.** Forest plots of direct comparisons of (A) maintenance of clinical remission, (B) maintenance of CDAI-70, (C) maintenance of CDAI-100, and (D) risk of adverse events in maintenance therapy, (E) risk of serious adverse events in maintenance therapy, (F) risk of serious infections in maintenance therapy, between included biologic agents in overall patients with moderate-to-severe Crohn’s disease. 25](#_Toc183973821)

[**Supplementary Figure 5.** Forest plots of direct comparisons of (A) induction of clinical remission, (B) induction of CDAI-70, (C) induction of CDAI-100, between included biologic agents in tumor necrosis factor antagonist-naïve patients with moderate-to-severe Crohn’s disease. 31](#_Toc183973822)

[**Supplementary Figure 6.** Forest plots of direct comparisons of (A) induction of clinical remission, (B) induction of CDAI-70, (C) induction of CDAI-100, between included biologic agents in tumor necrosis factor antagonist-experienced patients with moderate-to-severe Crohn’s disease. 33](#_Toc183973823)

[**Supplementary Figure 7.** Forest plots of direct comparisons of maintenance for clinical remission between included biologic agents (A) in tumor necrosis factor antagonist-naïve patients, (B) in tumor necrosis factor antagonist-experienced patients, with moderate-to-severe Crohn’s disease. 35](#_Toc183973824)

[**Supplementary Figure 8.** Funnel plots of assessments of small study and publication bias in evaluation of (A) induction of clinical remission, (C) induction of CDAI-70, (E) induction of CDAI-100, in overall patients with moderate-to-severe Crohn’s disease, and (B) (D) (F) are their respectively plots moderated by including outcomes timepoint as a moderator. 36](#_Toc183973825)

[**Supplementary Figure 9.** Funnel plots of assessments of small study and publication bias in evaluation of (A) risk of adverse events in induction therapy, (C) risk of serious adverse events in induction therapy, (E) risk of serious infections in induction therapy, in overall patients with moderate-to-severe Crohn’s disease, and (B) (D) (F) are their respectively plots moderated by including outcomes timepoint as a moderator. 37](#_Toc183973826)

[**Supplementary Figure 10.** Funnel plots of assessments of small study and publication bias in evaluation of (A) maintenance of clinical remission, (C) maintenance of CDAI-70, (E) maintenance of CDAI-100, in overall patients with moderate-to-severe Crohn’s disease, and (B) (D) (F) are their respectively plots moderated by including outcomes timepoint as a moderator. 38](#_Toc183973827)

[**Supplementary Figure 11.** Funnel plots of assessments of small study and publication bias in evaluation of (A) risk of adverse events in maintenance therapy, (C) risk of serious adverse events in maintenance therapy, (E) risk of serious infections in maintenance therapy, in overall patients with moderate-to-severe Crohn’s disease, and (B) (D) (F) are their respectively plots moderated by including outcomes timepoint as a moderator. 39](#_Toc183973828)

[**Supplementary Figure 12.** Funnel plots of assessments of small study and publication bias in evaluation of (A) induction of clinical remission, (C) induction of CDAI-70, (E) induction of CDAI-100, in tumor necrosis factor antagonist-naïve patients with moderate-to-severe Crohn’s disease, and (B) (D) (F) are their respectively plots moderated by including outcomes timepoint as a moderator. 40](#_Toc183973829)

[**Supplementary Figure 13.** Funnel plots of assessments of small study and publication bias in evaluation of (A) induction of clinical remission, (C) induction of CDAI-70, (E) induction of CDAI-100, in tumor necrosis factor antagonist-experienced patients with moderate-to-severe Crohn’s disease, and (B) (D) (F) are their respectively plots moderated by including outcomes timepoint as a moderator. 41](#_Toc183973830)

[**Supplementary Figure 14.** Funnel plots of assessments of small study and publication bias in evaluation of maintenance of clinical remission (A) in tumor necrosis factor antagonist-naïve patients, (C) in tumor necrosis factor antagonist-experienced patients, with moderate-to-severe Crohn’s disease, and (B) (D) are their respectively plots moderated by including outcomes timepoint as a moderator. 42](#_Toc183973831)

[**Supplementary Figure 15.** Leverage plots of assessing model fit of evaluations of (A) induction of clinical remission, (B) induction of CDAI-70, (C) induction of CDAI-100, (D) risk of adverse events in induction therapy, (E) risk of serious adverse events in induction therapy, in overall patients with moderate-to-severe Crohn’s disease. 43](#_Toc183973832)

[**Supplementary Figure 16.** Leverage plots of assessing model fit of evaluations of (A) maintenance of clinical remission, (B) maintenance of CDAI-70, (C) maintenance of CDAI-100, (D) risk of adverse events in maintenance therapy, (E) risk of serious adverse events in maintenance therapy, in overall patients with moderate-to-severe Crohn’s disease. 44](#_Toc183973833)

[**Supplementary Figure 17.** Leverage plots of assessing model fit of evaluations of (A) induction of clinical remission, (B) induction of CDAI-70, (C) induction of CDAI-100, in tumor necrosis factor antagonist-naïve patients with moderate-to-severe Crohn’s disease. 45](#_Toc183973834)

[**Supplementary Figure 18.** Leverage plots of assessing model fit of evaluations of (A) induction of clinical remission, (B) induction of CDAI-70, (C) induction of CDAI-100, in tumor necrosis factor antagonist-experienced patients with moderate-to-severe Crohn’s disease. 45](#_Toc183973835)

[**Supplementary Figure 19.** Leverage plots of assessing model fit of evaluations of maintenance of clinical remission (A) in tumor necrosis factor antagonist-naïve patients, (B) in tumor necrosis factor antagonist-experienced patients, with moderate-to-severe Crohn’s disease. 46](#_Toc183973836)

[**Supplementary Figure 20.** Brooks-Gelman-Rubin diagnosis plot for Markov chain Monte Carlo (MCMC) chains of evaluations of (A) inducing clinical remission, (B) inducing CDAI-70, (C) inducing CDAI-100, and (D) risk of adverse events in induction therapy, (E) risk of serious adverse events in induction therapy, (F) risk of serious infections in induction therapy, between included biologic agents in overall patients with moderate-to-severe Crohn’s disease. 47](#_Toc183973837)

[**Supplementary Figure 21.** Brooks-Gelman-Rubin diagnosis plot for Markov chain Monte Carlo (MCMC) chains of evaluations of (A) maintaining clinical remission, (B) maintaining CDAI-70, (C) maintaining CDAI-100, and (D) risk of adverse events in maintenance therapy, (E) risk of serious adverse events in maintenance therapy, (F) risk of serious infections in maintenance therapy, between included biologic agents in overall patients with moderate-to-severe Crohn’s disease. 51](#_Toc183973838)

[**Supplementary Figure 22.** Brooks-Gelman-Rubin diagnosis plot for Markov chain Monte Carlo (MCMC) chains of evaluations of (A) inducing clinical remission, (B) inducing CDAI-70, (C) inducing CDAI-100, between included biologic agents in tumor necrosis factor antagonist-naïve patients with moderate-to-severe Crohn’s disease. 56](#_Toc183973839)

[**Supplementary Figure 23.** Brooks-Gelman-Rubin diagnosis plot for Markov chain Monte Carlo (MCMC) chains of evaluations of (A) inducing clinical remission, (B) inducing CDAI-70, (C) inducing CDAI-100, between included biologic agents in tumor necrosis factor antagonist-experienced patients with moderate-to-severe Crohn’s disease. 58](#_Toc183973840)

[**Supplementary Figure 24.** Brooks-Gelman-Rubin diagnosis plot for Markov chain Monte Carlo (MCMC) chains of evaluations of maintaining clinical remission between included biologic agents (A) in tumor necrosis factor antagonist-naïve patients, (B) in tumor necrosis factor antagonist-experienced patients, with moderate-to-severe Crohn’s disease. 60](#_Toc183973841)

[**Supplementary Figure 25.** League heat plots of mixed comparisons of inducing clinical remission between included biologic agents in overall patients with moderate-to-severe Crohn’s disease, reporting odds ratios on the logarithmic scale (log OR) with 95% confidence intervals. Log OR greater or less than 0 are indicated in red or blue, respectively, in comparisons of inducing clinical remission, and the darker the color, the greater the odds ratios. Bold type represents statistically significant superiority/inferiority of the intervention over the comparator. 62](#_Toc183973842)

[**Supplementary Figure 26.** League heat plot of mixed comparisons of risk of adverse events and risk of serious adverse events in induction therapy between included biologic agents in overall patients with moderate-to-severe Crohn’s disease, reporting odds ratios on the logarithmic scale (log OR) with 95% confidence intervals. Log OR greater or less than 0 are indicated in red (yellow) or blue (green), respectively, in comparisons of risk of adverse events (risk of serious adverse events) in induction therapy, and the darker the color, the greater the odds ratios. Bold type represents statistically significant superiority/inferiority of the intervention over the comparator. 63](#_Toc183973843)

[**Supplementary Figure 27.** League heat plot of mixed comparisons of risk of serious infections in induction therapy between included biologic agents in overall patients with moderate-to-severe Crohn’s disease, reporting odds ratios on the logarithmic scale (log OR) with 95% confidence intervals. Log OR greater or less than 0 are indicated in red or blue, respectively, in comparisons of risk of serious infections in induction therapy, and the darker the color, the greater the odds ratios. Bold type represents statistically significant superiority/inferiority of the intervention over the comparator. 63](#_Toc183973844)

[**Supplementary Figure 28.** League heat plots of mixed comparisons of maintaining CDAI-100 and CDAI-70 between included biologic agents in overall patients with moderate-to-severe Crohn’s disease, reporting odds ratios on the logarithmic scale (log OR) with 95% confidence intervals. Log OR greater or less than 0 are indicated in red (yellow) or blue (green), respectively, in comparisons of maintaining CDAI-100 (CDAI-70), and the darker the color, the greater the odds ratios. Bold type represents statistically significant superiority/inferiority of the intervention over the comparator. 64](#_Toc183973845)

[**Supplementary Figure 29.** League heat plot of mixed comparisons of risk of adverse events and risk of serious adverse events in maintenance therapy between included biologic agents in overall moderate-to-severe Crohn’s disease, reporting odds ratios on the logarithmic scale (log OR) with 95% confidence intervals. Log OR greater or less than 0 are indicated in red (yellow) or blue (green), respectively, in comparisons of risk of adverse events (risk of serious adverse events) in maintenance therapy, and the darker the color, the greater the odds ratios. Bold type represents statistically significant superiority/inferiority of the intervention over the comparator. 65](#_Toc183973846)

[**Supplementary Figure 30.** League heat plot of mixed comparisons of risk of serious infections in maintenance therapy between included biologic agents in overall moderate-to-severe Crohn’s disease, reporting odds ratios on the logarithmic scale (log OR) with 95% confidence intervals. Log OR greater or less than 0 are indicated in red or blue, respectively, in comparisons of risk of serious infections in maintenance therapy, and the darker the color, the greater the odds ratios. Bold type represents statistically significant superiority/inferiority of the intervention over the comparator. 66](#_Toc183973847)

[**Supplementary Figure 31.** League heat plots of mixed comparisons of inducing clinical remission between included biologic agents in tumor necrosis factor antagonist-naïve patients with moderate-to-severe Crohn’s disease, reporting odds ratios on the logarithmic scale (log OR) with 95% confidence intervals. Log OR greater or less than 0 are indicated in red or blue, respectively, in comparisons of inducing clinical remission, and the darker the color, the greater the odds ratios. Bold type represents statistically significant superiority/inferiority of the intervention over the comparator. 67](#_Toc183973848)

[**Supplementary Figure 32.** League heat plots of mixed comparisons of inducing clinical remission between included biologic agents in tumor necrosis factor antagonist-experienced patients with moderate-to-severe Crohn’s disease, reporting odds ratios on the logarithmic scale (log OR) with 95% confidence intervals. Log OR greater or less than 0 are indicated in red or blue, respectively, in comparisons of inducing clinical remission, and the darker the color, the greater the odds ratios. Bold type represents statistically significant superiority/inferiority of the intervention over the comparator. 67](#_Toc183973849)

[**Supplementary Figure 33.** Sets of plots (including SUCRA plot, rankogram, and forest plot from top to bottom) of mixed comparisons of (A) inducing clinical remission, (B) inducing CDAI-70, (C) inducing CDAI-100, between included biologic agents in overall patients with moderate-to-severe Crohn’s disease. 68](#_Toc183973850)

[**Supplementary Figure 34.** Sets of plots (including SUCRA plot, rankogram, and forest plot from top to bottom) of mixed comparisons of (A) risk of adverse events, (B) risk of serious adverse events, (C) risk of serious infections, in induction therapy between included biologic agents in overall patients with moderate-to-severe Crohn’s disease. 69](#_Toc183973851)

[**Supplementary Figure 35.** Sets of plots (including SUCRA plot, rankogram, and forest plot from top to bottom) of mixed comparisons of (A) maintaining clinical remission, (B) maintaining CDAI-70, (C) maintaining CDAI-100, between included biologic agents in overall patients with moderate-to-severe Crohn’s disease. 70](#_Toc183973852)

[**Supplementary Figure 36.** Sets of plots (including SUCRA plot, rankogram, and forest plot from top to bottom) of mixed comparisons of (A) risk of adverse events, (B) risk of serious adverse events, (C) risk of serious infections, in maintenance therapy between included biologic agents in overall patients with moderate-to-severe Crohn’s disease. 71](#_Toc183973853)

[**Supplementary Figure 37.** Sets of plots (including SUCRA plot, rankogram, and forest plot from top to bottom) of mixed comparisons of (A) inducing clinical remission, (B) inducing CDAI-70, (C) inducing CDAI-100, between included biologic agents in tumor necrosis factor antagonist-naïve patients with moderate-to-severe Crohn’s disease. 72](#_Toc183973854)

[**Supplementary Figure 38.** Sets of plots (including SUCRA plot, rankogram, and forest plot from top to bottom) of mixed comparisons of (A) inducing clinical remission, (B) inducing CDAI-70, (C) inducing CDAI-100, between included biologic agents in tumor necrosis factor antagonist-experienced patients with moderate-to-severe Crohn’s disease. 73](#_Toc183973855)

[**Supplementary Figure 39.** Sets of plots (including SUCRA plot, rankogram, and forest plot from top to bottom) of mixed comparisons of inducing clinical remission, between included biologic agents (A) in tumor necrosis factor antagonist-naïve patients, (B) in tumor necrosis factor antagonist-experienced patients, with moderate-to-severe Crohn’s disease. 74](#_Toc183973856)

[**Supplementary Figure 40.** Consistency vs inconsistency plots of evaluations of (A) inducing clinical remission, (B) inducing CDAI-70, (C) inducing CDAI-100, (D) risk of adverse events in induction therapy, (E) risk of serious adverse events in induction therapy, (F) risk of serious infections in induction therapy, (G) maintaining clinical remission, (H) maintaining CDAI-70, (I) maintaining CDAI-100, (J) risk of adverse events in maintenance therapy, (K) risk of serious adverse events in maintenance therapy, (L) risk of serious infections in maintenance therapy in over patients with Crohn’s disease; (M) inducing clinical remission, (N) inducing CDAI-70, (O) inducing CDAI-100, (P) maintaining clinical remission, in tumor necrosis factor antagonist-naïve patients with Crohn’s disease; (Q) inducing clinical remission, (R) inducing CDAI-70, (S) inducing CDAI-100, (T) maintaining clinical remission, in tumor necrosis factor antagonist-experienced patients with Crohn’s disease. 75](#_Toc183973857)

[**Supplementary Table 2.** Certainty of evidence on the efficacy of biologic agents in the induction therapy of overall patients with moderate-to-severe Crohn’s disease, based on GRADE approach for network meta-analysis. 76](#_Toc183973858)

[**Supplementary Table 3.** Certainty of evidence on the efficacy of biologic agents in the induction therapy of TNF antagonist-naïve patients with moderate-to-severe Crohn’s disease, based on GRADE approach for network meta-analysis. 79](#_Toc183973859)

[**Supplementary Table 4.** Certainty of evidence on the efficacy of biologic agents in the induction therapy of TNF antagonist-experienced patients with moderate-to-severe Crohn’s disease, based on GRADE approach for network meta-analysis. 81](#_Toc183973860)

[**Supplementary Table 5.** Certainty of evidence on the efficacy of biologic agents in the maintenance therapy of overall patients with moderate-to-severe Crohn’s disease, based on GRADE approach for network meta-analysis. 82](#_Toc183973861)

[**Supplementary Table 6.** Certainty of evidence on the efficacy of biologic agents for the maintenance of clinical remission, based on GRADE approach for network meta-analysis. 86](#_Toc183973862)

[**Supplementary Figure 41.** Flow diagram of selection. 88](#_Toc183973863)

[**Supplementary Table 7:** Characteristics of included randomized controlled trials comparing biologic agents for induction therapy in patients with moderate-to-severe Crohn’s disease. 89](#_Toc183973864)

[**Supplementary Table 8:** Characteristics of included randomized controlled trials comparing biologic agents for maintenance therapy in patients with moderate-to-severe Crohn’s disease. 93](#_Toc183973865)

[**Supplementary Result 1.** Result of evaluation of induction for clinical remission in moderate-to-severe CD patients. 100](#_Toc183973866)

[**Supplementary Result 2.** Result of evaluation of maintenance for clinical response in moderate-to-severe CD patients. 100](#_Toc183973867)

[**Supplementary Result 3.** Result of evaluation of induction for clinical remission in TNF antagonists-naïve CD patients. 100](#_Toc183973868)

[**Supplementary Result 4.** Result of evaluation of induction for clinical remission in TNF antagonists-experienced CD patients. 100](#_Toc183973869)

**Supplementary Table 1.** Search strategy

| **PubMed** | |
| --- | --- |
| #1 | “Crohn Disease”[MeSH Terms] OR Crohn's Enteritis[Title/Abstract] OR Regional Enteritis[Title/Abstract] OR Crohn's Disease[Title/Abstract] OR Crohns Disease[Title/Abstract] OR Inflammatory Bowel Disease 1[Title/Abstract] OR Enteritis, Granulomatous[Title/Abstract] OR Granulomatous Enteritis[Title/Abstract] OR Enteritis, Regional[Title/Abstract] OR Ileocolitis[Title/Abstract] OR Colitis, Granulomatous[Title/Abstract] OR Granulomatous Colitis[Title/Abstract] OR Ileitis, Terminal[Title/Abstract] OR Terminal Ileitis[Title/Abstract] OR Ileitis, Regional[Title/Abstract] OR Regional Ileitis[Title/Abstract] OR crohn* |
| #2 | "Tumor Necrosis Factor Inhibitors"[Mesh] OR “Tumor Necrosis Factor Blockers”[Title/Abstract] OR “TNF Inhibitors”[Title/Abstract] OR “Inhibitors, TNF”[Title/Abstract] OR “TNF Blockers”[Title/Abstract] OR “Blockers, TNF”[Title/Abstract] OR “Tumor Necrosis Factor Blocker”[Title/Abstract] OR “Tumor Necrosis Factor Antagonist”[Title/Abstract] OR “Tumor Necrosis Factor Inhibitor”[Title/Abstract] OR “TNF Antagonist”[Title/Abstract] OR “Antagonist, TNF”[Title/Abstract] OR “TNF Blocker”[Title/Abstract] OR “Blocker, TNF”[Title/Abstract] OR “TNF Inhibitor”[Title/Abstract] OR “Inhibitor, TNF”[Title/Abstract] OR “Tumor Necrosis Factor Antagonists”[Title/Abstract] OR “TNF Antagonists”[Title/Abstract] OR “Antagonists, TNF”[Title/Abstract] OR “Tumor Necrosis Factor-a (TNF-a) Antagonists”[Title/Abstract] OR “Tumor Necrosis Factor-a (TNF-a) Antagonist”[Title/Abstract] OR “Tumor Necrosis Factor-a (TNF-a) Inhibitors”[Title/Abstract] OR “Tumor Necrosis Factor-a (TNF-a) Inhibitor”[Title/Abstract] OR “Tumor Necrosis Factor-a (TNF-a) Blockers”[Title/Abstract] OR “Tumor Necrosis Factor-a (TNF-a) Blocker”[Title/Abstract] OR anti-tum* OR anti-TNF* OR anti-alpha OR “Infliximab”[MeSH Terms] OR “MAb cA2”[Title/Abstract] OR “Monoclonal Antibody cA2”[Title/Abstract] OR “Antibody cA2, Monoclonal”[Title/Abstract] OR “cA2, Monoclonal Antibody”[Title/Abstract] OR “Infliximab-dyyb”[Title/Abstract] OR “Infliximab dyyb”[Title/Abstract] OR “Inflectra”[Title/Abstract] OR Remicade[Title/Abstract] OR “Infliximab-abda”[Title/Abstract] OR “Infliximab abda”[Title/Abstract] OR Renflexis[Title/Abstract] OR “Adalimumab”[MeSH Terms] OR Humira[Title/Abstract] OR exemptia[Title/Abstract] OR “Adalimumab-adbm”[Title/Abstract] OR Amjevita[Title/Abstract] OR “Adalimumab-atto”[Title/Abstract] OR Cyltezo[Title/Abstract] OR “D2E7 Antibody”[Title/Abstract] OR “Antibody, D2E7”[Title/Abstract] OR “Certolizumab Pegol”[MeSH Terms] OR Cimzia[Title/Abstract] OR CDP870[Title/Abstract] OR CDP 870[Title/Abstract] OR “Interleukin-12”[MeSH Terms] OR “Natural Killer Cell Stimulatory Factor”[Title/Abstract] OR IL-12[Title/Abstract] OR “Cytotoxic Lymphocyte Maturation Factor”[Title/Abstract] OR “IL 12”[Title/Abstract] OR “IL-12 p70”[Title/Abstract] OR “Interleukin-12 p70”[Title/Abstract] OR “Interleukin 12 p70”[Title/Abstract] OR “Interleukin 12”[Title/Abstract] OR IL12[Title/Abstract] OR “Edodekin Alfa”[Title/Abstract] OR “Interleukin-23”[MeSH Terms] OR “Interleukin 23”[Title/Abstract] OR IL-23[Title/Abstract] OR "Ustekinumab"[Mesh] OR Stelara[Title/Abstract] OR “CNTO 1275”[Title/Abstract] OR “CNTO-1275”[Title/Abstract] OR "risankizumab"[Supplementary Concept] OR ABBV-066[Title/Abstract] OR skyrizi[Title/Abstract] OR risankizumab-rzaa[Title/Abstract] OR “BI 655066”[Title/Abstract] OR BI-655066[Title/Abstract] OR “integrin alpha4beta7” OR “intestinal homing receptor alpha4beta7”[Title/Abstract] OR “lymphocyte-Peyer's patch adhesion molecule”[Title/Abstract] OR "alpha(4)beta(7) integrin"[Title/Abstract] OR "alpha(4)beta(p) integrin"[Title/Abstract] OR "anti‐alpha4*" OR "anti alpha 4*" OR "anti‐alpha4beta7*" OR "alpha4beta7 antibod*" OR “alpha4beta7 inhibit*” OR "vedolizumab"[Supplementary Concept] OR Entyvio[Title/Abstract] OR MLN0002[Title/Abstract] OR MLN02[Title/Abstract] OR MLN-0002[Title/Abstract] OR MLN-02[Title/Abstract] |
| #3 | ("clinical"[Title/Abstract] AND "trial"[Title/Abstract]) OR "clinical trials as topic"[MeSH Terms] OR "clinical trial"[Publication Type] OR "random*"[Title/Abstract] OR "random allocation"[MeSH Terms] OR "therapeutic use"[MeSH Subheading] |
| #4 | #1 AND #2 AND #3 |
| #5 | Limit #4 to humans |
| **Embase** | |
| #1 | 'crohn disease'/syn OR crohn* |
| #2 | 'tumor necrosis factor inhibitor'/syn OR 'tumor necrosis factor antibody'/syn OR 'anti-tum*' OR 'anti-tnf*' OR 'anti-alpha' OR 'infliximab'/syn OR 'adalimumab'/syn OR 'certolizumab pegol'/syn OR 'interleukin 12'/syn OR 'interleukin 12p40'/syn OR 'interleukin 23'/syn OR 'interleukin 23p19'/syn OR 'ustekinumab'/syn OR 'risankizumab/syn' OR 'integrin alpha4beta7'/syn OR 'integrin alpha4 beta7'/syn OR 'anti-alpha4*' OR 'anti alpha4*' OR 'antialpha4*' OR 'alpha4beta7 antibod*' OR 'vedolizumab'/syn |
| #3 | 'clinical':ti,ab AND 'trial':ti,ab OR 'clinical trial'/exp OR random* OR 'drug therapy':lnk |
| #4 | ('animal'/syn OR animal:kw OR 'nonhuman'/syn) NOT (('human'/syn OR human) AND 'cell'/syn OR human:ti OR humans:ti) |
| #5 | #1 AND #2 AND #3 NOT #4 |
| **Web of Science** | |
| #1 | TS=("Crohn Disease" OR "Crohn's Enteritis" OR "Regional Enteritis" OR "Crohn's Disease" OR "Crohns Disease" OR "Inflammatory Bowel Disease 1" OR "Enteritis, Granulomatous" OR "Granulomatous Enteritis" OR "Enteritis, Regional" OR Ileocolitis OR "Colitis, Granulomatous" OR "Granulomatous Colitis" OR "Ileitis, Terminal" OR "Terminal Ileitis" OR "Ileitis, Regional" OR "Regional Ileitides" OR "Regional Ileitis" OR crohn*) |
| #2 | TS=("Tumor Necrosis Factor Inhibitors" OR “Tumor Necrosis Factor Blockers” OR “TNF Inhibitors” OR “Inhibitors, TNF” OR “TNF Blockers” OR “Blockers, TNF” OR “Tumor Necrosis Factor Blocker” OR “Tumor Necrosis Factor Antagonist” OR “Tumor Necrosis Factor Inhibitor” OR “TNF Antagonist” OR “Antagonist, TNF” OR “TNF Blocker” OR “Blocker, TNF” OR “TNF Inhibitor” OR “Inhibitor, TNF” OR “Tumor Necrosis Factor Antagonists” OR “TNF Antagonists” OR “Antagonists, TNF” OR “Tumor Necrosis Factor-a (TNF-a) Antagonists” OR “Tumor Necrosis Factor-a (TNF-a) Antagonist” OR “Tumor Necrosis Factor-a (TNF-a) Inhibitors” OR “Tumor Necrosis Factor-a (TNF-a) Inhibitor” OR “Tumor Necrosis Factor-a (TNF-a) Blockers” OR “Tumor Necrosis Factor-a (TNF-a) Blocker” OR anti-tum* OR anti-TNF* OR anti-alpha OR Infliximab OR "MAb cA2" OR "Monoclonal Antibody cA2" OR "Antibody cA2, Monoclonal" OR "cA2, Monoclonal Antibody" OR Infliximab-dyyb OR "Infliximab dyyb" OR Inflectra OR Remicade OR Infliximab-abda OR "Infliximab abda" OR Renflexis OR Adalimumab OR Humira OR exemptia OR Adalimumab-adbm OR Amjevita OR Adalimumab-atto OR Cyltezo OR "D2E7 Antibody" OR "Antibody, D2E7" OR “Certolizumab Pegol” OR Cimzia OR CDP870 OR “CDP 870” OR Interleukin-12 OR "Natural Killer Cell Stimulatory Factor" OR IL-12 OR "Cytotoxic Lymphocyte Maturation Factor" OR "IL 12" OR "IL-12 p70" OR "Interleukin-12 p70" OR "Interleukin 12 p70" OR "Interleukin 12" OR IL12 OR "Edodekin Alfa" OR Interleukin-23 OR "Interleukin 23" OR IL-23 OR Ustekinumab OR Stelara OR “CNTO 1275”OR CNTO-1275 OR Risankizumab OR ABBV-066 OR skyrizi OR risankizumab-rzaa OR “BI 655066” OR BI-655066 OR "integrin alpha4beta7" OR "intestinal homing receptor alpha4beta7" OR "lymphocyte-Peyer's patch adhesion molecule" OR "alpha(4)beta(7) integrin" OR "alpha(4)beta(p) integrin" OR anti‐alpha4* OR "anti alpha4*" OR anti‐alpha4beta7* OR "alpha4beta7 antibod*" OR "alpha4beta7 inhibit*" OR vedolizumab OR Entyvio OR MLN0002 OR MLN02 OR MLN-0002 OR MLN-02) |
| #3 | TS=(“random* controlled trial” OR random* OR placebo*) |
| #4 | #1 AND #2 AND #3 |
| **Scopus** | |
|  | TITLE-ABS-KEY("Crohn Disease" OR "Crohn’s Enteritis" OR "Regional Enteritis" OR "Crohn’s Disease" OR "Crohns Disease" OR "Inflammatory Bowel Disease 1" OR "Enteritis, Granulomatous" OR "Granulomatous Enteritis" OR "Enteritis, Regional" OR Ileocolitis OR "Colitis, Granulomatous" OR "Granulomatous Colitis" OR "Ileitis, Terminal" OR "Terminal Ileitis" OR "Ileitis, Regional" OR "Regional Ileitides" OR "Regional Ileitis" OR crohn*) |
| #2 | TITLE-ABS-KEY("Tumor Necrosis Factor Inhibitors" OR “Tumor Necrosis Factor Blockers” OR “TNF Inhibitors” OR “Inhibitors, TNF” OR “TNF Blockers” OR “Blockers, TNF” OR “Tumor Necrosis Factor Blocker” OR “Tumor Necrosis Factor Antagonist” OR “Tumor Necrosis Factor Inhibitor” OR “TNF Antagonist” OR “Antagonist, TNF” OR “TNF Blocker” OR “Blocker, TNF” OR “TNF Inhibitor” OR “Inhibitor, TNF” OR “Tumor Necrosis Factor Antagonists” OR “TNF Antagonists” OR “Antagonists, TNF” OR “Tumor Necrosis Factor-a (TNF-a) Antagonists” OR “Tumor Necrosis Factor-a (TNF-a) Antagonist” OR “Tumor Necrosis Factor-a (TNF-a) Inhibitors” OR “Tumor Necrosis Factor-a (TNF-a) Inhibitor” OR “Tumor Necrosis Factor-a (TNF-a) Blockers” OR “Tumor Necrosis Factor-a (TNF-a) Blocker” OR anti-tum* OR anti-TNF* OR anti-alpha OR Infliximab OR "MAb cA2" OR "Monoclonal Antibody cA2" OR "Antibody cA2, Monoclonal" OR "cA2, Monoclonal Antibody" OR Infliximab-dyyb OR "Infliximab dyyb" OR Inflectra OR Remicade OR Infliximab-abda OR "Infliximab abda" OR "Renflexis" OR "Adalimumab" OR "Humira" OR exemptia OR Adalimumab-adbm OR Amjevita OR Adalimumab-atto OR Cyltezo OR "D2E7 Antibody" OR "Antibody, D2E7" OR “Certolizumab Pegol” OR Cimzia OR CDP870 OR “CDP 870” OR Interleukin-12 OR "Natural Killer Cell Stimulatory Factor" OR IL-12 OR "Cytotoxic Lymphocyte Maturation Factor" OR "IL 12" OR "IL-12 p70" OR "Interleukin-12 p70" OR "Interleukin 12 p70" OR "Interleukin 12" OR IL12 OR "Edodekin Alfa" OR Interleukin-23 OR "Interleukin 23" OR IL-23 OR Ustekinumab OR Stelara OR “CNTO 1275”OR CNTO-1275 OR Risankizumab OR ABBV-066 OR skyrizi OR risankizumab-rzaa OR “BI 655066” OR BI-655066 OR "integrin alpha4beta7" OR "intestinal homing receptor alpha4beta7" OR "lymphocyte-Peyer's patch adhesion molecule" OR "alpha(4)beta(7) integrin" OR "alpha(4)beta(p) integrin" OR anti‐alpha4* OR "anti alpha4*" OR anti‐alpha4beta7* OR "alpha4beta7 antibod*" OR "alpha4beta7 inhibit*" OR vedolizumab OR Entyvio OR MLN0002 OR MLN02 OR MLN-0002 OR MLN-02) |
| #3 | TITLE-ABS-KEY("random* controlled trial" OR random* OR placebo*) |
| #4 | #1 AND #2 AND #3 |
| **CENTRAL** | |
| #1 | MeSH descriptor: [Crohn Disease] explode all trees AND (“Crohn's Enteritis” OR “Regional Enteritis” OR “Crohn's Disease” OR “Crohns Disease” OR “Inflammatory Bowel Disease 1” OR “Enteritis, Granulomatous” OR “Granulomatous Enteritis” OR “Enteritis, Regional” OR “Ileocolitis” OR “Colitis, Granulomatous” OR “Granulomatous Colitis” OR “Ileitis, Terminal” OR “Terminal Ileitis” OR “Ileitis, Regional” OR “Regional Ileitis”):ti,ab,kw AND crohn* |
| #2 | MeSH descriptor: [Tumor Necrosis Factor Inhibitors] explode all trees OR (“Tumor Necrosis Factor Blockers” OR “TNF Inhibitors” OR “Inhibitors, TNF” OR “TNF Blockers” OR “Blockers, TNF” OR “Tumor Necrosis Factor Blocker” OR “Tumor Necrosis Factor Antagonist” OR “Tumor Necrosis Factor Inhibitor” OR “TNF Antagonist” OR “Antagonist, TNF” OR “TNF Blocker” OR “Blocker, TNF” OR “TNF Inhibitor” OR “Inhibitor, TNF” OR “Tumor Necrosis Factor Antagonists” OR “TNF Antagonists” OR “Antagonists, TNF” OR “Tumor Necrosis Factor-a (TNF-a) Antagonists” OR “Tumor Necrosis Factor-a (TNF-a) Antagonist” OR “Tumor Necrosis Factor-a (TNF-a) Inhibitors” OR “Tumor Necrosis Factor-a (TNF-a) Inhibitor” OR “Tumor Necrosis Factor-a (TNF-a) Blockers” OR “Tumor Necrosis Factor-a (TNF-a) Blocker”):ti,ab,kw OR anti-tum* OR anti-TNF* OR anti-alpha OR MeSH descriptor: [Infliximab] explode all trees OR ("MAb cA2" OR "Monoclonal Antibody cA2" OR "Antibody cA2, Monoclonal" OR "cA2, Monoclonal Antibody" OR Infliximab-dyyb OR "Infliximab dyyb" OR Inflectra OR Remicade OR Infliximab-abda OR "Infliximab abda" OR Renflexis):ti,ab,kw OR MeSH descriptor: [Adalimumab] explode all trees OR (Humira OR exemptia OR Adalimumab-adbm OR Amjevita OR Adalimumab-atto OR Cyltezo OR "D2E7 Antibody" OR "Antibody, D2E7"):ti,ab,kw OR MeSH descriptor: [Certolizumab Pegol] explode all trees OR (Cimzia OR CDP870 OR “CDP 870”):ti,ab,kw OR MeSH descriptor: [Interleukin-12] explode all trees OR ("Natural Killer Cell Stimulatory Factor" OR IL-12 OR "Cytotoxic Lymphocyte Maturation Factor" OR "IL 12" OR "IL-12 p70" OR "Interleukin-12 p70" OR "Interleukin 12 p70" OR "Interleukin 12" OR IL12 OR "Edodekin Alfa"):ti,ab,kw OR MeSH descriptor: [Interleukin-23] explode all trees OR ("Interleukin 23" OR IL-23):ti,ab,kw OR MeSH descriptor: [Ustekinumab] explode all trees OR (Stelara OR “CNTO 1275”OR CNTO-1275):ti,ab,kw OR (Risankizumab OR ABBV-066 OR skyrizi OR risankizumab-rzaa OR “BI 655066” OR BI-655066):ti,ab,kw OR ("integrin alpha4beta7" OR "intestinal homing receptor alpha4beta7" OR "lymphocyte-Peyer's patch adhesion molecule" OR "alpha(4)beta(7) integrin" OR "alpha(4)beta(p) integrin" OR anti‐alpha4* OR "anti alpha4*" OR anti‐alpha4beta7* OR "alpha4beta7 antibod*" OR "alpha4beta7 inhibit*"):ti,ab,kw OR (vedolizumab OR Entyvio OR MLN0002 OR MLN02 OR MLN-0002 OR MLN-02):ti,ab,kw |
| #3 | #1 AND #2 |
| **ClinicalTrials** | |
|  | Infliximab OR Adalimumab OR "Certolizumab Pegol" OR Ustekinumab OR Risankizumab OR Vedolizumab \| Interventional Studies \| Crohn Disease |
| **ICTRP** | |
|  | "Crohn Disease" AND Infliximab OR Adalimumab OR “Certolizumab Pegol” OR Ustekinumab OR Risankizumab OR Vedolizumab |

**Supplementary Information.** detailed PICOS.

**Participants/population:** adult patients (18 to 75 years old) with moderate-to-severe (defined by a Crohn's Disease Activity Index [CDAI] 220-450) active Crohn's disease.

**Intervention(s), exposure(s):** Infliximab, Adalimumab, Certolizumab pegol, Ustekinumab, Risankizumab, Vedolizumab, and related biosimilars that be searched.

**Comparator(s)/control:** placebo or other active agents.

**Main outcome(s):** the primary efficacy outcome chosen in induction trials included achievement of clinical response (defined by a reduction in the Crohn's Disease Activity Index ≥70 [CDAI-70] or ≥100 [CDAI-100] points compared to baseline). For maintenance trials, the primary efficacy outcome was maintenance of clinical remission (CDAI < 150). The safety outcomes evaluated in induction or maintenance trials were the risk of Adverse Events, Serious Adverse Events, and Serious Infections.

**Types of study to be included:** clinical randomized controlled trials.

**Supplementary Figure 1.** Traffic light plot of the risk of bias assessment.


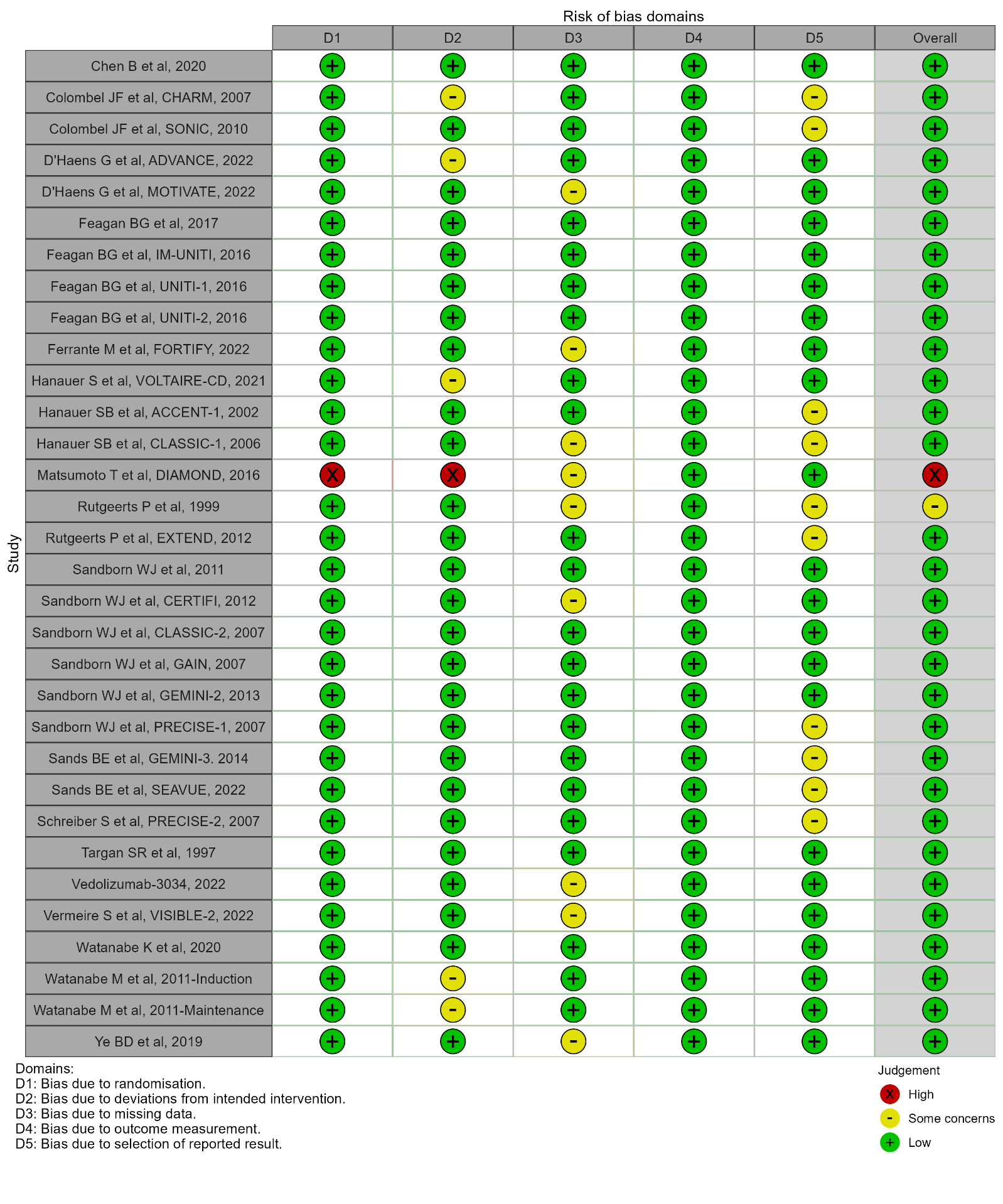


**Supplementary Figure 2A.** Network plots of the available direct comparisons of (a) induction of clinical remission, (b) induction of CDAI-70, (c) induction of CDAI-100, and (d) risk of adverse events in induction therapy, (e) risk of serious adverse events in induction therapy, (f) risk of serious infections in induction therapy, in patients with moderate-to-severe Crohn’s disease in included studies. The size of the nodes and the thickness of the edges are weighted according to the number of studies evaluating each treatment and of directly comparative arms, respectively.

| a | b |
| --- | --- |
| 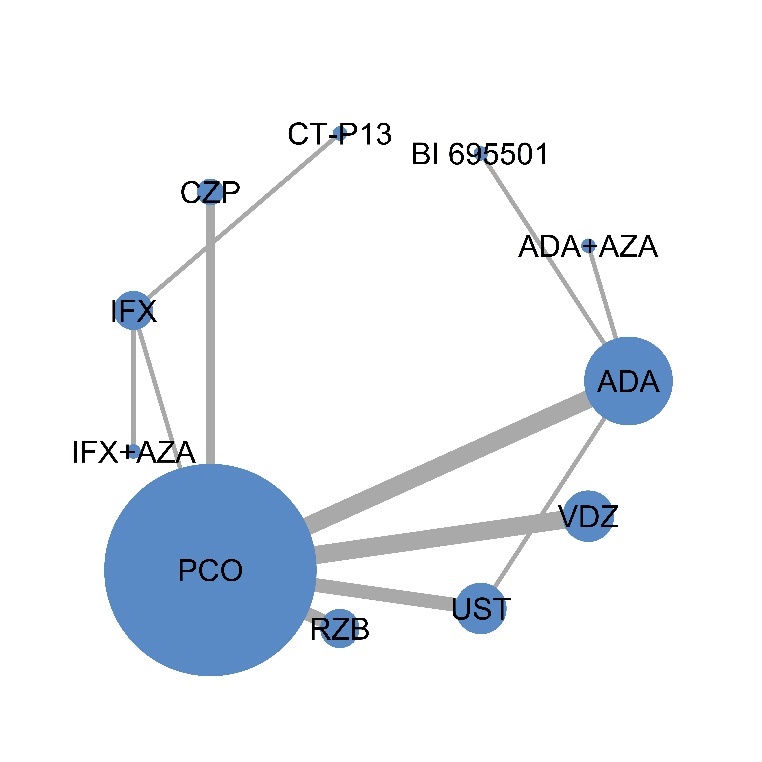 | 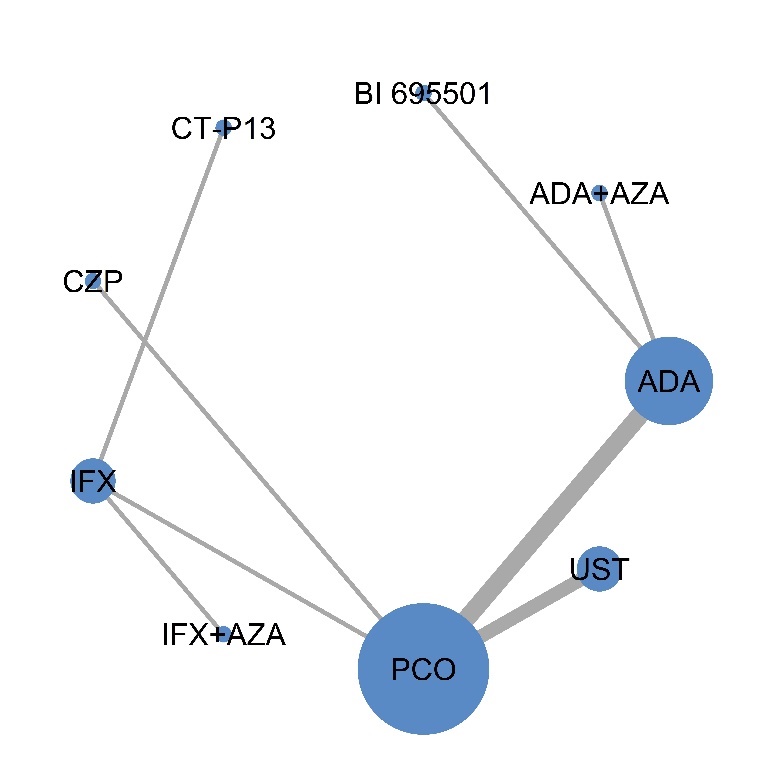 |
| c | d |
| 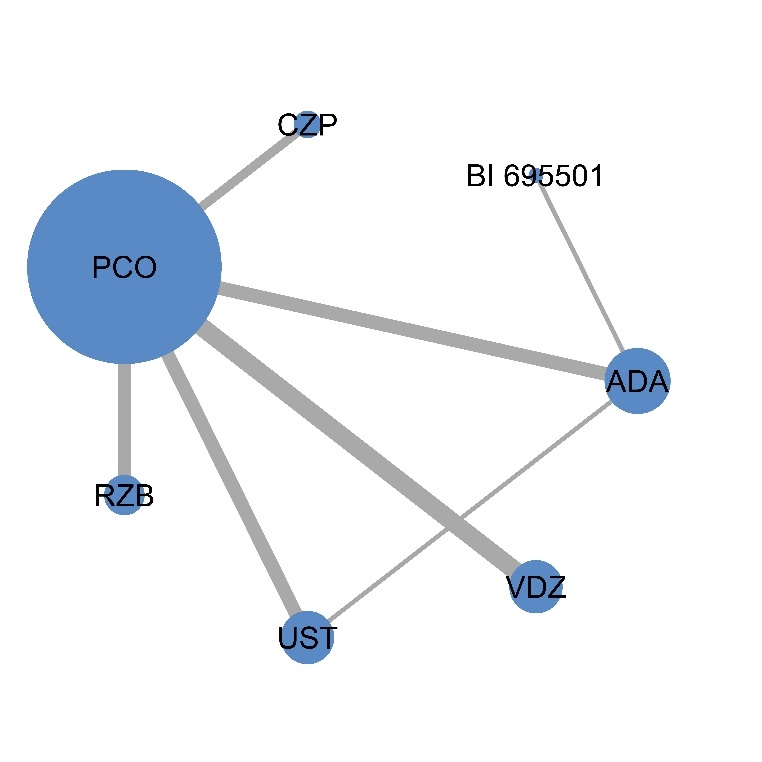 | 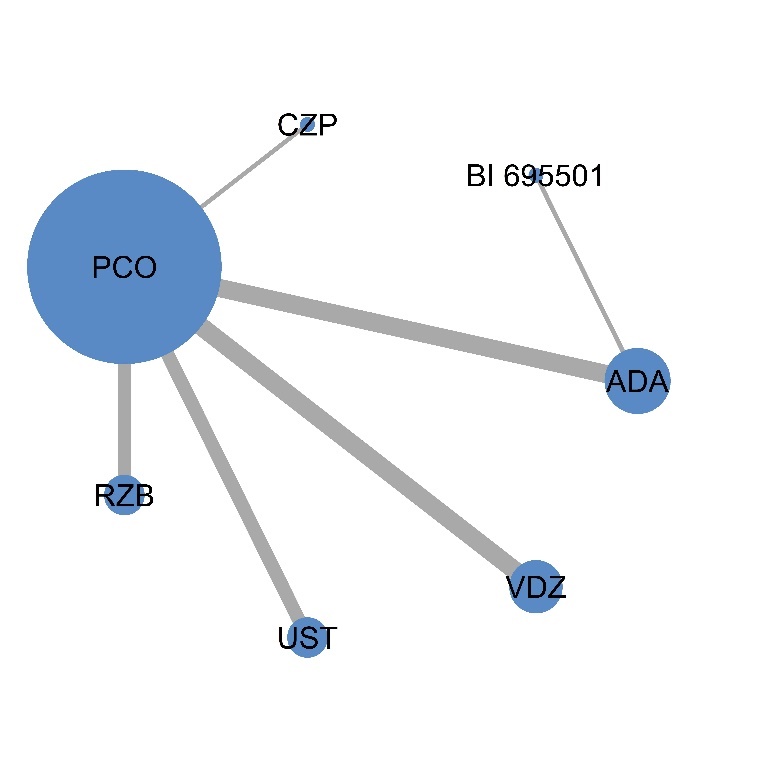 |

| e | f |
| --- | --- |
| 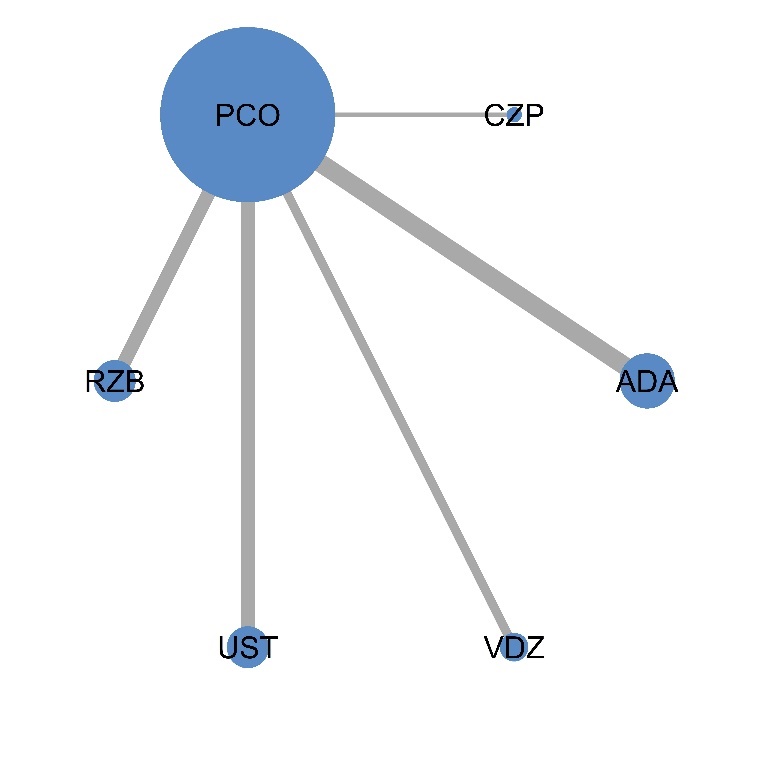 | 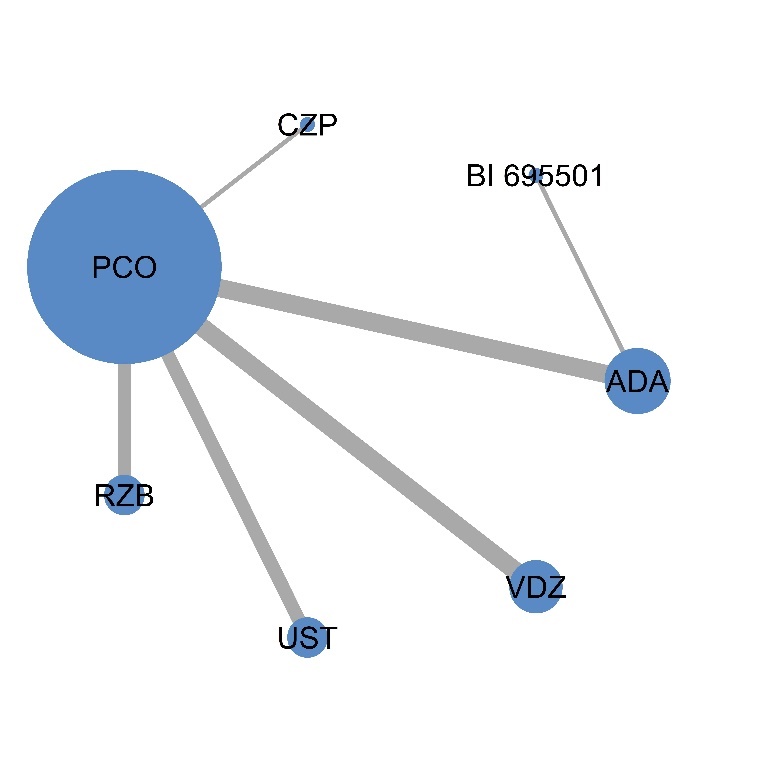 |

**Supplementary Figure 2B.** Network plots of the available direct comparisons of (a) maintenance of clinical remission, (b) maintenance of CDAI-70, (c) maintenance of CDAI-100, and (d) risk of adverse events in maintenance therapy, (e) risk of serious adverse events in maintenance therapy, (f) risk of serious infections in maintenance therapy, in patients with moderate-to-severe Crohn’s disease in included studies. The size of the nodes and the thickness of the edges are weighted according to the number of studies evaluating each treatment and of directly comparative arms, respectively.

| a | b |
| --- | --- |
| 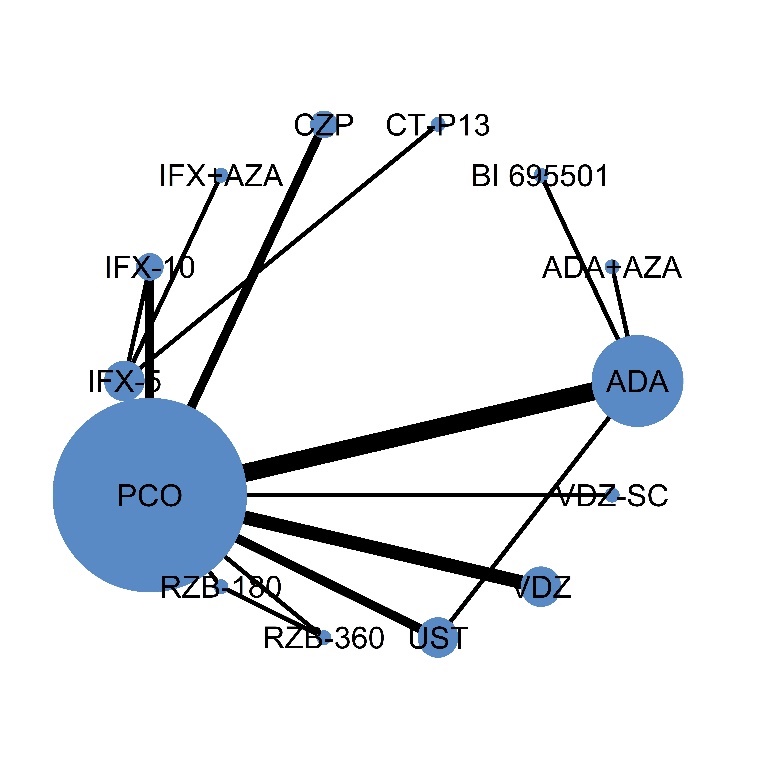 | 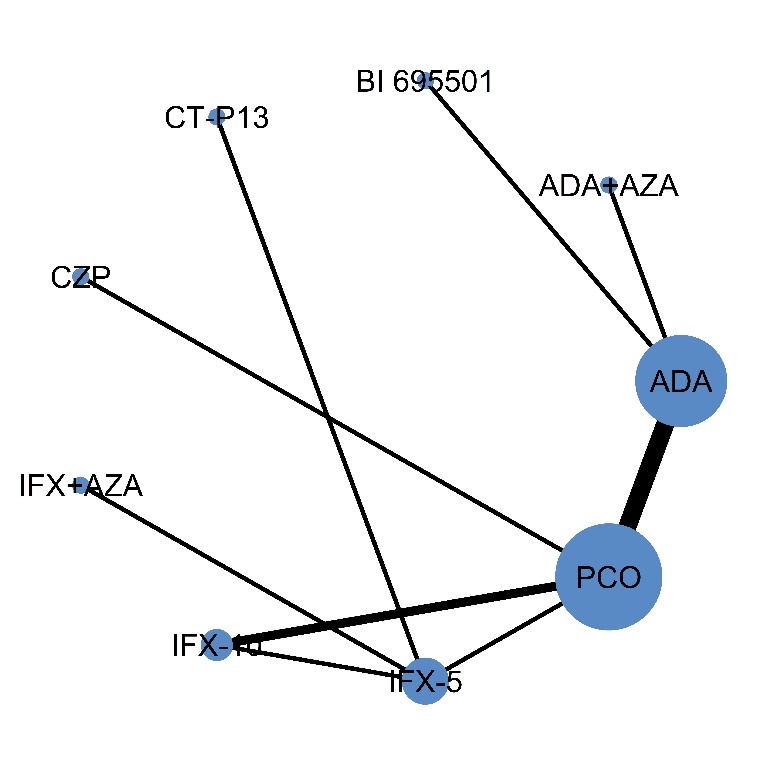 |
| c | d |
| 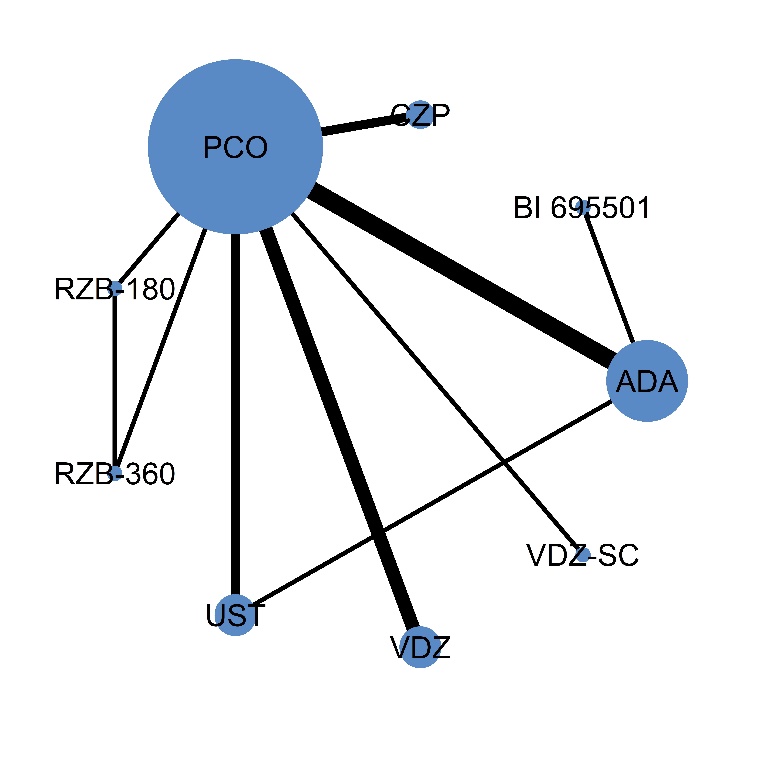 | 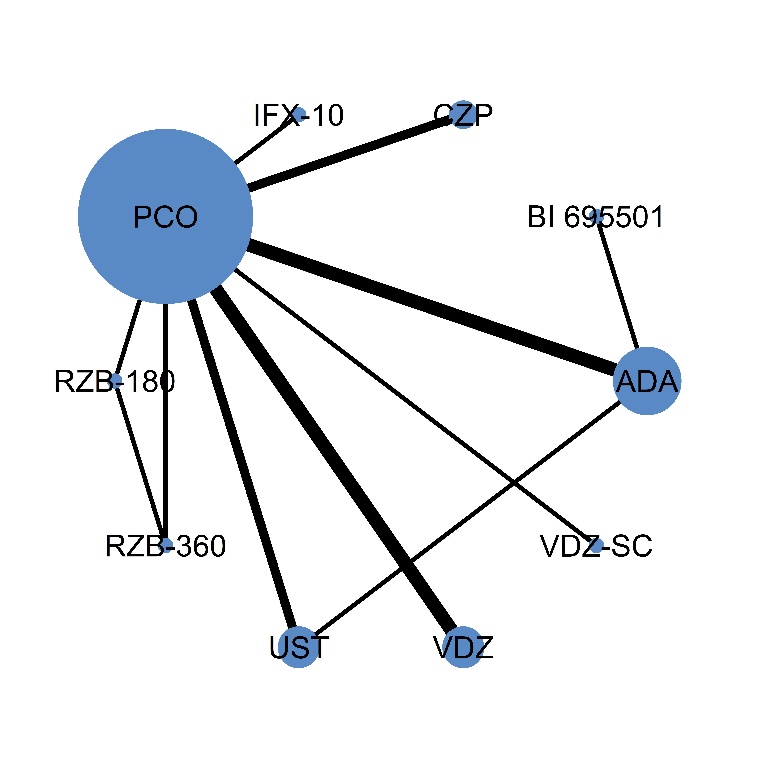 |
| e | f |
| 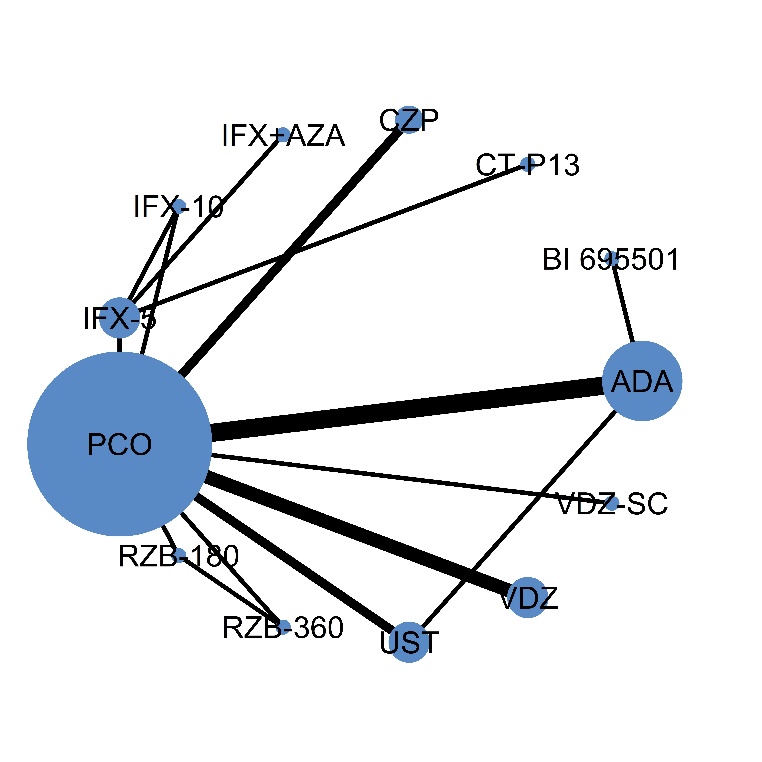 | 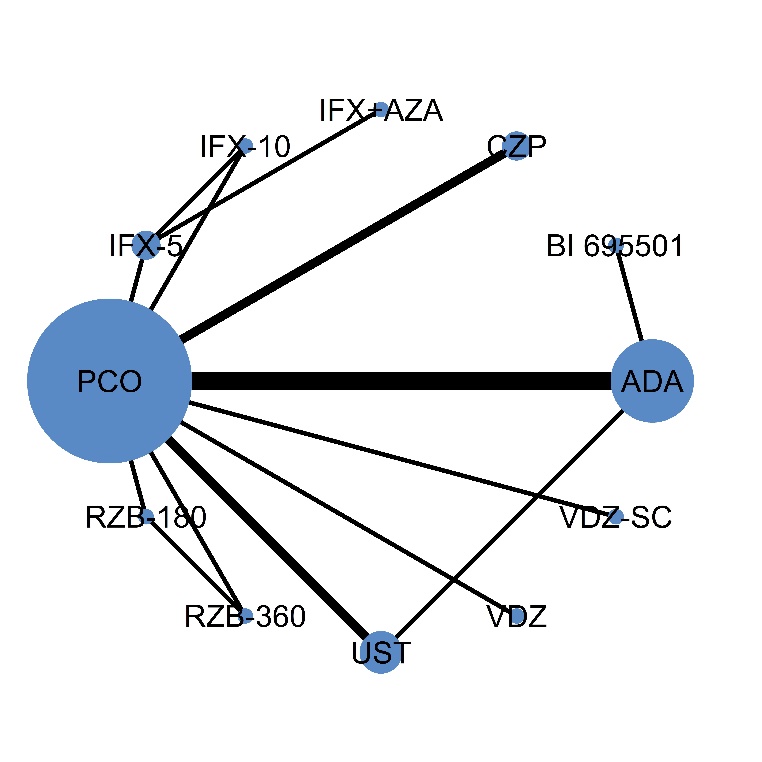 |

**Supplementary Figure 2C.** Network plots of the available direct comparisons of (a) induction of clinical remission, (b) induction of CDAI-70, (c) induction of CDAI-100, in tumor necrosis factor antagonist-naïve patients with moderate-to-severe Crohn’s disease in included studies. The size of the nodes and the thickness of the edges are weighted according to the number of studies evaluating each treatment and of directly comparative arms, respectively.

| a | b |
| --- | --- |
| 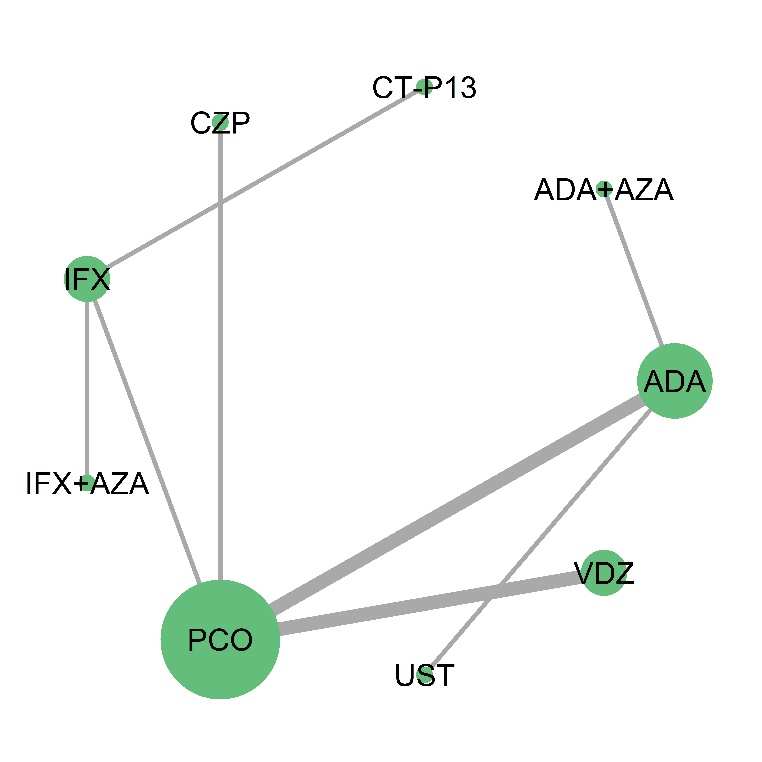 | 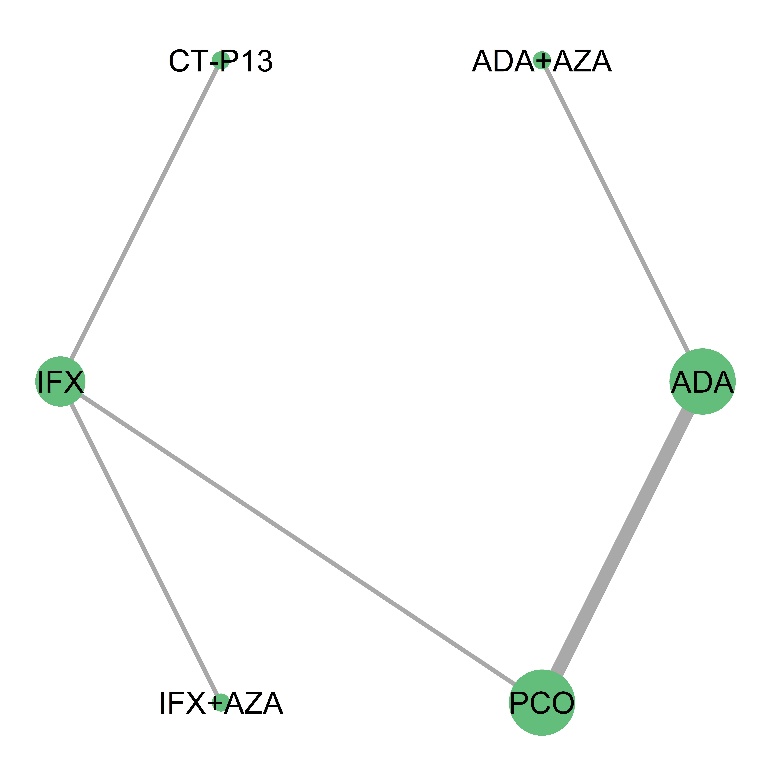 |
| c |  |
| 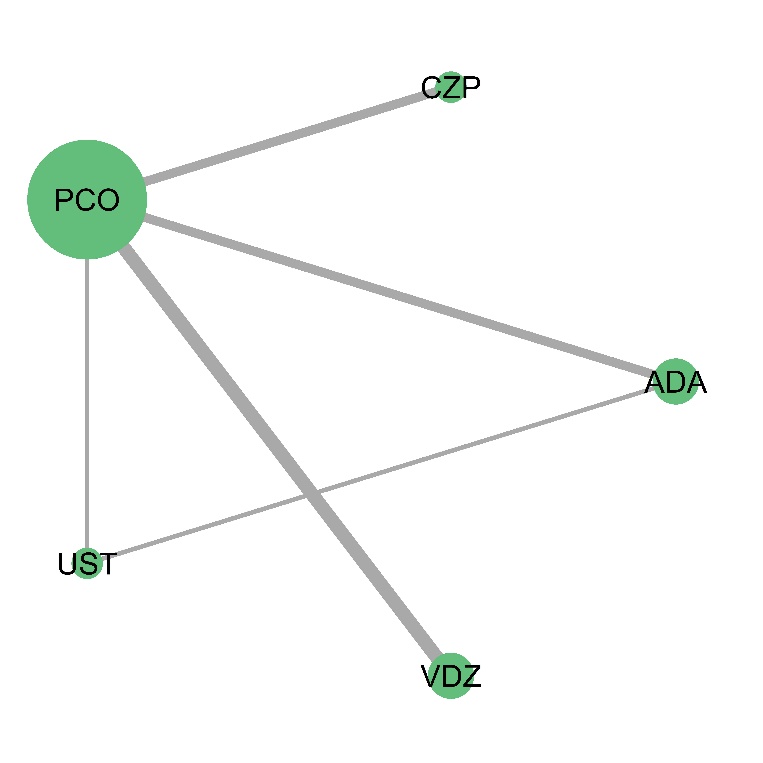 |  |

**Supplementary Figure 2D.** Network plots of the available direct comparisons of (a) induction of clinical remission, (b) induction of CDAI-70, (c) induction of CDAI-100, in tumor necrosis factor antagonist-experienced patients with moderate-to-severe Crohn’s disease in included studies. The size of the nodes and the thickness of the edges are weighted according to the number of studies evaluating each treatment and of directly comparative arms, respectively.

| a | b |
| --- | --- |
| 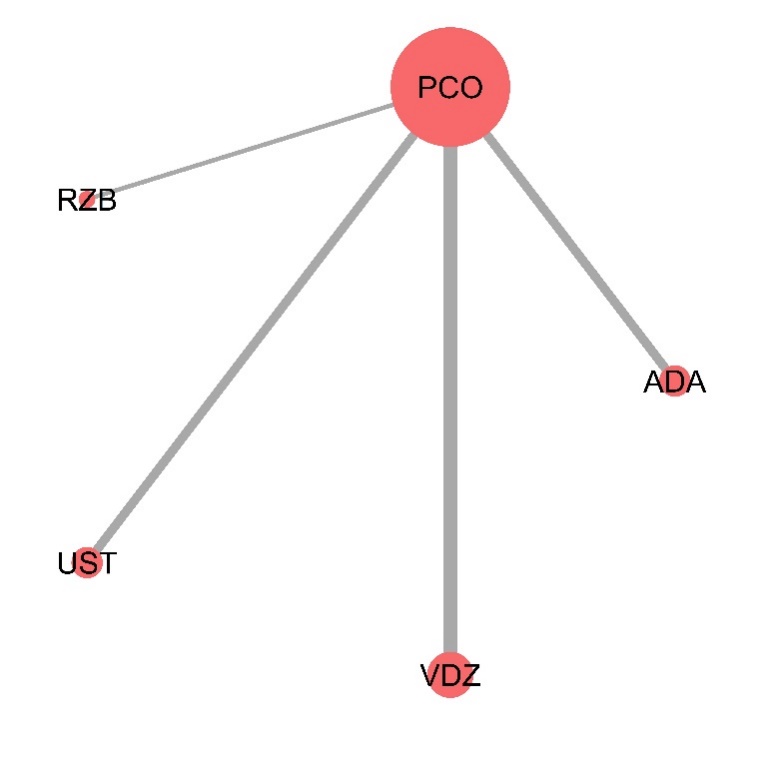 | 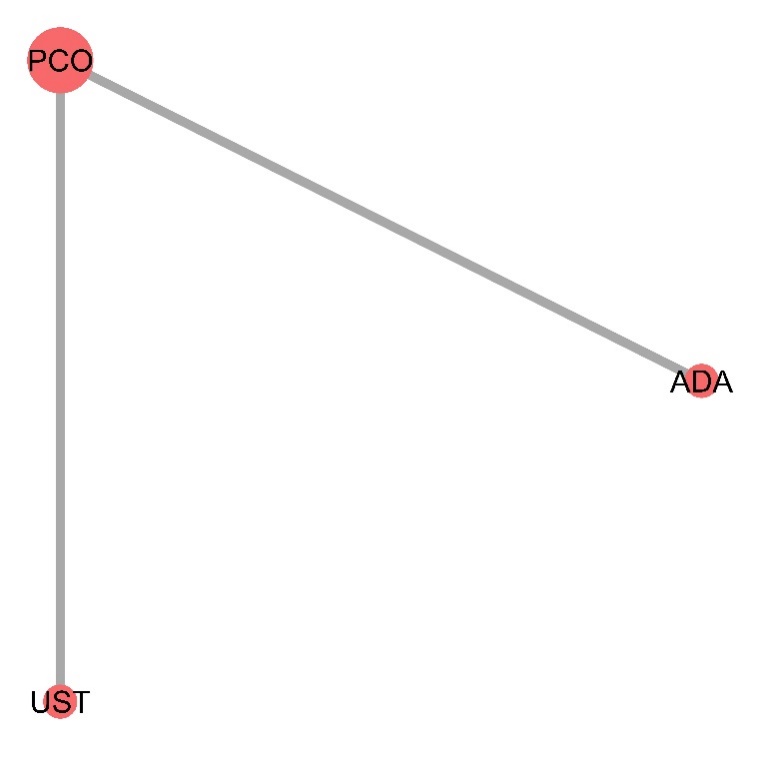 |
| c |  |
| 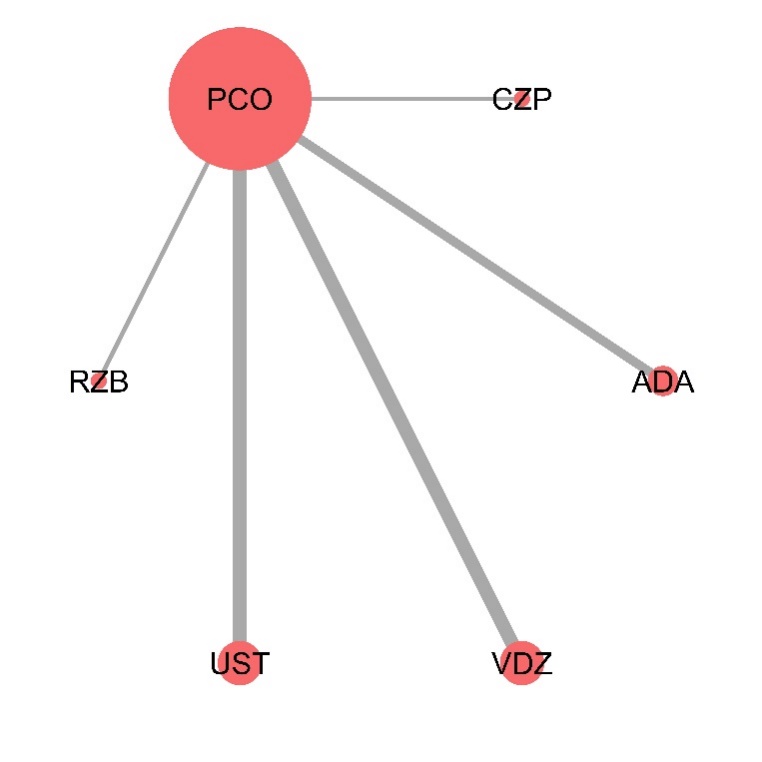 |  |

**Supplementary Figure 2E.** Network plots of the available direct comparisons of maintenance of clinical remission in (a) tumor necrosis factor antagonist-naïve patients and (b) tumor necrosis factor antagonist-experienced patients, with moderate-to-severe Crohn’s disease in included studies. The size of the nodes and the thickness of the edges are weighted according to the number of studies evaluating each treatment and of directly comparative arms, respectively.

| a | b |
| --- | --- |
| 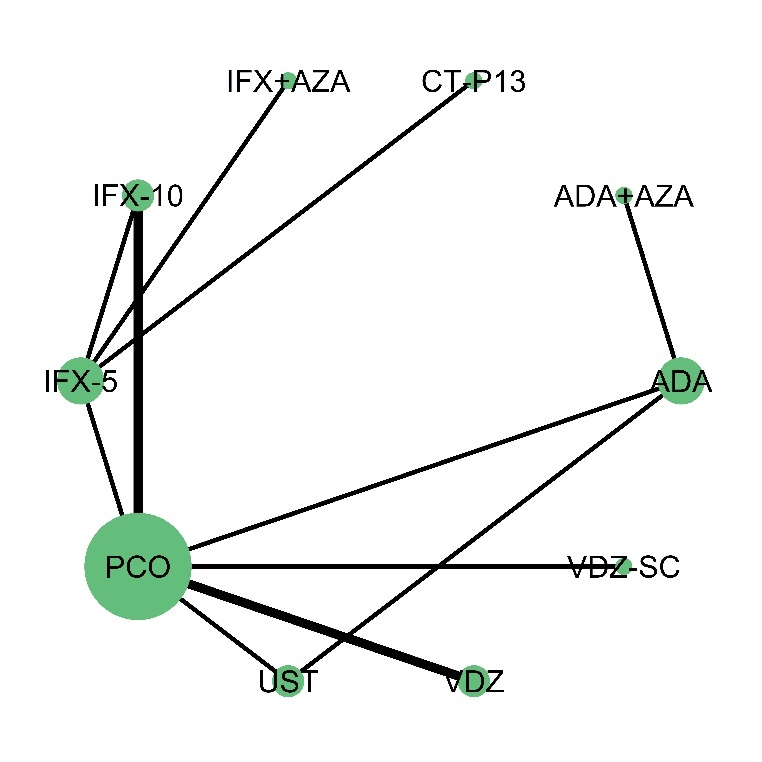 | 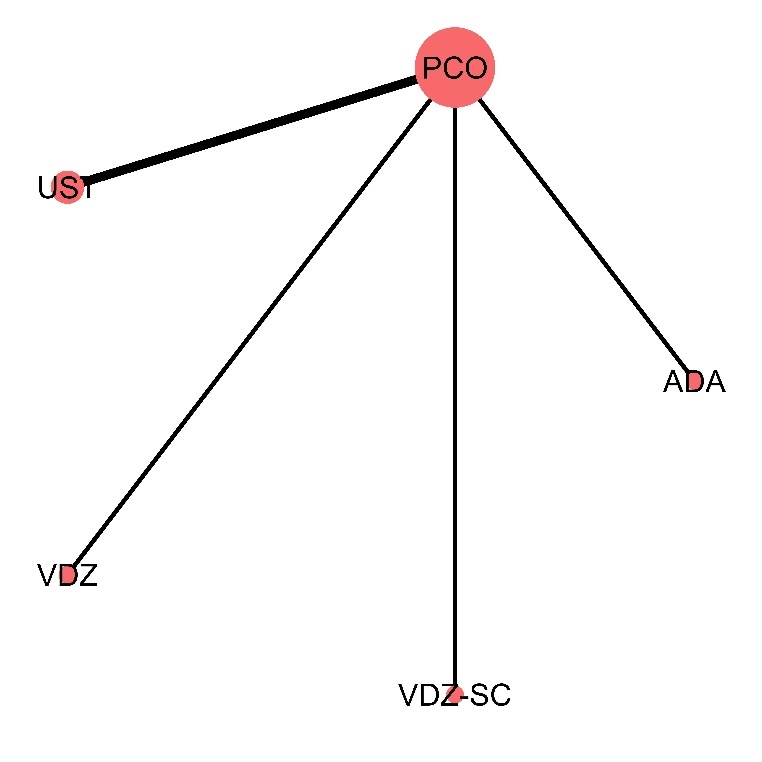 |

**Supplementary Figure 3.** Forest plots of direct comparisons of (A) induction of clinical remission, (B) induction of CDAI-70, (C) induction of CDAI-100, and (D) risk of adverse events in induction therapy, (E) risk of serious adverse events in induction therapy, (F) risk of serious infections in induction therapy, between included biologic agents in overall patients with moderate-to-severe Crohn’s disease.

A.


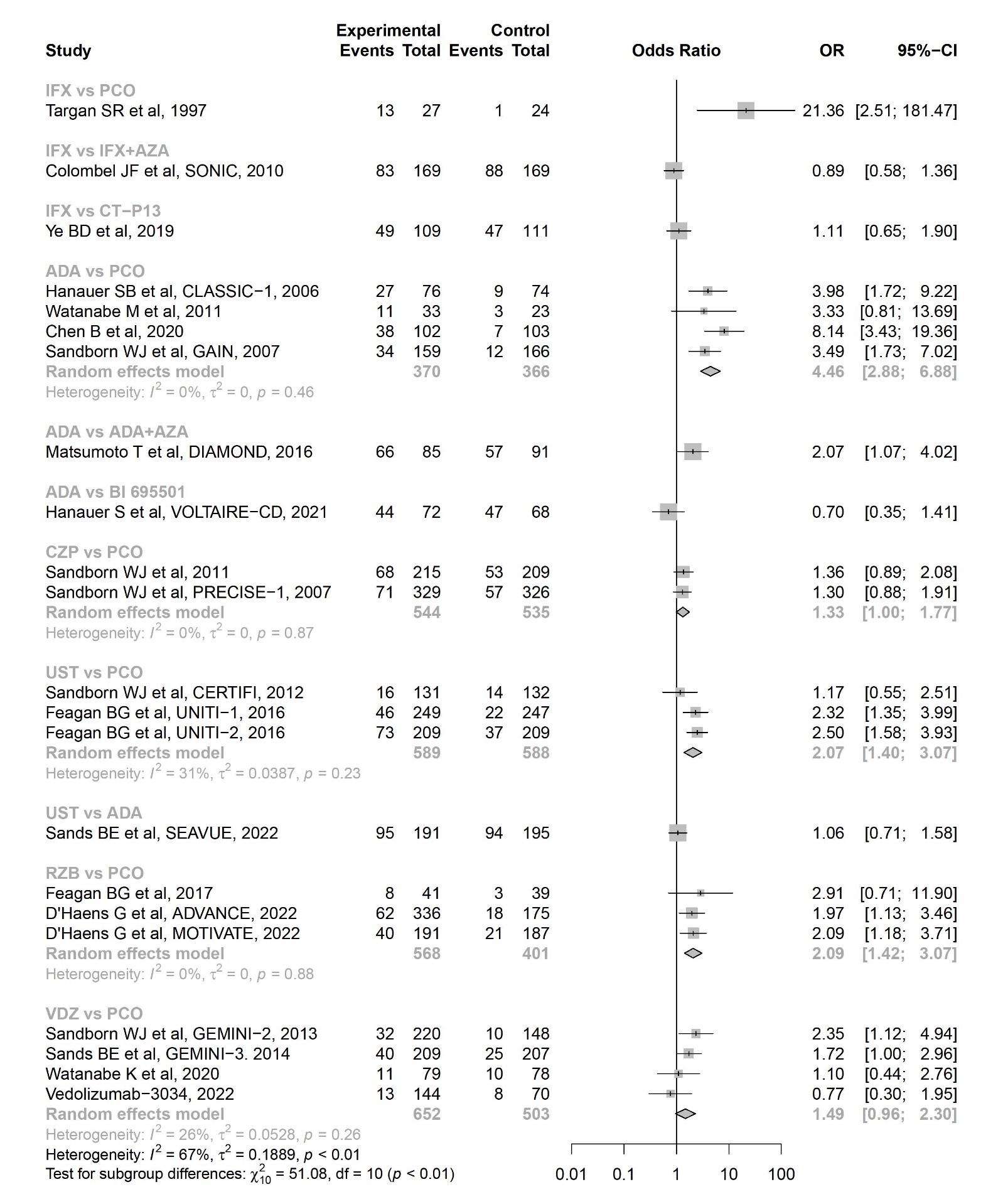


B.
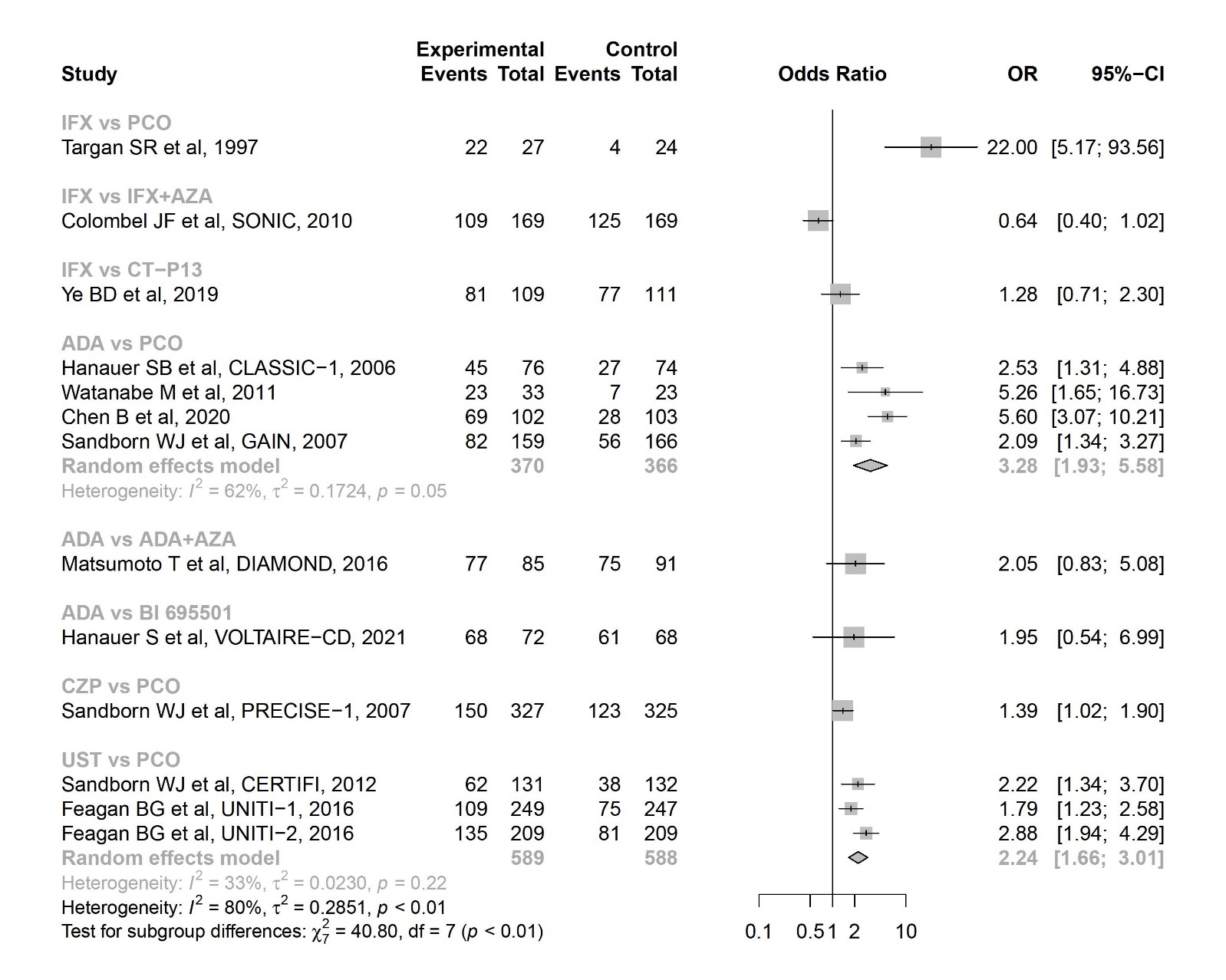


C.


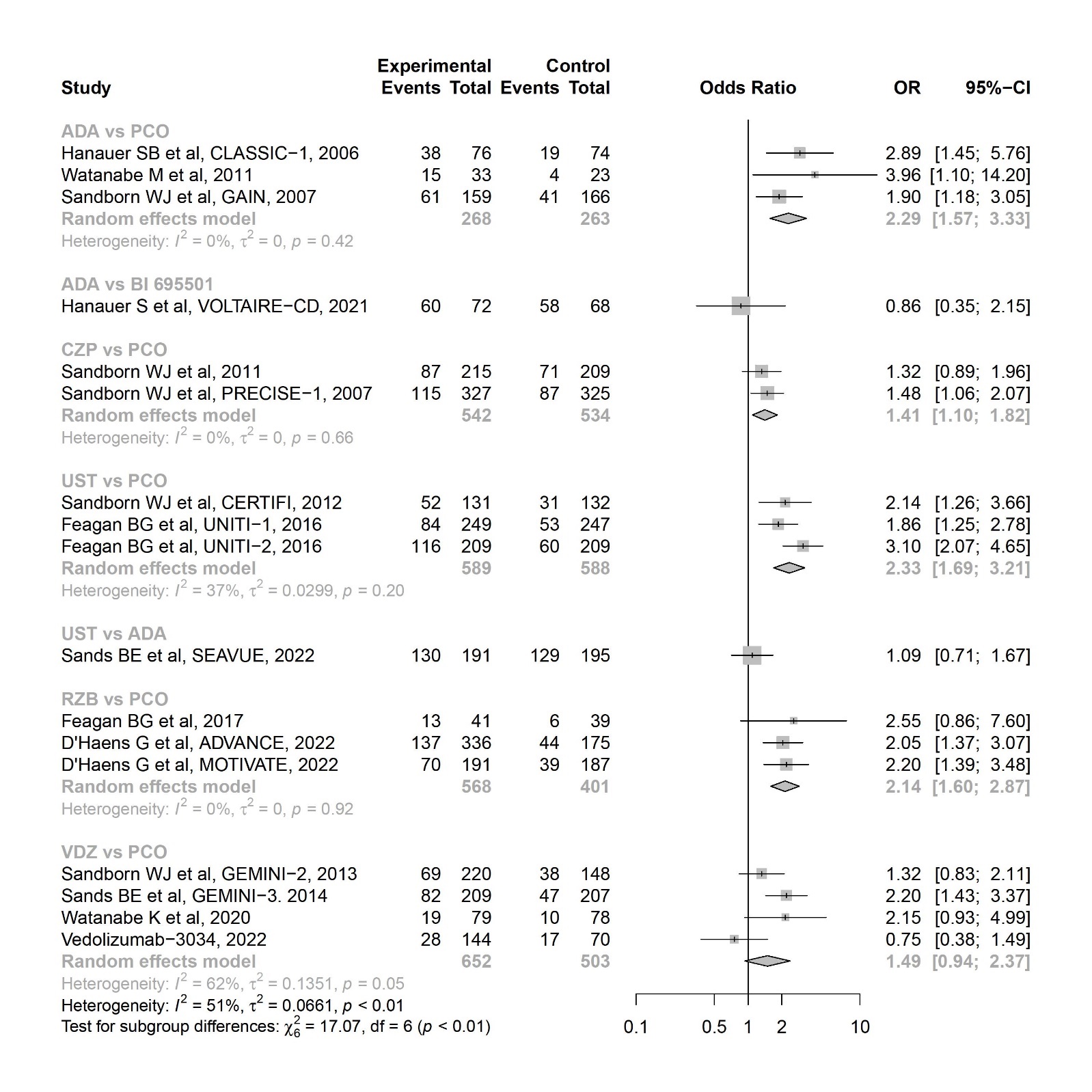


D.


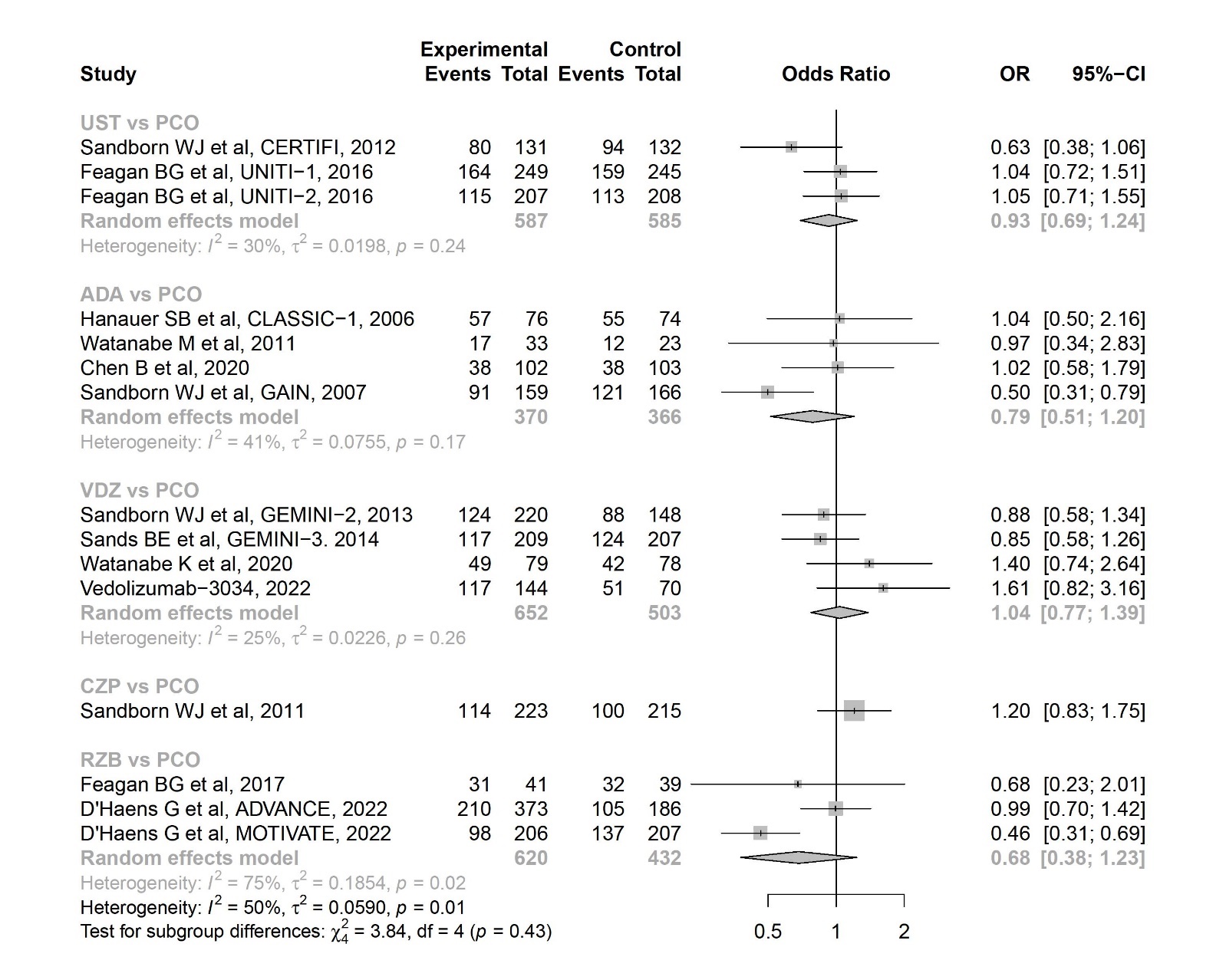


E.


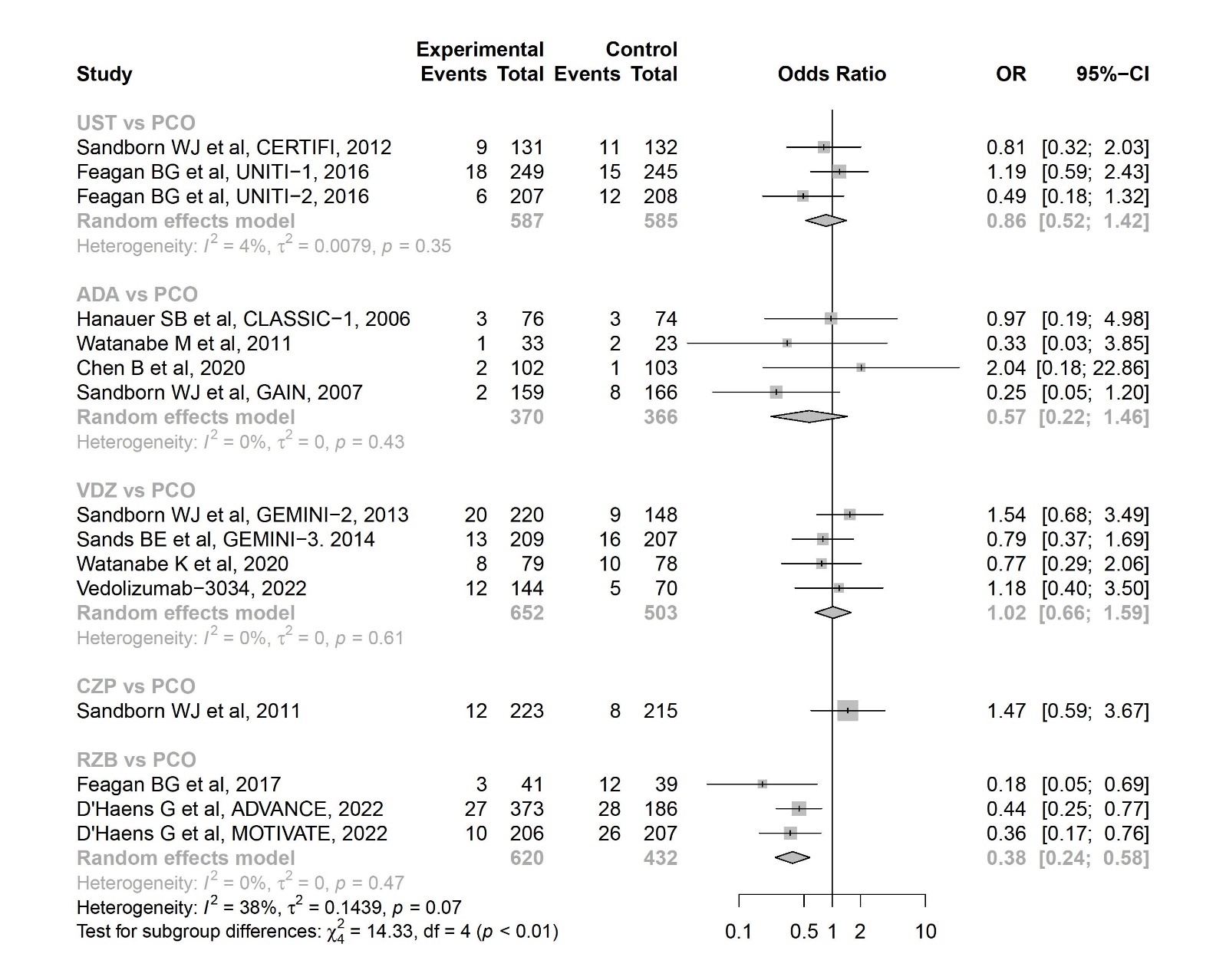


F.


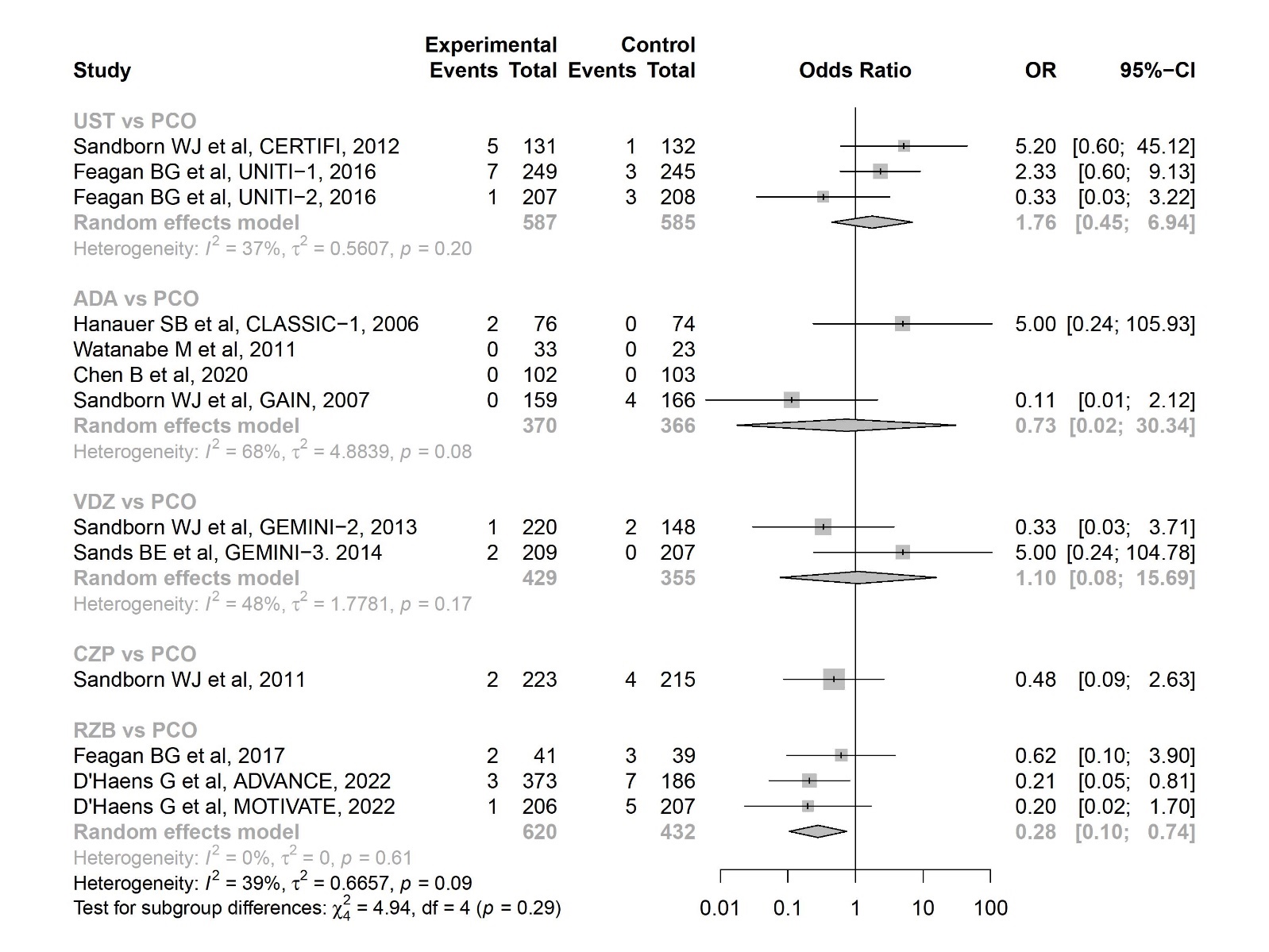


**Supplementary Figure 4.** Forest plots of direct comparisons of (A) maintenance of clinical remission, (B) maintenance of CDAI-70, (C) maintenance of CDAI-100, and (D) risk of adverse events in maintenance therapy, (E) risk of serious adverse events in maintenance therapy, (F) risk of serious infections in maintenance therapy, between included biologic agents in overall patients with moderate-to-severe Crohn’s disease.

A.


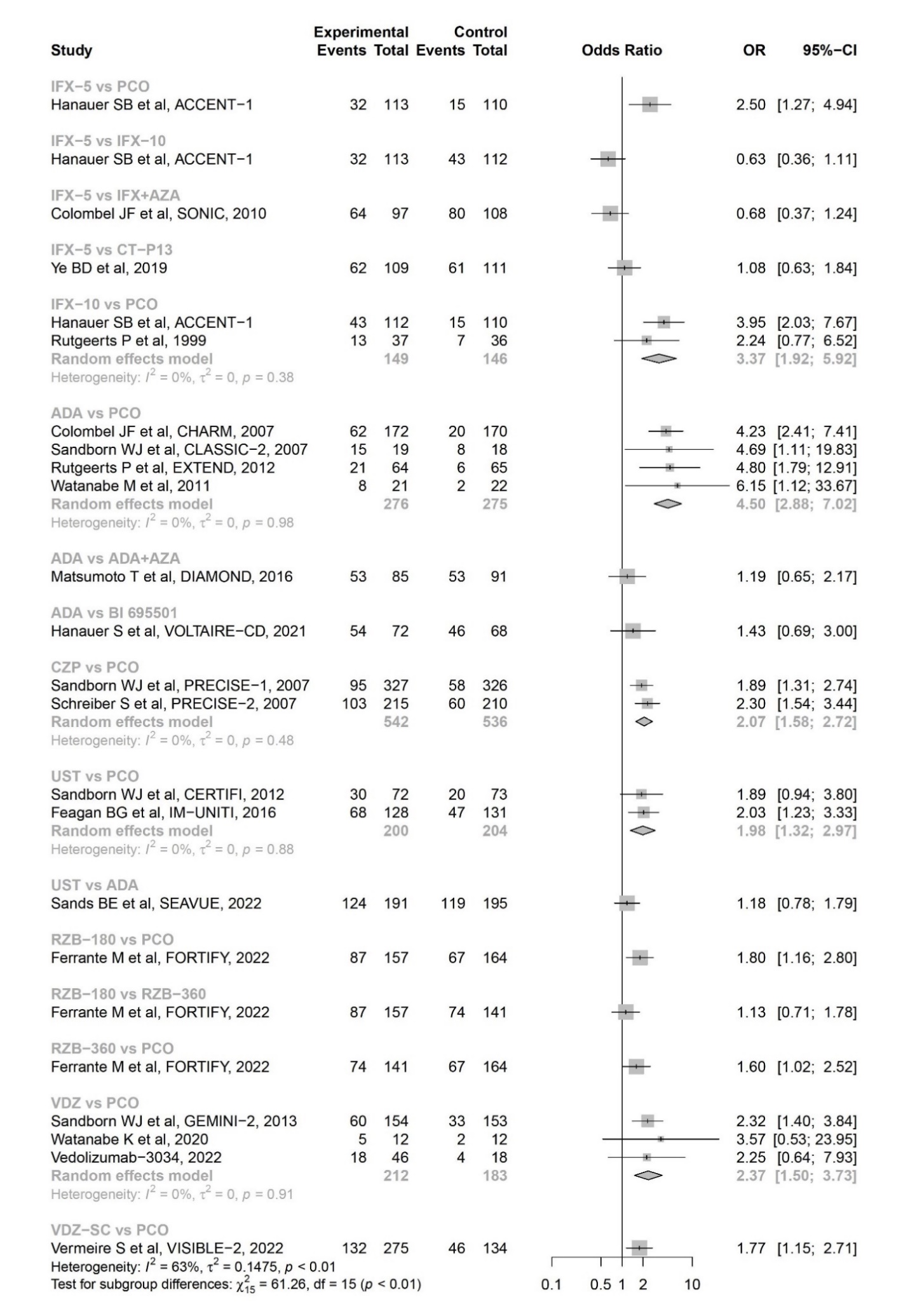


B.


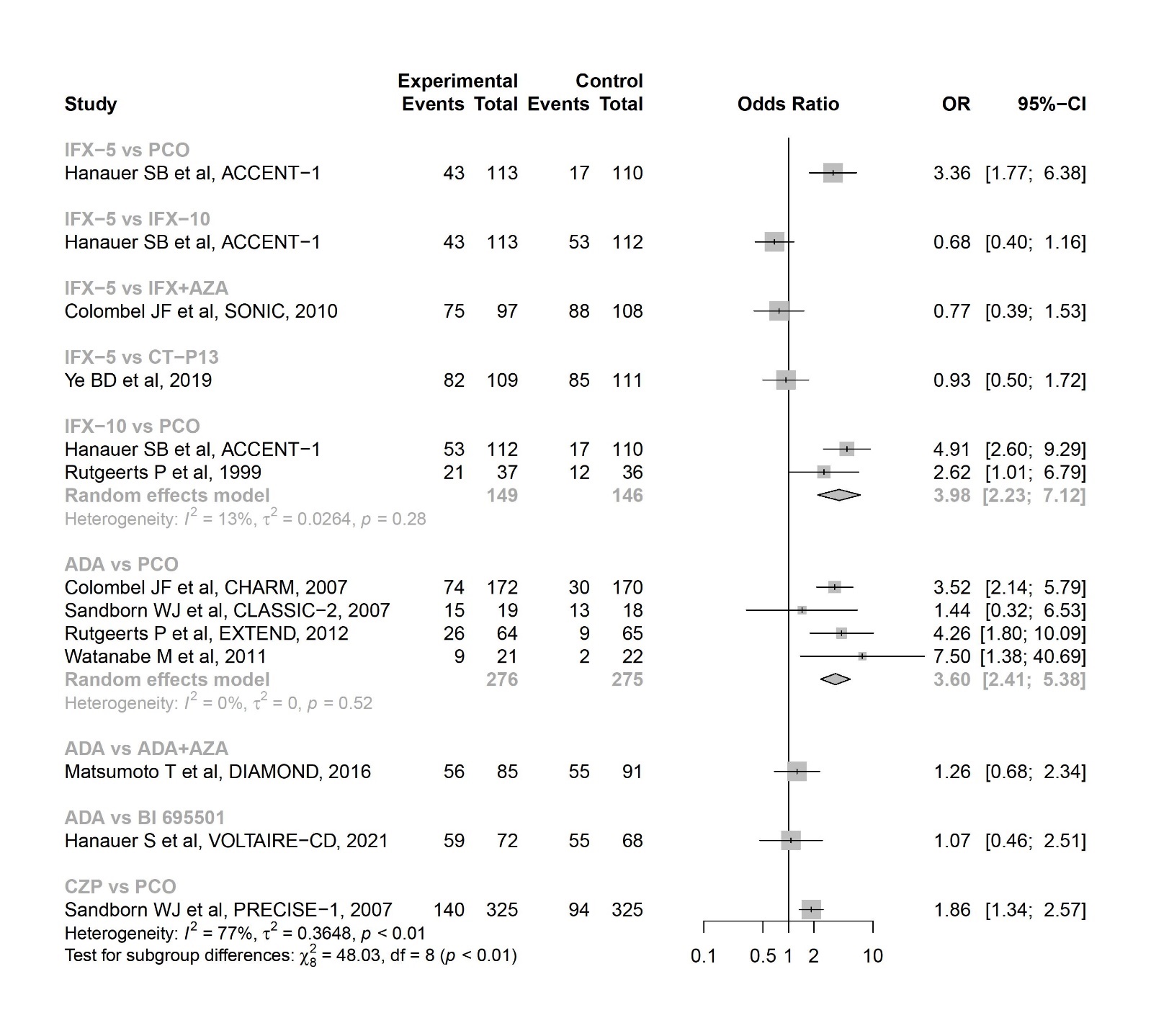


C.


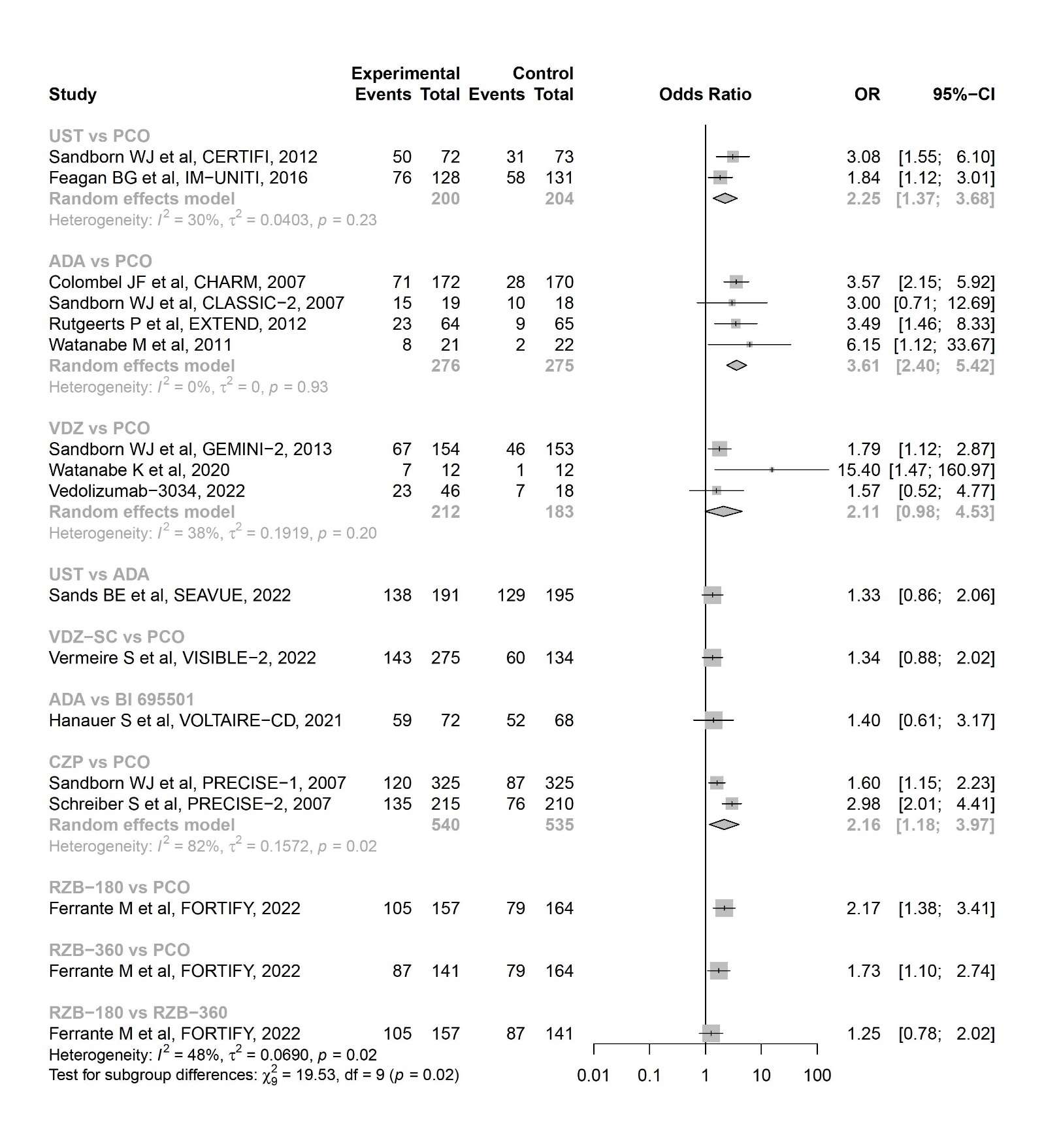


D.


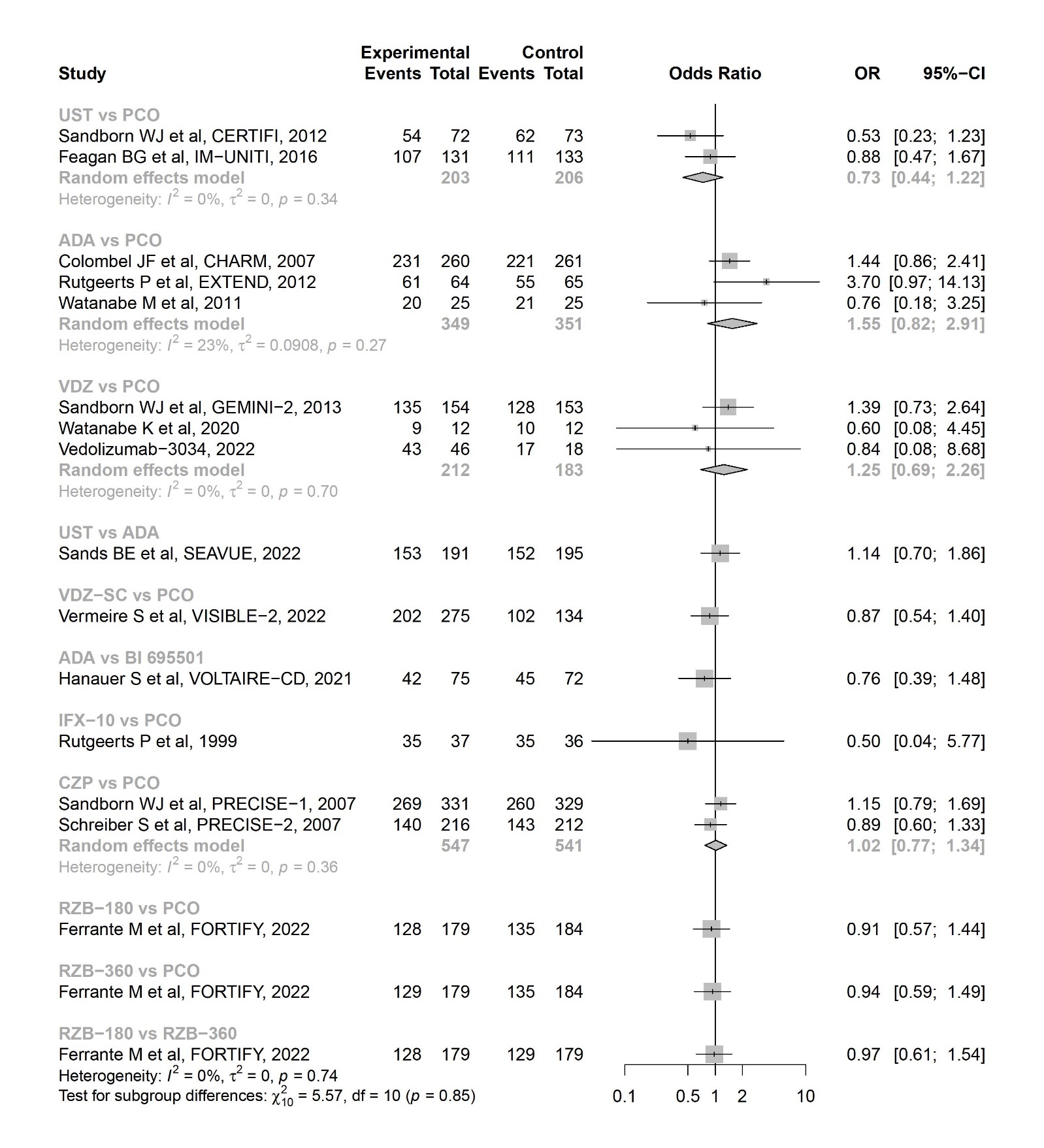


E.


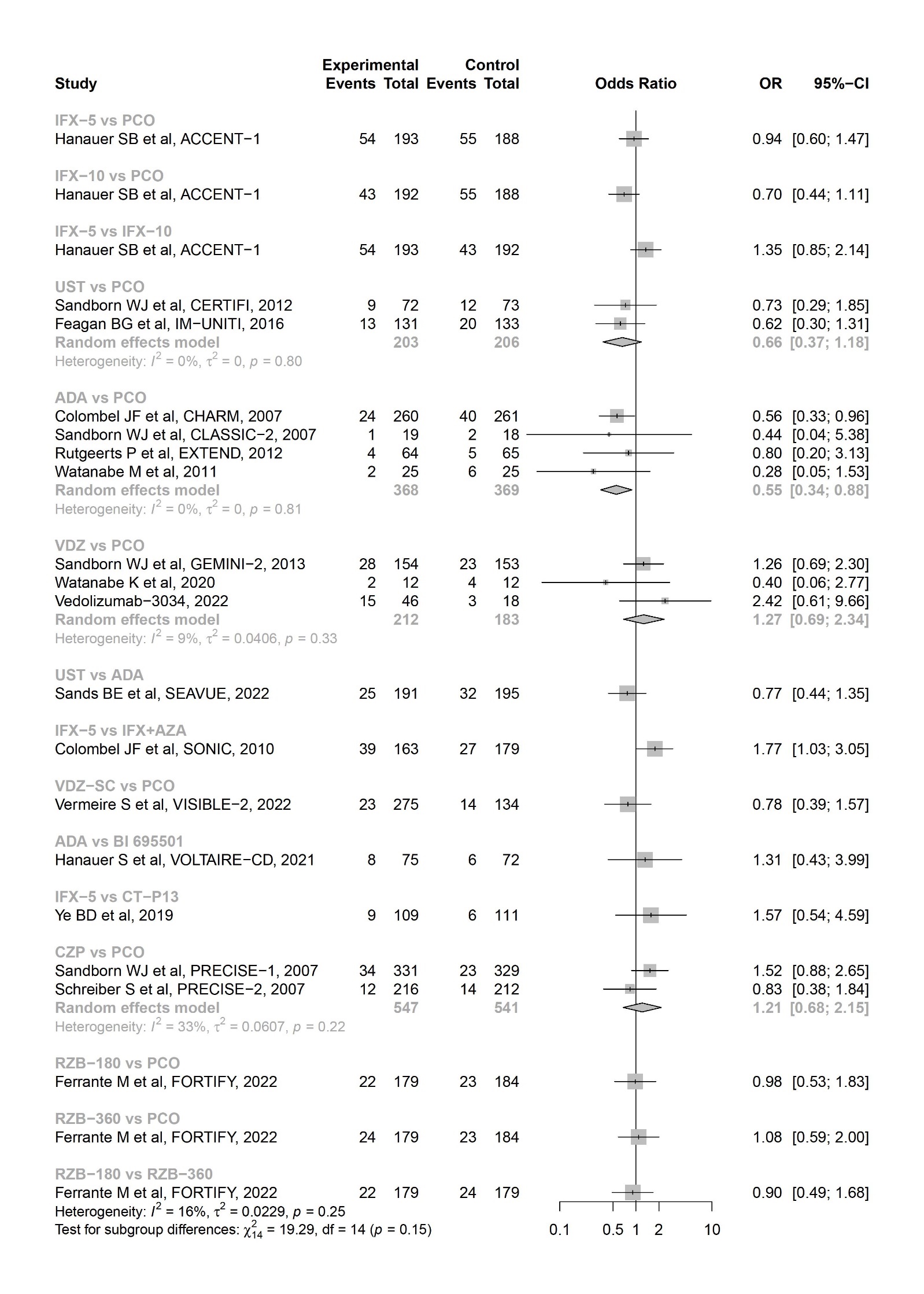


F.


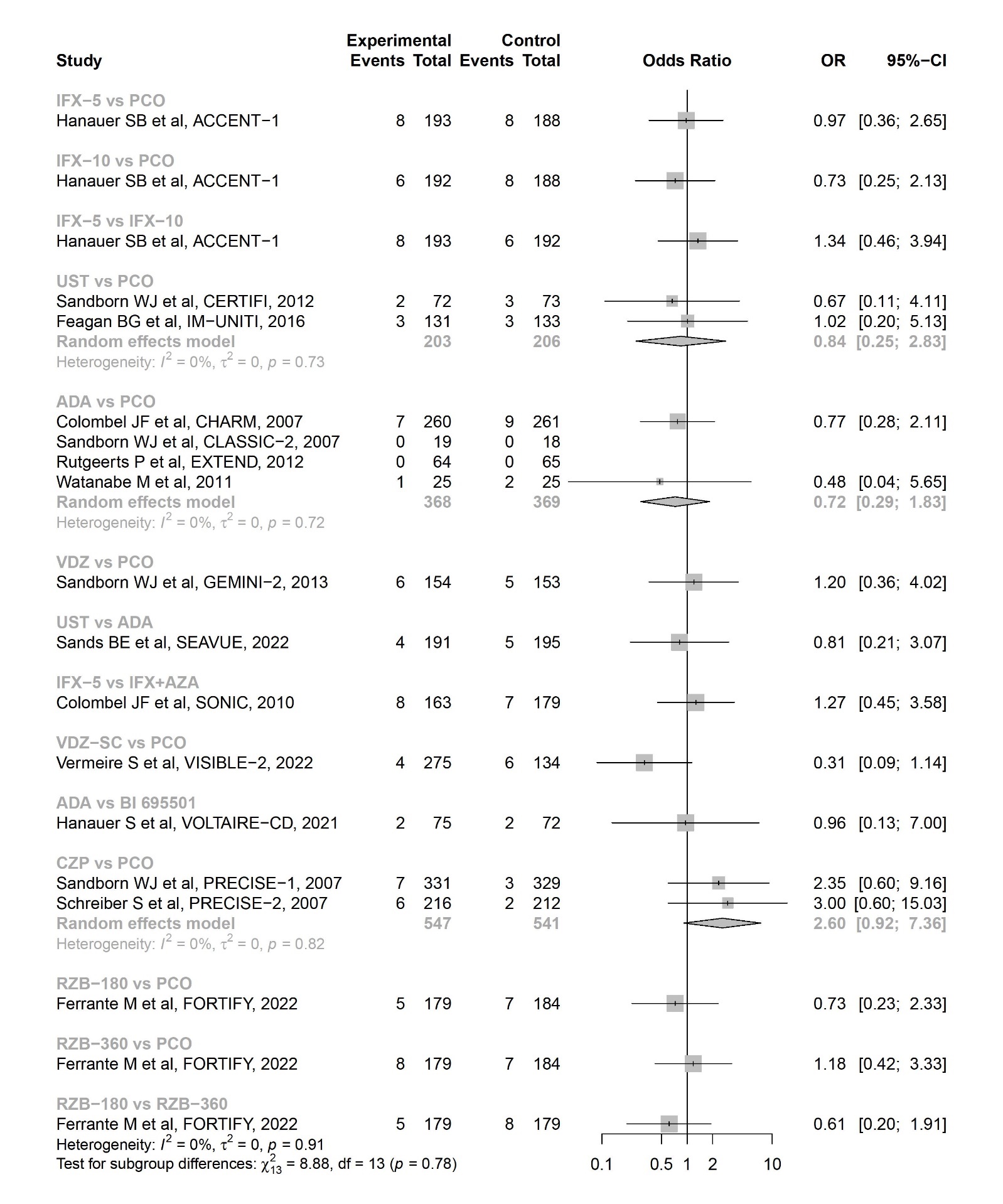


**Supplementary Figure 5.** Forest plots of direct comparisons of (A) induction of clinical remission, (B) induction of CDAI-70, (C) induction of CDAI-100, between included biologic agents in tumor necrosis factor antagonist-naïve patients with moderate-to-severe Crohn’s disease.

A.


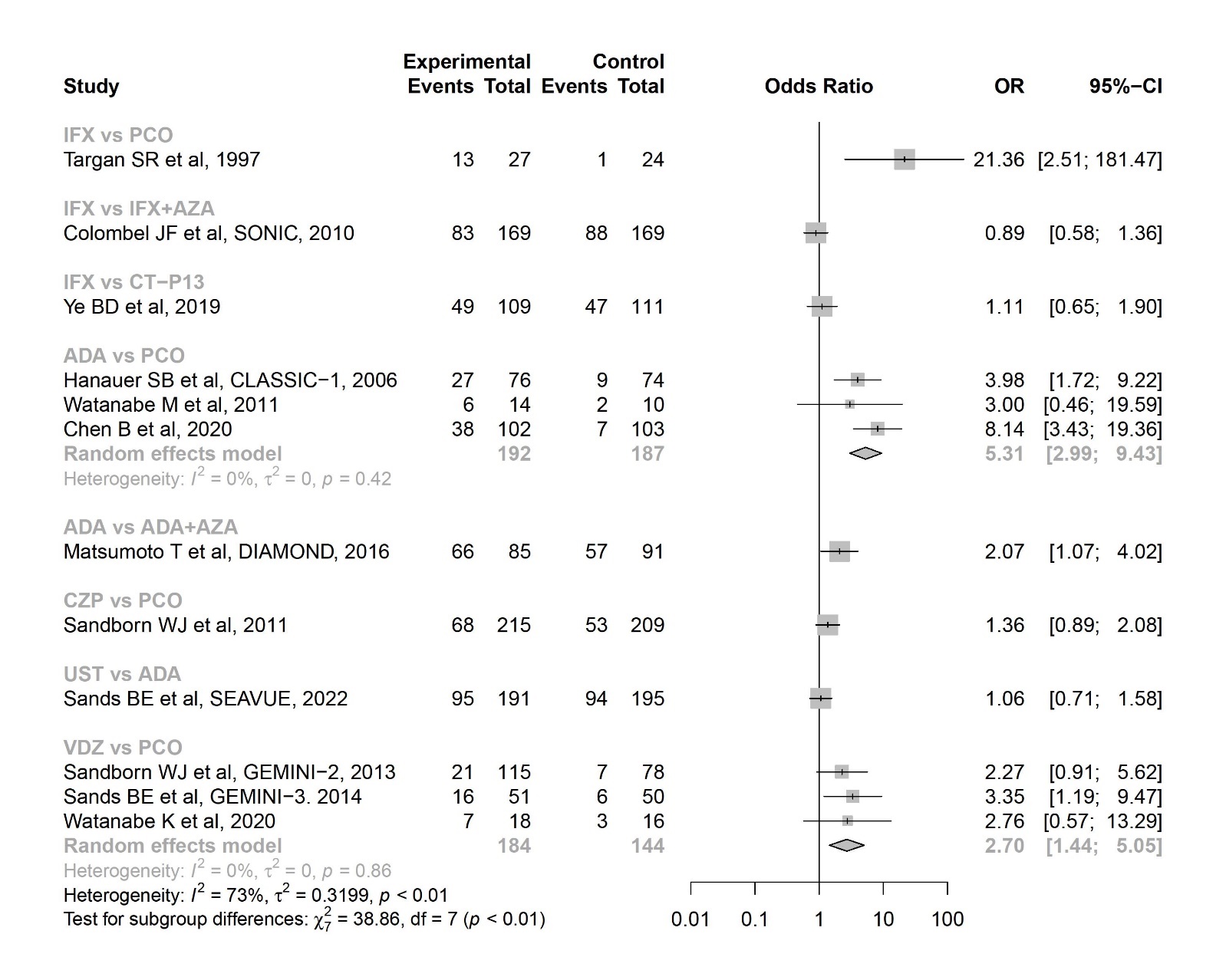


B.


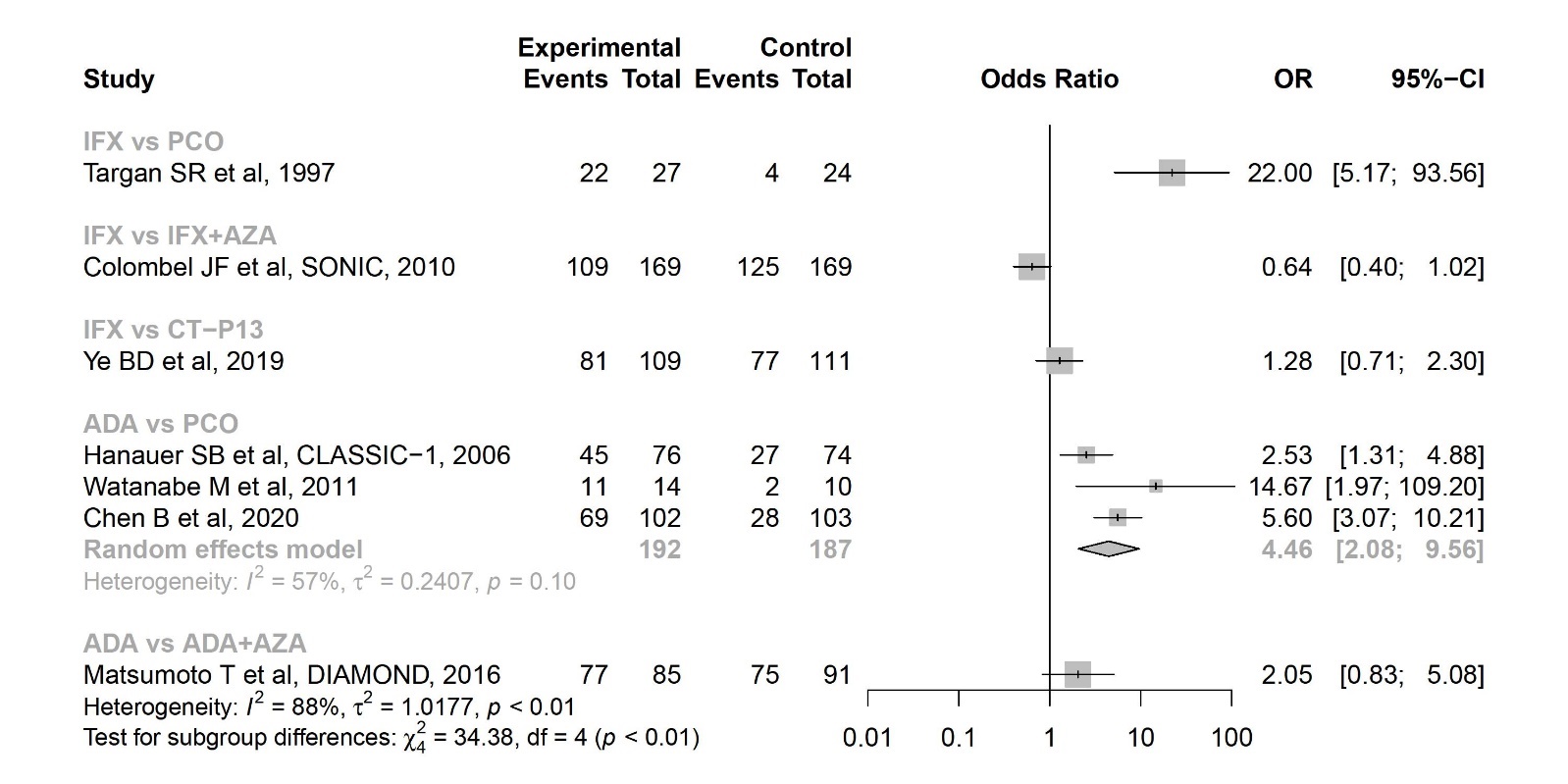


C.


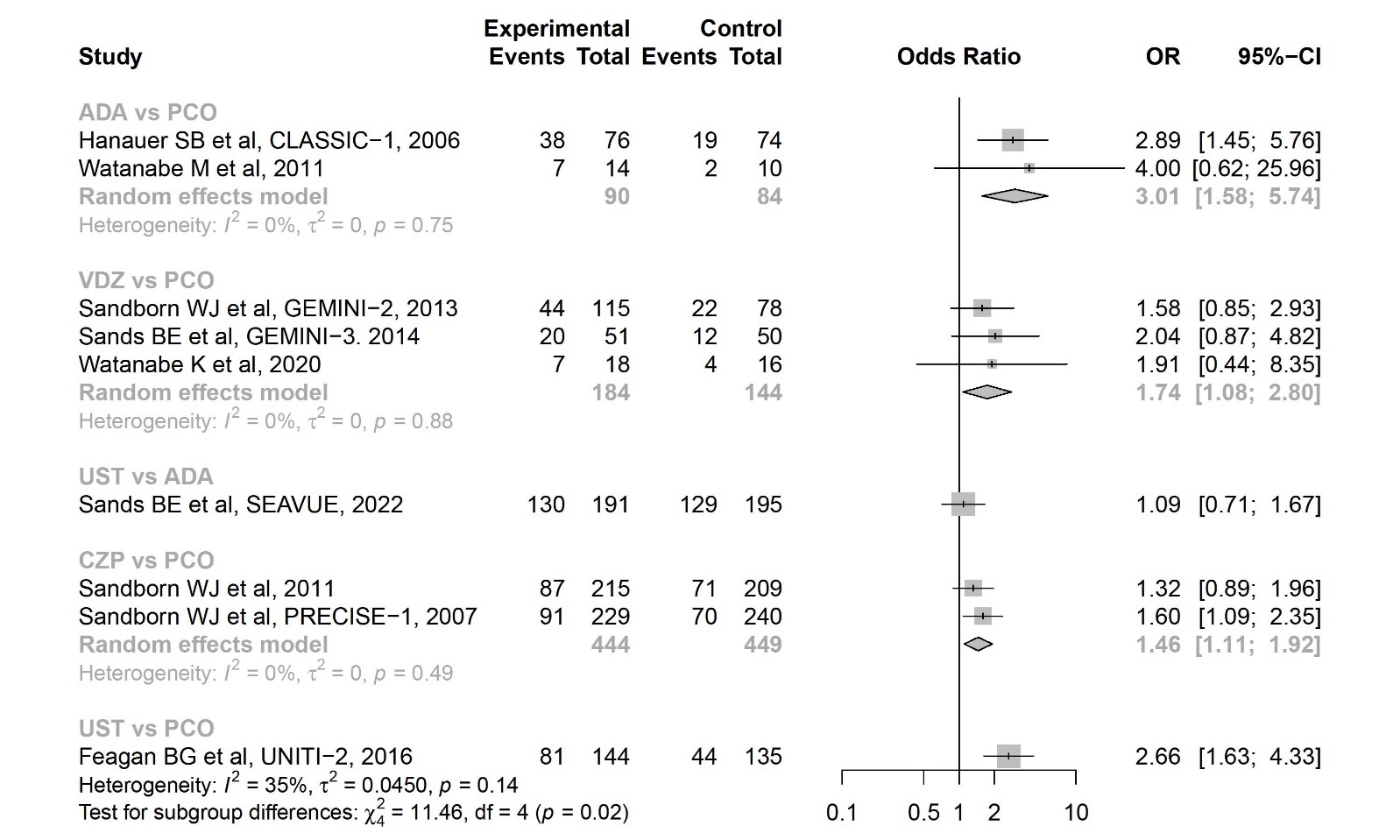


**Supplementary Figure 6.** Forest plots of direct comparisons of (A) induction of clinical remission, (B) induction of CDAI-70, (C) induction of CDAI-100, between included biologic agents in tumor necrosis factor antagonist-experienced patients with moderate-to-severe Crohn’s disease.

A.


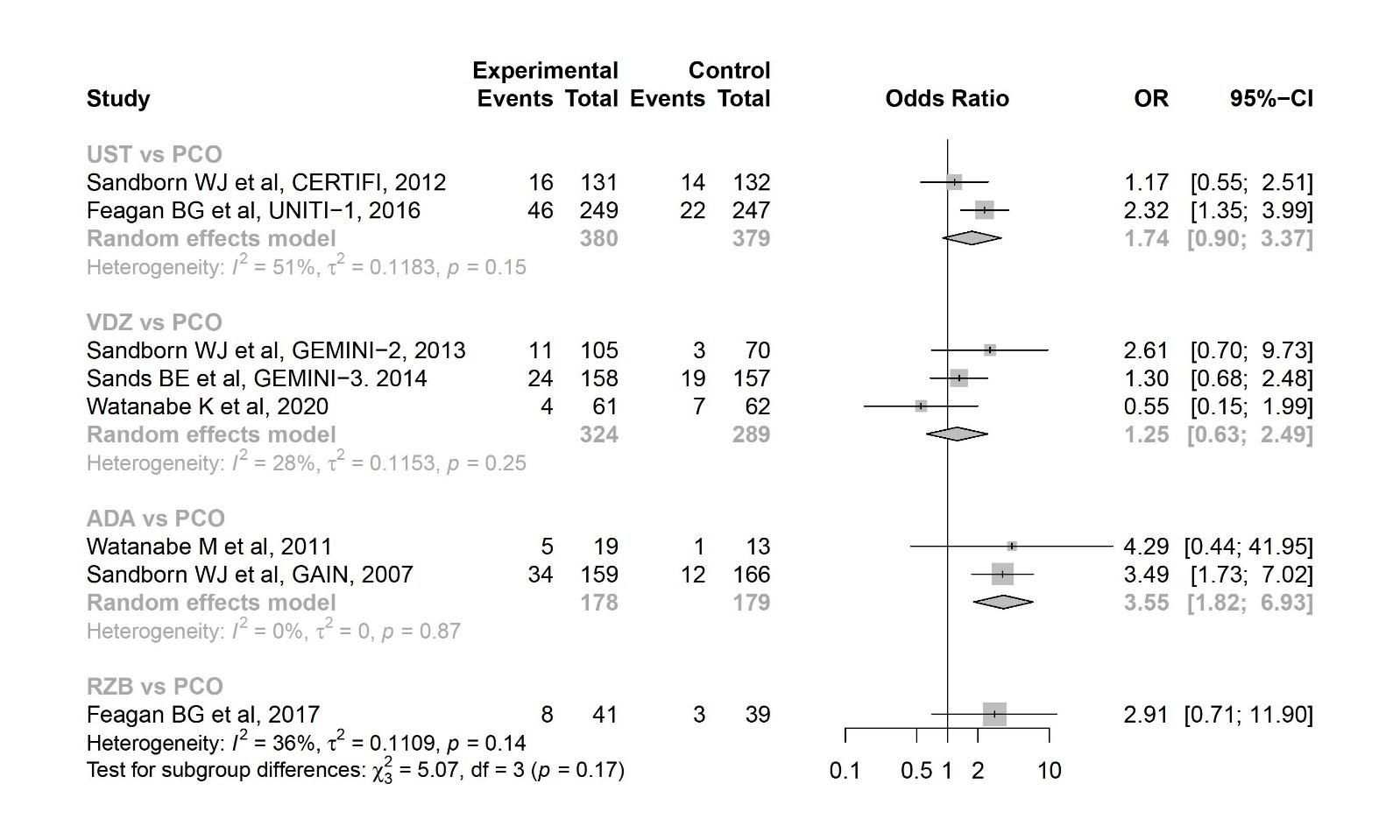


B.


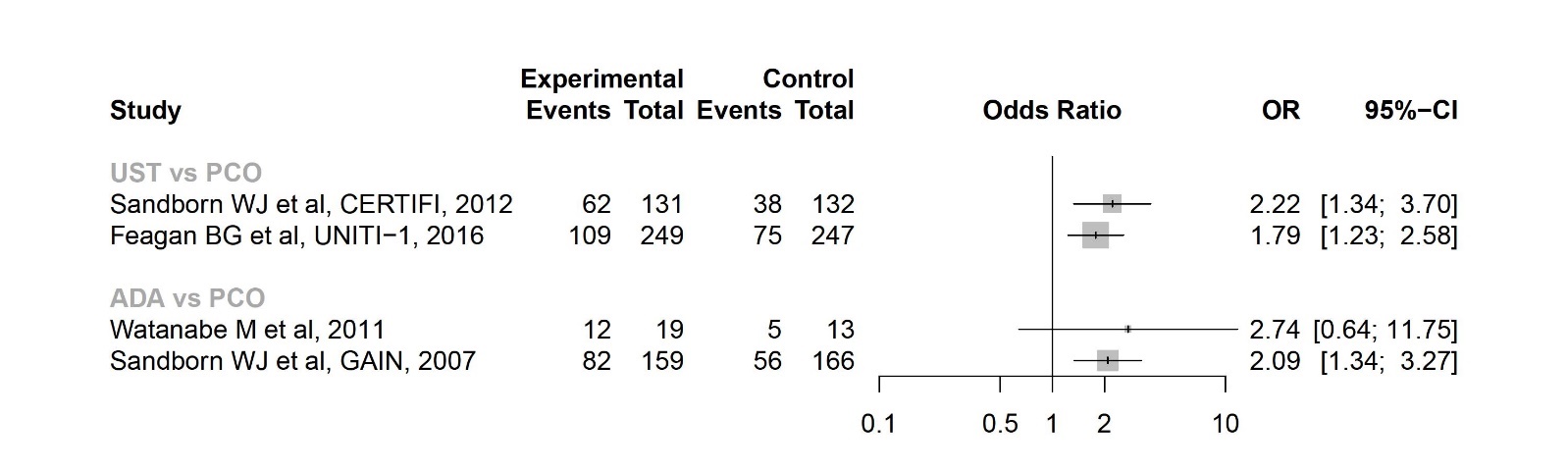


C.


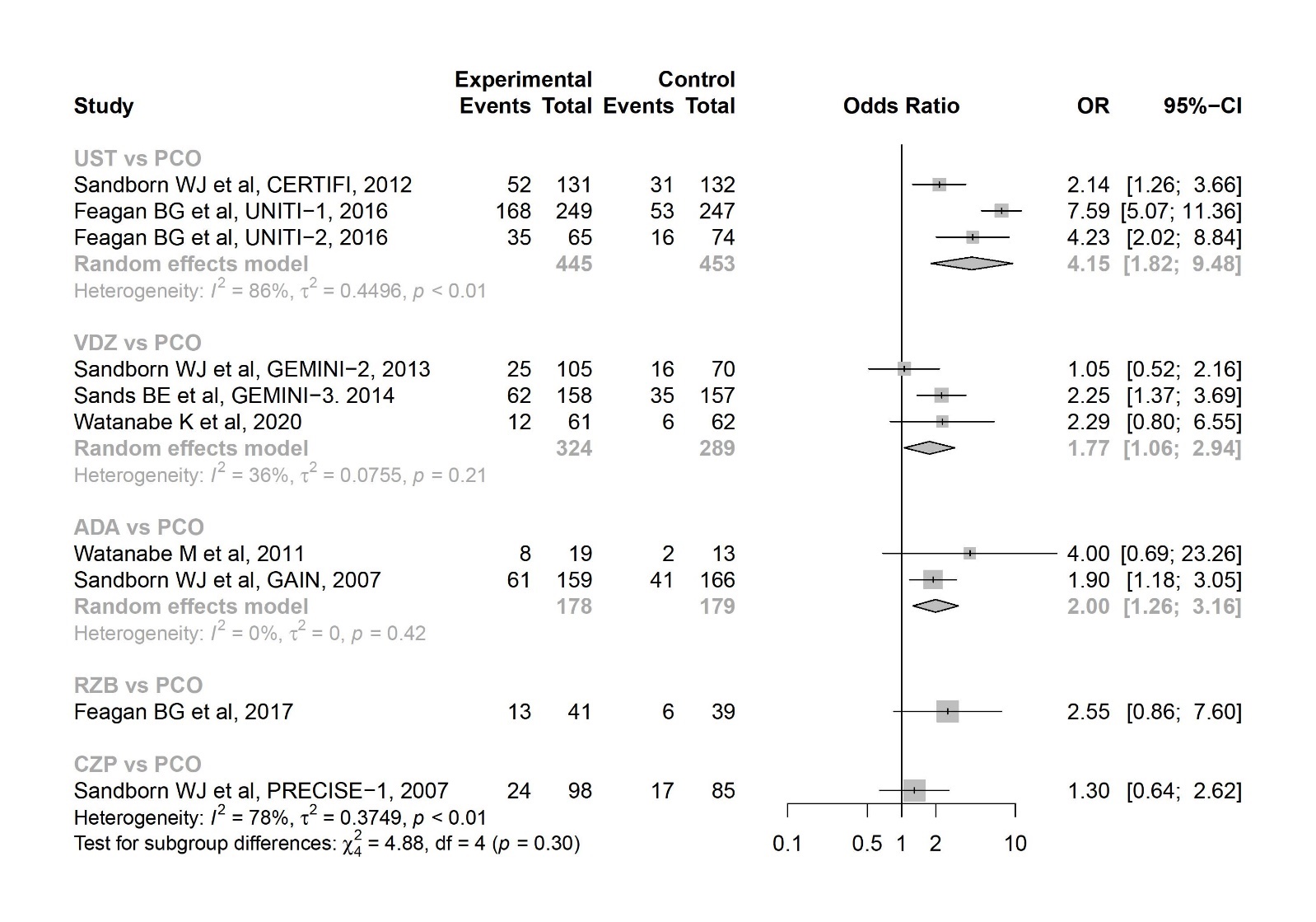


**Supplementary Figure 7.** Forest plots of direct comparisons of maintenance for clinical remission between included biologic agents (A) in tumor necrosis factor antagonist-naïve patients, (B) in tumor necrosis factor antagonist-experienced patients, with moderate-to-severe Crohn’s disease.

A.


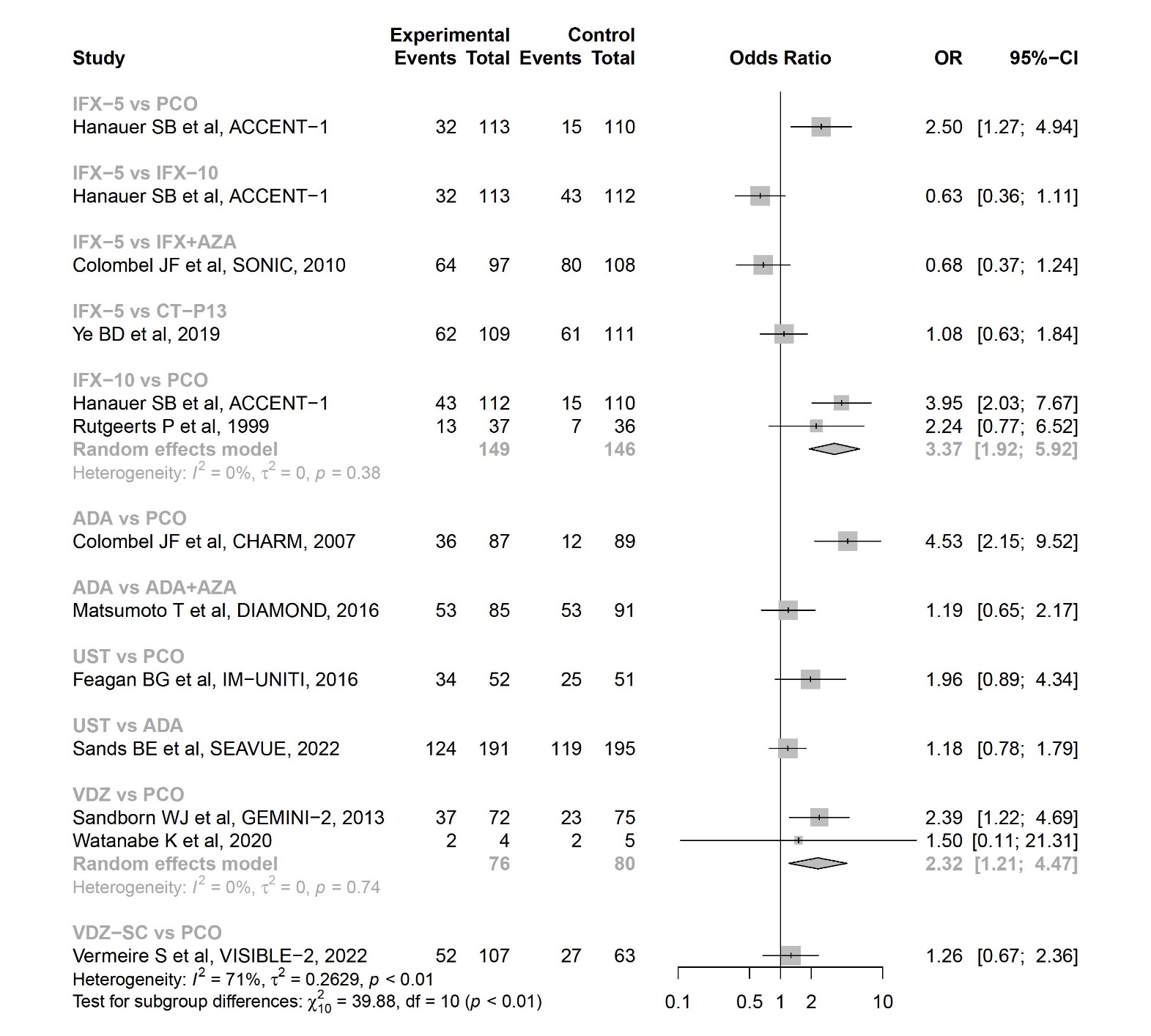


B.


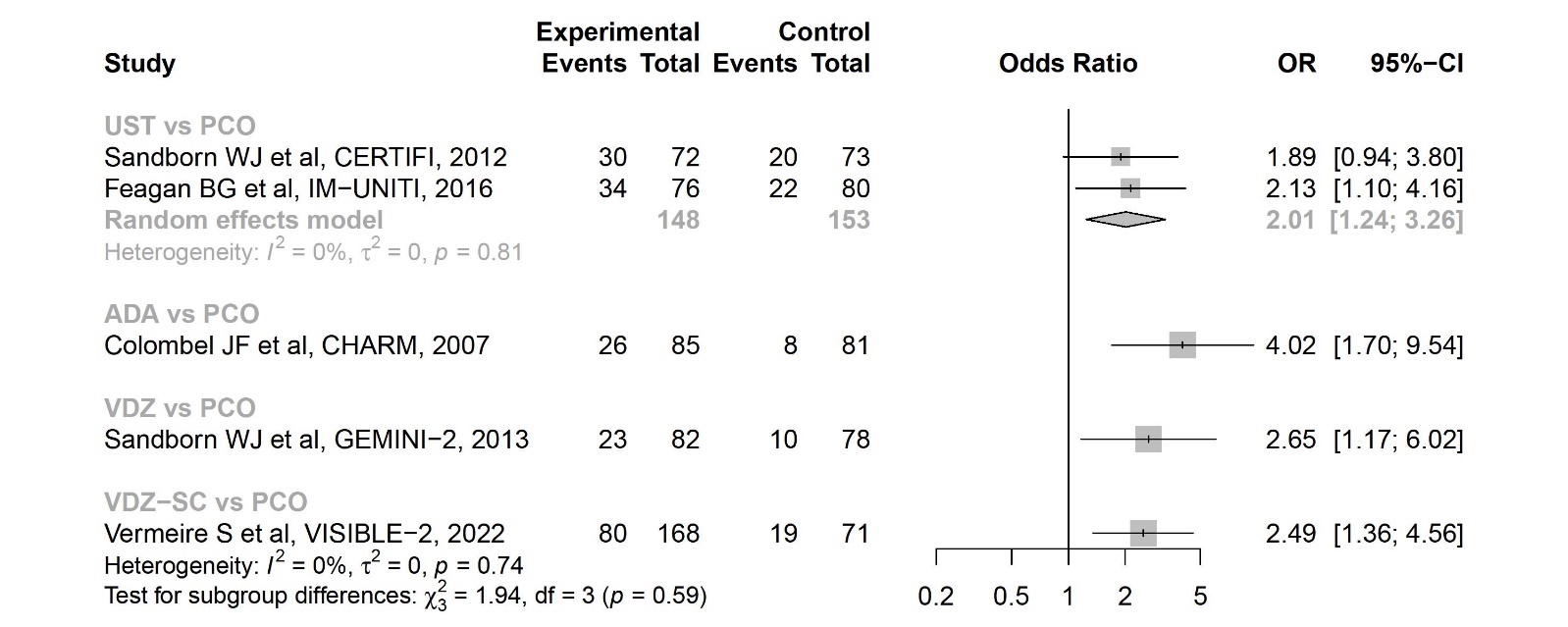


**Supplementary Figure 8.** Funnel plots of assessments of small study and publication bias in evaluation of (A) induction of clinical remission, (C) induction of CDAI-70, (E) induction of CDAI-100, in overall patients with moderate-to-severe Crohn’s disease, and (B) (D) (F) are their respectively plots moderated by including outcomes timepoint as a moderator.


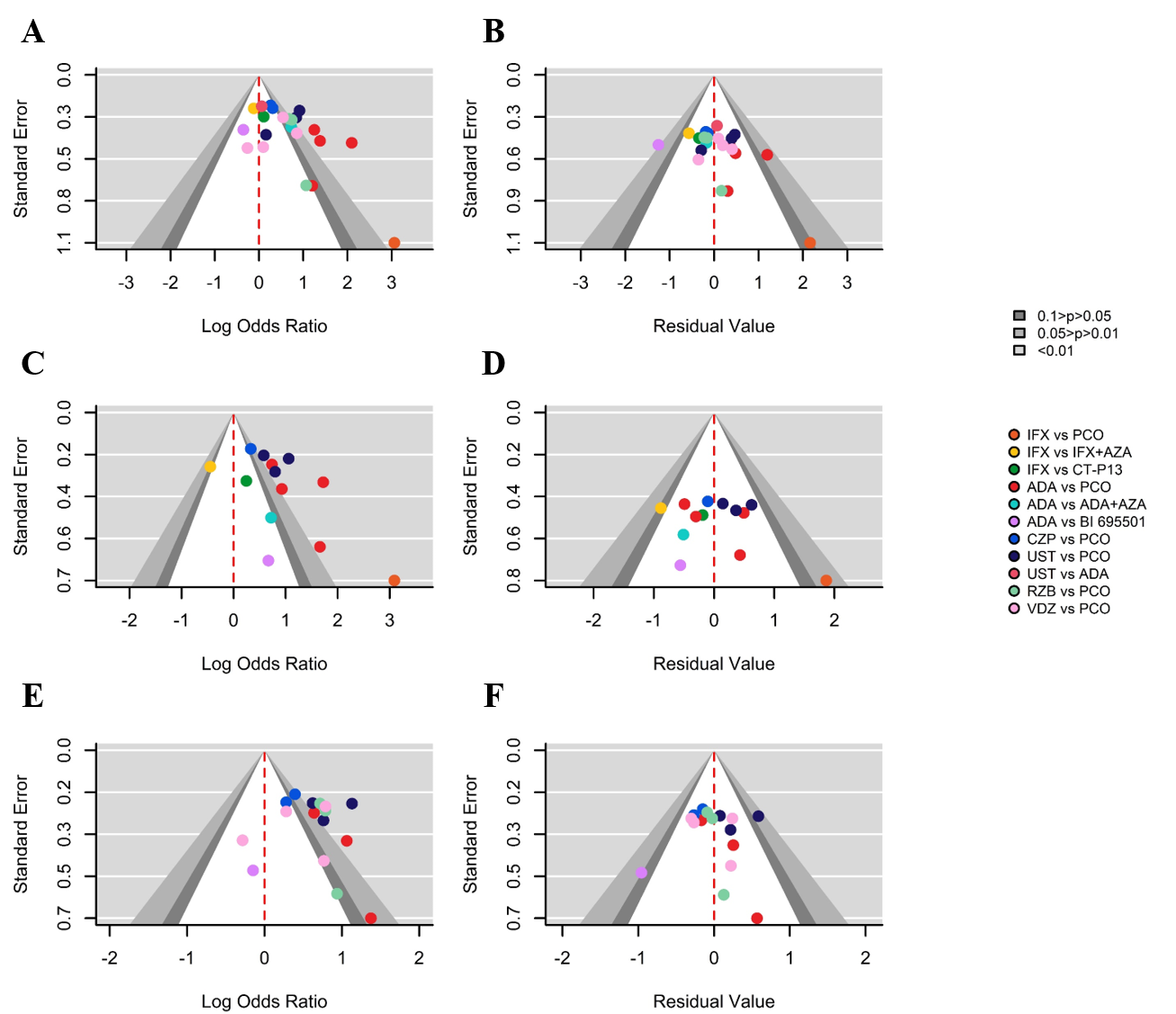


**Supplementary Figure 9.** Funnel plots of assessments of small study and publication bias in evaluation of (A) risk of adverse events in induction therapy, (C) risk of serious adverse events in induction therapy, (E) risk of serious infections in induction therapy, in overall patients with moderate-to-severe Crohn’s disease, and (B) (D) (F) are their respectively plots moderated by including outcomes timepoint as a moderator.


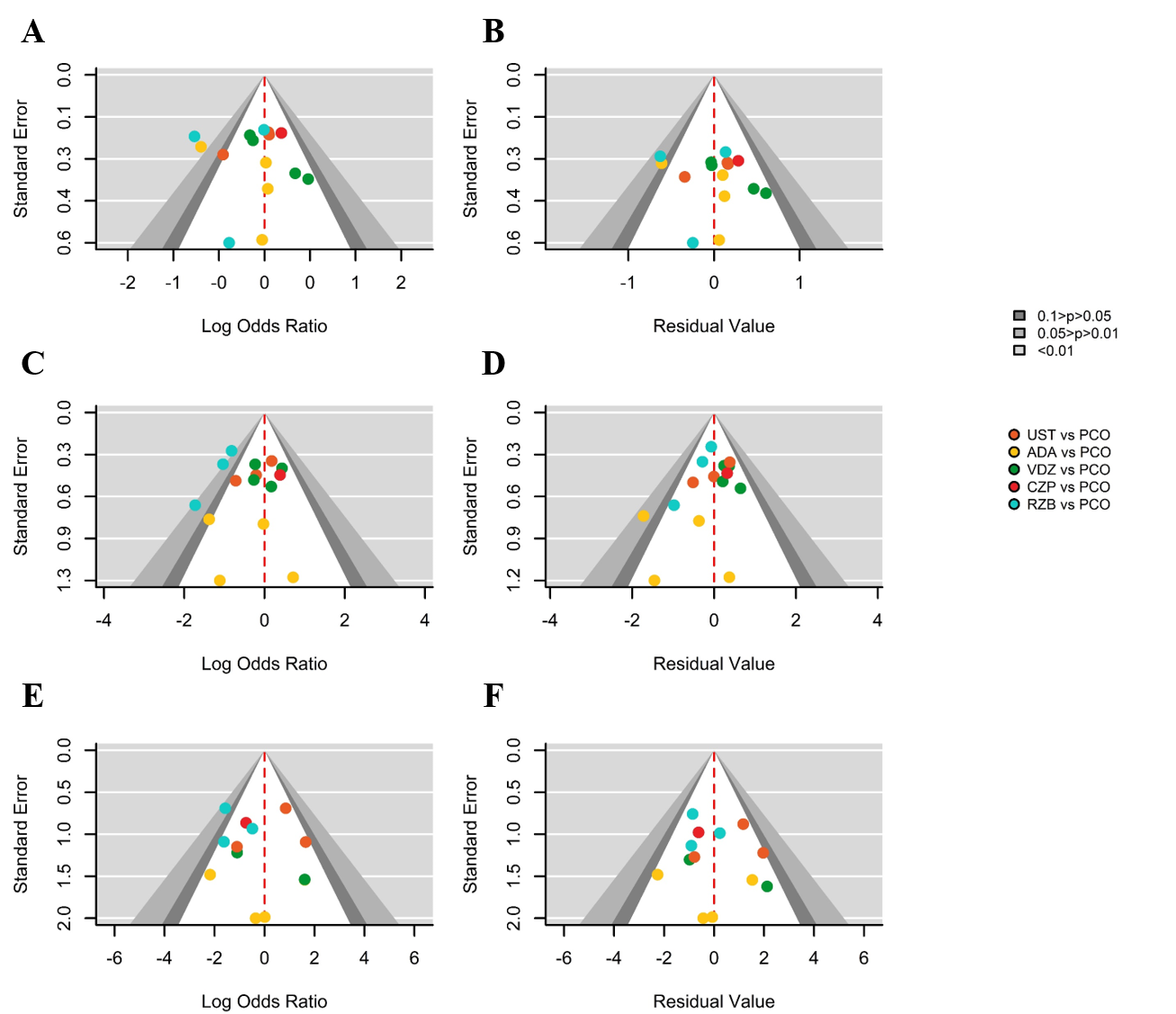


**Supplementary Figure 10.** Funnel plots of assessments of small study and publication bias in evaluation of (A) maintenance of clinical remission, (C) maintenance of CDAI-70, (E) maintenance of CDAI-100, in overall patients with moderate-to-severe Crohn’s disease, and (B) (D) (F) are their respectively plots moderated by including outcomes timepoint as a moderator.


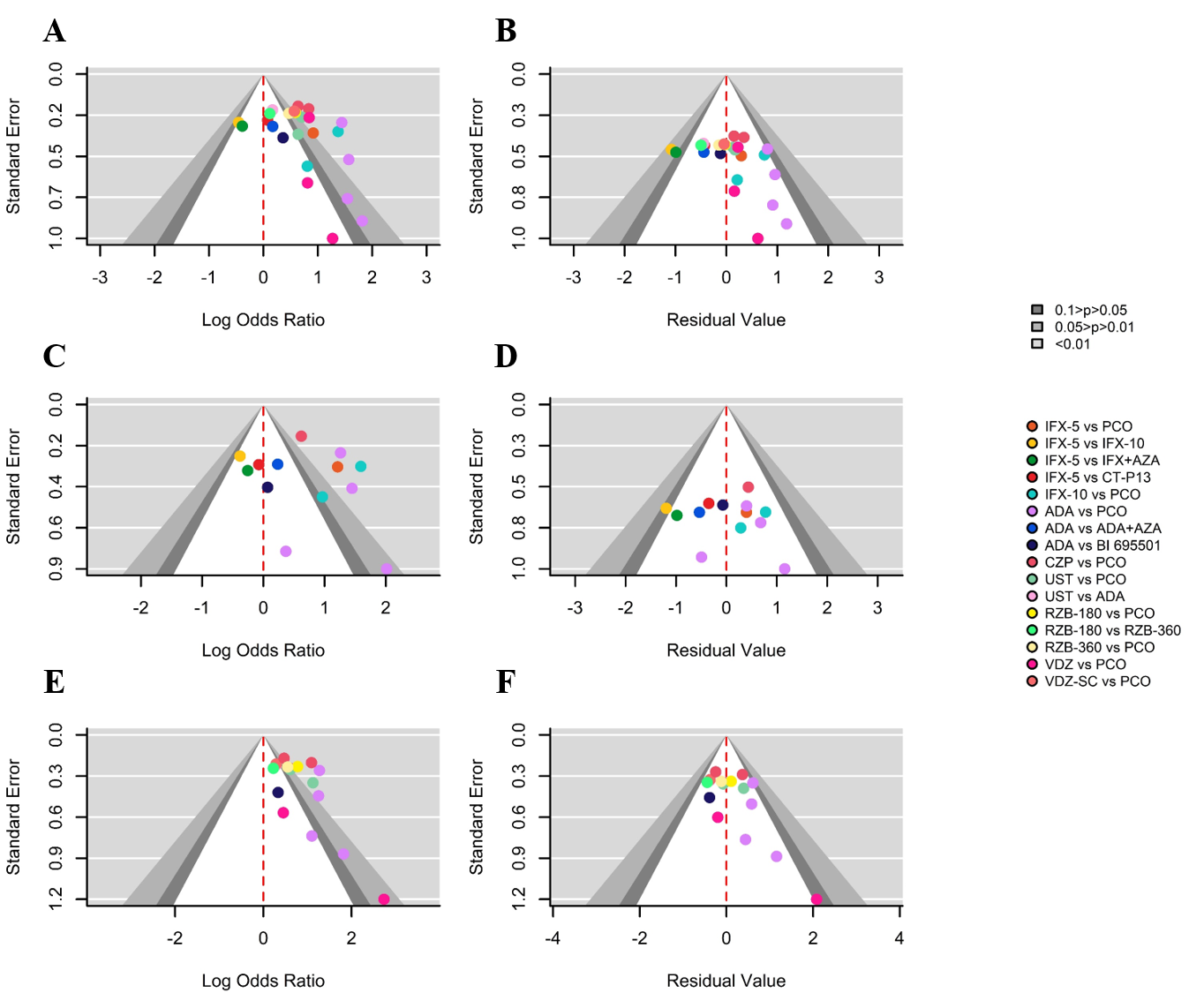


**Supplementary Figure 11.** Funnel plots of assessments of small study and publication bias in evaluation of (A) risk of adverse events in maintenance therapy, (C) risk of serious adverse events in maintenance therapy, (E) risk of serious infections in maintenance therapy, in overall patients with moderate-to-severe Crohn’s disease, and (B) (D) (F) are their respectively plots moderated by including outcomes timepoint as a moderator.


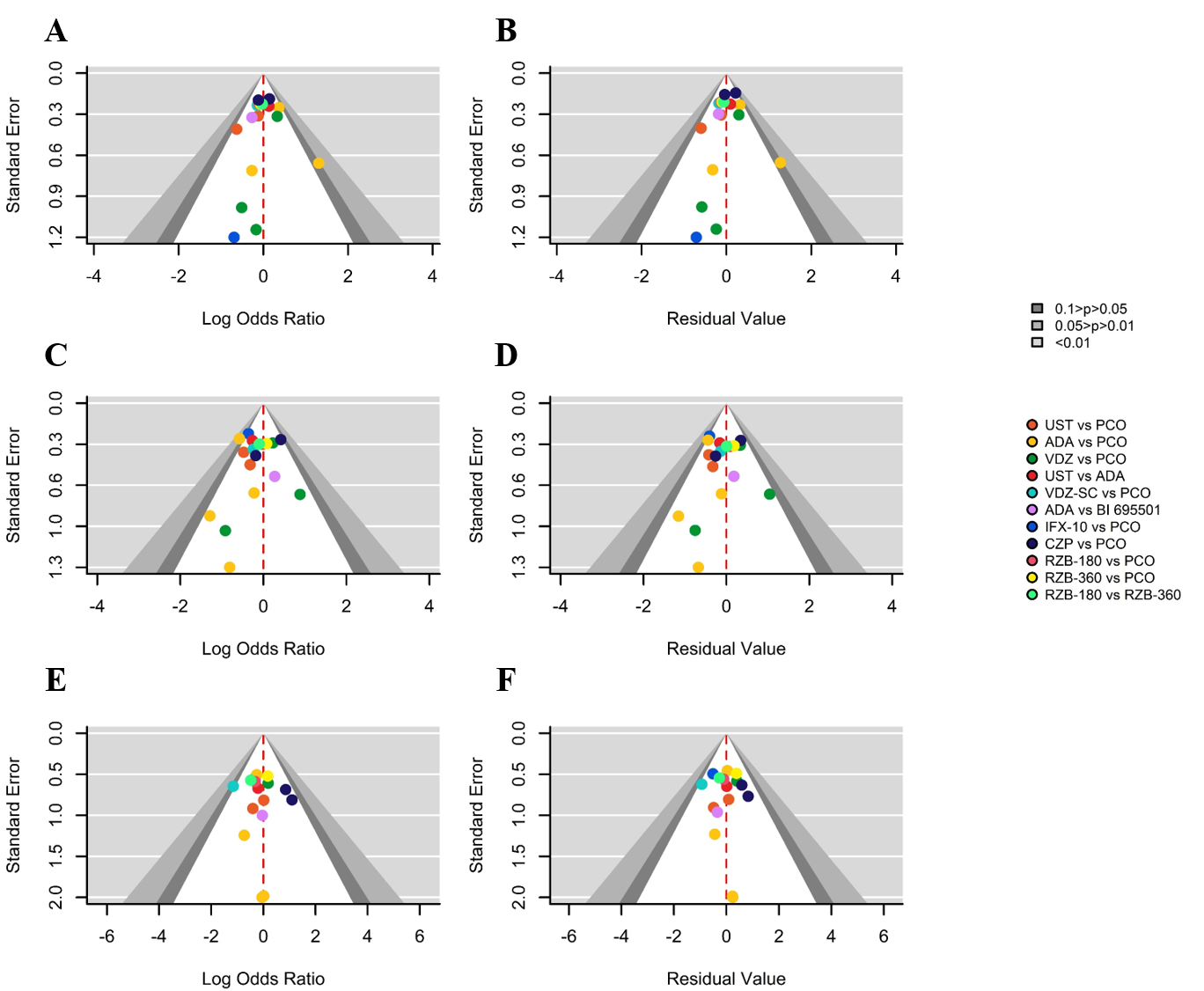


**Supplementary Figure 12.** Funnel plots of assessments of small study and publication bias in evaluation of (A) induction of clinical remission, (C) induction of CDAI-70, (E) induction of CDAI-100, in tumor necrosis factor antagonist-naïve patients with moderate-to-severe Crohn’s disease, and (B) (D) (F) are their respectively plots moderated by including outcomes timepoint as a moderator.


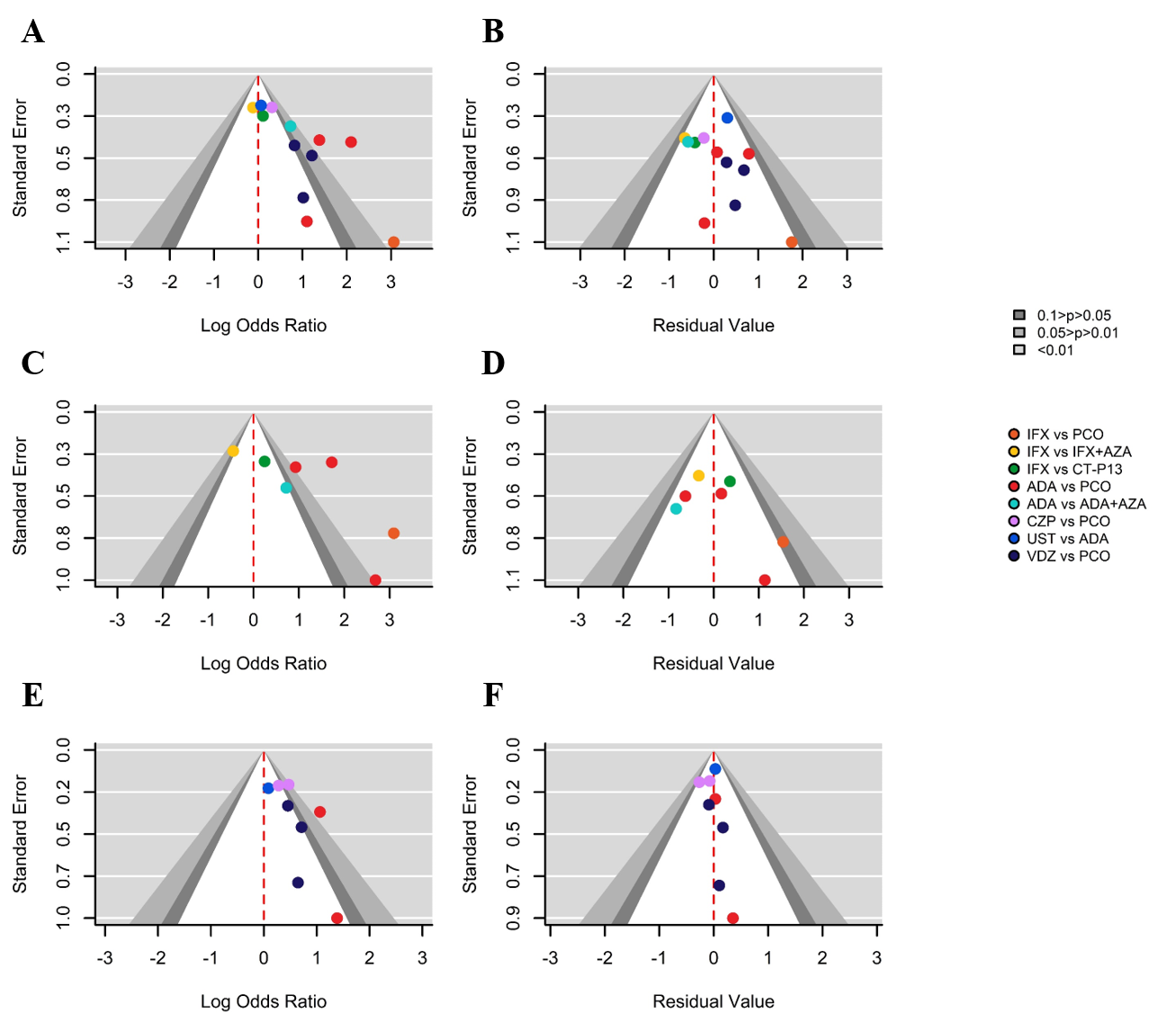


**Supplementary Figure 13.** Funnel plots of assessments of small study and publication bias in evaluation of (A) induction of clinical remission, (C) induction of CDAI-70, (E) induction of CDAI-100, in tumor necrosis factor antagonist-experienced patients with moderate-to-severe Crohn’s disease, and (B) (D) (F) are their respectively plots moderated by including outcomes timepoint as a moderator.


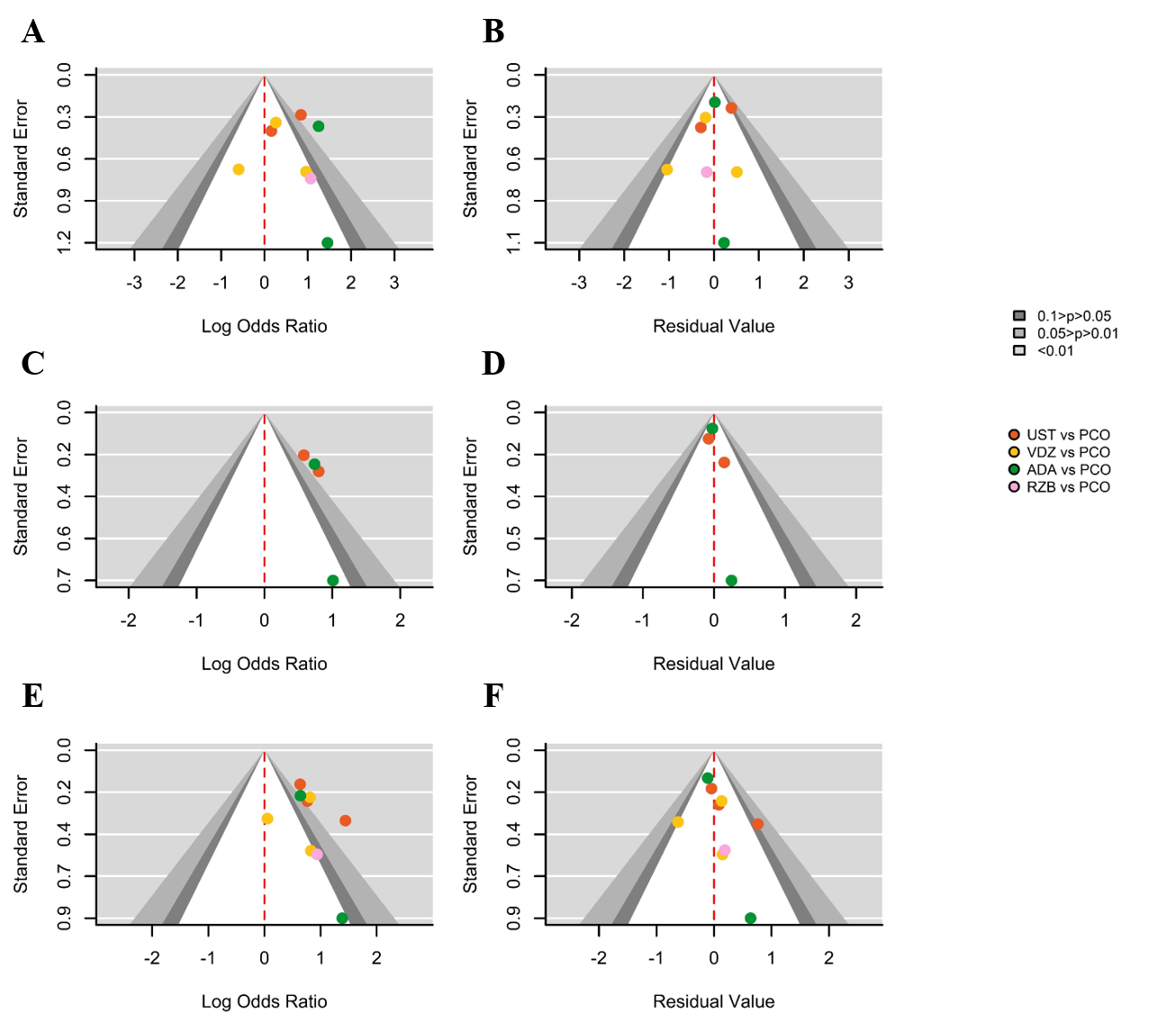


**Supplementary Figure 14.** Funnel plots of assessments of small study and publication bias in evaluation of maintenance of clinical remission (A) in tumor necrosis factor antagonist-naïve patients, (C) in tumor necrosis factor antagonist-experienced patients, with moderate-to-severe Crohn’s disease, and (B) (D) are their respectively plots moderated by including outcomes timepoint as a moderator.


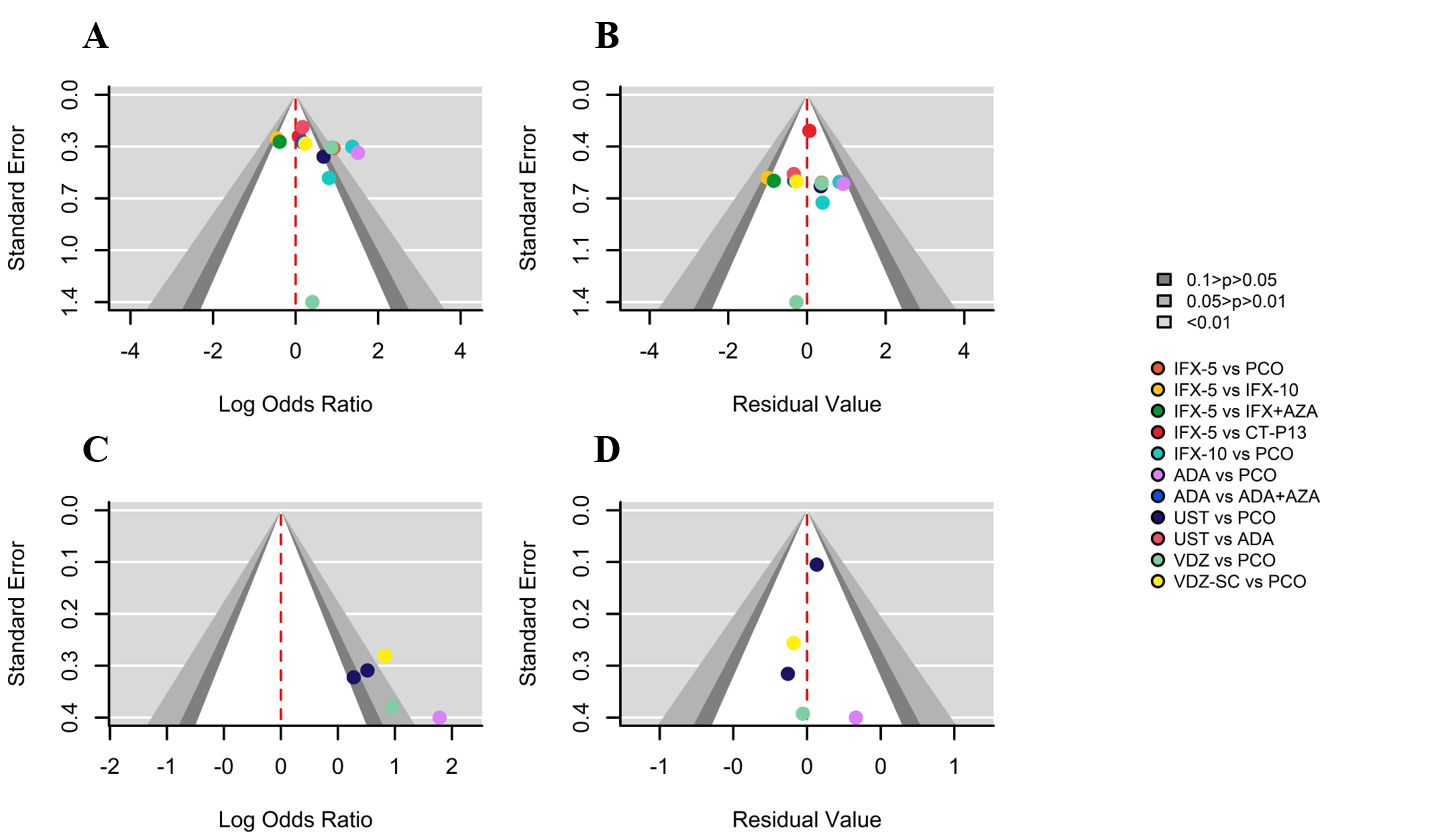


**Supplementary Figure 15.** Leverage plots of assessing model fit of evaluations of (A) induction of clinical remission, (B) induction of CDAI-70, (C) induction of CDAI-100, (D) risk of adverse events in induction therapy, (E) risk of serious adverse events in induction therapy, in overall patients with moderate-to-severe Crohn’s disease.


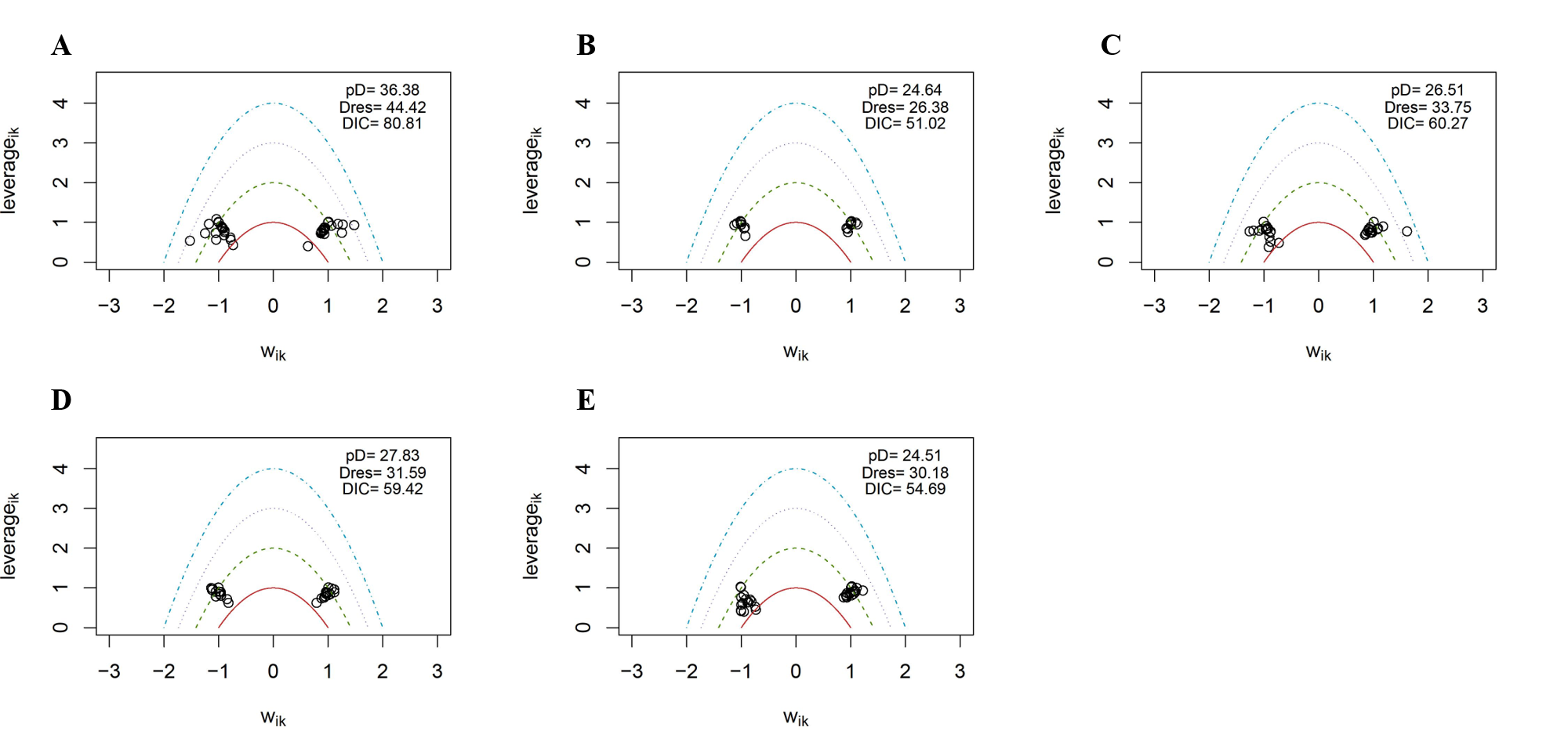


**Supplementary Figure 16.** Leverage plots of assessing model fit of evaluations of (A) maintenance of clinical remission, (B) maintenance of CDAI-70, (C) maintenance of CDAI-100, (D) risk of adverse events in maintenance therapy, (E) risk of serious adverse events in maintenance therapy, in overall patients with moderate-to-severe Crohn’s disease.


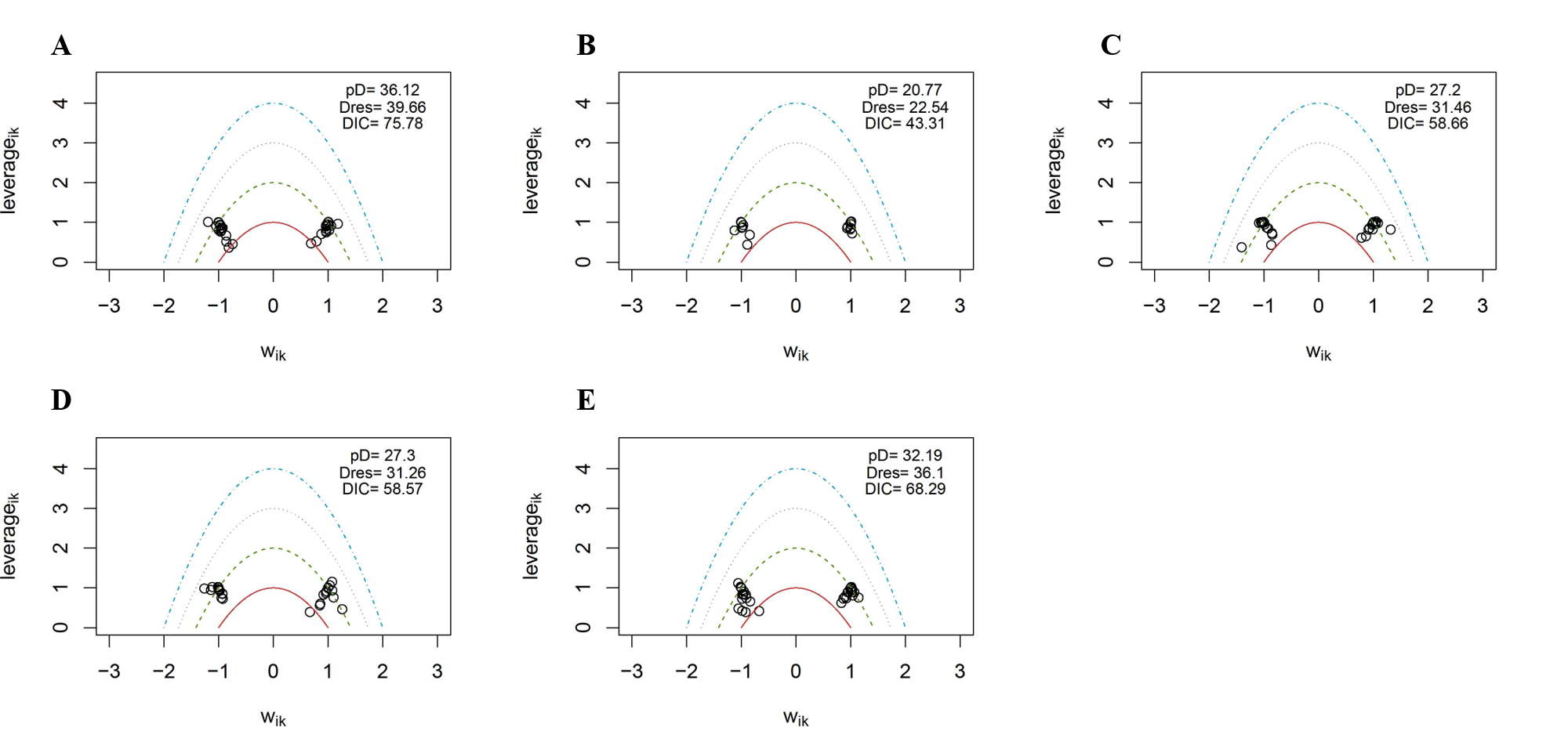


**Supplementary Figure 17.** Leverage plots of assessing model fit of evaluations of (A) induction of clinical remission, (B) induction of CDAI-70, (C) induction of CDAI-100, in tumor necrosis factor antagonist-naïve patients with moderate-to-severe Crohn’s disease.


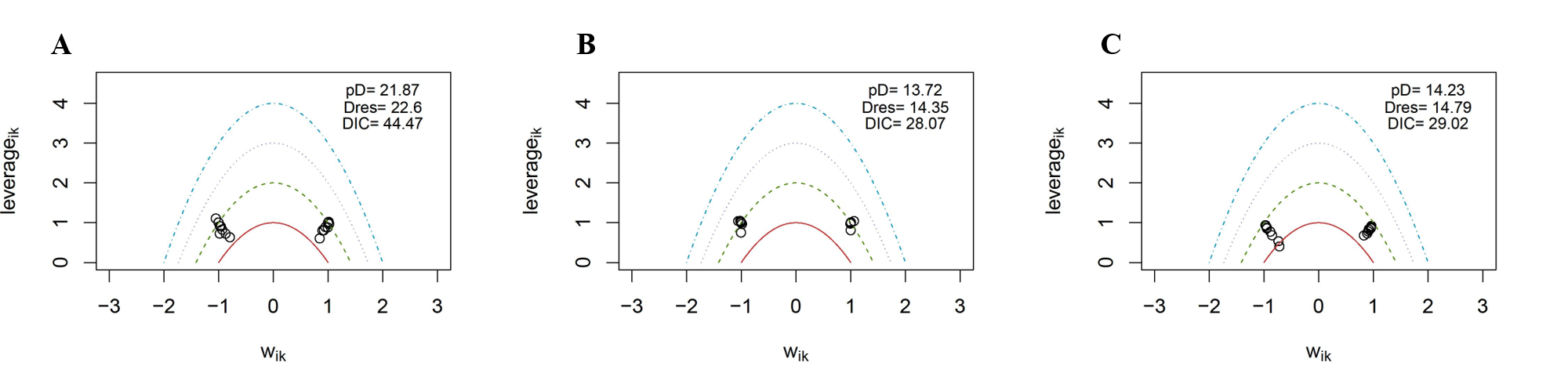


**Supplementary Figure 18.** Leverage plots of assessing model fit of evaluations of (A) induction of clinical remission, (B) induction of CDAI-70, (C) induction of CDAI-100, in tumor necrosis factor antagonist-experienced patients with moderate-to-severe Crohn’s disease.


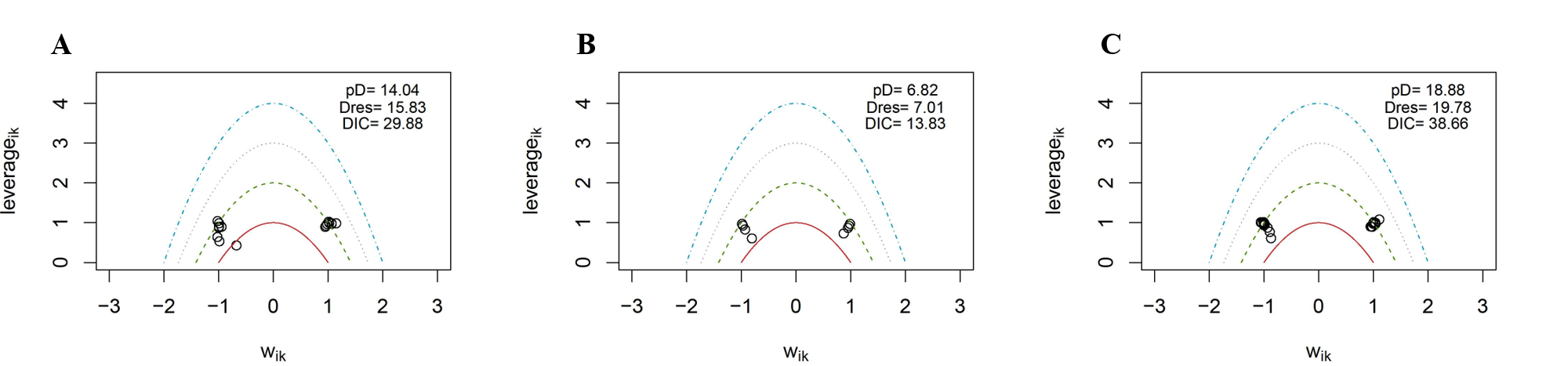


**Supplementary Figure 19.** Leverage plots of assessing model fit of evaluations of maintenance of clinical remission (A) in tumor necrosis factor antagonist-naïve patients, (B) in tumor necrosis factor antagonist-experienced patients, with moderate-to-severe Crohn’s disease.


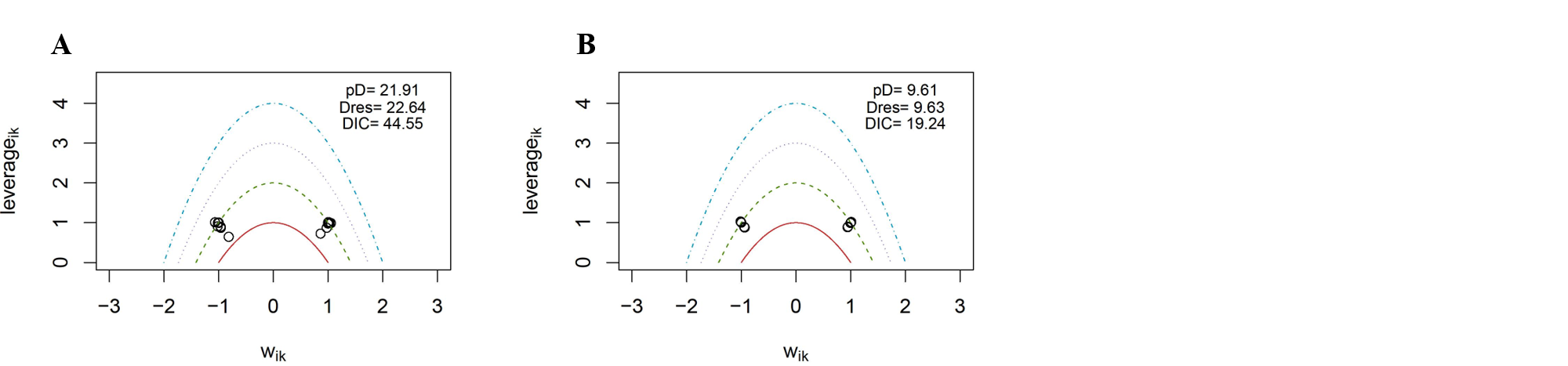


**Supplementary Figure 20.** Brooks-Gelman-Rubin diagnosis plot for Markov chain Monte Carlo (MCMC) chains of evaluations of (A) inducing clinical remission, (B) inducing CDAI-70, (C) inducing CDAI-100, and (D) risk of adverse events in induction therapy, (E) risk of serious adverse events in induction therapy, (F) risk of serious infections in induction therapy, between included biologic agents in overall patients with moderate-to-severe Crohn’s disease.

A.


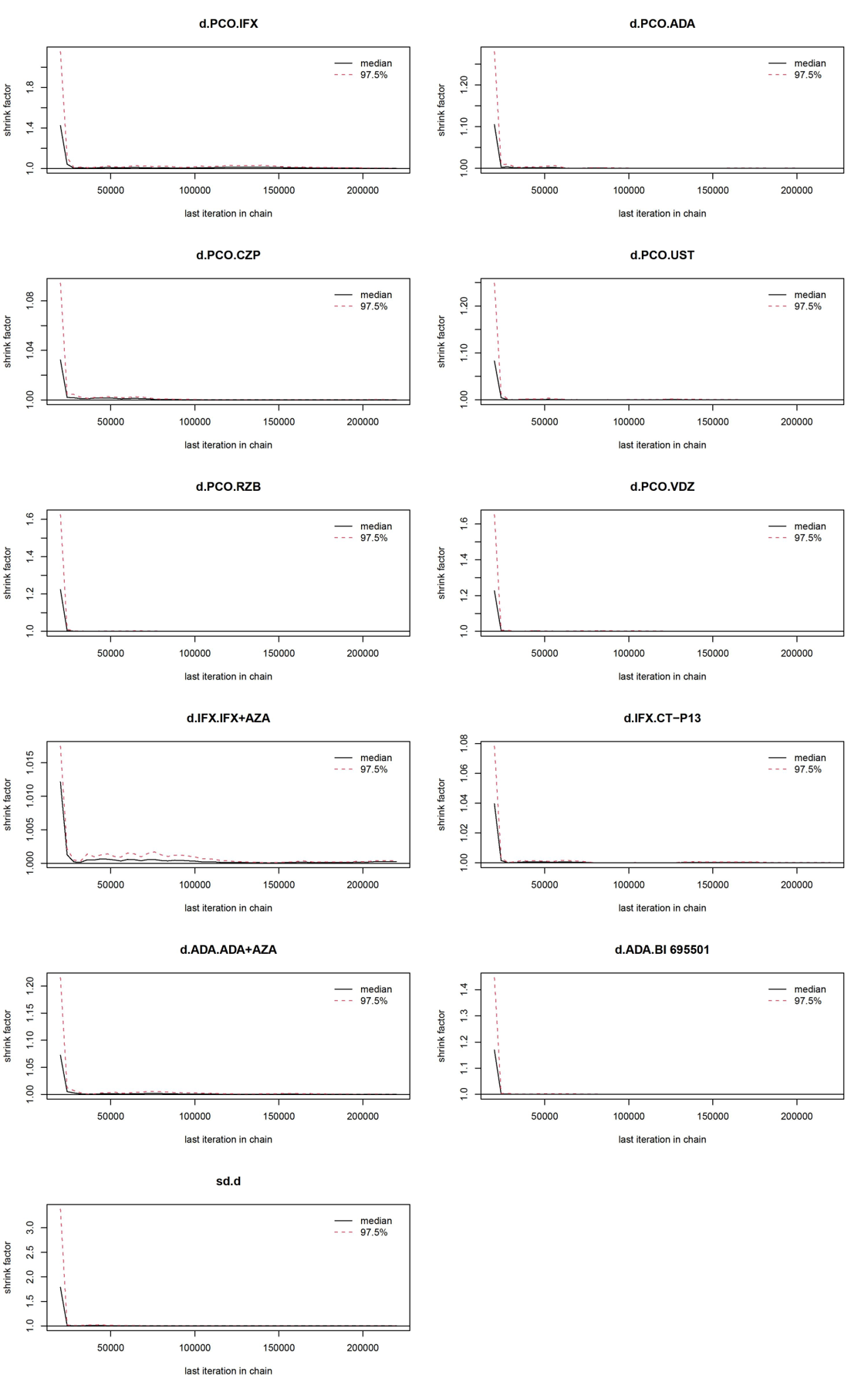


B.


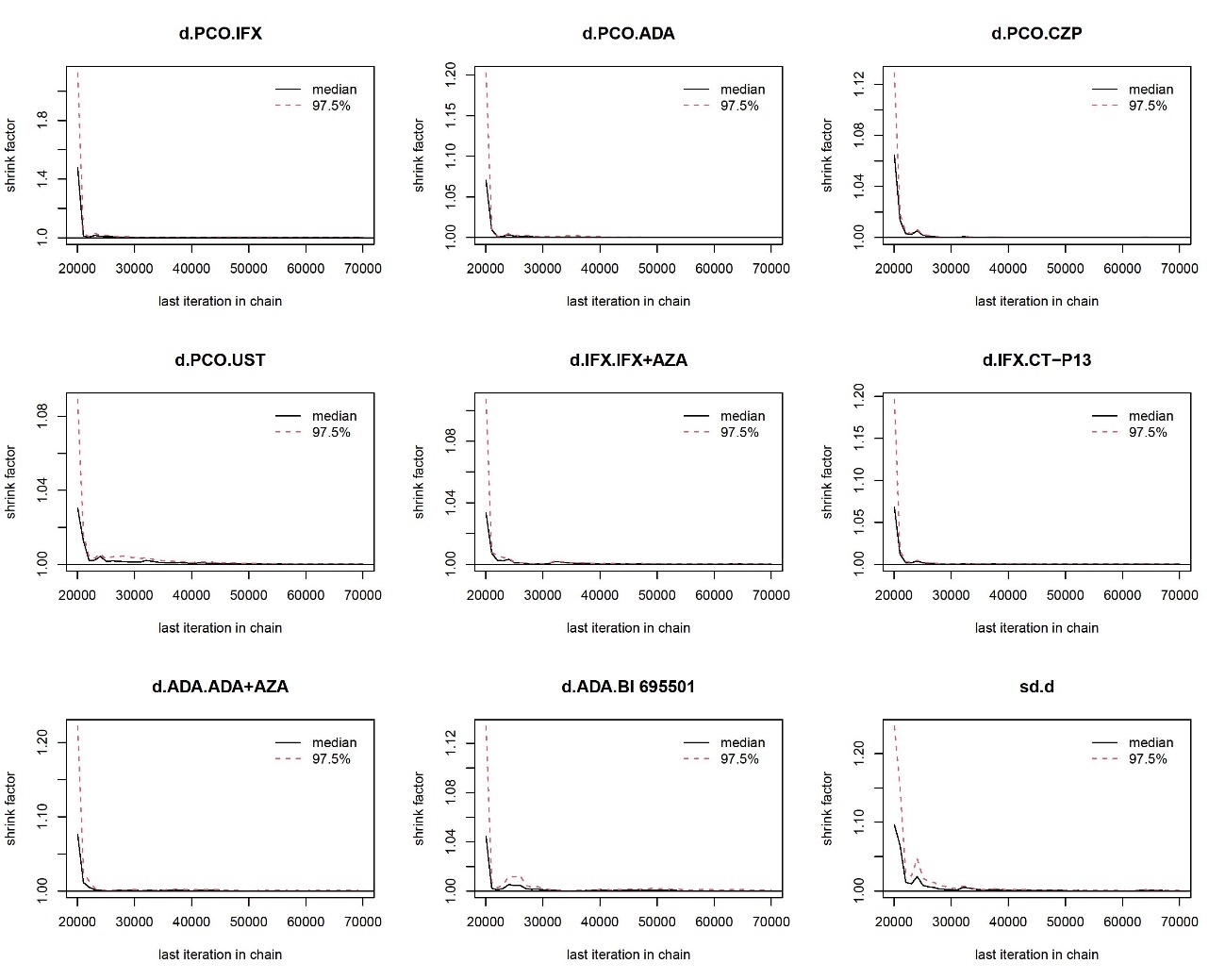


C.


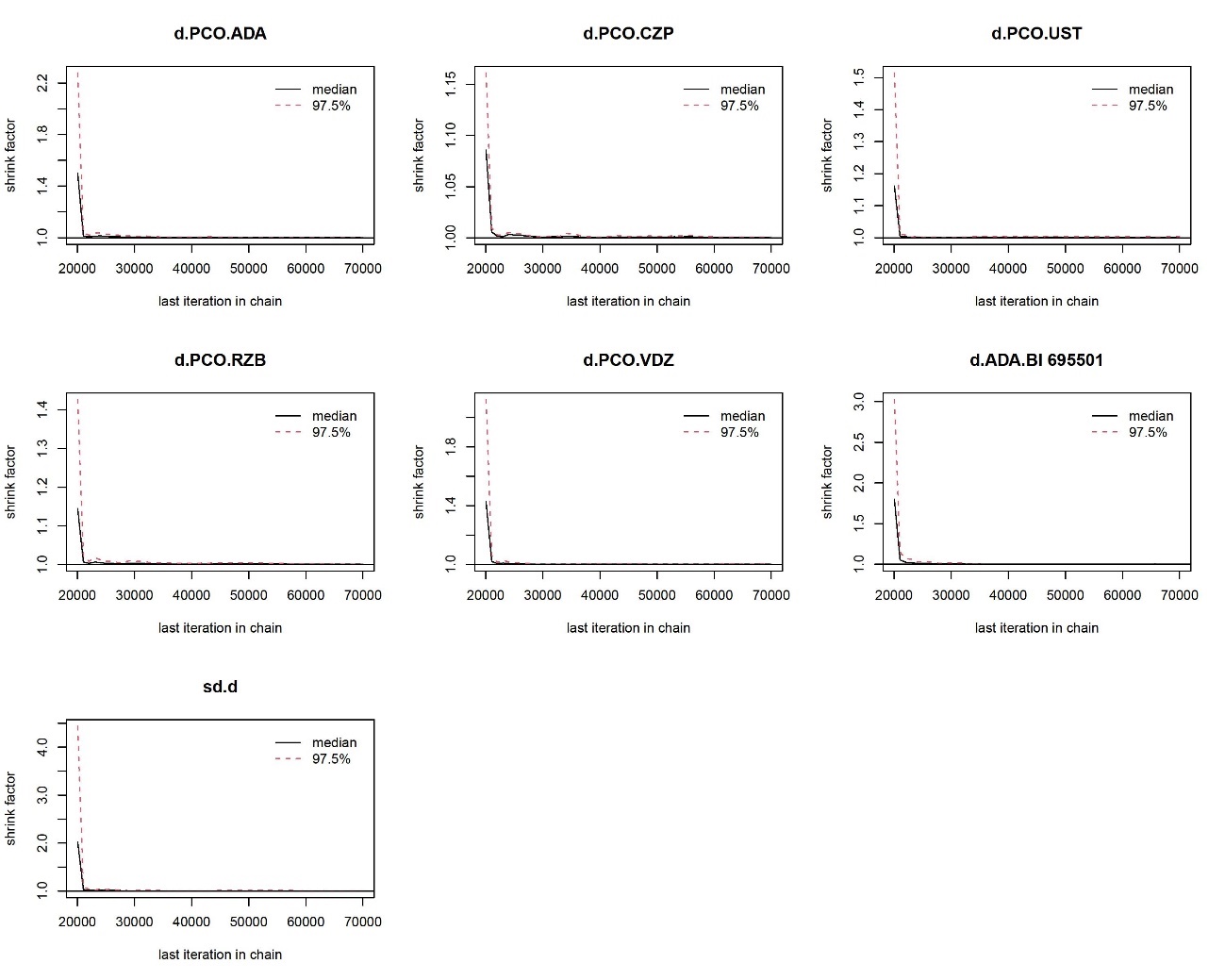


D.


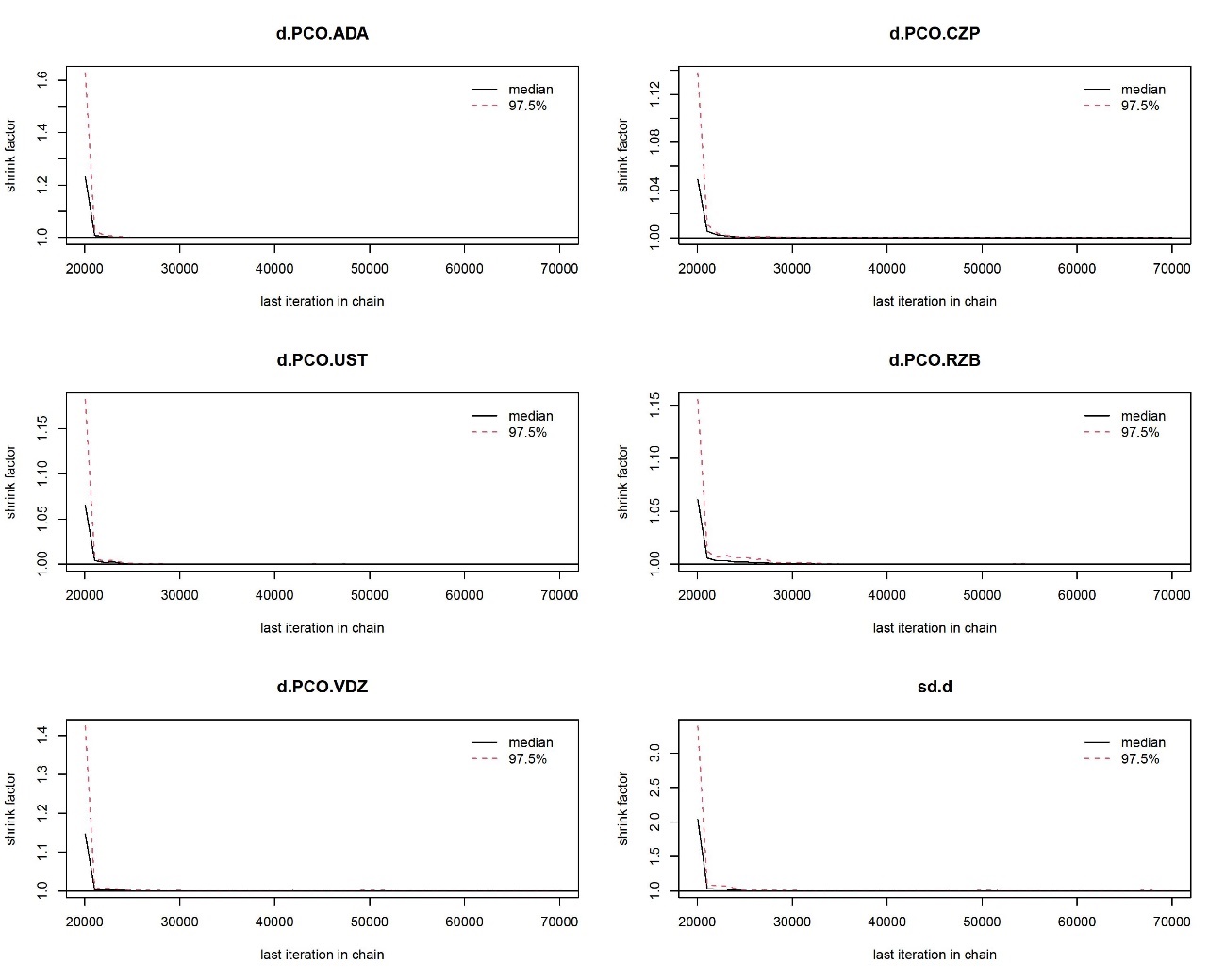


E.


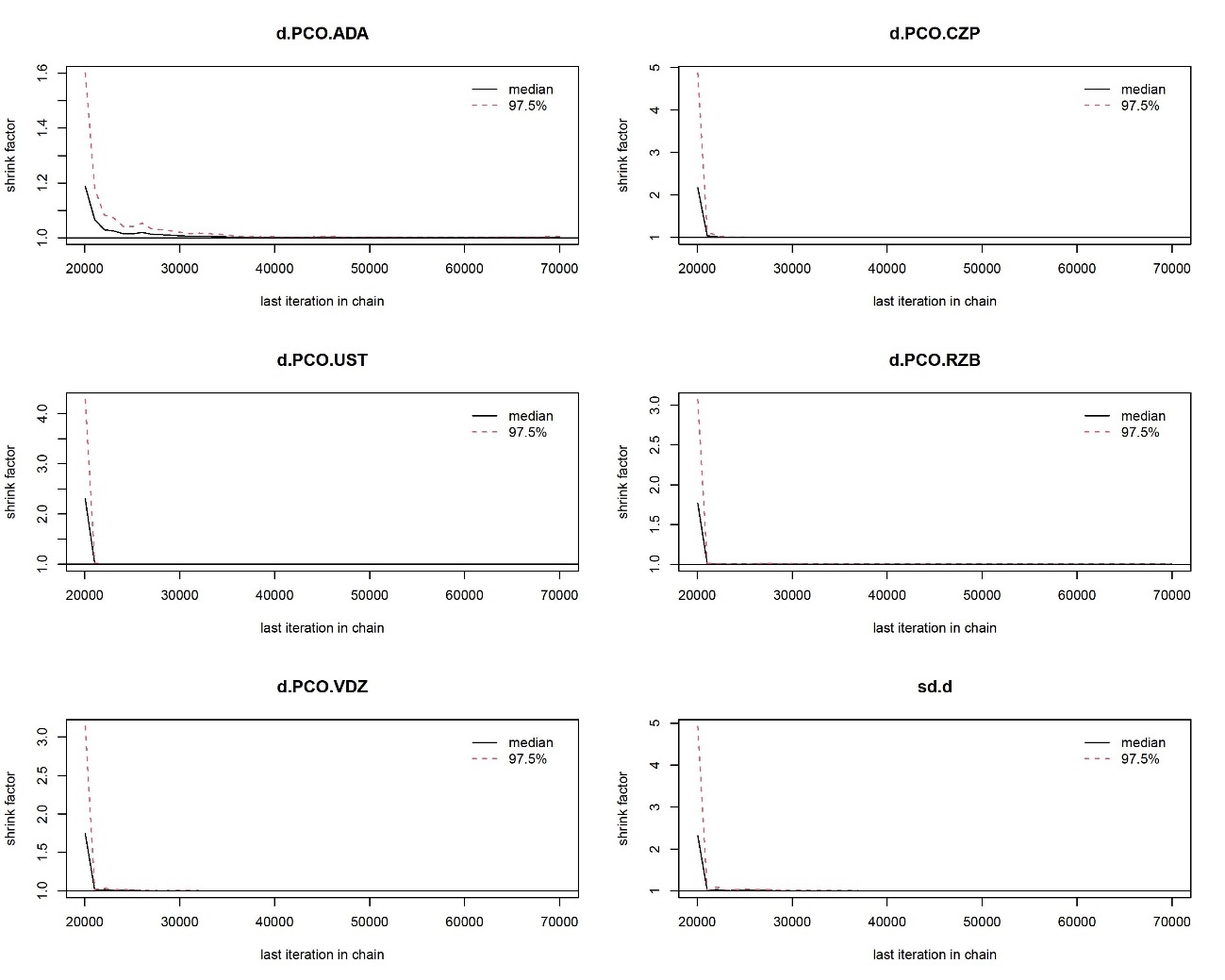


F.


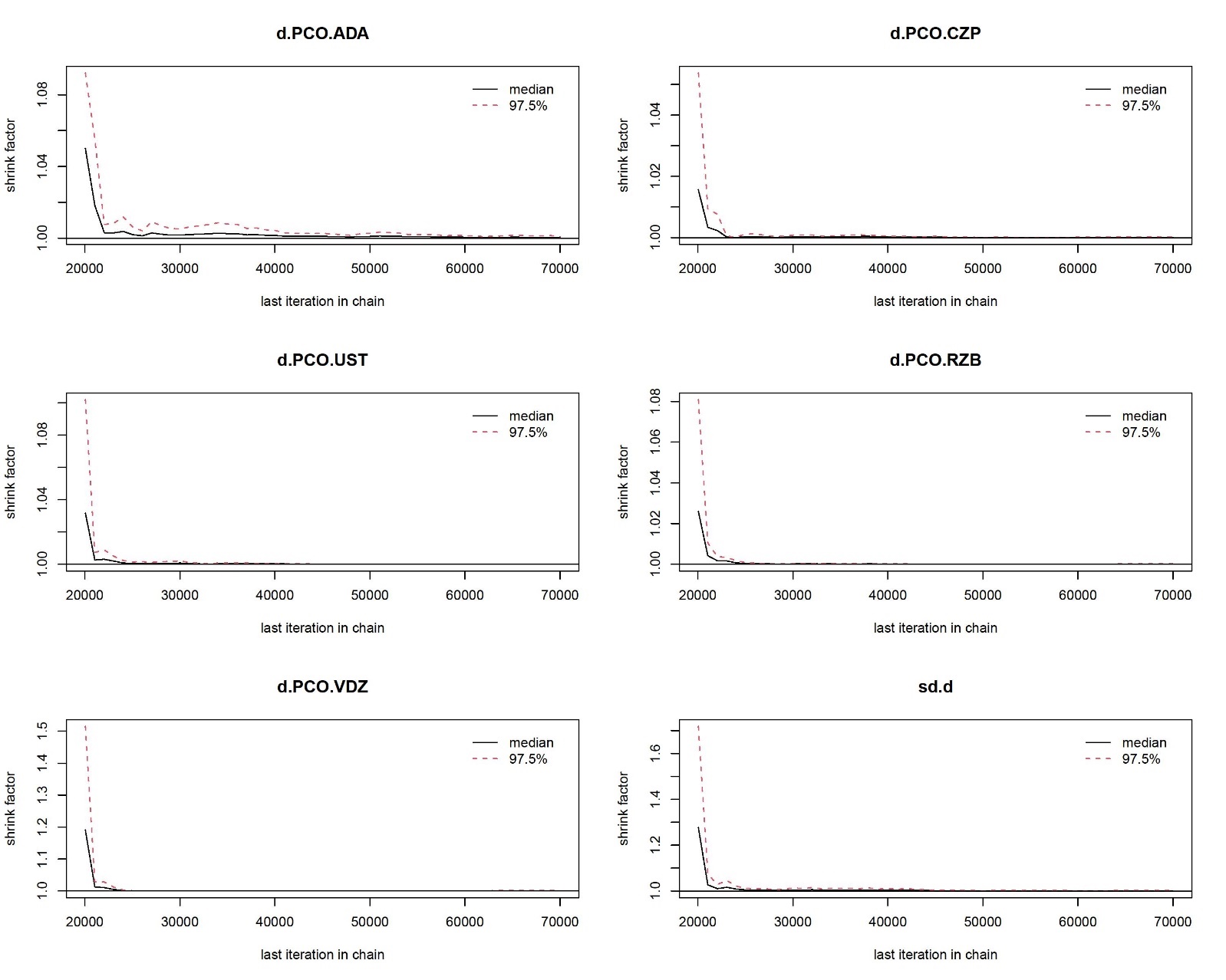


**Supplementary Figure 21.** Brooks-Gelman-Rubin diagnosis plot for Markov chain Monte Carlo (MCMC) chains of evaluations of (A) maintaining clinical remission, (B) maintaining CDAI-70, (C) maintaining CDAI-100, and (D) risk of adverse events in maintenance therapy, (E) risk of serious adverse events in maintenance therapy, (F) risk of serious infections in maintenance therapy, between included biologic agents in overall patients with moderate-to-severe Crohn’s disease.

A.


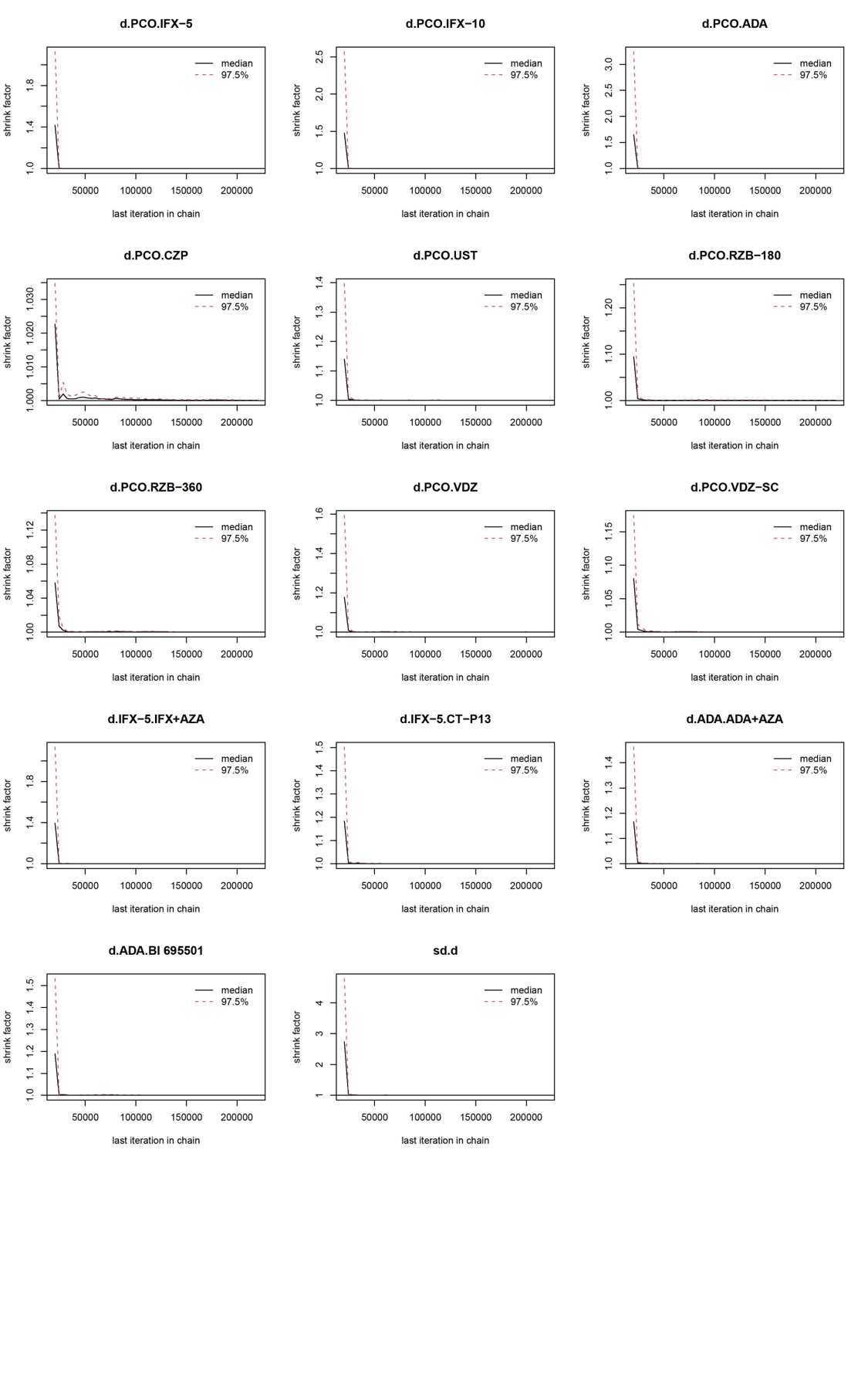


B.


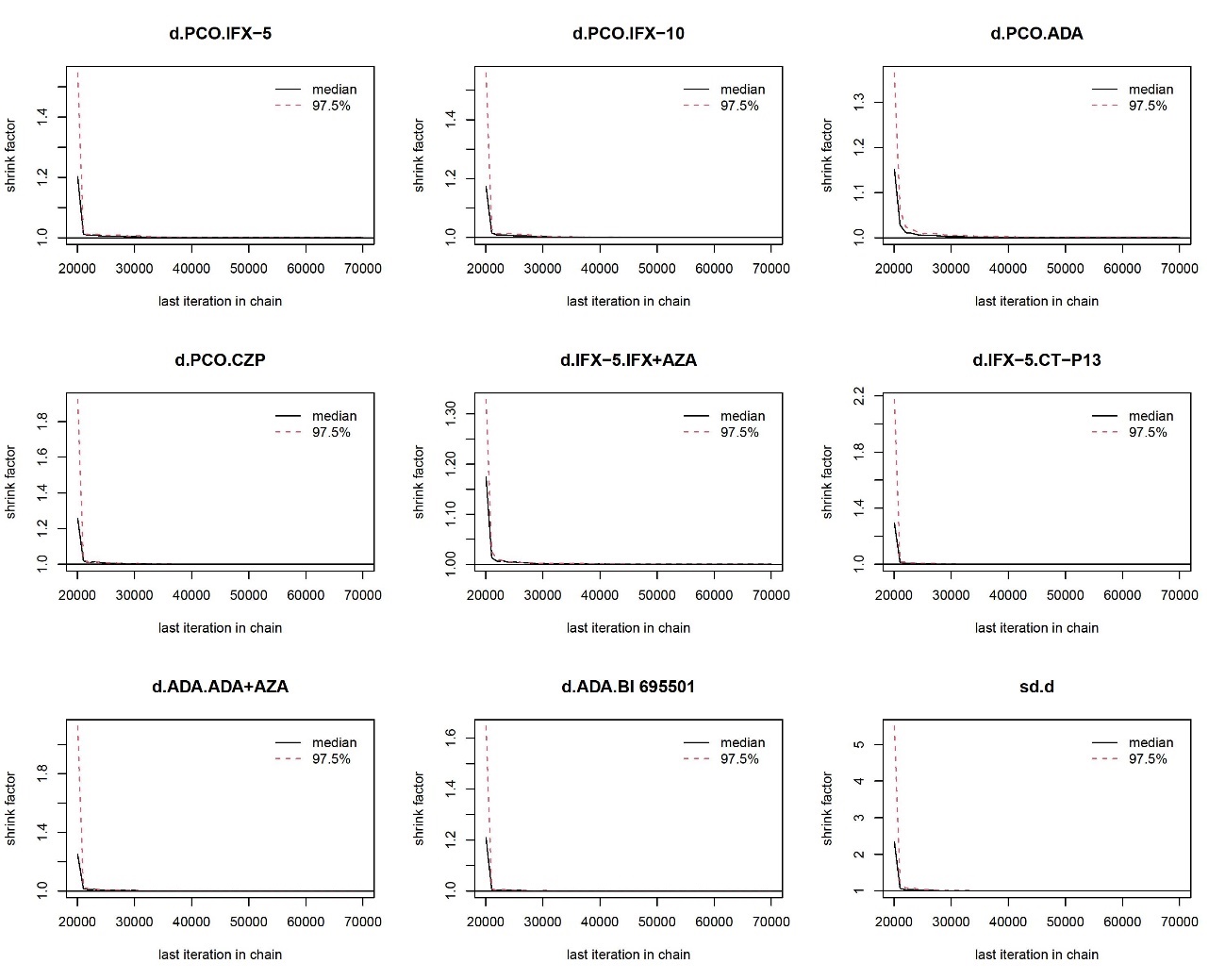


C.


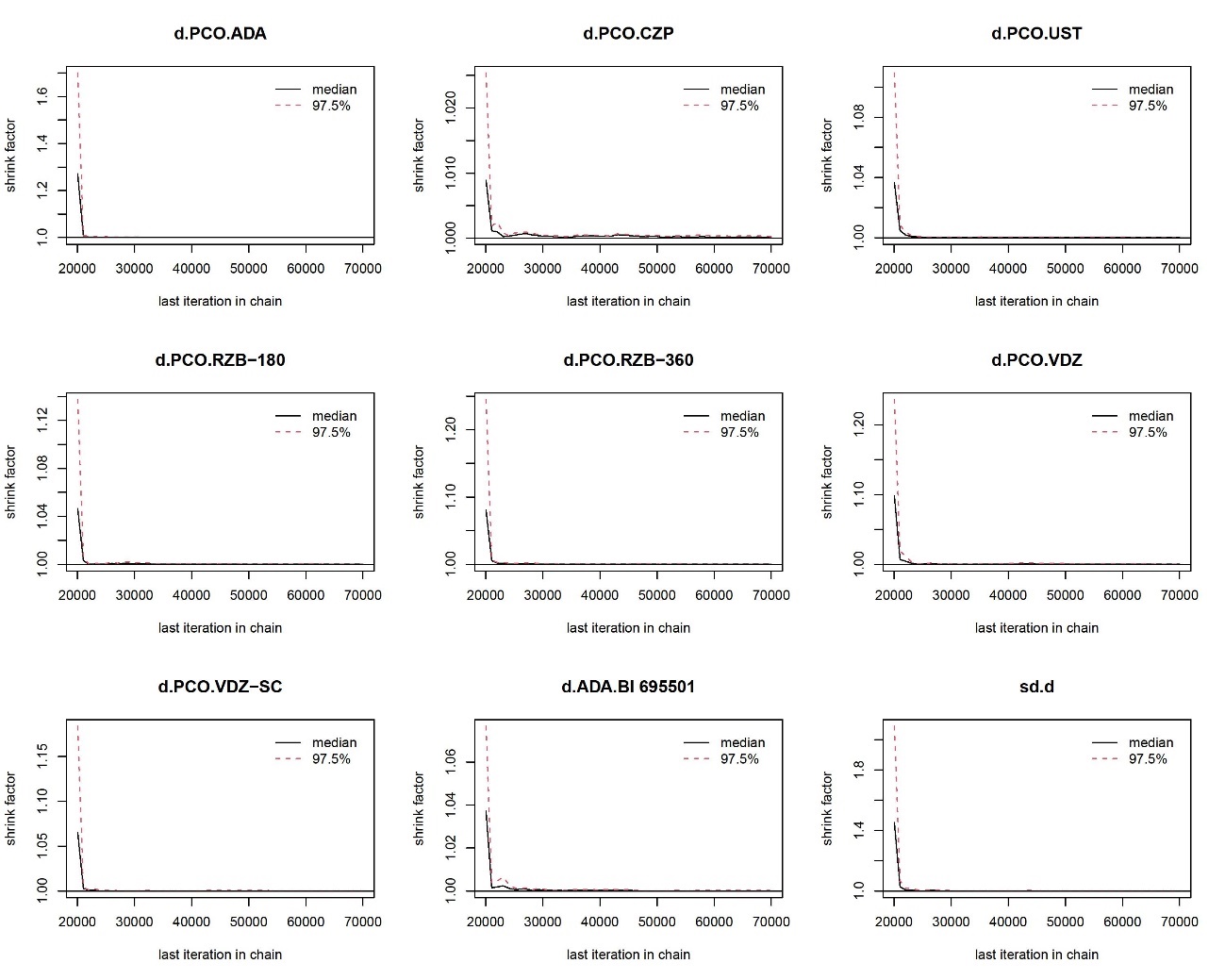


D.


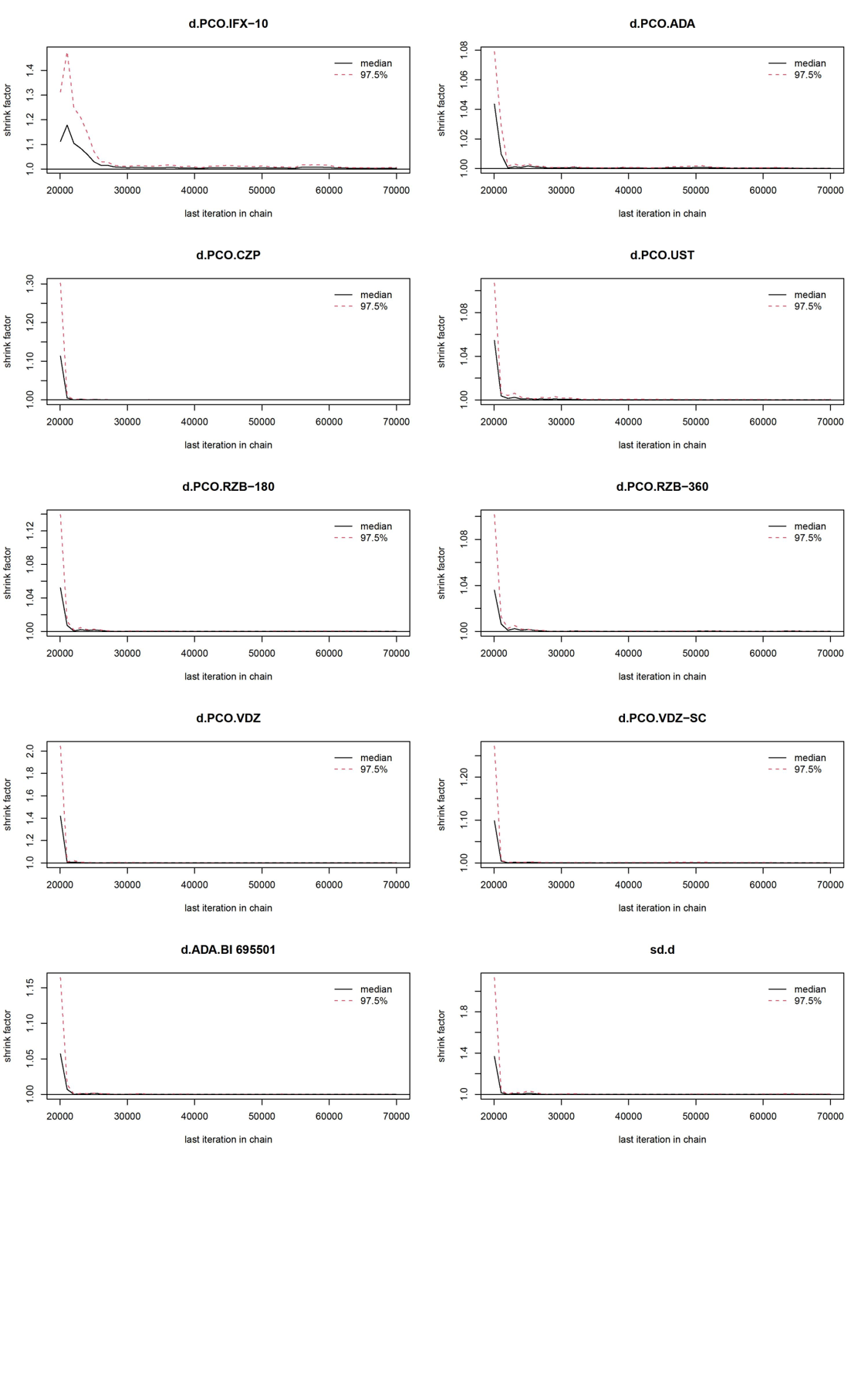


E.


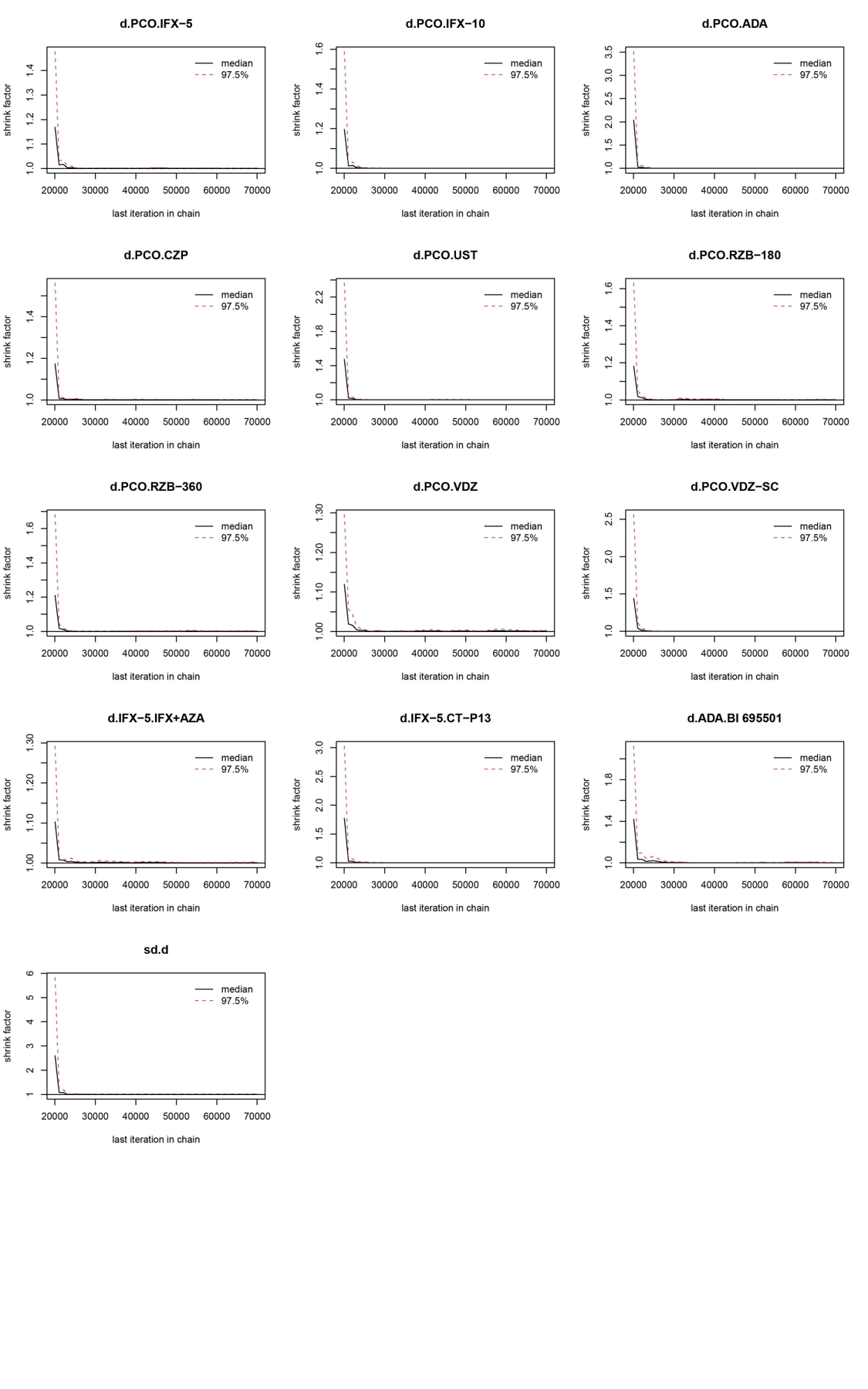


F.


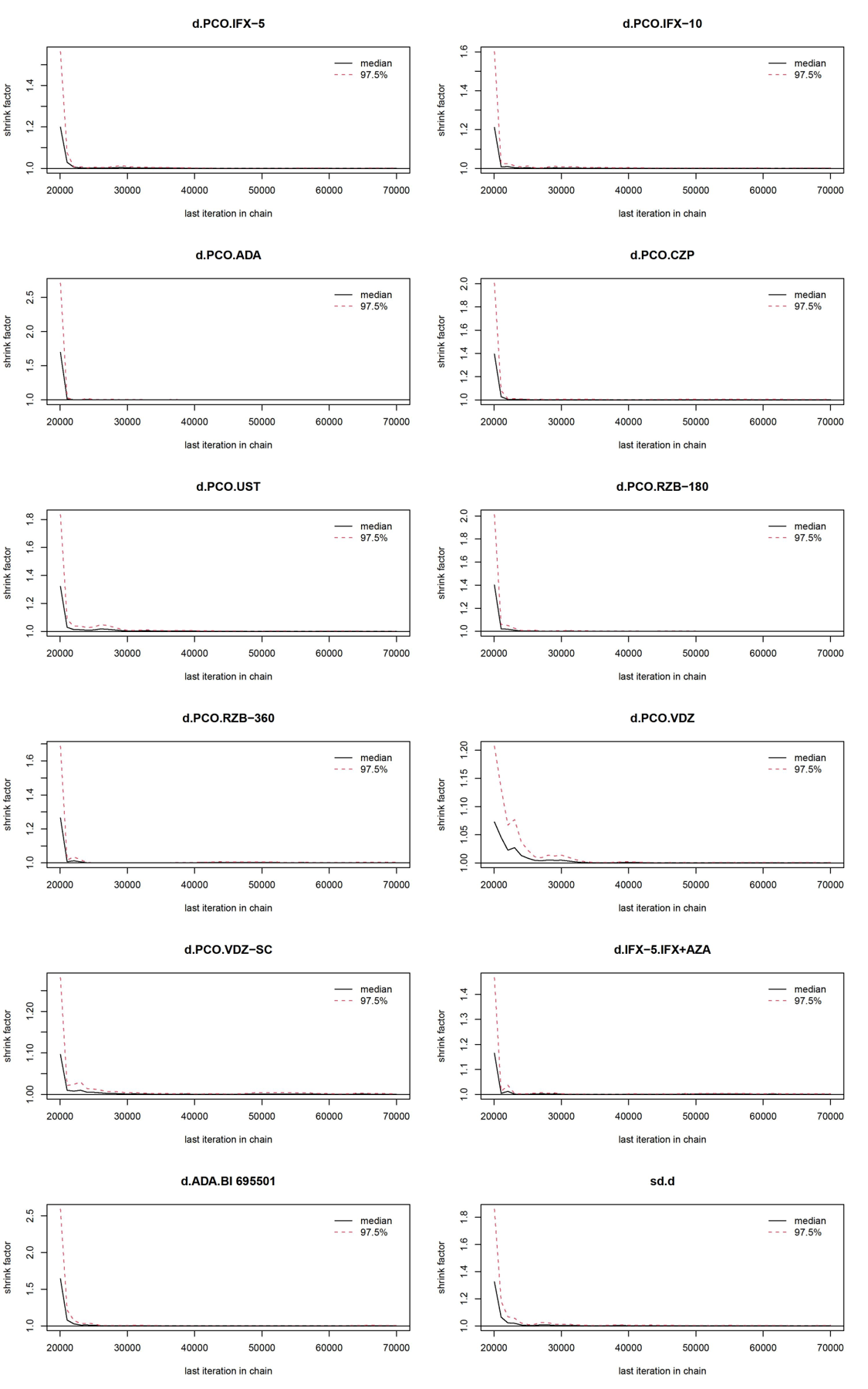


**Supplementary Figure 22.** Brooks-Gelman-Rubin diagnosis plot for Markov chain Monte Carlo (MCMC) chains of evaluations of (A) inducing clinical remission, (B) inducing CDAI-70, (C) inducing CDAI-100, between included biologic agents in tumor necrosis factor antagonist-naïve patients with moderate-to-severe Crohn’s disease.

A.


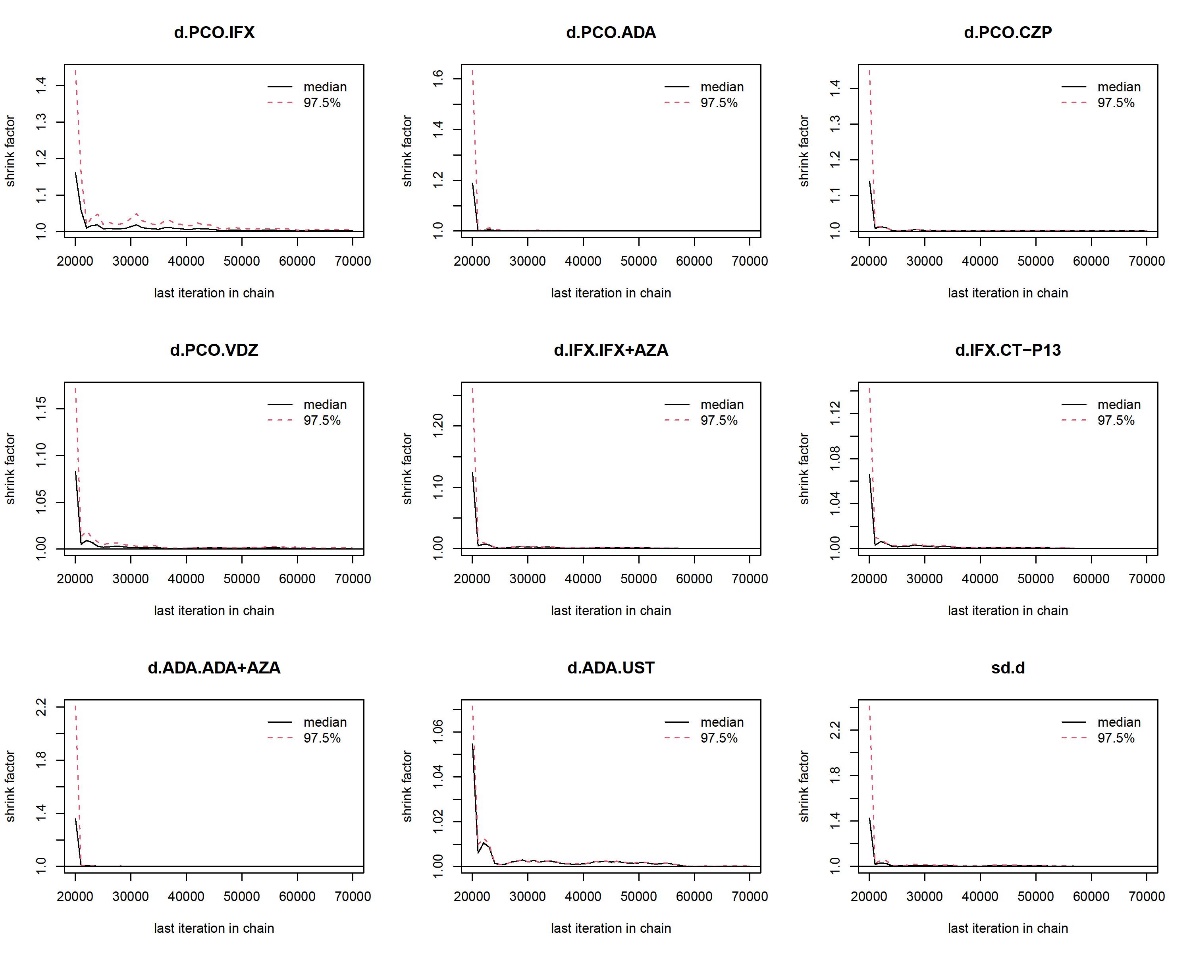


B.


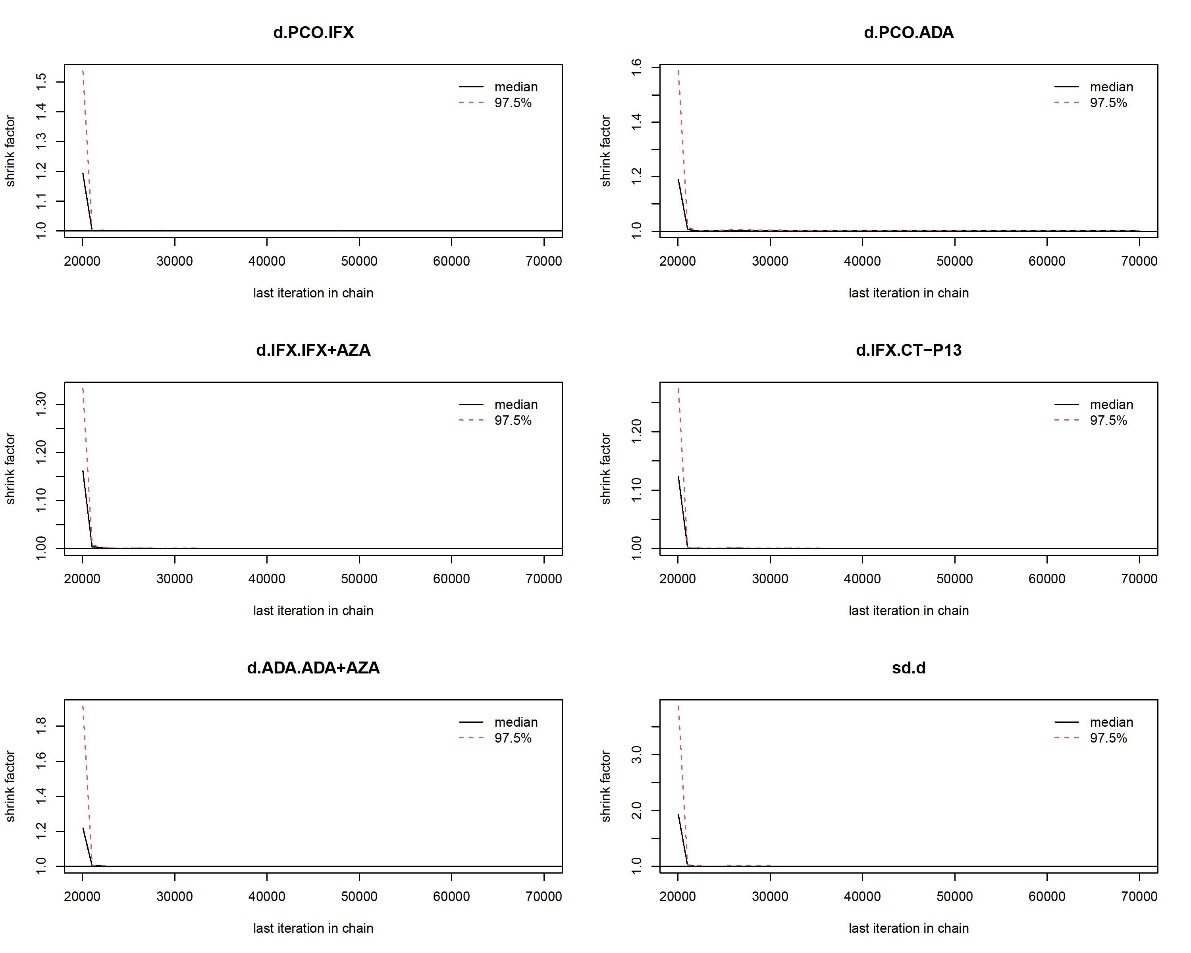


C.


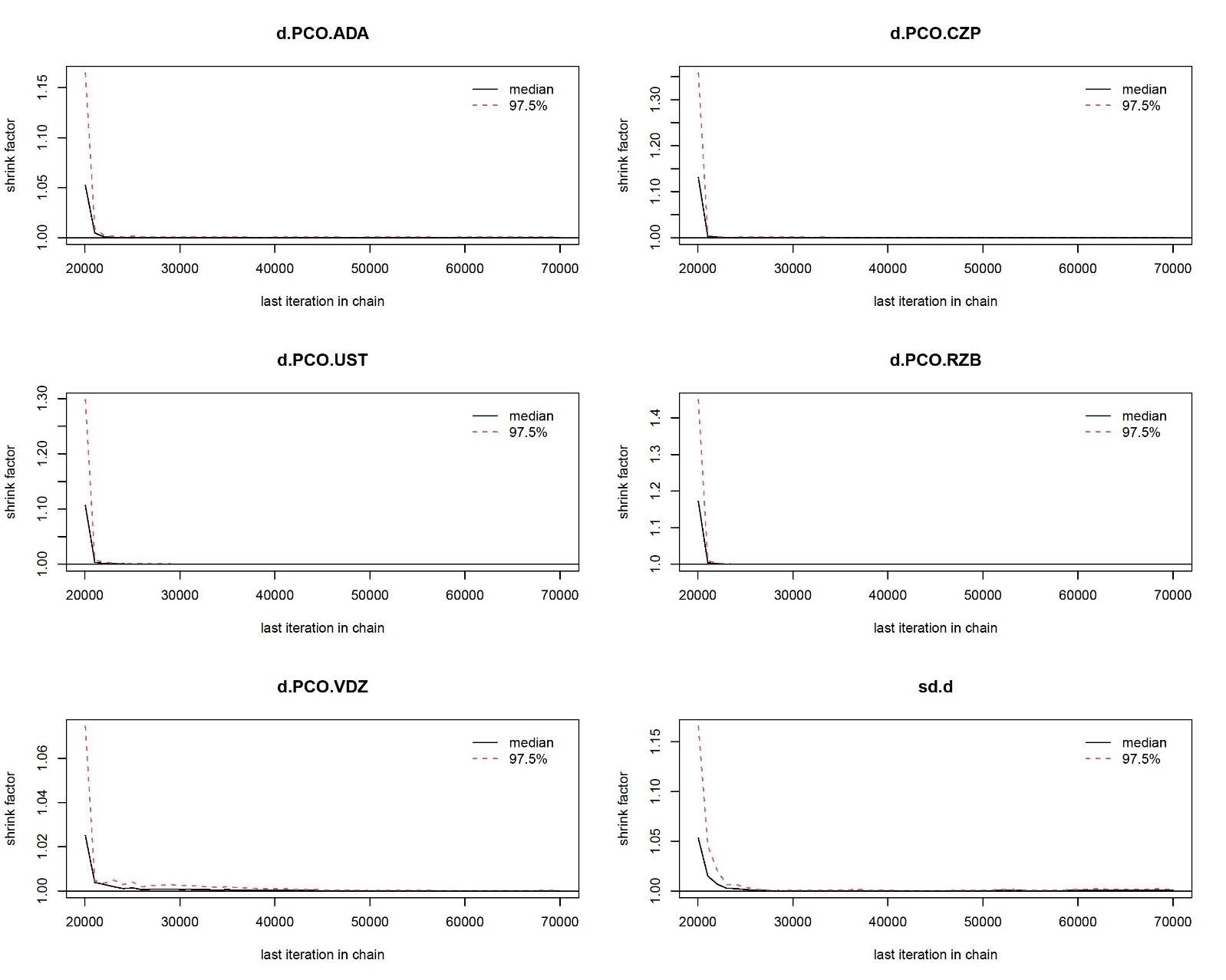


**Supplementary Figure 23.** Brooks-Gelman-Rubin diagnosis plot for Markov chain Monte Carlo (MCMC) chains of evaluations of (A) inducing clinical remission, (B) inducing CDAI-70, (C) inducing CDAI-100, between included biologic agents in tumor necrosis factor antagonist-experienced patients with moderate-to-severe Crohn’s disease.

A.


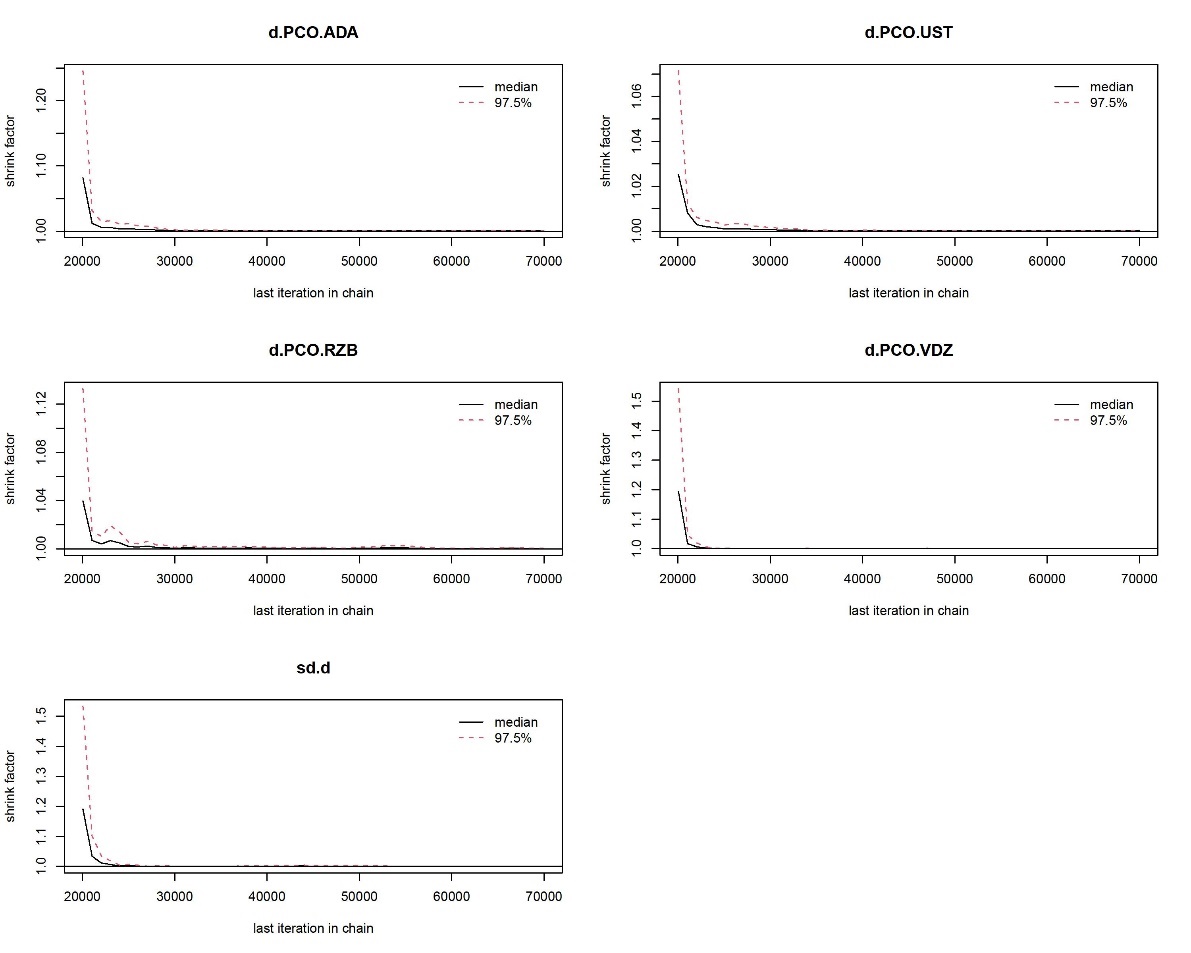


B.


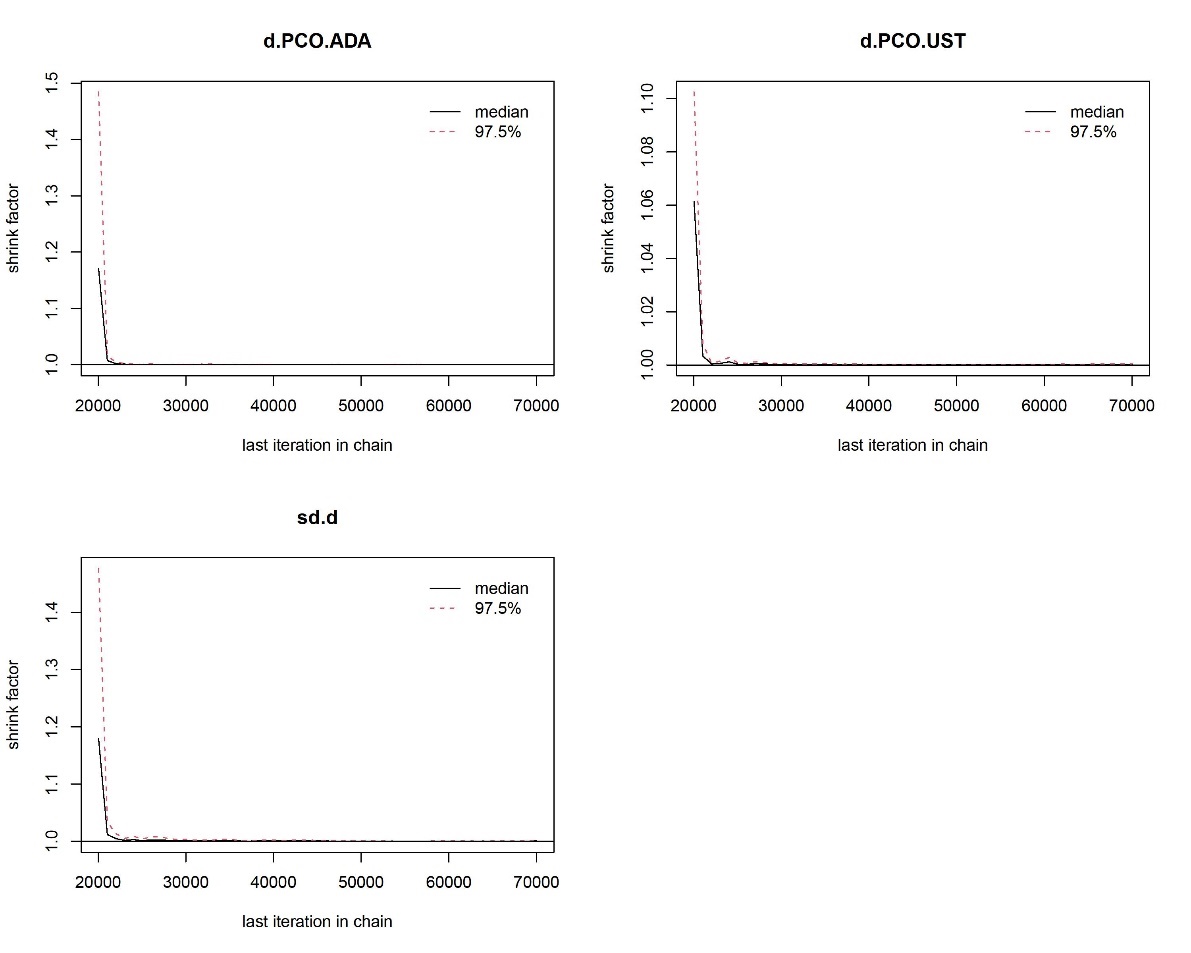


C.


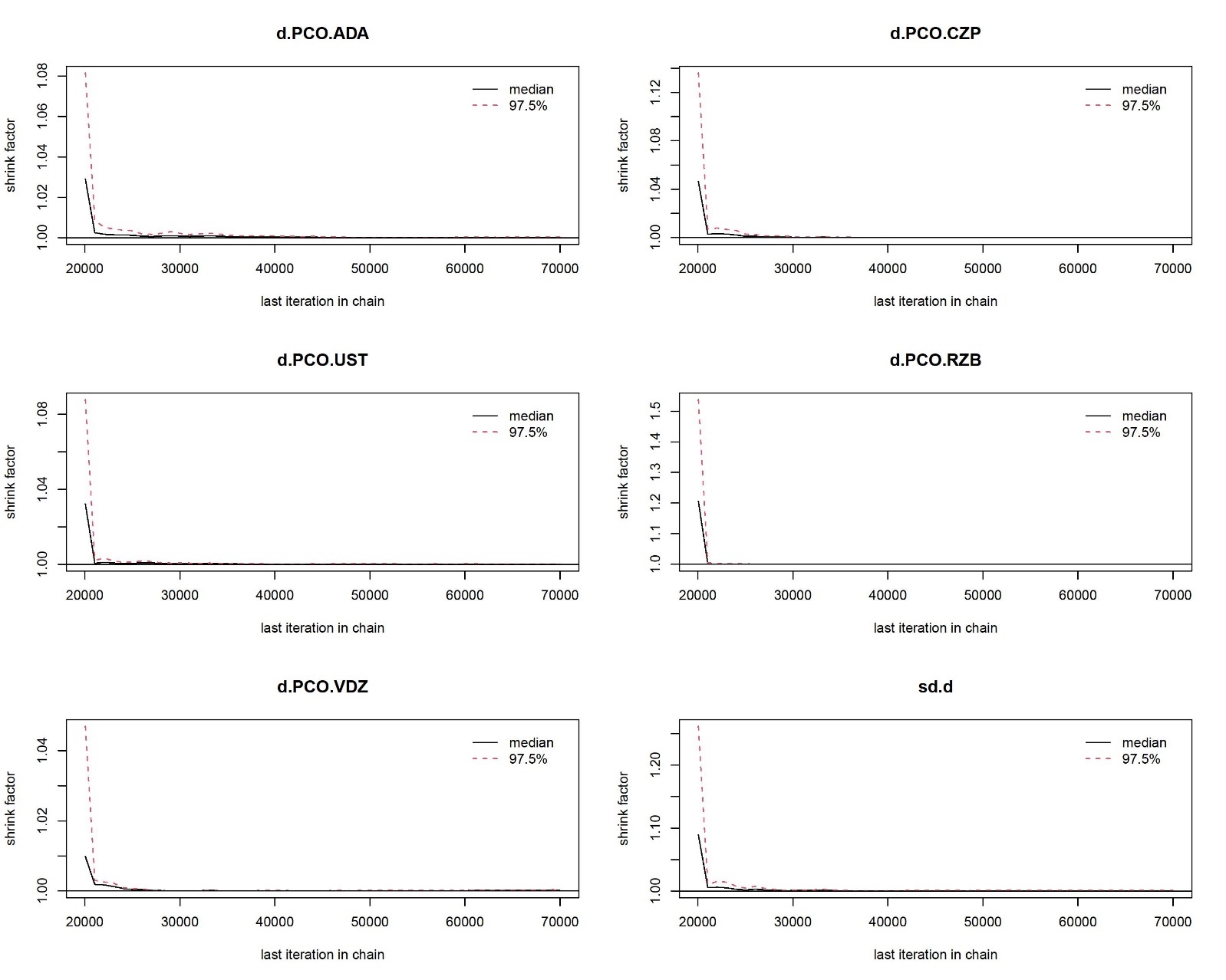


**Supplementary Figure 24.** Brooks-Gelman-Rubin diagnosis plot for Markov chain Monte Carlo (MCMC) chains of evaluations of maintaining clinical remission between included biologic agents (A) in tumor necrosis factor antagonist-naïve patients, (B) in tumor necrosis factor antagonist-experienced patients, with moderate-to-severe Crohn’s disease.

A.


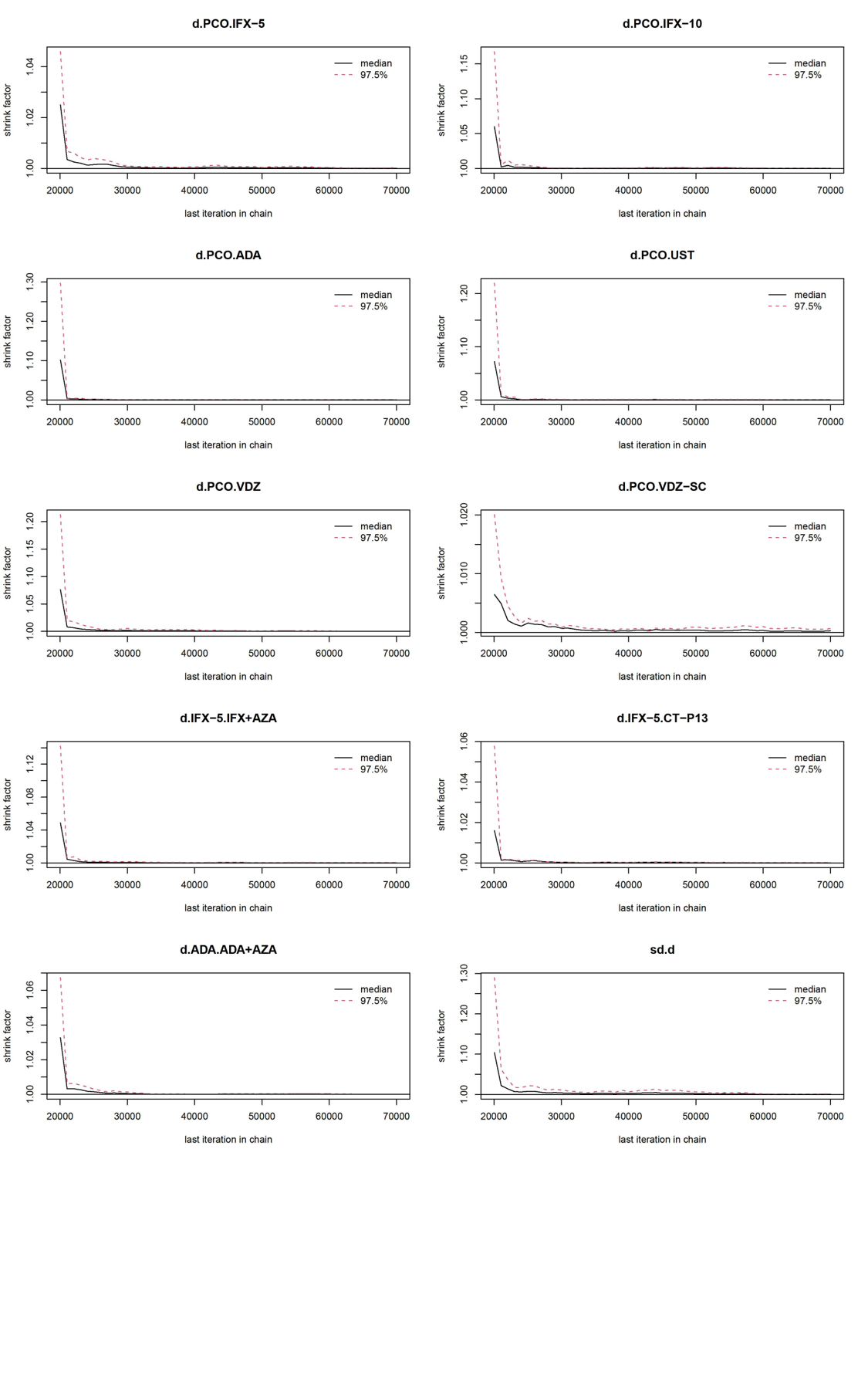


B.


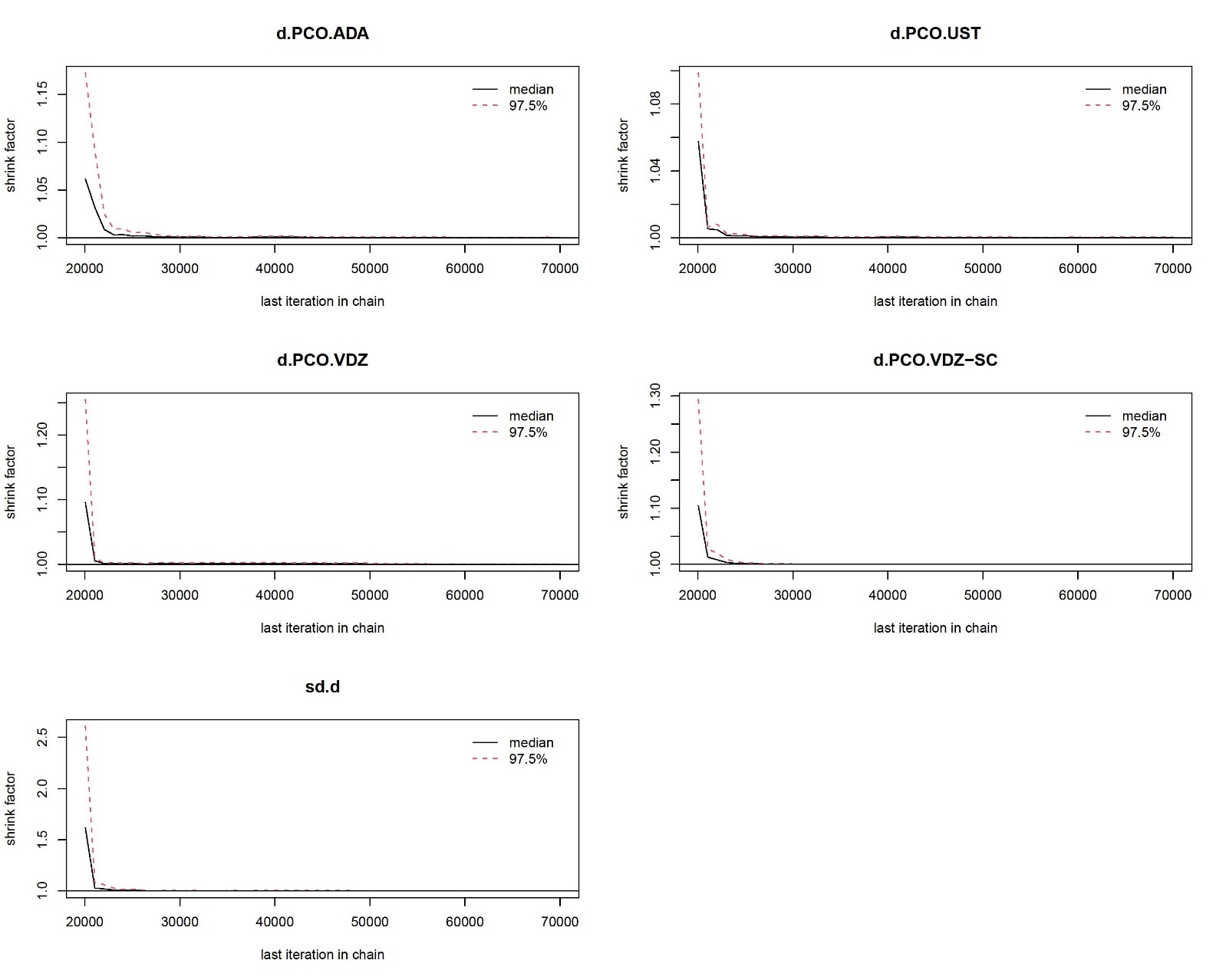


**Supplementary Figure 25.** League heat plots of mixed comparisons of inducing clinical remission between included biologic agents in overall patients with moderate-to-severe Crohn’s disease, reporting odds ratios on the logarithmic scale (log OR) with 95% confidence intervals. Log OR greater or less than 0 are indicated in red or blue, respectively, in comparisons of inducing clinical remission, and the darker the color, the greater the odds ratios. Bold type represents statistically significant superiority/inferiority of the intervention over the comparator.


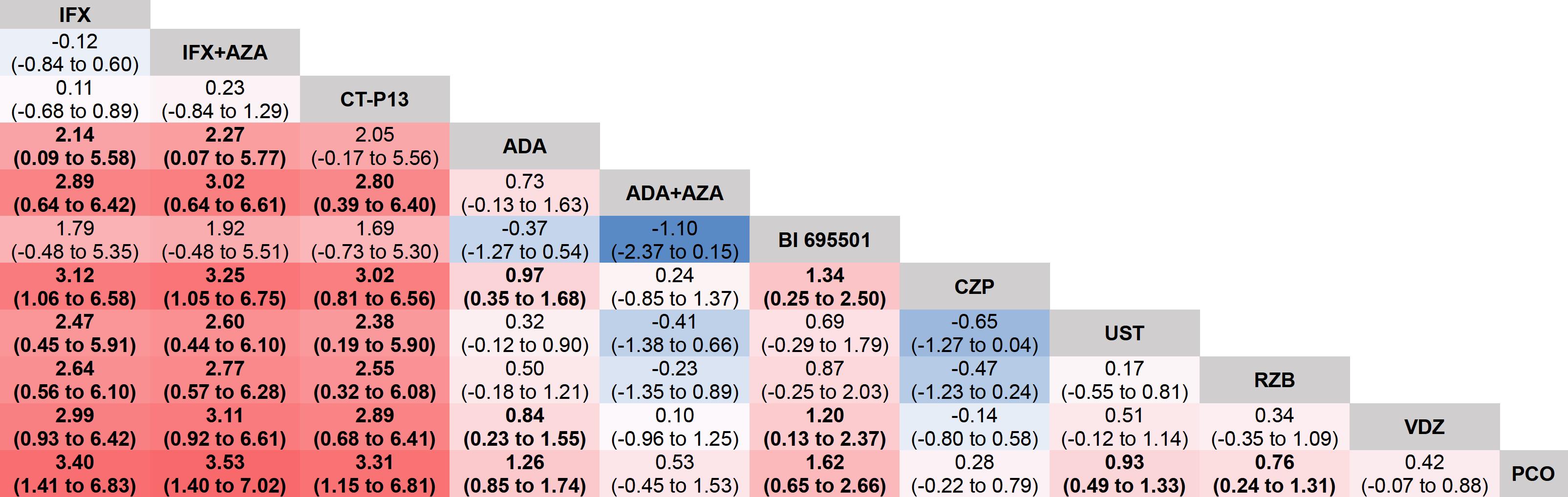


**Supplementary Figure 26.** League heat plot of mixed comparisons of risk of adverse events and risk of serious adverse events in induction therapy between included biologic agents in overall patients with moderate-to-severe Crohn’s disease, reporting odds ratios on the logarithmic scale (log OR) with 95% confidence intervals. Log OR greater or less than 0 are indicated in red (yellow) or blue (green), respectively, in comparisons of risk of adverse events (risk of serious adverse events) in induction therapy, and the darker the color, the greater the odds ratios. Bold type represents statistically significant superiority/inferiority of the intervention over the comparator.


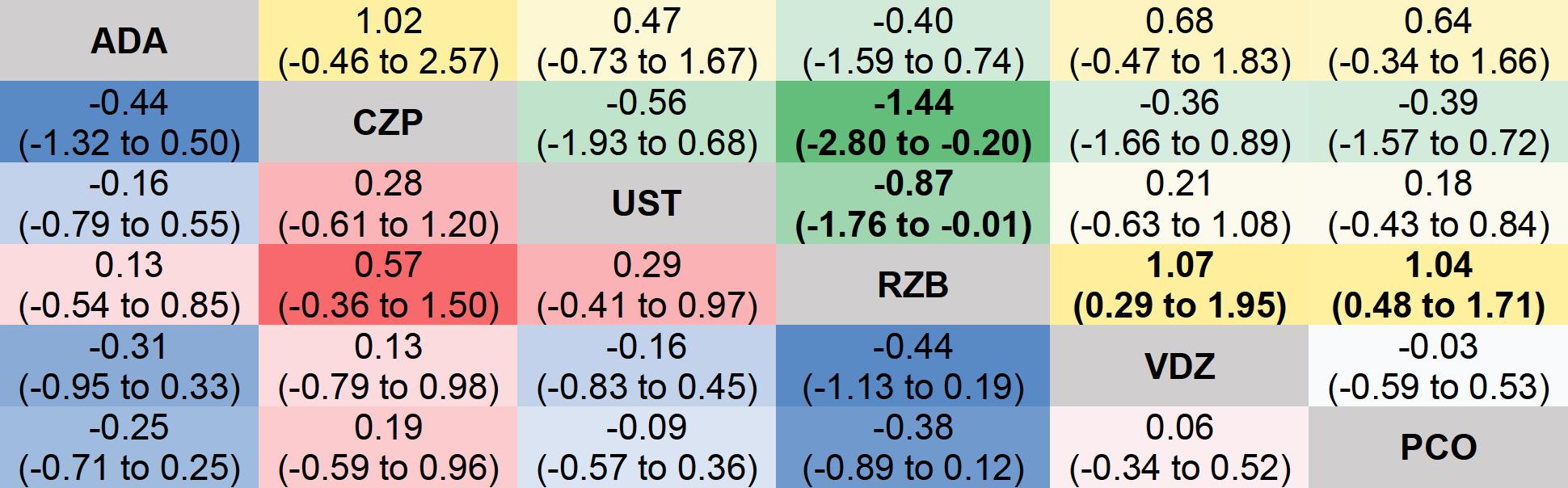


**Supplementary Figure 27.** League heat plot of mixed comparisons of risk of serious infections in induction therapy between included biologic agents in overall patients with moderate-to-severe Crohn’s disease, reporting odds ratios on the logarithmic scale (log OR) with 95% confidence intervals. Log OR greater or less than 0 are indicated in red or blue, respectively, in comparisons of risk of serious infections in induction therapy, and the darker the color, the greater the odds ratios. Bold type represents statistically significant superiority/inferiority of the intervention over the comparator.


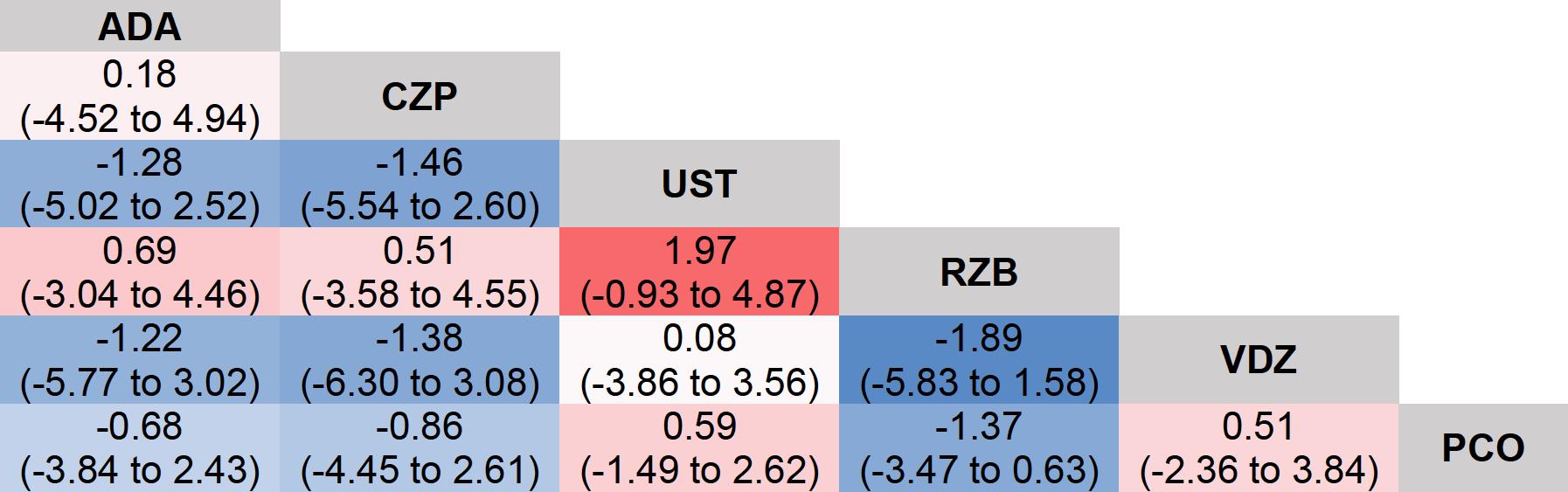


**Supplementary Figure 28.** League heat plots of mixed comparisons of maintaining CDAI-100 and CDAI-70 between included biologic agents in overall patients with moderate-to-severe Crohn’s disease, reporting odds ratios on the logarithmic scale (log OR) with 95% confidence intervals. Log OR greater or less than 0 are indicated in red (yellow) or blue (green), respectively, in comparisons of maintaining CDAI-100 (CDAI-70), and the darker the color, the greater the odds ratios. Bold type represents statistically significant superiority/inferiority of the intervention over the comparator.


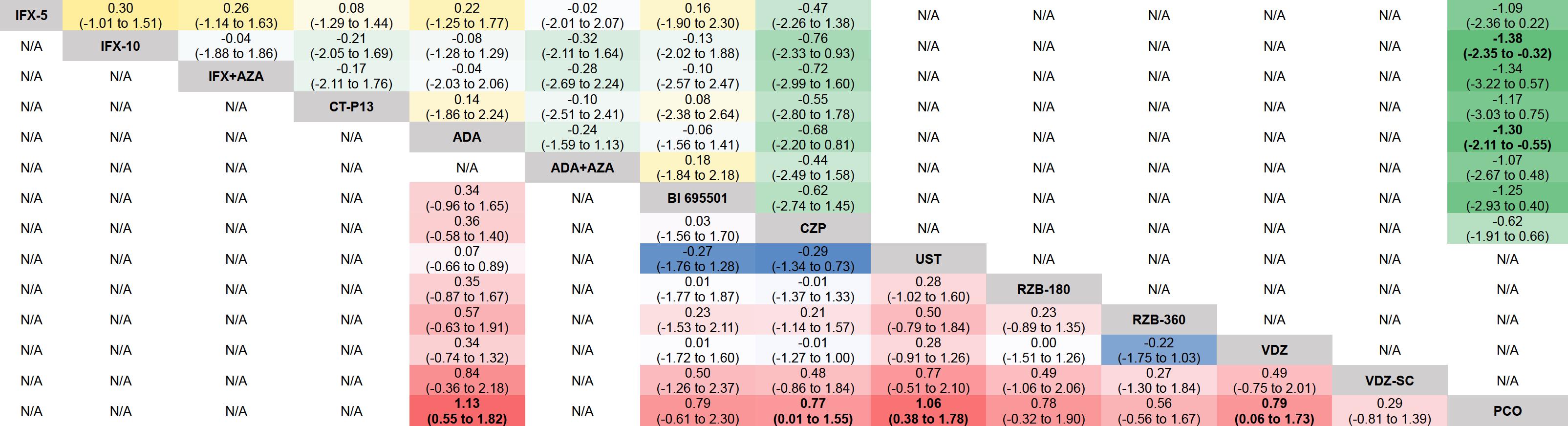


**Supplementary Figure 29.** League heat plot of mixed comparisons of risk of adverse events and risk of serious adverse events in maintenance therapy between included biologic agents in overall moderate-to-severe Crohn’s disease, reporting odds ratios on the logarithmic scale (log OR) with 95% confidence intervals. Log OR greater or less than 0 are indicated in red (yellow) or blue (green), respectively, in comparisons of risk of adverse events (risk of serious adverse events) in maintenance therapy, and the darker the color, the greater the odds ratios. Bold type represents statistically significant superiority/inferiority of the intervention over the comparator.


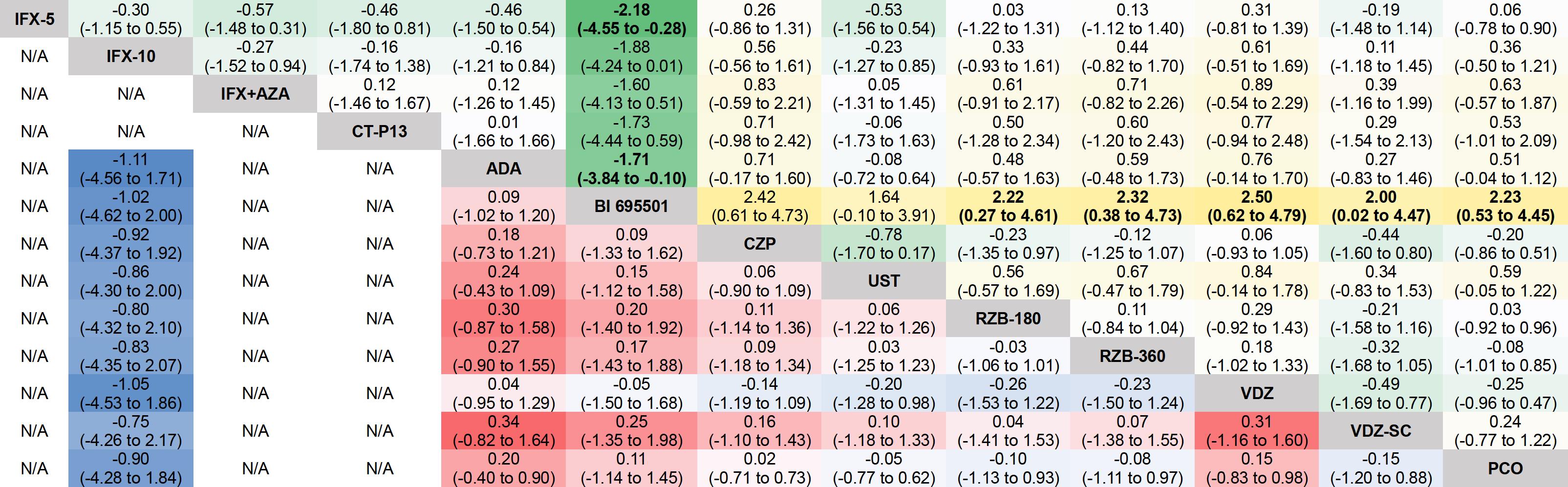


**Supplementary Figure 30.** League heat plot of mixed comparisons of risk of serious infections in maintenance therapy between included biologic agents in overall moderate-to-severe Crohn’s disease, reporting odds ratios on the logarithmic scale (log OR) with 95% confidence intervals. Log OR greater or less than 0 are indicated in red or blue, respectively, in comparisons of risk of serious infections in maintenance therapy, and the darker the color, the greater the odds ratios. Bold type represents statistically significant superiority/inferiority of the intervention over the comparator.


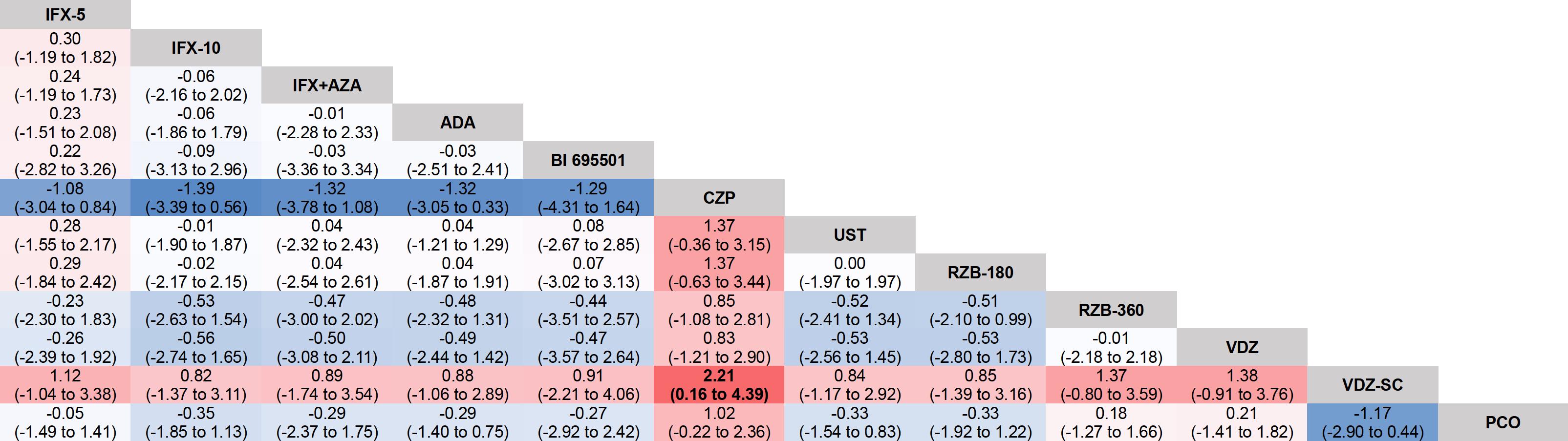


**Supplementary Figure 31.** League heat plots of mixed comparisons of inducing clinical remission between included biologic agents in tumor necrosis factor antagonist-naïve patients with moderate-to-severe Crohn’s disease, reporting odds ratios on the logarithmic scale (log OR) with 95% confidence intervals. Log OR greater or less than 0 are indicated in red or blue, respectively, in comparisons of inducing clinical remission, and the darker the color, the greater the odds ratios. Bold type represents statistically significant superiority/inferiority of the intervention over the comparator.


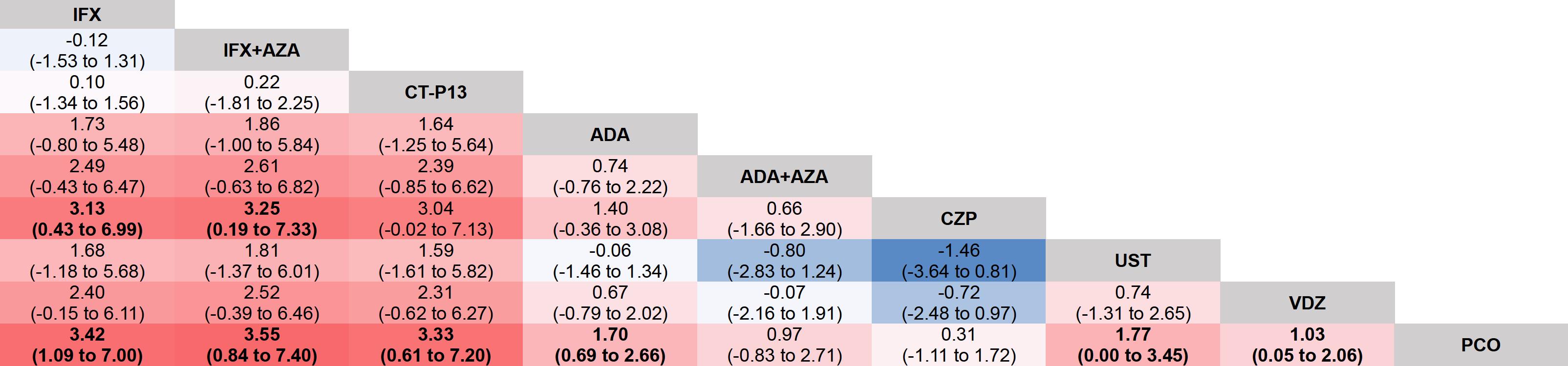


**Supplementary Figure 32.** League heat plots of mixed comparisons of inducing clinical remission between included biologic agents in tumor necrosis factor antagonist-experienced patients with moderate-to-severe Crohn’s disease, reporting odds ratios on the logarithmic scale (log OR) with 95% confidence intervals. Log OR greater or less than 0 are indicated in red or blue, respectively, in comparisons of inducing clinical remission, and the darker the color, the greater the odds ratios. Bold type represents statistically significant superiority/inferiority of the intervention over the comparator.


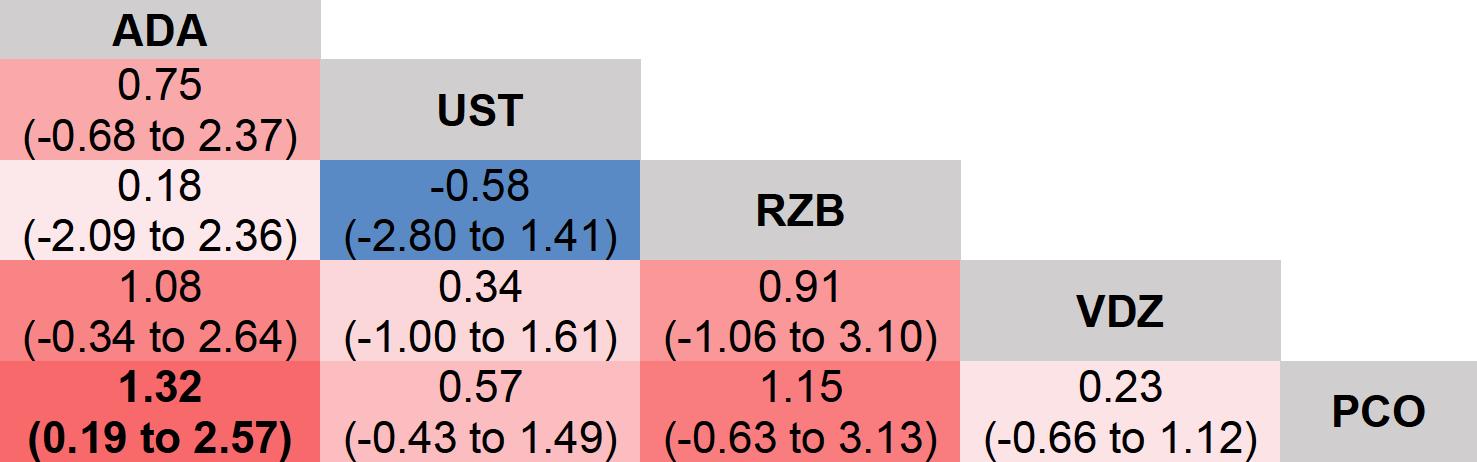


**Supplementary Figure 33.** Sets of plots (including SUCRA plot, rankogram, and forest plot from top to bottom) of mixed comparisons of (A) inducing clinical remission, (B) inducing CDAI-70, (C) inducing CDAI-100, between included biologic agents in overall patients with moderate-to-severe Crohn’s disease.


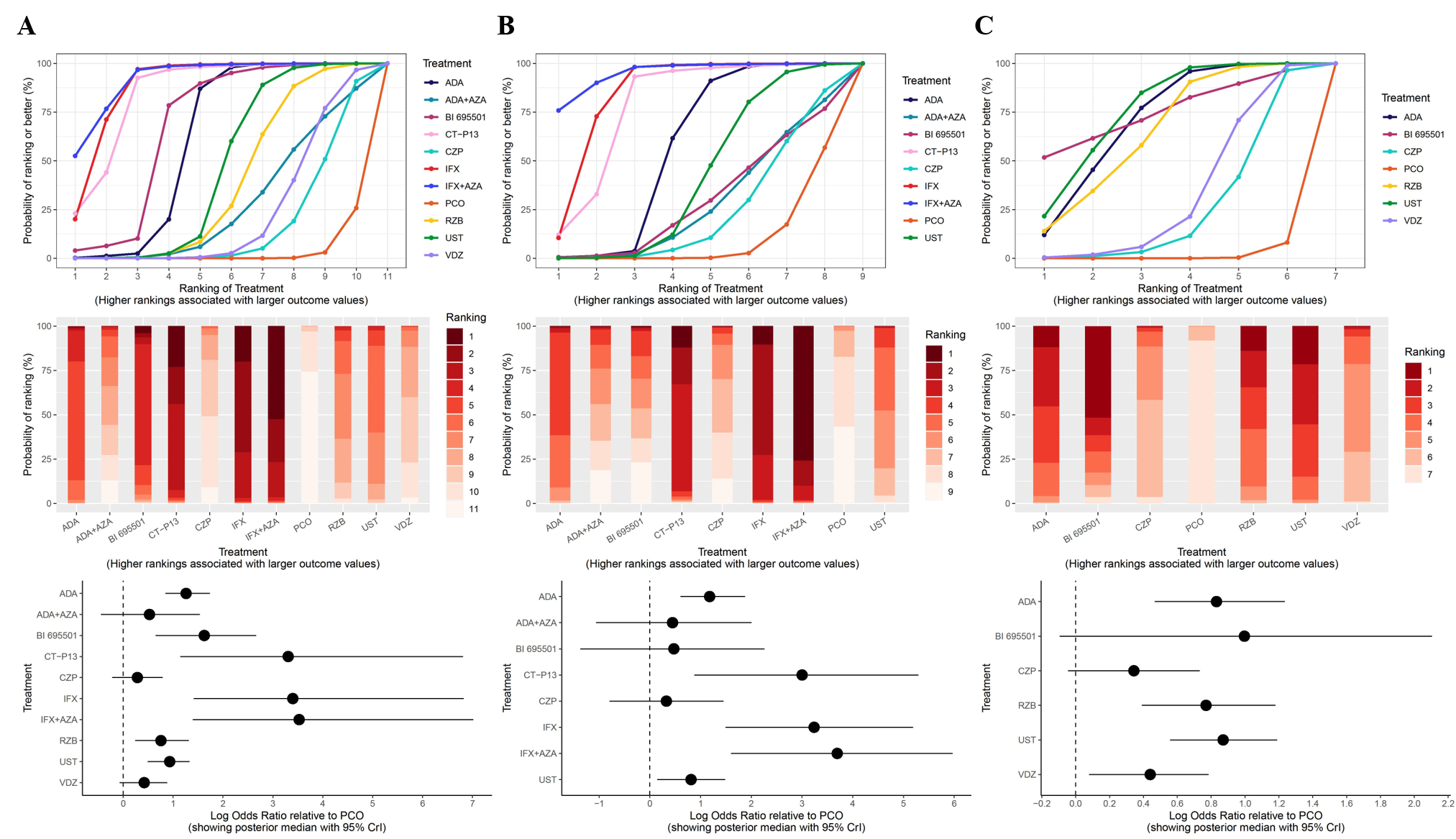


**Supplementary Figure 34.** Sets of plots (including SUCRA plot, rankogram, and forest plot from top to bottom) of mixed comparisons of (A) risk of adverse events, (B) risk of serious adverse events, (C) risk of serious infections, in induction therapy between included biologic agents in overall patients with moderate-to-severe Crohn’s disease.


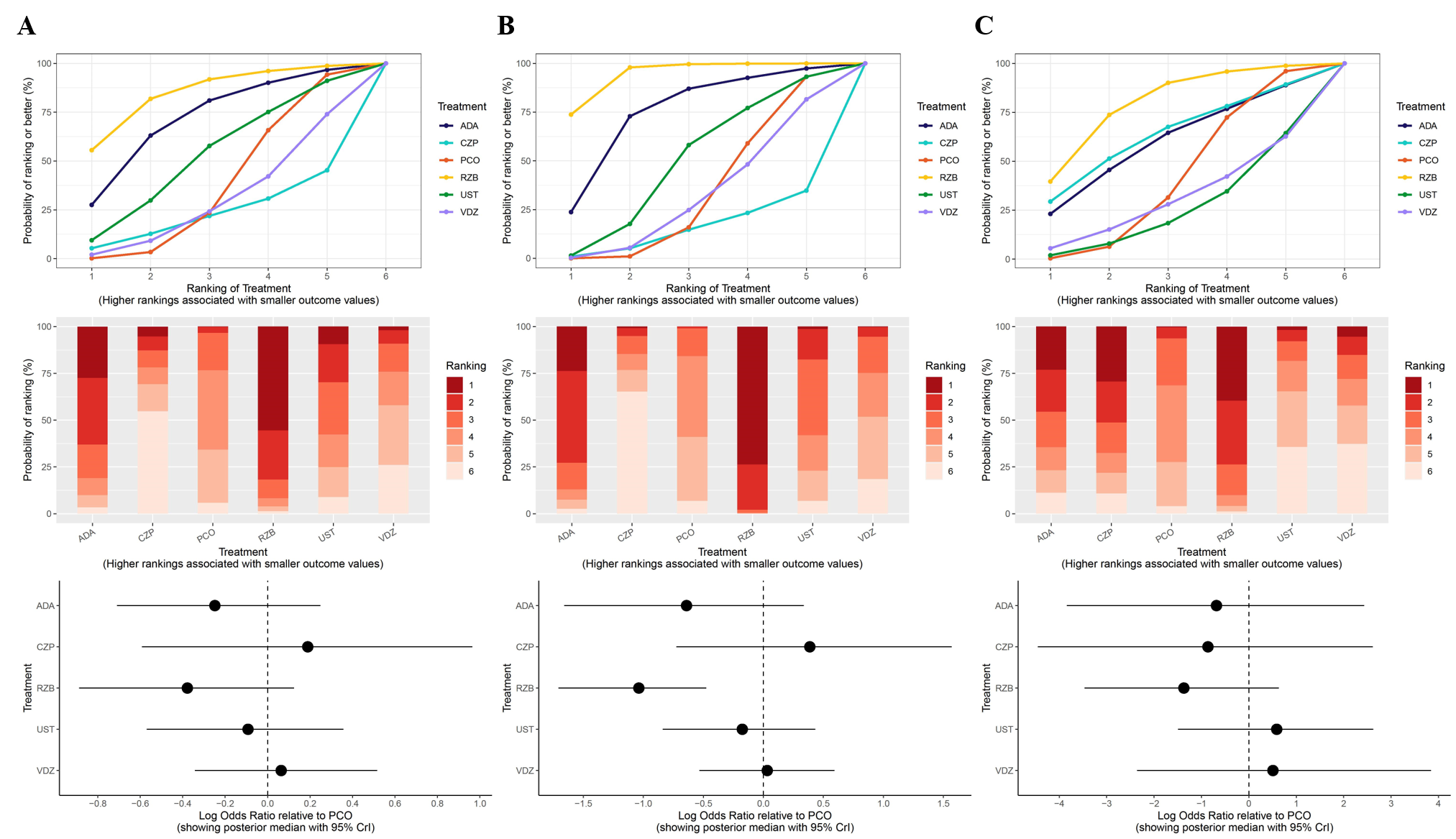


**Supplementary Figure 35.** Sets of plots (including SUCRA plot, rankogram, and forest plot from top to bottom) of mixed comparisons of (A) maintaining clinical remission, (B) maintaining CDAI-70, (C) maintaining CDAI-100, between included biologic agents in overall patients with moderate-to-severe Crohn’s disease.


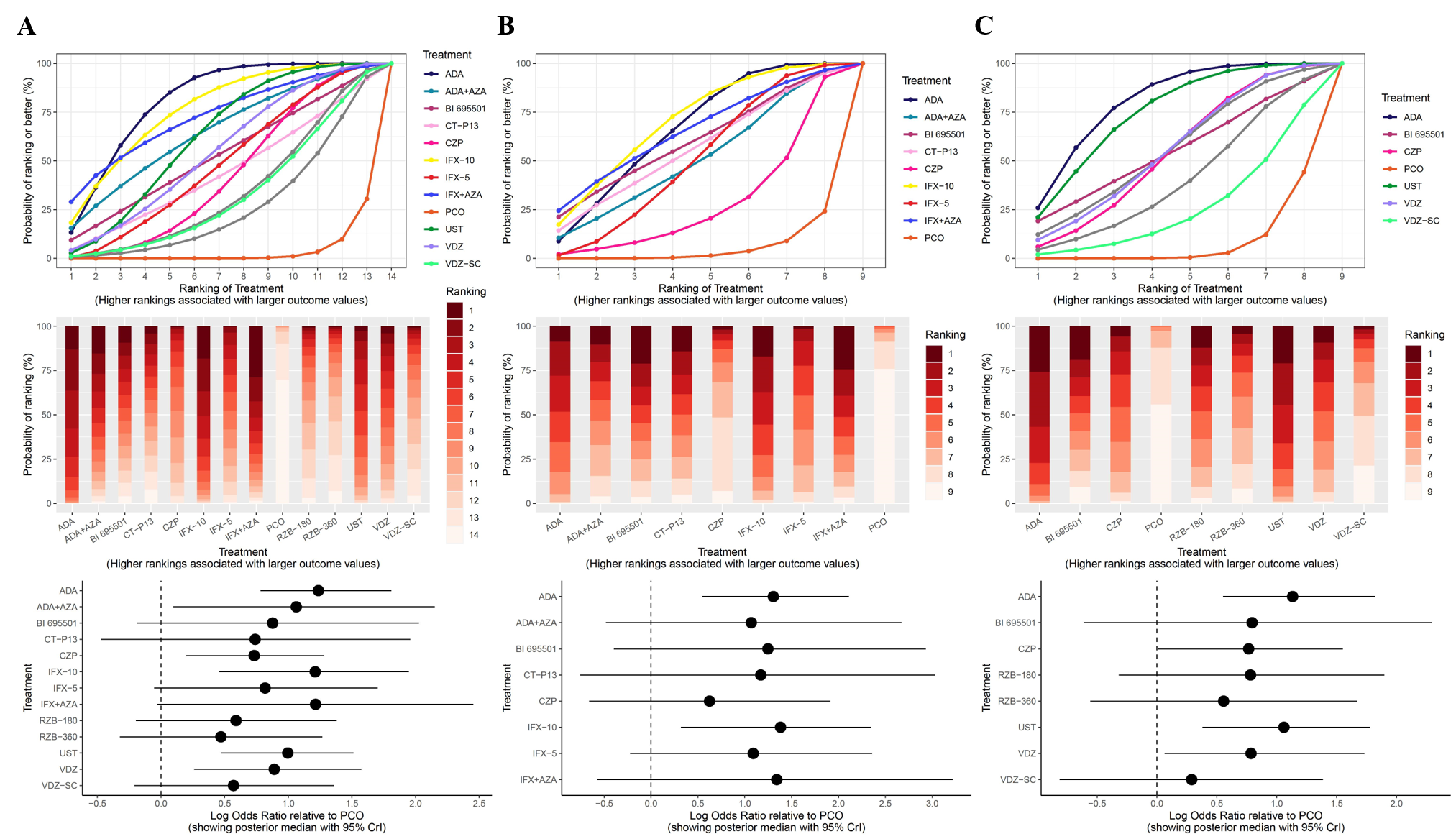


**Supplementary Figure 36.** Sets of plots (including SUCRA plot, rankogram, and forest plot from top to bottom) of mixed comparisons of (A) risk of adverse events, (B) risk of serious adverse events, (C) risk of serious infections, in maintenance therapy between included biologic agents in overall patients with moderate-to-severe Crohn’s disease.


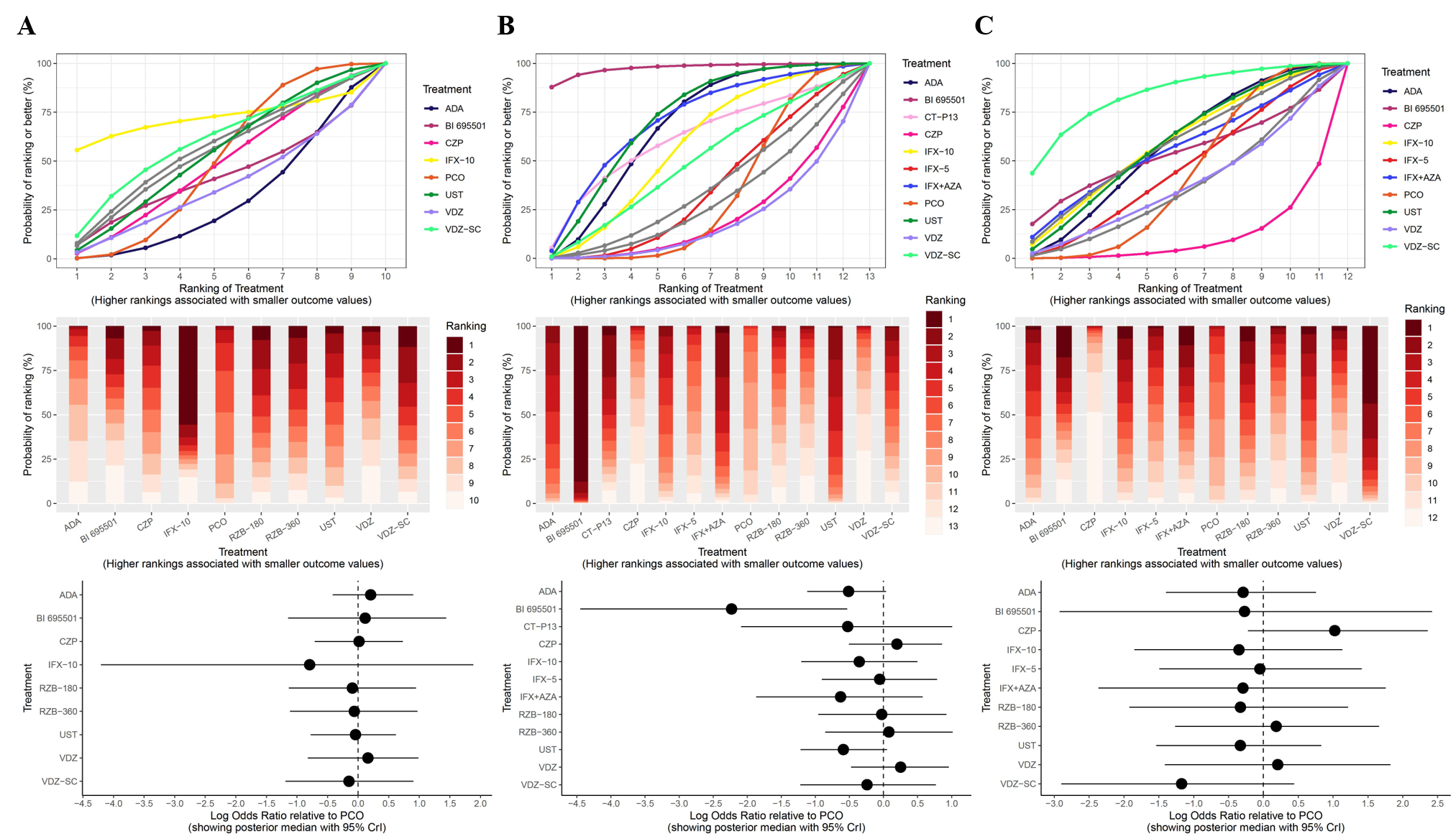


**Supplementary Figure 37.** Sets of plots (including SUCRA plot, rankogram, and forest plot from top to bottom) of mixed comparisons of (A) inducing clinical remission, (B) inducing CDAI-70, (C) inducing CDAI-100, between included biologic agents in tumor necrosis factor antagonist-naïve patients with moderate-to-severe Crohn’s disease.


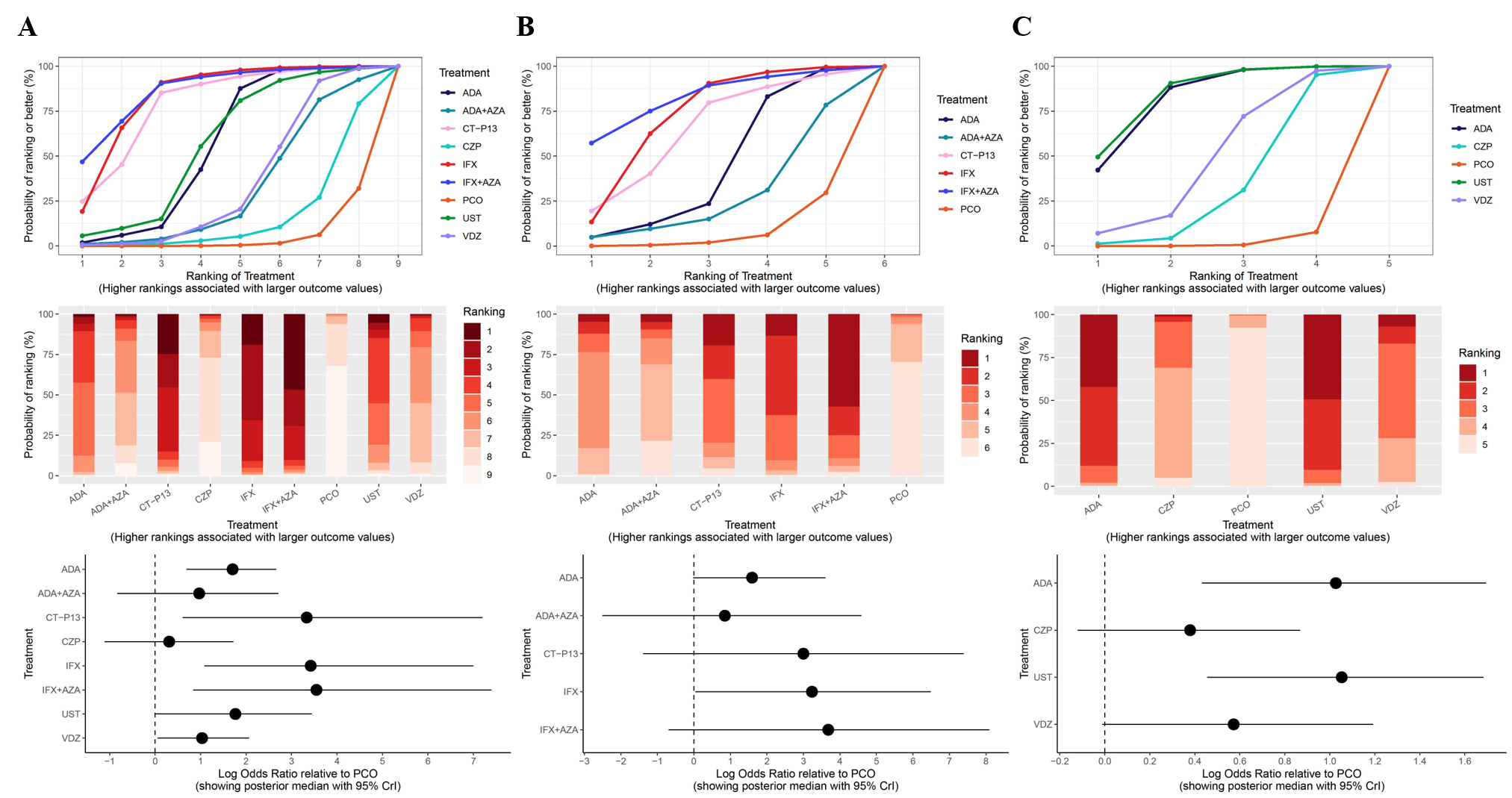


**Supplementary Figure 38.** Sets of plots (including SUCRA plot, rankogram, and forest plot from top to bottom) of mixed comparisons of (A) inducing clinical remission, (B) inducing CDAI-70, (C) inducing CDAI-100, between included biologic agents in tumor necrosis factor antagonist-experienced patients with moderate-to-severe Crohn’s disease.


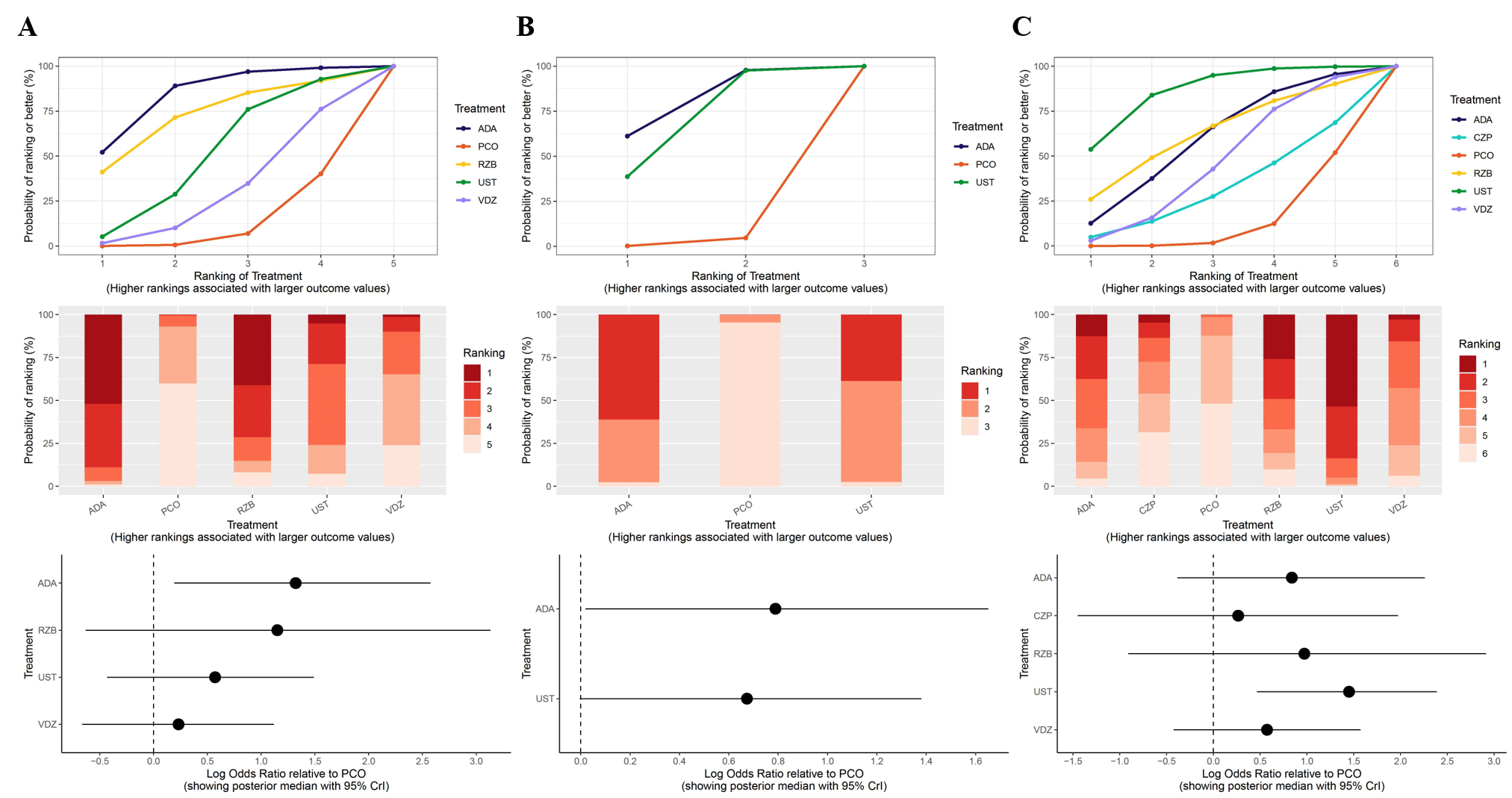


**Supplementary Figure 39.** Sets of plots (including SUCRA plot, rankogram, and forest plot from top to bottom) of mixed comparisons of inducing clinical remission, between included biologic agents (A) in tumor necrosis factor antagonist-naïve patients, (B) in tumor necrosis factor antagonist-experienced patients, with moderate-to-severe Crohn’s disease.


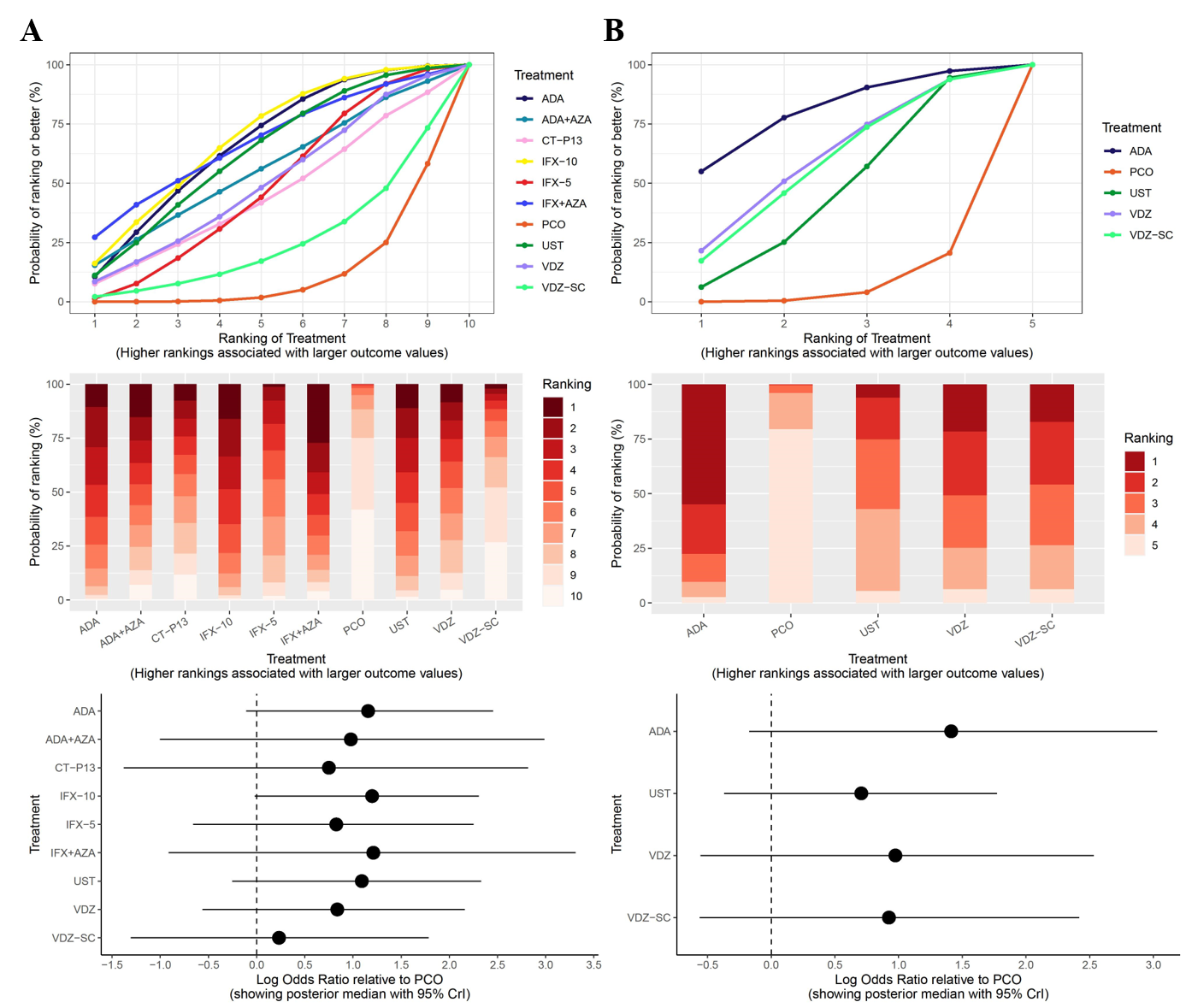


**Supplementary Figure 40.** Consistency vs inconsistency plots of evaluations of (A) inducing clinical remission, (B) inducing CDAI-70, (C) inducing CDAI-100, (D) risk of adverse events in induction therapy, (E) risk of serious adverse events in induction therapy, (F) risk of serious infections in induction therapy, (G) maintaining clinical remission, (H) maintaining CDAI-70, (I) maintaining CDAI-100, (J) risk of adverse events in maintenance therapy, (K) risk of serious adverse events in maintenance therapy, (L) risk of serious infections in maintenance therapy in over patients with Crohn’s disease; (M) inducing clinical remission, (N) inducing CDAI-70, (O) inducing CDAI-100, (P) maintaining clinical remission, in tumor necrosis factor antagonist-naïve patients with Crohn’s disease; (Q) inducing clinical remission, (R) inducing CDAI-70, (S) inducing CDAI-100, (T) maintaining clinical remission, in tumor necrosis factor antagonist-experienced patients with Crohn’s disease.


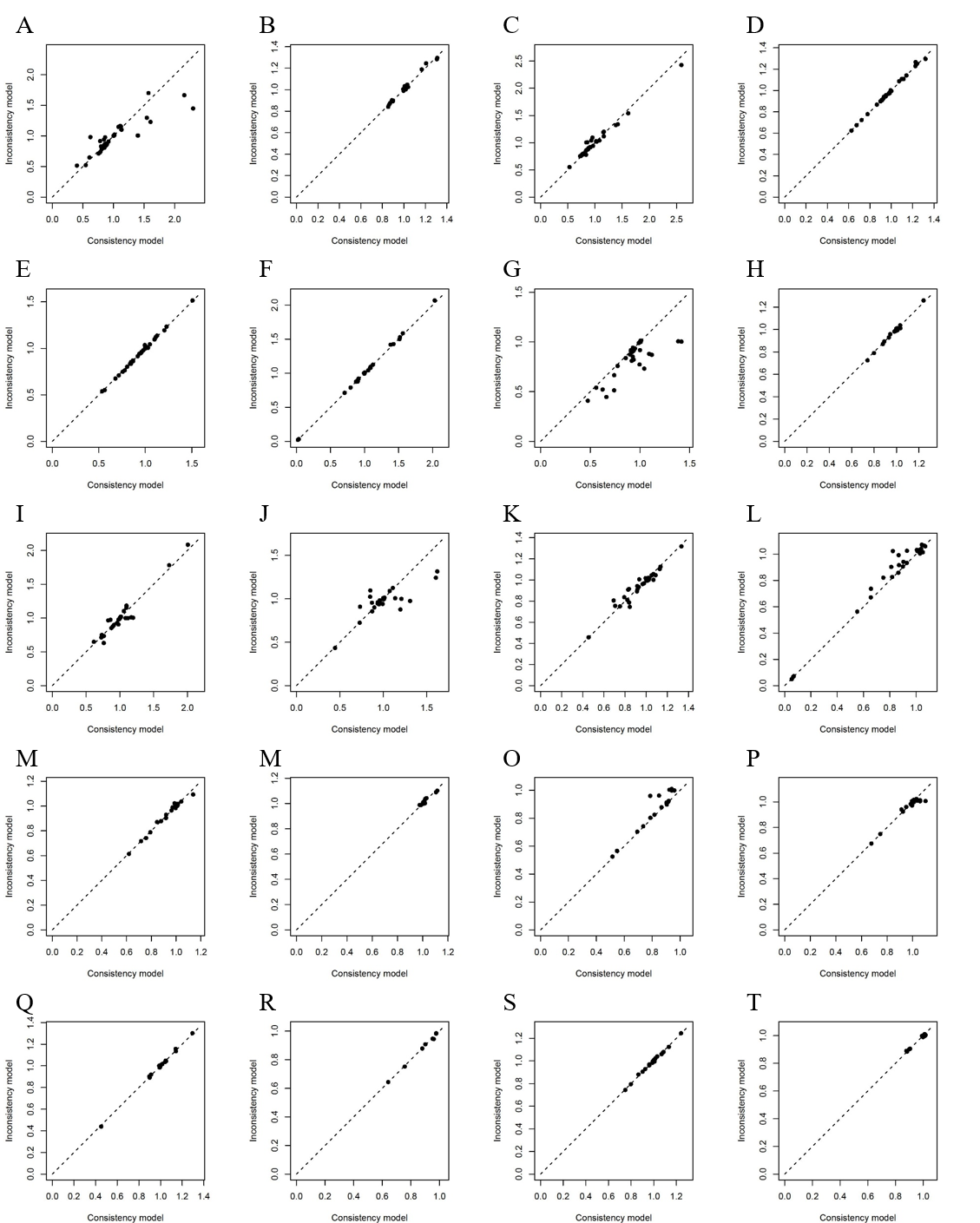


**Supplementary Table 2.** Certainty of evidence on the efficacy of biologic agents in the induction therapy of overall patients with moderate-to-severe Crohn’s disease, based on GRADE approach for network meta-analysis.

| Intervention | Induction of clinical remission | | | Induction of CDAI-70 | | | Induction of CDAI-100 | | |
| --- | --- | --- | --- | --- | --- | --- | --- | --- | --- |
|  | Direct | Indirect | Network | Direct | Indirect | Network | Direct | Indirect | Network |
| Compared with Infliximab | | | | | | | | | |
| Infliximab+azathioprine | LOW^a^ | N/A | LOW^a^ | MODERATE^c^ | N/A | MODERATE^c^ | N/A | N/A | N/A |
| Adalimumab | N/A | VERY LOW^e,f^ | VERY LOW^e,f^ | N/A | VERY LOW^e,f^ | VERY LOW^e,f^ | N/A | N/A | N/A |
| Adalimumab+azathioprine | N/A | VERY LOW^e,f,g^ | VERY LOW^e,f,g^ | N/A | VERY LOW^e,f,g^ | VERY LOW^e,f,g^ | N/A | N/A | N/A |
| Certolizumab pegol | N/A | VERY LOW^e^ | VERY LOW^e^ | N/A | VERY LOW^e^ | VERY LOW^e^ | N/A | N/A | N/A |
| CT-P13 | MODERATE^a^ | N/A | MODERATE^a^ | MODERATE^a^ | N/A | MODERATE^a^ | N/A | N/A | N/A |
| BI 695501 | N/A | VERY LOW^e,f,g^ | VERY LOW^e,f,g^ | N/A | VERY LOW^e,f,g^ | VERY LOW^e,f,g^ | N/A | N/A | N/A |
| Ustekinumab | N/A | VERY LOW^e^ | VERY LOW^e^ | N/A | VERY LOW^e^ | VERY LOW^e^ | N/A | N/A | N/A |
| Risankizumab | N/A | VERY LOW^e^ | VERY LOW^e^ | N/A | N/A | N/A | N/A | N/A | N/A |
| Vedolizumab | N/A | VERY LOW^e^ | VERY LOW^e^ | N/A | N/A | N/A | N/A | N/A | N/A |
| Placebo | VERY LOW^a,b,c^ | N/A | VERY LOW^a,b,c^ | VERY LOW^a,b,c^ | N/A | VERY LOW^a,b,c^ | N/A | N/A | N/A |
| Compared with Infliximab+azathioprine | | | | | | | | | |
| Adalimumab | N/A | VERY LOW^e,f,g^ | VERY LOW^e,f,g^ | N/A | VERY LOW^e,f,g^ | VERY LOW^e,f,g^ | N/A | N/A | N/A |
| Adalimumab+azathioprine | N/A | VERY LOW^e,f,g^ | VERY LOW^e,f,g^ | N/A | VERY LOW^e,f,g^ | VERY LOW^e,f,g^ | N/A | N/A | N/A |
| Certolizumab pegol | N/A | VERY LOW^e,g^ | VERY LOW^e,g^ | N/A | VERY LOW^e,g^ | VERY LOW^e,g^ | N/A | N/A | N/A |
| CT-P13 | N/A | VERY LOW^e^ | VERY LOW^e^ | N/A | VERY LOW^e^ | VERY LOW^e^ | N/A | N/A | N/A |
| BI 695501 | N/A | VERY LOW^e,f,g^ | VERY LOW^e,f,g^ | N/A | VERY LOW^e,f,g^ | VERY LOW^e,f,g^ | N/A | N/A | N/A |
| Ustekinumab | N/A | VERY LOW^e,g^ | VERY LOW^e,g^ | N/A | VERY LOW^e,g^ | VERY LOW^e,g^ | N/A | N/A | N/A |
| Risankizumab | N/A | VERY LOW^e,g^ | VERY LOW^e,g^ | N/A | N/A | N/A | N/A | N/A | N/A |
| Vedolizumab | N/A | VERY LOW^e,g^ | VERY LOW^e,g^ | N/A | N/A | N/A | N/A | N/A | N/A |
| Placebo | N/A | VERY LOW^e^ | VERY LOW^e^ | N/A | VERY LOW^e^ | VERY LOW^e^ | N/A | N/A | N/A |
| Compared with Adalimumab | | | | | | | | | |
| Adalimumab+azathioprine | LOW^a,d^ | N/A | LOW^a,d^ | LOW^a,d^ | N/A | LOW^a,d^ | N/A | N/A | N/A |
| Certolizumab pegol | N/A | VERY LOW^f^ | VERY LOW^f^ | N/A | MODERATE^f^ | MODERATE^f^ | N/A | LOW^f^ | LOW^f^ |
| CT-P13 | N/A | VERY LOW^e,f,g^ | VERY LOW^e,f,g^ | N/A | VERY LOW^e,f,g^ | VERY LOW^e,f,g^ | N/A | N/A | N/A |
| BI 695501 | LOW^a,c^ | N/A | LOW^a,c^ | MODERATE^a^ | N/A | MODERATE^a^ | LOW^a,c^ | N/A | LOW^a,c^ |
| Ustekinumab | MODERATE^a^ | VERY LOW^f^ | MODERATE^a^ | N/A | MODERATE^f^ | MODERATE^f^ | HIGH | LOW^f^ | HIGH |
| Risankizumab | N/A | VERY LOW^f^ | VERY LOW^f^ | N/A | N/A | N/A | N/A | LOW^f^ | LOW^f^ |
| Vedolizumab | N/A | VERY LOW^f^ | VERY LOW^f^ | N/A | N/A | N/A | N/A | LOW^f^ | LOW^f^ |
| Placebo | LOW^a,c^ | LOW^e^ | LOW^e^ | HIGH | N/A | HIGH | MODERATE^a^ | LOW^e^ | MODERATE^a^ |
| Compared with Adalimumab+azathioprine | | | | | | | | | |
| Certolizumab pegol | N/A | VERY LOW^e,f,g^ | VERY LOW^e,f,g^ | N/A | VERY LOW^e,f,g^ | VERY LOW^e,f,g^ | N/A | N/A | N/A |
| CT-P13 | N/A | VERY LOW^e,f,g^ | VERY LOW^e,f,g^ | N/A | VERY LOW^e,f,g^ | VERY LOW^e,f,g^ | N/A | N/A | N/A |
| BI 695501 | N/A | VERY LOW^e^ | VERY LOW^e^ | N/A | VERY LOW^e^ | VERY LOW^e^ | N/A | N/A | N/A |
| Ustekinumab | N/A | VERY LOW^e^ | VERY LOW^e^ | N/A | VERY LOW^e,f,g^ | VERY LOW^e,f,g^ | N/A | N/A | N/A |
| Risankizumab | N/A | VERY LOW^e,f,g^ | VERY LOW^e,f,g^ | N/A | N/A | N/A | N/A | N/A | N/A |
| Vedolizumab | N/A | VERY LOW^e,f,g^ | VERY LOW^e,f,g^ | N/A | N/A | N/A | N/A | N/A | N/A |
| Placebo | N/A | VERY LOW^e^ | VERY LOW^e^ | N/A | VERY LOW^e,f^ | VERY LOW^e,f^ | N/A | N/A | N/A |
| Compared with Certolizumab pegol | | | | | | | | | |
| CT-P13 | N/A | VERY LOW^e,g^ | VERY LOW^e,g^ | N/A | VERY LOW^e,g^ | VERY LOW^e,g^ | N/A | N/A | N/A |
| BI 695501 | N/A | VERY LOW^f,g^ | VERY LOW^f,g^ | N/A | LOW^f,g^ | LOW^f,g^ | N/A | VERY LOW^f,g^ | VERY LOW^f,g^ |
| Ustekinumab | N/A | HIGH | HIGH | N/A | HIGH | HIGH | N/A | MODERATE | MODERATE |
| Risankizumab | N/A | MODERATE | MODERATE | N/A | N/A | N/A | N/A | HIGH | HIGH |
| Vedolizumab | N/A | MODERATE | MODERATE | N/A | N/A | N/A | N/A | HIGH | HIGH |
| Placebo | HIGH | N/A | HIGH | HIGH | N/A | HIGH | HIGH | N/A | HIGH |
| Compared with CT-P13 | | | | | | | | | |
| BI 695501 | N/A | VERY LOW^e,f,g^ | VERY LOW^e,f,g^ | N/A | VERY LOW^e,f,g^ | VERY LOW^e,f,g^ | N/A | N/A | N/A |
| Ustekinumab | N/A | VERY LOW^e,g^ | VERY LOW^e,g^ | N/A | VERY LOW^e,g^ | VERY LOW^e,g^ | N/A | N/A | N/A |
| Risankizumab | N/A | VERY LOW^e,g^ | VERY LOW^e,g^ | N/A | N/A | N/A | N/A | N/A | N/A |
| Vedolizumab | N/A | VERY LOW^e,g^ | VERY LOW^e,g^ | N/A | N/A | N/A | N/A | N/A | N/A |
| Placebo | N/A | VERY LOW^e^ | VERY LOW^e^ | N/A | VERY LOW^e^ | VERY LOW^e^ | N/A | N/A | N/A |
| Compared with BI 695501 | | | | | | | | | |
| Ustekinumab | N/A | VERY LOW^e^ | VERY LOW^e^ | N/A | VERY LOW^f,g^ | VERY LOW^f,g^ | N/A | VERY LOW^e^ | VERY LOW^e^ |
| Risankizumab | N/A | VERY LOW^f,g^ | VERY LOW^f,g^ | N/A | N/A | N/A | N/A | VERY LOW^f,g^ | VERY LOW^f,g^ |
| Vedolizumab | N/A | VERY LOW^f,g^ | VERY LOW^f,g^ | N/A | N/A | N/A | N/A | VERY LOW^f,g^ | VERY LOW^f,g^ |
| Placebo | N/A | VERY LOW^f^ | VERY LOW^f^ | N/A | LOW^f^ | LOW^f^ | N/A | VERY LOW^f^ | VERY LOW^f^ |
| Compared with Ustekinumab | | | | | | | | | |
| Risankizumab | N/A | MODERATE | MODERATE | N/A | N/A | N/A | N/A | MODERATE | MODERATE |
| Vedolizumab | N/A | MODERATE | MODERATE | N/A | N/A | N/A | N/A | MODERATE | MODERATE |
| Placebo | HIGH | VERY LOW^e^ | HIGH | HIGH | N/A | HIGH | MODERATE^c^ | LOW^e^ | MODERATE^c^ |
| Compared with Risankizumab | | | | | | | | | |
| Vedolizumab | N/A | MODERATE | MODERATE | N/A | N/A | N/A | N/A | HIGH | HIGH |
| Placebo | MODERATE^a^ | N/A | MODERATE^a^ | N/A | N/A | N/A | HIGH | N/A | HIGH |
| Compared with Vedolizumab | | | | | | | | | |
| Placebo | MODERATE^a^ | N/A | MODERATE^a^ | N/A | N/A | N/A | HIGH | N/A | HIGH |

a. Rated down for imprecision since optimal information size not met (<200 events).

b. Rated down for imprecision due to wide confidence intervals.

c. Rated down for publication bias strongly suspected.

d. Rated down for serious risk of bias.

e. Rated down for indirectness due to intransitivity from different patient history of TNF antagonist treatment.

f. Rated down for indirectness due to the GAIN trial selectively include the patients with intolerance or secondary non-response to Infliximab.

g. Evidence derived from second/higher-order loops, rated down for very serious indirectness.

**Supplementary Table 3.** Certainty of evidence on the efficacy of biologic agents in the induction therapy of TNF antagonist-naïve patients with moderate-to-severe Crohn’s disease, based on GRADE approach for network meta-analysis.

| Intervention | Induction of clinical remission | | | Induction of CDAI-70 | | | Induction of CDAI-100 | | |
| --- | --- | --- | --- | --- | --- | --- | --- | --- | --- |
|  | Direct | Indirect | Network | Direct | Indirect | Network | Direct | Indirect | Network |
| Compared with Infliximab | | | | | | | | | |
| Infliximab+azathioprine | MODERATE^a^ | N/A | MODERATE^a^ | HIGH | N/A | HIGH | N/A | N/A | N/A |
| Adalimumab | N/A | LOW | LOW | N/A | VERY LOW^e^ | VERY LOW^e^ | N/A | N/A | N/A |
| Adalimumab+azathioprine | N/A | VERY LOW^e^ | VERY LOW^e^ | N/A | VERY LOW^e^ | VERY LOW^e^ | N/A | N/A | N/A |
| Certolizumab pegol | N/A | LOW | LOW | N/A | N/A | N/A | N/A | N/A | N/A |
| CT-P13 | MODERATE^a^ | N/A | MODERATE^a^ | MODERATE^a^ | N/A | MODERATE^a^ | N/A | N/A | N/A |
| Ustekinumab | N/A | VERY LOW^e^ | VERY LOW^e^ | N/A | N/A | N/A | N/A | N/A | N/A |
| Vedolizumab | N/A | LOW | LOW | N/A | N/A | N/A | N/A | N/A | N/A |
| Placebo | LOW^a,b^ | N/A | LOW^a,b^ | VERY LOW^a,b,c^ | N/A | VERY LOW^a,b,c^ | N/A | N/A | N/A |
| Compared with Infliximab+azathioprine | | | | | | | | | |
| Adalimumab | N/A | VERY LOW^e^ | VERY LOW^e^ | N/A | VERY LOW^e^ | VERY LOW^e^ | N/A | N/A | N/A |
| Adalimumab+azathioprine | N/A | VERY LOW^e^ | VERY LOW^e^ | N/A | VERY LOW^e^ | VERY LOW^e^ | N/A | N/A | N/A |
| Certolizumab pegol | N/A | VERY LOW^e^ | VERY LOW^e^ | N/A | N/A | N/A | N/A | N/A | N/A |
| CT-P13 | N/A | MODERATE | MODERATE | N/A | N/A | N/A | N/A | N/A | N/A |
| Ustekinumab | N/A | VERY LOW^e^ | VERY LOW^e^ | N/A | N/A | N/A | N/A | N/A | N/A |
| Vedolizumab | N/A | VERY LOW^e^ | VERY LOW^e^ | N/A | N/A | N/A | N/A | N/A | N/A |
| Placebo | N/A | LOW | LOW | N/A | N/A | N/A | N/A | N/A | N/A |
| Compared with Adalimumab | | | | | | | | | |
| Adalimumab+azathioprine | LOW^a,d^ | N/A | LOW^a,d^ | LOW^a,d^ | N/A | LOW^a,d^ | N/A | N/A | N/A |
| Certolizumab pegol | N/A | MODERATE | MODERATE | N/A | N/A | N/A | N/A | MODERATE | MODERATE |
| CT-P13 | N/A | VERY LOW^e^ | VERY LOW^e^ | N/A | N/A | N/A | N/A | N/A | N/A |
| Ustekinumab | MODERATE^a^ | N/A | MODERATE^a^ | N/A | N/A | N/A | HIGH | MODERATE | HIGH |
| Vedolizumab | N/A | MODERATE | MODERATE | N/A | N/A | N/A | N/A | MODERATE | MODERATE |
| Placebo | MODERATE^a^ | N/A | MODERATE^a^ | MODERATE^a^ | N/A | MODERATE^a^ | MODERATE^a^ | MODERATE | MODERATE^a^ |
| Compared with Adalimumab+azathioprine | | | | | | | | | |
| Certolizumab pegol | N/A | VERY LOW^e^ | VERY LOW^e^ | N/A | N/A | N/A | N/A | N/A | N/A |
| CT-P13 | N/A | VERY LOW^e^ | VERY LOW^e^ | N/A | N/A | N/A | N/A | N/A | N/A |
| Ustekinumab | N/A | LOW | LOW | N/A | N/A | N/A | N/A | N/A | N/A |
| Vedolizumab | N/A | VERY LOW^e^ | VERY LOW^e^ | N/A | N/A | N/A | N/A | N/A | N/A |
| Placebo | N/A | LOW | LOW | N/A | N/A | N/A | N/A | N/A | N/A |
| Compare with Certolizumab pegol | | | | | | | | | |
| CT-P13 | N/A | VERY LOW^e^ | VERY LOW^e^ | N/A | N/A | N/A | N/A | N/A | N/A |
| Ustekinumab | N/A | LOW^e^ | LOW^e^ | N/A | N/A | N/A | N/A | MDERATE | MDERATE |
| Vedolizumab | N/A | MODERATE | MODERATE | N/A | N/A | N/A | N/A | MODERATE | MODERATE |
| Placebo | MODERATE^a^ | N/A | MODERATE^a^ | N/A | N/A | N/A | HIGH | N/A | HIGH |
| Compared with CT-P13 | | | | | | | | | |
| Ustekinumab | N/A | VERY LOW^e^ | VERY LOW^e^ | N/A | N/A | N/A | N/A | N/A | N/A |
| Vedolizumab | N/A | VERY LOW^e^ | VERY LOW^e^ | N/A | N/A | N/A | N/A | N/A | N/A |
| Placebo | N/A | LOW | LOW | N/A | N/A | N/A | N/A | N/A | N/A |
| Compared with Ustekinumab | | | | | | | | | |
| Vedolizumab | N/A | LOW^e^ | LOW^e^ | N/A | N/A | N/A | N/A | MODERATE | MODERATE |
| Placebo | N/A | MODERATE | MODERATE | N/A | N/A | N/A | MODERATE^a^ | MODERATE | MODERATE^a^ |
| Compared with Vedolizumab | | | | | | | | | |
| Placebo | MODERATE^a^ | N/A | MODERATE^a^ | N/A | N/A | N/A | MODERATE^a^ | N/A | MODERATE^a^ |

a. Rated down for imprecision since optimal information size not met (<200 events).

b. Rated down for imprecision due to wide confidence intervals.

c. Rated down for publication bias strongly suspected.

d. Rated down for serious risk of bias.

e. Evidence derived from second/higher-order loops, rated down for very serious indirectness.

**Supplementary Table 4.** Certainty of evidence on the efficacy of biologic agents in the induction therapy of TNF antagonist-experienced patients with moderate-to-severe Crohn’s disease, based on GRADE approach for network meta-analysis.

| Intervention | Induction of clinical remission | | | Induction of CDAI-70 | | | Induction of CDAI-100 | | |
| --- | --- | --- | --- | --- | --- | --- | --- | --- | --- |
|  | Direct | Indirect | Network | Direct | Indirect | Network | Direct | Indirect | Network |
| Compared with Adalimumab | | | | | | | | | |
| Certolizumab pegol | N/A | N/A | N/A | N/A | N/A | N/A | N/A | LOW^c^ | LOW^c^ |
| Ustekinumab | N/A | VERY LOW^c^ | VERY LOW^c^ | N/A | MODERATE^c^ | MODERATE^c^ | N/A | LOW^c^ | LOW^c^ |
| Risankizumab | N/A | LOW^c^ | LOW^c^ | N/A | N/A | N/A | N/A | LOW^c^ | LOW^c^ |
| Vedolizumab | N/A | LOW^c^ | LOW^c^ | N/A | N/A | N/A | N/A | LOW^c^ | LOW^c^ |
| Placebo | MODERATE^a^ | N/A | MODERATE^a^ | MODERATE^a^ | N/A | MODERATE^a^ | MODERATE^a^ | N/A | MODERATE^a^ |
| Compared with Certolizumab pegol | | | | | | | | | |
| Ustekinumab | N/A | N/A | N/A | N/A | N/A | N/A | N/A | MODERATE | MODERATE |
| Risankizumab | N/A | N/A | N/A | N/A | N/A | N/A | N/A | MODERATE | MODERATE |
| Vedolizumab | N/A | N/A | N/A | N/A | N/A | N/A | N/A | MODERATE | MODERATE |
| Placebo | N/A | N/A | N/A | N/A | N/A | N/A | MODERATE^a^ | N/A | MODERATE^a^ |
| Compared with Ustekinumab | | | | | | | | | |
| Risankizumab | N/A | LOW | LOW | N/A | N/A | N/A | N/A | MODERATE | MODERATE |
| Vedolizumab | N/A | LOW | LOW | N/A | N/A | N/A | N/A | MODERATE | MODERATE |
| Placebo | LOW^a,b^ | N/A | LOW^a,b^ | HIGH | N/A | HIGH | MODERATE^b^ | N/A | MODERATE^b^ |
| Compared with Risankizumab | | | | | | | | | |
| Vedolizumab | N/A | MODERATE | MODERATE | N/A | N/A | N/A | N/A | MODERATE | MODERATE |
| Placebo | MODERATE^a^ | N/A | MODERATE^a^ | N/A | N/A | N/A | MODERATE^a^ | N/A | MODERATE^a^ |
| Compared with Vedolizumab | | | | | | | | | |
| Placebo | MODERATE^a^ | N/A | MODERATE^a^ | N/A | N/A | N/A | MODERATE^a^ | N/A | MODERATE^a^ |

a. Rated down for imprecision since optimal information size not met (<200 events).

b. Rated down for publication bias strongly suspected.

c. Rated down for indirectness due to the GAIN trial selectively include the patients with intolerance or secondary non-response to Infliximab.

**Supplementary Table 5.** Certainty of evidence on the efficacy of biologic agents in the maintenance therapy of overall patients with moderate-to-severe Crohn’s disease, based on GRADE approach for network meta-analysis.

| Intervention | Maintenance of clinical remission | | | Maintenance of CDAI-70 | | | Maintenance of CDAI-100 | | |
| --- | --- | --- | --- | --- | --- | --- | --- | --- | --- |
|  | Direct | Indirect | Network | Direct | Indirect | Network | Direct | Indirect | Network |
| Compared with IFX-5 | | | | | | | | | |
| IFX-10 | LOW^a,b^ | LOW | LOW^a,b^ | LOW^a,b^ | LOW | LOW^a,b^ | N/A | N/A | N/A |
| Infliximab+azathioprine | LOW^a,b^ | N/A | LOW^a,b^ | MODERATE^a^ | N/A | MODERATE^a^ | N/A | N/A | N/A |
| Adalimumab | N/A | LOW | LOW | N/A | MODERATE | MODERATE | N/A | N/A | N/A |
| Adalimumab+azathioprine | N/A | VERY LOW^d,e^ | VERY LOW^d,e^ | N/A | VERY LOW^d,e^ | VERY LOW^d,e^ | N/A | N/A | N/A |
| Certolizumab pegol | N/A | MODERATE | MODERATE | N/A | MODERATE | MODERATE | N/A | N/A | N/A |
| CT-P13 | MODERATE^a^ | N/A | MODERATE^a^ | MODERATE^a^ | N/A | MODERATE^a^ | N/A | N/A | N/A |
| BI 695501 | N/A | VERY LOW^e^ | VERY LOW^e^ | N/A | LOW^e^ | LOW^e^ | N/A | N/A | N/A |
| Ustekinumab | N/A | MODERATE | MODERATE | N/A | N/A | N/A | N/A | N/A | N/A |
| RZB-180 | N/A | MODERATE | MODERATE | N/A | N/A | N/A | N/A | N/A | N/A |
| RZB-360 | N/A | MODERATE | MODERATE | N/A | N/A | N/A | N/A | N/A | N/A |
| Vedolizumab | N/A | MODERATE | MODERATE | N/A | N/A | N/A | N/A | N/A | N/A |
| VDZ-SC | N/A | MODERATE | MODERATE | N/A | N/A | N/A | N/A | N/A | N/A |
| Placebo | MODERATE^a^ | LOW | MODERATE^a^ | MODERATE^a^ | LOW | MODERATE^a^ | N/A | N/A | N/A |
| Compared with IFX-10 | | | | | | | | | |
| Infliximab+azathioprine | N/A | VERY LOW^d^ | VERY LOW^d^ | N/A | VERY LOW^d^ | VERY LOW^d^ | N/A | N/A | N/A |
| Adalimumab | N/A | LOW | LOW | N/A | LOW | LOW | N/A | N/A | N/A |
| Adalimumab+azathioprine | N/A | VERY LOW^d,e^ | VERY LOW^d,e^ | N/A | VERY LOW^d,e^ | VERY LOW^d,e^ | N/A | N/A | N/A |
| Certolizumab pegol | N/A | LOW | LOW | N/A | LOW | LOW | N/A | N/A | N/A |
| CT-P13 | N/A | VERY LOW^d^ | VERY LOW^d^ | N/A | VERY LOW^d^ | VERY LOW^d^ | N/A | N/A | N/A |
| BI 695501 | N/A | VERY LOW^e^ | VERY LOW^e^ | N/A | VERY LOW^e^ | VERY LOW^e^ | N/A | N/A | N/A |
| Ustekinumab | N/A | LOW | LOW | N/A | N/A | N/A | N/A | N/A | N/A |
| RZB-180 | N/A | LOW | LOW | N/A | N/A | N/A | N/A | N/A | N/A |
| RZB-360 | N/A | MODERATE | MODERATE | N/A | N/A | N/A | N/A | N/A | N/A |
| Vedolizumab | N/A | MODERATE | MODERATE | N/A | N/A | N/A | N/A | N/A | N/A |
| VDZ-SC | N/A | MODERATE | MODERATE | N/A | N/A | N/A | N/A | N/A | N/A |
| Placebo | LOW^a,c^ | LOW | LOW^a,c^ | LOW^a,c^ | LOW | LOW^a,c^ | N/A | N/A | N/A |
| Compared with Infliximab+azathioprine | | | | | | | | | |
| Adalimumab | N/A | VERY LOW^d,e^ | VERY LOW^d,e^ | N/A | VERY LOW^d,e^ | VERY LOW^d,e^ | N/A | N/A | N/A |
| Adalimumab+azathioprine | N/A | VERY LOW^d,e^ | VERY LOW^d,e^ | N/A | VERY LOW^d,e^ | VERY LOW^d,e^ | N/A | N/A | N/A |
| Certolizumab pegol | N/A | VERY LOW^d,e^ | VERY LOW^d,e^ | N/A | VERY LOW^d,e^ | VERY LOW^d,e^ | N/A | N/A | N/A |
| CT-P13 | N/A | VERY LOW^d^ | VERY LOW^d^ | N/A | VERY LOW^d^ | VERY LOW^d^ | N/A | N/A | N/A |
| BI 695501 | N/A | VERY LOW^d,e^ | VERY LOW^d,e^ | N/A | VERY LOW^d,e^ | VERY LOW^d,e^ | N/A | N/A | N/A |
| Ustekinumab | N/A | VERY LOW^d,e^ | VERY LOW^d,e^ | N/A | N/A | N/A | N/A | N/A | N/A |
| RZB-180 | N/A | VERY LOW^d,e^ | VERY LOW^d,e^ | N/A | N/A | N/A | N/A | N/A | N/A |
| RZB-360 | N/A | VERY LOW^d,e^ | VERY LOW^d,e^ | N/A | N/A | N/A | N/A | N/A | N/A |
| Vedolizumab | N/A | VERY LOW^d,e^ | VERY LOW^d,e^ | N/A | N/A | N/A | N/A | N/A | N/A |
| VDZ-SC | N/A | VERY LOW^d,e^ | VERY LOW^d,e^ | N/A | N/A | N/A | N/A | N/A | N/A |
| Placebo | N/A | VERY LOW^d^ | VERY LOW^d^ | N/A | LOW^d^ | LOW^d^ | N/A | N/A | N/A |
| Compared with Adalimumab | | | | | | | | | |
| Adalimumab+azathioprine | LOW^a,c^ | N/A | LOW^a,c^ | LOW^a,c^ | N/A | LOW^a,c^ | N/A | N/A | N/A |
| Certolizumab pegol | N/A | LOW | LOW | N/A | MODERATE | MODERATE | N/A | LOW | LOW |
| CT-P13 | N/A | VERY LOW^d,e^ | VERY LOW^d,e^ | N/A | VERY LOW^d,e^ | VERY LOW^d,e^ | N/A | N/A | N/A |
| BI 695501 | MODERATE^a^ | N/A | MODERATE^a^ | MODERATE^a^ | N/A | MODERATE^a^ | MODERATE^a^ | N/A | MODERATE^a^ |
| Ustekinumab | HIGH | LOW | HIGH | N/A | N/A | N/A | HIGH | LOW | HIGH |
| RZB-180 | N/A | LOW | LOW | N/A | N/A | N/A | N/A | LOW | LOW |
| RZB-360 | N/A | LOW | LOW | N/A | N/A | N/A | N/A | LOW | LOW |
| Vedolizumab | N/A | LOW | LOW | N/A | N/A | N/A | N/A | LOW | LOW |
| VDZ-SC | N/A | LOW | LOW | N/A | N/A | N/A | N/A | LOW | LOW |
| Placebo | LOW^a,b^ | LOW^d^ | LOW^d^ | MODERATE^a^ | N/A | MODERATE^a^ | LOW^a,b^ | MODERATE^d^ | MODERATE^d^ |
| Compared with Adalimumab+azathioprine | | | | | | | | | |
| Certolizumab pegol | N/A | VERY LOW^d,e^ | VERY LOW^d,e^ | N/A | VERY LOW^d,e^ | VERY LOW^d,e^ | N/A | N/A | N/A |
| CT-P13 | N/A | VERY LOW^d,e^ | VERY LOW^d,e^ | N/A | VERY LOW^d,e^ | VERY LOW^d,e^ | N/A | N/A | N/A |
| BI 695501 | N/A | VERY LOW^d^ | VERY LOW^d^ | N/A | VERY LOW^d^ | VERY LOW^d^ | N/A | N/A | N/A |
| Ustekinumab | N/A | VERY LOW^d^ | VERY LOW^d^ | N/A | N/A | N/A | N/A | N/A | N/A |
| RZB-180 | N/A | VERY LOW^d,e^ | VERY LOW^d,e^ | N/A | N/A | N/A | N/A | N/A | N/A |
| RZB-360 | N/A | VERY LOW^d,e^ | VERY LOW^d,e^ | N/A | N/A | N/A | N/A | N/A | N/A |
| Vedolizumab | N/A | VERY LOW^d,e^ | VERY LOW^d,e^ | N/A | N/A | N/A | N/A | N/A | N/A |
| VDZ-SC | N/A | VERY LOW^d,e^ | VERY LOW^d,e^ | N/A | N/A | N/A | N/A | N/A | N/A |
| Placebo | N/A | VERY LOW^d^ | VERY LOW^d^ | N/A | VERY LOW^d^ | VERY LOW^d^ | N/A | N/A | N/A |
| Compared with Certolizumab pegol | | | | | | | | | |
| CT-P13 | N/A | VERY LOW^d,e^ | VERY LOW^d,e^ | N/A | VERY LOW^d,e^ | VERY LOW^d,e^ | N/A | N/A | N/A |
| BI 695501 | N/A | VERY LOW^e^ | VERY LOW^e^ | N/A | LOW^e^ | LOW^e^ | N/A | VERY LOW^e^ | VERY LOW^e^ |
| Ustekinumab | N/A | MODERATE | MODERATE | N/A | N/A | N/A | N/A | HIGH | HIGH |
| RZB-180 | N/A | MODERATE | MODERATE | N/A | N/A | N/A | N/A | MODERATE | MODERATE |
| RZB-360 | N/A | MODERATE | MODERATE | N/A | N/A | N/A | N/A | MODERATE | MODERATE |
| Vedolizumab | N/A | MODERATE | MODERATE | N/A | N/A | N/A | N/A | LOW | LOW |
| VDZ-SC | N/A | MODERATE | MODERATE | N/A | N/A | N/A | N/A | HIGH | HIGH |
| Placebo | HIGH | N/A | HIGH | HIGH | N/A | HIGH | HIGH | N/A | HIGH |
| Compared with CT-P13 | | | | | | | | | |
| BI 695501 | N/A | VERY LOW^d,e^ | VERY LOW^d,e^ | N/A | VERY LOW^d,e^ | VERY LOW^d,e^ | N/A | N/A | N/A |
| Ustekinumab | N/A | VERY LOW^d,e^ | VERY LOW^d,e^ | N/A | N/A | N/A | N/A | N/A | N/A |
| RZB-180 | N/A | VERY LOW^d,e^ | VERY LOW^d,e^ | N/A | N/A | N/A | N/A | N/A | N/A |
| RZB-360 | N/A | VERY LOW^d,e^ | VERY LOW^d,e^ | N/A | N/A | N/A | N/A | N/A | N/A |
| Vedolizumab | N/A | VERY LOW^d,e^ | VERY LOW^d,e^ | N/A | N/A | N/A | N/A | N/A | N/A |
| VDZ-SC | N/A | VERY LOW^d,e^ | VERY LOW^d,e^ | N/A | N/A | N/A | N/A | N/A | N/A |
| Placebo | N/A | LOW^d^ | LOW^d^ | N/A | LOW^d^ | LOW^d^ | N/A | N/A | N/A |
| Compared with BI 695501 | | | | | | | | | |
| Ustekinumab | N/A | LOW^d^ | LOW^d^ | N/A | N/A | N/A | N/A | LOW^d^ | LOW^d^ |
| RZB-180 | N/A | VERY LOW^e^ | VERY LOW^e^ | N/A | N/A | N/A | N/A | VERY LOW^e^ | VERY LOW^e^ |
| RZB-360 | N/A | VERY LOW^e^ | VERY LOW^e^ | N/A | N/A | N/A | N/A | VERY LOW^e^ | VERY LOW^e^ |
| Vedolizumab | N/A | VERY LOW^e^ | VERY LOW^e^ | N/A | N/A | N/A | N/A | VERY LOW^e^ | VERY LOW^e^ |
| VDZ-SC | N/A | VERY LOW^e^ | VERY LOW^e^ | N/A | N/A | N/A | N/A | VERY LOW^e^ | VERY LOW^e^ |
| Placebo | N/A | LOW | LOW | N/A | MODERATE | MODERATE | N/A | LOW | LOW |
| Compared with Ustekinumab | | | | | | | | | |
| RZB-180 | N/A | MODERATE | MODERATE | N/A | N/A | N/A | N/A | MODERATE | MODERATE |
| RZB-360 | N/A | MODERATE | MODERATE | N/A | N/A | N/A | N/A | MODERATE | MODERATE |
| Vedolizumab | N/A | MODERATE | MODERATE | N/A | N/A | N/A | N/A | LOW | LOW |
| VDZ-SC | N/A | MODERATE | MODERATE | N/A | N/A | N/A | N/A | HIGH | HIGH |
| Placebo | MODERATE^a^ | VERY LOW^d^ | MODERATE^a^ | N/A | N/A | N/A | HIGH | VERY LOW^d^ | HIGH |
| Compared with RZB-180 | | | | | | | | | |
| RZB-360 | MODERATE^a^ | MODERATE | MODERATE^a^ | N/A | N/A | N/A | MODERATE^a^ | MODERATE | MODERATE^a^ |
| Vedolizumab | N/A | MODERATE | MODERATE | N/A | N/A | N/A | N/A | LOW | LOW |
| VDZ-SC | N/A | MODERATE | MODERATE | N/A | N/A | N/A | N/A | MODERATE | MODERATE |
| Placebo | MODERATE^a^ | MODERATE | MODERATE^a^ | N/A | N/A | N/A | MODERATE^a^ | MODERATE | MODERATE^a^ |
| Compared with RZB-360 | | | | | | | | | |
| Vedolizumab | N/A | MODERATE | MODERATE | N/A | N/A | N/A | N/A | LOW | LOW |
| VDZ-SC | N/A | MODERATE | MODERATE | N/A | N/A | N/A | N/A | MODERATE | MODERATE |
| Placebo | MODERATE^a^ | MODERATE | MODERATE^a^ | N/A | N/A | N/A | MODERATE^a^ | MODERATE | MODERATE^a^ |
| Compared with Vedolizumab | | | | | | | | | |
| VDZ-SC | N/A | MODERATE | MODERATE | N/A | N/A | N/A | N/A | LOW | LOW |
| Placebo | MODERATE^a^ | N/A | MODERATE^a^ | N/A | N/A | N/A | LOW^a,b^ | N/A | LOW^a,b^ |
| Compared with VDZ-SC | | | | | | | | | |
| Placebo | MODERATE^a^ | N/A | MODERATE^a^ | N/A | N/A | N/A | HIGH | N/A | HIGH |

a. Rated down for imprecision since optimal information size not met (<200 events).

b. Rated down for publication bias strongly suspected.

c. Rated down for serious risk of bias.

d. Rated down for indirectness due to intransitivity from different patient history of TNF antagonist treatment.

e. Evidence derived from second/higher-order loops, rated down for very serious indirectness.

**Supplementary Table 6.** Certainty of evidence on the efficacy of biologic agents for the maintenance of clinical remission, based on GRADE approach for network meta-analysis.

| Intervention | Maintenance of clinical remission in TNF antagonist-naïve CD patients | | | Maintenance of clinical remission in TNF antagonist-experienced CD patients | | |
| --- | --- | --- | --- | --- | --- | --- |
|  | Direct | Indirect | Network | Direct | Indirect | Network |
| Compared with IFX-5 | | | | | | |
| IFX-10 | LOW^a,b^ | LOW | LOW^a,b^ | N/A | N/A | N/A |
| Infliximab+azathioprine | MODERATE^a^ | N/A | MODERATE^a^ | N/A | N/A | N/A |
| Adalimumab | N/A | MODERATE | MODERATE | N/A | N/A | N/A |
| Adalimumab+azathioprine | N/A | VERY LOW^d,e^ | VERY LOW^d,e^ | N/A | N/A | N/A |
| CT-P13 | MODERATE^a^ | N/A | MODERATE^a^ | N/A | N/A | N/A |
| Ustekinumab | N/A | MODERATE | MODERATE | N/A | N/A | N/A |
| Vedolizumab | N/A | MODERATE | MODERATE | N/A | N/A | N/A |
| VDZ-SC | N/A | MODERATE | MODERATE | N/A | N/A | N/A |
| Placebo | MODERATE^a^ | LOW | MODERATE^a^ | N/A | N/A | N/A |
| Compared with IFX-10 | | | | | | |
| Infliximab+azathioprine | N/A | VERY LOW^d^ | VERY LOW^d^ | N/A | N/A | N/A |
| Adalimumab | N/A | LOW | LOW | N/A | N/A | N/A |
| Adalimumab+azathioprine | N/A | VERY LOW^d,e^ | VERY LOW^d,e^ | N/A | N/A | N/A |
| CT-P13 | N/A | VERY LOW^d^ | VERY LOW^d^ | N/A | N/A | N/A |
| Ustekinumab | N/A | LOW | LOW | N/A | N/A | N/A |
| Vedolizumab | N/A | LOW | LOW | N/A | N/A | N/A |
| VDZ-SC | N/A | LOW | LOW | N/A | N/A | N/A |
| Placebo | LOW^a,c^ | LOW | LOW^a,c^ | N/A | N/A | N/A |
| Compared with Infliximab+azathioprine | | | | | | |
| Adalimumab | N/A | VERY LOW^d,e^ | VERY LOW^d,e^ | N/A | N/A | N/A |
| Adalimumab+azathioprine | N/A | VERY LOW^d,e^ | VERY LOW^d,e^ | N/A | N/A | N/A |
| CT-P13 | N/A | VERY LOW^d^ | VERY LOW^d^ | N/A | N/A | N/A |
| Ustekinumab | N/A | VERY LOW^d,e^ | VERY LOW^d,e^ | N/A | N/A | N/A |
| Vedolizumab | N/A | VERY LOW^d,e^ | VERY LOW^d,e^ | N/A | N/A | N/A |
| VDZ-SC | N/A | VERY LOW^d,e^ | VERY LOW^d,e^ | N/A | N/A | N/A |
| Placebo | N/A | LOW^d^ | LOW^d^ | N/A | N/A | N/A |
| Compared with Adalimumab | | | | | | |
| Adalimumab+azathioprine | LOW^a,c^ | N/A | LOW^a,c^ | N/A | N/A | N/A |
| CT-P13 | N/A | VERY LOW^d,e^ | VERY LOW^d,e^ | N/A | N/A | N/A |
| Ustekinumab | MODERATE | MODERATE | MODERATE | N/A | MODERATE | MODERATE |
| Vedolizumab | N/A | MODERATE | MODERATE | N/A | MODERATE | MODERATE |
| VDZ-SC | N/A | MODERATE | MODERATE | N/A | MODERATE | MODERATE |
| Placebo | MODERATE^a^ | LOW^d^ | MODERATE^a^ | MODERATE^a^ | N/A | MODERATE^a^ |
| Compared with Adalimumab+azathioprine | | | | | | |
| CT-P13 | N/A | VERY LOW^d,e^ | VERY LOW^d,e^ | N/A | N/A | N/A |
| Ustekinumab | N/A | VERY LOW^d^ | VERY LOW^d^ | N/A | N/A | N/A |
| Vedolizumab | N/A | VERY LOW^d,e^ | VERY LOW^d,e^ | N/A | N/A | N/A |
| VDZ-SC | N/A | VERY LOW^d,e^ | VERY LOW^d,e^ | N/A | N/A | N/A |
| Placebo | N/A | VERY LOW^d^ | VERY LOW^d^ | N/A | N/A | N/A |
| Compared with CT-P13 | | | | | | |
| Ustekinumab | N/A | VERY LOW^d,e^ | VERY LOW^d,e^ | N/A | N/A | N/A |
| Vedolizumab | N/A | VERY LOW^d,e^ | VERY LOW^d,e^ | N/A | N/A | N/A |
| VDZ-SC | N/A | VERY LOW^d,e^ | VERY LOW^d,e^ | N/A | N/A | N/A |
| Placebo | N/A | LOW^d^ | LOW^d^ | N/A | N/A | N/A |
| Compared with Ustekinumab | | | | | | |
| Vedolizumab | N/A | MODERATE | MODERATE | N/A | MODERATE | MODERATE |
| VDZ-SC | N/A | MODERATE | MODERATE | N/A | MODERATE | MODERATE |
| Placebo | MODERATE^a^ | LOW^d^ | MODERATE^a^ | MODERATE^a^ | N/A | MODERATE^a^ |
| Compared with Vedolizumab | | | | | | |
| VDZ-SC | N/A | MODERATE | MODERATE | N/A | MODERATE | MODERATE |
| Placebo | MODERATE^a^ | N/A | MODERATE^a^ | MODERATE^a^ | N/A | MODERATE^a^ |
| Compared with VDZ-SC | | | | | | |
| Placebo | MODERATE^a^ | N/A | MODERATE^a^ | MODERATE^a^ | N/A | MODERATE^a^ |

a. Rated down for imprecision since optimal information size not met (<200 events).

b. Rated down for publication bias strongly suspected.

c. Rated down for serious risk of bias.

d. Rated down for indirectness due to intransitivity from different patient history of TNF antagonist treatment.

e. Evidence derived from second/higher-order loops, rated down for very serious indirectness.

**Supplementary Figure 41.** Flow diagram of selection.

16,252 records identified from:

PubMed (n=6,419)

Web of Science (n=3,065)

CENTRAL (n=583)

Embase (n=5,556)

ClinicalsTrials (n=250)

ICTRP-45

Scopus-334

13,038 records after 3,214 duplicates removed

12,935 records excluded based on title and abstract

103 of full-text articles evaluated for eligibility

75 of studies excluded for reasons:

1. fistulizing Crohn’s disease (n=1)

2. no results available (n=4)

3. not recruiting (n=6)

4. evaluation of treatment algorithms (n=20)

5. non-randomization (n=4)

6. no clear definition of Crohn’s disease severity (n=2)

7. non-controlled (n=1)

8. postoperative patients (n=13)

9. uncompleted (n=7)

10. no full-text (n=9)

11. different definitions of outcomes (n=3)

12. non-approved dose (n=5)

31 clinical trials in 28 studies records included quantitative synthesis

**Supplementary Table 7:** Characteristics of included randomized controlled trials comparing biologic agents for induction therapy in patients with moderate-to-severe Crohn’s disease.

| Study | Country; number of sites or centers | Sample size/TNF antagonist-naïve; total | Trial design | Dosage and schedule of included active/comparator group | severity of CD at randomization | Definition of outcomes | Outcome timepoints | concomitant medications | |
| --- | --- | --- | --- | --- | --- | --- | --- | --- | --- |
|  |  |  |  |  |  |  |  | Active | Placebo or Comparator |
| **Infliximab** | | | | | | | | | |
| Targan SR et al, 1997 | North America and Europe; 18 | 108; 108 | Patients were randomly assigned to receive either placebo i.v. or IFX 5 mg/kg i.v., IFX 10 mg/kg i.v., or IFX 20 mg/kg i.v. at week 0. | IFX 5 mg/kg i.v. | CDAI 220-400 | CRM and CDAI-70 | week 4 | 5-ASA: 47 (56.6%) IMM: 29 (34.9%) CS: 48 (57.8%) | 5-ASA: 17 (68.0%) IMM: 11 (44.0%) CS: 16 (64.0%) |
| Colombel JF et al, SONIC, 2010 | North America, Europe and Israel, etc.; 92 | 508; 508 | Patients were randomly assigned to receive either IFX 5 mg/kg i.v. at weeks 0, 2, 6 and then E8W, AZA p.o. 2.5 mg/kg daily, or combination, followed through week 30 with blinded 20-week extension trial through week 50. | IFX 5 mg/kg i.v. at weeks 0, 2, 6 and then E8W | CDAI 220-450 | CRM, CDAI-70 and CDAI-100 | week 6 | 5-ASA: 85 (50.3%) CS: 47 (27.8%) | 5-ASA: 87 (51.5%)  CS: 52 (30.8%) |
| Ye BD et al, 2019 | The United States, Russia and Germany, etc.; 58 | 220; 220 | Patients were randomly assigned to receive CT-P13 (5 mg/kg i.v. at weeks 0, 2, 6 and then E8W up to week 54) followed by CT-P13 at week 30; CT-P13 followed by IFX (5 mg/kg i.v. at weeks 0, 2, 6 and then E8W up to week 54) at week 30; IFX followed by IFX at week 30; IFX followed by CT-P13 at week 30. | IFX or CT-P13 5 mg/kg i.v. at weeks 0, 2, 6 and then E8W up to week 54 | CDAI 220-450 | CRM, CDAI-70 and CDAI-100 | week 6 | IMM: 80 (73.4%) CS: 33 (30.3%) | IMM: 84 (75.7%) CS: 37 (33.3%) |
| **Adalimumab** | | | | | | | | | |
| Hanauer SB et al, CLASSIC-1, 2006 | The United States, Canada and Europe, etc.; 55 | 299; 299 | Patients were randomly assigned to receive placebo, ADA 40/20 mg s.q., ADA 80/40 mg s.q., or ADA 160/80 mg at week 0/2, and followed until week 4. | ADA 160/80 mg s.q. at week 0/2, and followed until week 4 | CDAI 220-450 | CRM, CDAI-70 and CDAI-100 | week 4 | 5-ASA: 116 (51.6%) IMM: 66 (29.3%) CS: 73 (32.4%) | 5-ASA: 37 (50.0%) IMM: 22 (29.7%) CS: 25 (33.8%) |
| Matsumoto T et al, DIAMOND, 2016 | Japan; N/A | 176; 176 | Patients were randomly assigned to received either ADA s.q. 160/80 mg at weeks 0/2, and thereafter 40 mg at E2W up to 52 weeks, with or without combination AZA 25 mg or 50 mg/day (the dose was allowed to be increased to a maximum of 100 mg during the initial four weeks). | ADA s.q. 160/80 mg at weeks 0/2, and thereafter 40 mg at E2W up to 52 weeks combined with AZA 25 mg or 50 mg/day | CDAI ≥220 | CRM and CDAI-70 | week 4 | 5-ASA: 59 (64.8%) CS: 5 (5.5%) | 5-ASA: 64 (75.3%) CS: 13 (15.3%) |
| Watanabe M et al, 2011 | Japan; N/A | 38; 90 | Patients were randomly assigned to received ADA 160/80 mg, ADA 80/40 mg, or placebo at baseline and week 2. | ADA 160/80 mg s.q. at baseline and week 2 | CDAI 220-450 | CRM, CDAI-70 and CDAI-100 | week 4 | 5-ASA: 59 (88.1%) IMM: 21 (31.3%) CS: 14 (20.9%) | 5-ASA: 23 (100.0%) IMM: 8 (34.8%) CS: 5 (21.7%) |
| Chen B et al, 2020 | China; 15 | 205; 205 | Patients were randomly assigned to received ADA 160/80 mg at weeks 0/2 and then ADA 40 mg E2W, or placebo at weeks 0/2 and then ADA 160/80 mg at weeks 4/6, followed through week 8 with open-label (ADA 40 mg E2W) trial through week 26. | ADA 160/80 mg at weeks 0/2 | CDAI 220-450 | CRM and CDAI-70 | week 4 | IMM: 61 (59.8%) CS: 31 (30.4%) | IMM: 65 (63.1%) CS: 32 (31.1%) |
| Hanauer S et al, VOLTAIRE-CD, 2021 | The United States and Europe, etc.; 92 | 128; 140 | Patients were randomly assigned to received BI 695501 (or ADA) 160/80 mg s.q. on days 1/15, followed by 40 mg E2W, until week 24. | ADA or BI 695501 160/80 mg on days 1/15 | CDAI 220-450 | CRM, CDAI-70 and CDAI-100 | week 4 | N/A | N/A |
| Sandborn WJ et al, GAIN, 2007 | The United States, Canada, Belgium, France; 52 | 0; 325 | Patients were randomly assigned to received either ADA 160/80 s.q. at weeks 0/2, or placebo through week 4. | ADA 160/80 mg s.q. at weeks 0/2 through week 4 | CDAI 220-450 | CRM, CDAI-70 and CDAI-100 | week 4 | 5-ASA: 45 (28.3%) IMM: 73 (45.9%) CS: 55 (34.6%) | 5-ASA: 60 (36.1%) IMM: 85 (51.2%) CS: 73 (44.0%) |
| **Certolizumab Pegol** | | | | | | | | | |
| Sandborn WJ et al, 2011 | Multinational; 120 | 438; 438 | Patients were randomly assigned to received CZP 400 mg s.q. or placebo at weeks 0, 2, 4. | CZP 400 mg s.q. at weeks 0, 2, 4 | CDAI 220-450 | CRM and CDAI-100 | week 6 | IMM: 77 (34.5%) CS: 97 (43.5%) | IMM: 67 (31.2%) CS: 98 (45.6%) |
| Sandborn WJ et al, PRECISE-1, 2007 | Multinational; 171 | 474; 659 | Patients were randomly assigned to received CZP 400 mg s.q. or placebo at weeks 0, 2, 4, and then every 4 weeks until week 26. | CZP 400 mg s.q. at weeks 0, 2, 4, and then every 4 weeks | CDAI 220-450 | CRM, CDAI-70 and CDAI-100 | week 6 | IMM: 126 (38.1%) CS: 129 (39.0%) | IMM: 121 (37.0%) CS: 130 (39.6%) |
| **Ustekinumab** | | | | | | | | | |
| Sandborn WJ et al, CERTIFI, 2012 | The United States, Britain, Australia, etc.; 153 | 0; 526 | Patients were randomly assigned to received either UST 1, 3, 6 mg/kg i.v. or placebo at week 0 to week 8. | UST 6 mg/kg i.v. at week 0 to week 8 | CDAI 220-450 | CRM, CDAI-70 and CDAI-100 | week 6 | 5-ASA: 67 (17.0%) IMM: 96 (24.4%) CS: 189 (48.0%) | 5-ASA: 24 (18.2%) IMM: 30 (22.7%) CS: 73 (55.3%) |
| Sands BE et al, SEAVUE, 2022 | Multinational; 121 | 386; 386 | Patients were randomly assigned to received either UST 6 mg/kg i.v. on day 0, then 90 mg s.q. E8W through week 56, with placebo s.q. once E2W, or ADA 160 mg s.q. plus placebo s.q. on day 0, 80 mg s.q. at week 2, then 40 mg s.q. E2W through week 56. | UST 6 mg/kg i.v. on day 0 | CDAI 220-450 | CRM and CDAI-100 | week 8 | CS: 70 (36.6%) | CS: 75 (38.5%) |
| Feagan BG et al, UNITI-1, 2016 | Multinational; 178 | 0; 741 | Patients were randomly assigned to received UST 130 mg i.v., UST 6 mg/kg i.v., or placebo at week 0. | UST 6 mg/kg i.v. at week 0 | CDAI 220-450 | CRM, CDAI-70 and CDAI-100 | week 6 | 5-ASA: 100 (20.2%) IMM: 152 (30.8%) CS: 229 (46.4%) | 5-ASA: 54 (21.9%) IMM: 81 (32.8%) CS: 111 (44.9%) |
| Feagan BG et al, UNITI-2, 2016 | Multinational; 175 | 427; 628 | Patients were randomly assigned to received UST 130 mg i.v., UST 6 mg/kg i.v., or placebo at week 0. | UST 6 mg/kg i.v. at week 0 | CDAI 220-450 | CRM, CDAI-70 and CDAI-100 | week 6 | 5-ASA: 182 (43.5%) IMM: 146 (34.9%) CS: 172 (41.1%) | 5-ASA: 89 (42.4%) IMM: 73 (34.8%) CS: 75 (35.7%) |
| **Risankizumab** | | | | | | | | | |
| Feagan BG et al, 2017 | North America, Europe, and southeast Asia, etc.; 36 | 0; 121 | Patients received either RZB 200 mg i.v., RZB 600 mg i.v., or placebo at weeks 0, 4, and 8. | RZB 600 mg i.v. at weeks 0, 4, and 8 | CDAI 220-450 | CRM and CDAI-100 | week 4 | IMM: 8 (19.5%) CS: 12 (29.3%) | IMM: 13 (33.3%) CS: 11 (28.2%) |
| D'Haens G et al, ADVANCE, 2022 | Multinational; 297 | N/A; 850 | Patients were randomly assigned to receive RZB 600 mg i.v., RZB 1200 mg i.v., or placebo at weeks 0, 4, and 8. | RZB 600 mg i.v. at weeks 0, 4, and 8 | CDAI 220-450 | CRM and CDAI-100 | week 4 | IMM: 88 (26.2%) CS: 102 (30.4%) | IMM: 42 (24.0%) CS: 50 (28.5%) |
| D'Haens G et al, MOTIVATE, 2022 | Multinational; 214 | N/A; 569 | Patients were randomly assigned to receive RZB 600 mg i.v., RZB 1200 mg i.v., or placebo at weeks 0, 4, and 8. | RZB 600 mg i.v. at weeks 0, 4, and 8 | CDAI 220-450 | CRM and CDAI-100 | week 4 | IMM: 36 (18.8%) CS: 65 (34.0%) | IMM: 40 (21.4%) CS: 68 (36.4%) |
| **Vedolizumab** | | | | | | | | | |
| Sandborn WJ et al, GEMINI-2, 2013 | Multinational; 285 | 185; 368 | Patients were randomly assigned (in a 3:2 ratio) to received VDZ 300 mg i.v. or placebo at weeks 0, 2, and were followed through week 6. | received VDZ 300 mg i.v. at weeks 0, 2, and were followed through week 6 | CDAI 220-450 | CRM and CDAI-100 | week 6 | IMM: 156 (16.1%) CS: 336 (34.7%) | IMM: 25 (16.9%) CS: 45 (30.4%) |
| Sands BE et al, GEMINI-3. 2014 | North America, Europe, Asia, Africa and Australia, etc.; 107 | 315; 416 | Patients were randomly assigned to received VDZ 300 mg i.v. or placebo at weeks 0, 2, and 6. | VDZ 300 mg i.v. at weeks 0, 2, and 6 | CDAI 220-400 | CRM and CDAI-100 | week 6 | 5-ASA: 68 (32.5%) IMM: 71 (34.0%) CS: 110 (52.6%) | 5-ASA: 61 (29.5%) IMM: 69 (33.3%) CS: 108 (52.2%) |
| Watanabe K et al, 2020 | Japan; 77 | 34; 157 | Patients were randomly assigned to receive placebo or VDZ 300 mg i.v. at weeks 0, 2, and 6. | VDZ 300 mg i.v. at weeks 0, 2, and 6 | CDAI 220-450 | CRM and CDAI-100 | week 6 | 5-ASA: 64 (81.0%) IMM: 36 (45.6%) CS: 22 (27.8%) | 5-ASA: 59 (75.6%) IMM: 40 (51.3%) CS: 18 (23.1%) |
| Vedolizumab-3034, 2022 | China; 27 | 94; 214 | Patients were randomly assigned (in a 1:2 ratio) to receive placebo or VDZ 300 mg i.v. at weeks 0, 2, and 6. | VDZ 300 mg i.v. at weeks 0, 2, and 6 | CDAI 220-400 | CRM and CDAI-100 | week 10 | IMM: 59 (41.0%) CS: 14 (9.7%) | IMM: 23 (32.9%) CS: 5 (7.1%) |
| **Abbreviations:** 5-ASA-5-aminosalicylates, ADA-Adalimumab, CDAI-70/100-clinical response defined by a reduction in the CDAI ≥70/100 points compared to baseline, CDAI-Crohn's Disease Activity Index, CD-Crohn's disease, CRM-clinical remission defined by a Crohn's Disease Activity Index < 150, CS-corticosteroids, CZP-Certolizumab Pegol, ExW-every x weeks (x refers to a specific number of weeks), i.v.-intravenous, IFX-Infliximab, IMM-immunosuppressants, p.o.-per oral, RZB-Risankizumab, s.q.-subcutaneous, TNF-tumor necrosis factor, UST-Ustekinumab, VDZ-Vedolizumab. | | | | | | | | | |

**Supplementary Table 8:** Characteristics of included randomized controlled trials comparing biologic agents for maintenance therapy in patients with moderate-to-severe Crohn’s disease.

| Study | Country; number of sites or centers | Sample size/TNF antagonist-naïve; total | Trial design | Dosage and schedule of included active/comparator group | severity of CD at randomization | Definition of outcomes | Outcome timepoints | concomitant medications | | |
| --- | --- | --- | --- | --- | --- | --- | --- | --- | --- | --- |
|  |  |  |  |  |  |  |  | Active | | Placebo or Comparator |
| **Infliximab** | | | | | | | | | | |
| Hanauer SB et al, ACCENT-1, 2002 | North America, Europe and Israel, etc.; 55 | 335; 335 | Patients received a IFX 5 mg/kg i.v. at week 0; patients with response were randomly assigned subsequent infusions, at weeks 2 and 6 and 8 weeks thereafter until week 46, of placebo, IFX 5 mg/kg i.v., or IFX 5 mg/kg i.v. at weeks 2 and 6 followed by 10 mg/kg thereafter. | IFX 5 mg/kg i.v. or IFX 10 mg/kg i.v. | decrease in CDAI score of 70 points or more from the baseline value and at least a 25% reduction in the total score in the reduction | CRM and CDAI-70 | week 54 | 5-ASA: 159 (47.5%) IMM: 91 (27.2%) CS: 175 (52.2%) | | |
| Colombel JF et al, SONIC, 2010 | North America, Europe and Israel, etc.; 92 | 508; 508 | Patients were randomly assigned to receive either IFX 5 mg/kg i.v. at weeks 0, 2, 6 and then E8W, AZA p.o. 2.5 mg/kg daily, or combination, followed through week 30 with blinded 20-week extension trial through week 50. | IFX 5 mg/kg i.v. at weeks 0, 2, 6 and then E8W combined with AZA p.o. 2.5 mg/kg daily | CDAI 220-450 | CRM, CDAI-70 and CDAI-100 | week 50 | 5-ASA: 85 (50.3%) CS: 47 (27.8%) | | 5-ASA: 87 (51.5%)  CS: 52 (30.8%) |
| Ye BD et al, 2019 | The United States, Russia and Germany, etc.; 58 | 220; 220 | Patients were randomly assigned to receive CT-P13 (5 mg/kg i.v. at weeks 0, 2, 6 and then E8W up to week 54) followed by CT-P13 at week 30; CT-P13 followed by IFX (5 mg/kg i.v. at weeks 0, 2, 6 and then E8W up to week 54) at week 30; IFX followed by IFX at week 30; IFX followed by CT-P13 at week 30. | CT-P13 5 mg/kg i.v. E8W up to week 30 | CDAI 220-450 | CRM, CDAI-70 and CDAI-100 | week 30 | IMM: 80 (73.4%) CS: 33 (30.3%) | | IMM: 84 (75.7%) CS: 37 (33.3%) |
| Rutgeerts P et al, 1999 | North America, Europe, etc.; 17 | 73; 73 | All patients received initial treatment with placebo, IFX 5, 10, or 20 mg/kg. Patients responded at week 8 of initial treatment were randomly assigned to retreatment with IFX 10 mg/kg i.v. or placebo at week 12. All patients, regardless of treatment group, were receive 4 infusions at weeks 12, 20, 28, and 36. | IFX 10 mg/kg i.v. E8W | decrease in CDAI score of 70 points or more from the baseline value in the reduction | CRM and CDAI-70 | week 48 | N/A | | N/A |
| **Adalimumab** | | | | | | | | | | |
| Colombel JF et al, CHARM, 2007 | Europe, the United States, Canada, Australia and South Africa, etc.; 92 | 261; 499 | All patients received open-label ADA 80 mg s.q. followed by a 40-mg dose at week 2. Patients were randomized to one of 3 treatment groups (ADA 40 mg s.q. E2W, ADA 40 mg s.q. weekly, or placebo) and continued treatment through week 56. Patients were stratified by responder status and previous exposure to TNF antagonists at week 4. | ADA 40 mg s.q. E2W | decrease in CDAI score of 70 points or more from the baseline value in the reduction | CRM, CDAI-70 and CDAI-100 | week 56 | 5-ASA: 206 (41.3%) IMM: 240 (48.1%) CS: 210 (42.1%) | | |
| Sandborn WJ et al, CLASSIC-2, 2007 | North America, Europe, etc.; 53 | 276; 276 | Patients were randomly assigned to receive blinded maintenance treatment with ADA 40 mg s.q. E2W, ADA 40 mg weekly, or placebo from weeks 4 to 55. | ADA 40 mg s.q. E2W | durable clinical remission (defined as CDAI score of less than 150 points) at both weeks 0 and 4 | CRM, CDAI-70 and CDAI-100 | week 56 | 5-ASA: 26 (70.3%) IMM: 9 (24.3%) CS: 17 (45.9%) | | 5-ASA: 8 (44.4%) IMM: 3 (16.7%) CS: 10 (55.6%) |
| Matsumoto T et al, DIAMOND, 2016 | Japan; N/A | 176; 176 | Patients were randomly assigned to receive either ADA s.q. 160/80 mg at weeks 0/2, and thereafter 40 mg at E2W up to 52 weeks, with or without combination AZA 25 mg or 50 mg/day (the dose was allowed to be increased to a maximum of 100 mg during the initial four weeks). | ADA s.q. 160/80 mg at weeks 0/2, and thereafter 40 mg at E2W up to 52 weeks combined with AZA 25 mg or 50 mg/day | CDAI ≥220 | CRM and CDAI-70 | week 52 | 5-ASA: 59 (64.8%) CS: 5 (5.5%) | | 5-ASA: 64 (75.3%) CS: 13 (15.3%) |
| Rutgeerts P et al, EXTEND, 2012 | Europe, the United States, Canada, etc; 19 | 62; 129 | Patients received ADA 160 mg s.q. at week 0 and 80 mg s.q. at week 2. At week 4, clinical response was assessed and patients were stratified by responder status. All patients were randomized to maintenance therapy with ADA 40 mg E2W or placebo. | ADA 40 mg s.q. E2W | CDAI 220-450 | CRM, CDAI-70 and CDAI-100 | week 52 | 5-ASA: 9 (14.1%) IMM: 28 (43.8%) CS: 9 (14.1%) | | 5-ASA: 19 (29.2%) IMM: 25 (38.5%) CS: 25 (38.5%) |
| Watanabe M et al, 2011 | Japan; N/A | 23; 50 | Patients were randomly assigned to receive ADA 160/80 mg, ADA 80/40 mg, or placebo at baseline and week 2. Patients achieving CDAI-70 at week 4 were randomly assigned to receive ADA 40 mg E2W or placebo. | ADA 40 mg s.q. E2W | decrease in CDAI score of 70 points or more from the baseline value in the reduction | CRM, CDAI-70 and CDAI-100 | week 52 | 5-ASA: 25 (100.0%) IMM: 11 (44.0%) CS: 3 (12.0%) | | 5-ASA: 19 (76.0%) IMM: 7 (28.0%) CS: 5 (20.0%) |
| Hanauer S et al, VOLTAIRE-CD, 2021 | The United States and Europe, etc.; 92 | 128; 140 | Patients were randomly assigned to receive BI 695501 (or ADA) 160/80 mg s.q. on days 1/15, followed by 40 mg E2W, until week 24. | ADA 40 mg E2W, until week 24 | CDAI 220-450 | CRM, CDAI-70 and CDAI-100 | week 24 | N/A | | N/A |
| **Certolizumab Pegol** | | | | | | | | | | |
| Sandborn WJ et al, PRECISE-1, 2007 | Multinational; 171 | 474; 659 | Patients were randomly assigned to receive CZP 400 mg s.q. or placebo at weeks 0, 2, 4, and then every 4 weeks until week 26. | CZP 400 mg s.q. at weeks 0, 2, 4, and then every 4 weeks | CDAI 220-450 | CRM, CDAI-70 and CDAI-100 | week 26 | IMM: 126 (38.1%) CS: 129 (39.0%) | | IMM: 121 (37.0%) CS: 130 (39.6%) |
| Schreiber S et al, PRECISE-2, 2007 | Multinational; 147 | 322; 425 | Patients received CZP 400 mg s.q. at weeks 0, 2, 4. Patients who had a CDAI-100 at week were randomly assigned to receive CZP 400 mg s.q. or placebo at weeks 8, 12, 16, 20, and 24 and were followed through week 26. | CZP 400 mg s.q. at weeks 0, 2, 4, and then every 4 weeks | decrease in CDAI score of 100 points or more from the baseline value in the reduction | CRM and CDAI-100 | week 26 | IMM: 87 (40.5%) CS: 75 (34.9%) | | IMM: 86 (41.0%) CS: 78 (37.1%) |
| **Ustekinumab** | | | | | | | | | | |
| Sandborn WJ et al, CERTIFI, 2012 | The United States, Britain, Australia, etc; 153 | 0; 145 | Patients were randomly assigned to receive UST i.v. in doses of 1, 3, or 6 mg/kg or placebo. Patients who had a response to UST at week 8 underwent randomization to receive UST 90 mg s.q. or placebo at weeks 8 and 16, with efficacy assessed at week 22. | UST 90 mg s.q. at weeks 8 and 16 | decrease in CDAI score of 100 points or more from the baseline value in the reduction | CRM and CDAI-100 | week 22 | N/A | | N/A |
| Sands BE et al, SEAVUE, 2022 | Multinational; 121 | 386; 386 | Patients were randomly assigned to receive either UST 6 mg/kg i.v. on day 0, then 90 mg s.q. E8W through week 56, with placebo s.q. once E2W, or ADA 160 mg s.q. plus placebo s.q. on day 0, 80 mg s.q. at week 2, then 40 mg s.q. E2W through week 56. | ADA 160/80 mg s.q. at week 0/2, then 40 mg s.q. E2W through week 56 UST 6 mg/kg i.v. on day 0, then 90 mg s.q. E8W through week 56 | CDAI 220-450 | CRM and CDAI-100 | week 52 | CS: 70 (36.6%) | | CS: 75 (38.5%) |
| Feagan BG et al, IM-UNITI, 2016 | Multinational; 260 | 157; 397 | Patients who had a response to UST at week 8 were randomly assigned to receive UST 90 mg s.q. E8W, UST 90 mg s.q. E12W, or placebo through week 40. | UST 90 mg s.q. E8W through week 40 | decrease in CDAI score of 100 points or more from the baseline value in the reduction | CRM and CDAI-100 | week 44 | 5-ASA: 96 (36.4%) IMM: 96 (36.4%) CS: 122 (46.2%) | | 5-ASA: 46 (34.6%) IMM: 47 (35.3%) CS: 59 (44.4%) |
| **Risankizumab** | | | | | | | | | | |
| Ferrante M et al, FORTIFY, 2022 | Multinational; 285 | N/A; 462 | Patients with CDAI-100 in the ADVANCE or MOTIVATE induction studies were randomly assigned to receive RZB 180 mg s.q., RZB 360 mg s.q., or placebo E8W. | RZB 180 mg s.q. E8W RZB 360 mg s.q. E8W | decrease in CDAI score of 100 points or more from the baseline value in the reduction | CRM and CDAI-100 | week 52 | 180 mg RZB/ IMM: 41 (26.1%) CS: 51 (32.5%) | 360 mg RZB/ IMM: 40 (28.4%) CS: 42 (30.0%) | IMM: 40 (24.4%) CS: 51 (31.1%) |
| **Vedolizumab** | | | | | | | | | | |
| Sandborn WJ et al, GEMINI-2, 2013 | Multinational; 285 | 208; 461 | Patients were randomly assigned (in a 3:2 ratio) to receive VDZ 300 mg i.v. or placebo at weeks 0, 2, and were followed through week 6 (cohort 1). Patients from cohort 1 and cohort 2 (an open-label group) who had a clinical response with VDZ at week 6 were randomly assigned to receive VDZ E8W, VDZ E4W, or placebo, for up to 52 weeks. | VDZ 300 mg i.v. E8W for up to 52 weeks | decrease in CDAI score of 70 points or more from the baseline value in the reduction | CRM and CDAI-100 | week 52 | IMM: 103(33.4%)  CS: 162 (52.6%) | | IMM: 49 (32.0%)  CS: 82 (53.6%) |
| Vermeire S et al, VISIBLE-2, 2022 | 30 countries in America, Europe and East Asia; 169 | 170; 409 | All patients received open-label VDZ 300 i.v. at weeks 0 and 2. Patients who responded to VDZ 300 mg i.v. at week 6 were randomized to receive VDZ 108 mg s.q. or placebo, E2W at week 6 and continuing through week 50. | VDZ 108 mg s.q. at week 6 and continuing through week 50 | decrease in CDAI score of 70 points or more from the baseline value in the reduction | CRM and CDAI-100 | week 52 | IMM: 82 (29.8%) CS: 95 (34.5%) | | IMM: 47 (35.1%) CS: 44 (32.8%) |
| Watanabe K et al, 2020 | Japan; 77 | 9; 24 | Patients were randomly assigned to receive placebo or VDZ 300 mg i.v. at weeks 0, 2, and 6. VDZ-treatment patients who achieved a reduction in CDAI-70 at week 10 were randomized to receive placebo or VDZ 300 mg, then E8W until week 54. | VDZ 300 mg i.v. E8W until week 54 | decrease in CDAI score of 70 points or more from the baseline value in the reduction | CRM and CDAI-100 | week 60 | 5-ASA: 8 (66.7%) IMM: 9 (75.0%) CS: 5 (41.7%) | | 5-ASA: 11 (91.7%) IMM: 6 (50.0%) CS: 3 (25%) |
| Vedolizumab-3034, 2022 | China; 27 | 21; 56 | Patients were randomly assigned (in a 1:2 ratio) to receive placebo or VDZ 300 mg i.v. at weeks 0, 2, and 6. Patients who achieved clinical response at week 10 with placebo received placebo, achieved clinical response with VDZ received 300 mg E8W. | VDZ 300 mg i.v. E8W at week 10 | decrease in CDAI score of 70 points or more from the baseline value in the reduction | CRM and CDAI-100 | week 60 | IMM: 20 (43.4%) CS: 7 (15.2%) | | IMM: 7 (38.9%) CS: 2 (11.1%) |
| **Abbreviations:** 5-ASA-5-aminosalicylates, ADA-Adalimumab, CDAI-70/100-clinical response defined by a reduction in the CDAI ≥70/100 points compared to baseline, CDAI-Crohn's Disease Activity Index, CD-Crohn's disease, CRM-clinical remission defined by a Crohn's Disease Activity Index < 150, CS-corticosteroids, CZP-Certolizumab Pegol, ExW-every x weeks (x refers to a specific number of weeks), i.v.-intravenous, IFX-Infliximab, IMM-immunosuppressants, p.o.-per oral, RZB-Risankizumab, s.q.-subcutaneous, TNF-tumor necrosis factor, UST-Ustekinumab, VDZ-Vedolizumab. | | | | | | | | | | |

**Supplementary Result 1.** Result of evaluation of induction for clinical remission in moderate-to-severe CD patients.

In the evaluation of inducing clinical remission in moderate-to-severe CD patients, data point for the Ustekinumab arm of the CERTIFI trial in the leverage plot (lay outside the purple dotted line) may contribute to the model’s poor fit. A sensitivity analysis was conducted excluding the CERTIFI trial in this evaluation. All but Vedolizumab (OR 1.49 [95% CI: 0.96, 2.30]) were significantly superior to placebo on direct meta-analysis, and only Adalimumab had a significant superiority over Adalimumab in combination with azathioprine (OR 2.07 [95% CI: 1.07, 4.02]) in the comparison of active agent regimens. On network meta-analysis, Infliximab in combination with azathioprine (SUCRA 92.26), Infliximab (SUCRA 88.62), and CT-P13 (SUCRA 85.23) were ranked highest.

**Supplementary Result 2.** Result of evaluation of maintenance for clinical response in moderate-to-severe CD patients.

In the evaluations of maintaining clinical response in moderate-to-severe CD patients, the results of funnel plot evaluation, model fit evaluation and model convergence evaluation were all within appropriate and controllable range. On direct meta-analysis, all agents except Ustekinumab, Vedolizumab, and Risankizumab, which CDAI-70 was not evaluated in included RCTs, were significantly superior to placebo for maintaining CDAI-70. In the evaluation of maintaining CDAI-100, all but Vedolizumab (OR 2.11 [95% CI: 0.98, 4.53]) and Vedolizumab SC (OR 1.34 [95% CI: 0.88, 2.02]) were significantly superior to placebo. Comparisons between active agent regimens did not show significant differences in either the maintenance CDAI-70 and CDAI-100 evaluations. On network meta-analysis, IFX-10 (SUCRA 69.81), Adalimumab (SUCRA 65.89), Infliximab in combination with azathioprine (SUCRA 64.94) were ranked highest for maintenance CDAI-70, and Adalimumab (SUCRA 80.41), Ustekinumab (SUCRA 74.71), Vedolizumab (SUCRA 55.94) ranked highest for maintenance CDAI-100.

**Supplementary Result 3.** Result of evaluation of induction for clinical remission in TNF antagonists-naïve CD patients.

In the evaluation of inducing clinical remission in TNF antagonists-naïve CD patients, all agents except Certolizumab Pegol (OR 1.36 [95% CI: 0.89, 2.08]) were significantly superior to placebo on direct meta-analysis. There was no benefit of Infliximab alone over either CT-P13 (OR 1.11 [95% CI: 0.65, 1.90]) or in combination with azathioprine (OR 0.89 [95% CI: 0.58, 1.36]), and no benefit of Ustekinumab over Adalimumab (OR 1.06 [95% CI: 0.71, 1.58]). On network meta-analysis, Infliximab in combination with azathioprine (SUCRA 86.68), Infliximab alone (SUCRA 83.48), and CT-P13 (SUCRA 79.32) were ranked highest, followed by Ustekinumab (SUCRA 56.78), Adalimumab alone (SUCRA 55.72), Vedolizumab (SUCRA 35.18), Adalimumab in combination with azathioprine (SUCRA 31.92), and Certolizumab Pegol (SUCRA 15.88).

**Supplementary Result 4.** Result of evaluation of induction for clinical remission in TNF antagonists-experienced CD patients.

In the evaluation of inducing clinical remission in TNF antagonists-experienced CD patients, only Adalimumab had a significant superiority over placebo (OR 3.55 [95% CI: 1.82, 6.93]) on direct meta-analysis. On network meta-analysis, Adalimumab (SUCRA 84.30) and Risankizumab (SUCRA 72.45) were ranked highest, followed by Ustekinumab (SUCRA 50.70) and Vedolizumab (SUCRA 30.62).
